# Clonal relationships between lobular carcinoma in situ and other breast malignancies

**DOI:** 10.1186/s13058-016-0727-z

**Published:** 2016-06-23

**Authors:** Colin B. Begg, Irina Ostrovnaya, Jose V. Scarpa Carniello, Rita A. Sakr, Dilip Giri, Russell Towers, Michail Schizas, Marina De Brot, Victor P. Andrade, Audrey Mauguen, Venkatraman E. Seshan, Tari A. King

**Affiliations:** Department of Epidemiology and Biostatistics, Memorial Sloan Kettering Cancer Center, 1275 York Avenue, New York, NY 10065 USA; Department of Surgery, Memorial Sloan Kettering Cancer Center, 1275 York Avenue, New York, NY 10065 USA; Department of Pathology, Memorial Sloan Kettering Cancer Center, 1275 York Avenue, New York, NY 10065 USA; Department of Pathology, Federal University of Minas Gerais, Avenida Presidente Antônio Carlos, 6627 - Pampulha, Belo Horizonte, MG 31270-901 Brazil; Department of Pathology, AC Camargo Cancer Center, Rua Professor Antônio Prudente, 211, Liberdade, São Paulo, SP CEP 01509 - 010 Sao Paulo, Brazil

**Keywords:** Lobular carcinoma in situ, Clonal relatedness, Whole-exome sequencing, Copy number array, Breast cancer, Premalignant lesions, Molecular pathology in clinical prevention

## Abstract

**Background:**

Recent evidence suggests that lobular carcinoma in situ (LCIS) can be a clonal precursor of invasive breast cancers of both the ductal and lobular phenotypes. We sought to confirm these findings with an extensive study of fresh frozen breast specimens from women undergoing mastectomy.

**Methods:**

Patients with a history of LCIS presenting for therapeutic mastectomy were identified prospectively. Frozen tissue blocks were collected, screened for lesions of interest, and subjected to microdissection and DNA extraction. Copy number profiling, whole-exome sequencing, or both were performed. Clonal relatedness was assessed using specialized statistical techniques developed for this purpose.

**Results:**

After exclusions for genotyping failure, a total of 84 lesions from 30 patients were evaluated successfully. Strong evidence of clonal relatedness was observed between an LCIS lesion and the invasive cancer for the preponderance of cases with lobular carcinoma. Anatomically distinct in situ lesions of both ductal and lobular histology were also shown to be frequently clonally related.

**Conclusions:**

These data derived from women with LCIS with or without invasive cancer confirm that LCIS is commonly the clonal precursor of invasive lobular carcinoma and that distinct foci of LCIS frequently share a clonal origin, as do foci of LCIS and ductal carcinoma in situ.

**Electronic supplementary material:**

The online version of this article (doi:10.1186/s13058-016-0727-z) contains supplementary material, which is available to authorized users.

## Background

Since the first description of lobular carcinoma in situ (LCIS) by Foote and Stewart in 1941, the biological significance of LCIS has remained controversial [1]. Initially regarded as a direct precursor to invasive lobular carcinoma (ILC), LCIS was treated by mastectomy. Subsequent reports from the 1970s and 1980s demonstrated that the rate of breast cancer development following a diagnosis of LCIS was lower than expected for a direct precursor lesion (approximately 1 % per year) and was conferred equally to both breasts [2, 3]. This generated controversy regarding the significance of these lesions and led to disparate recommendations for optimal management, ranging from observation only to bilateral mastectomy [4–6].

In current practice, a diagnosis of LCIS is typically perceived as a risk factor rather than a precursor of subsequent carcinoma, and, as such, radical treatment has fallen out of favor [7]. Yet, molecular data demonstrating that LCIS is a clonal neoplastic proliferation that commonly harbors the same genetic aberrations as those found in adjacent invasive cancers have reinstated the notion that LCIS is both a nonobligate precursor and a risk factor for invasive breast cancer [8–14]. Evidence suggesting that LCIS is a precursor lesion includes comparative genomic hybridization studies demonstrating losses on chromosomes 16q and 17p in both LCIS and ILC [11–13]; truncating mutations in the E-cadherin gene and loss of heterozygosity of the wild-type E-cadherin allele in LCIS and adjacent ILCs [8–10]; and recent studies, including one by our group, demonstrating a clonal relationship in a small number of coexisting LCIS and ILC on the basis of similarities in genome-wide copy number profiles [14].

In the present study, we sought to provide more definitive evidence of the frequency of clonality between LCIS and synchronous malignancies. Extensive sampling of mastectomy specimens allowed us to explore the relationship between distinct foci of LCIS in cases with multifocal lesions, a unique clinical feature of LCIS reported in more than 50 % of cases [15–17]. Copy number profiling was attempted in all specimens with sufficient DNA. Further, the advent of next-generation sequencing made it possible to investigate the clonal relationship of concurrent breast lesions using mutational profiling in many of the cases.

## Methods

### Patients and samples

Patients with a documented history of LCIS presenting for therapeutic mastectomy between 2005 and 2014 who provided informed consent were enrolled preoperatively in Memorial Sloan Kettering Cancer Center institutional review board-approved protocols (IRB 01-135, 99-030). The cases reported in our earlier study [14] were ascertained from 2005 to 2008. The cases reported here were ascertained in the later period. There is no overlap in the cases presented. Following standard clinical sampling, random sampling of mastectomy specimens from all breast quadrants was performed, generating up to ten fresh frozen blocks per quadrant. Blocks were stored at −80 °C, and standard hematoxylin and eosin-stained sections were reviewed by the study pathologist (DG) to identify lesions of interest (LCIS, with or without ductal carcinoma in situ [DCIS], invasive ductal carcinoma [IDC], or ILC) according to 2012 World Health Organization histologic criteria [18]. Samples were anonymized before tissue processing. Matched germline DNA was available from blood or normal tissue for all cases. Sample pairs were selected on the basis of availability of an LCIS lesion and at least one additional LCIS, ILC, DCIS, or IDC lesion. Our report is restricted to pairs of lesions located in the same breast.

### Microdissection and DNA extraction

Selected frozen samples were processed as previously described [14]. Briefly, 10-μm-thick sections from representative frozen samples were stained with hematoxylin and the in situ and/or invasive lesions were microdissected separately with a needle under a stereomicroscope to ensure tumor cell enrichment [19]. The number of microdissected sections per case varied by lesion size and cellularity, with an average of 35 sections per lesion (range 6–89). DNA was extracted from tissue samples using the QIAamp DNA Micro Kit (QIAGEN, Valencia, CA, USA) and from available blood samples using the QIAamp DNA Blood Maxi Kit (QIAGEN). All samples were subjected to quantity and quality control and then submitted for array comparative genomic hybridization profiling and/or whole-exome sequencing. In cases with sufficient DNA, we performed both assays. In cases with insufficient DNA for copy number profiling, only whole-exome sequencing was performed.

### Copy number profiling

Digested DNA was labeled by random priming using BioPrime reagents (Life Technologies, Carlsbad, CA, USA), cyanine 5 (Cy5)-2′-deoxyuridine 5′-triphosphate (dUTP) for tumor DNA, and Cy3-dUTP for normal DNA, and then hybridized to Agilent Human 4 × 180 KM comparative genomic hybridization arrays (Agilent Technologies, Santa Clara, CA, USA) using matched normal genomic DNA as a reference. Hybridized slides were scanned and images quantified using Feature Extraction 8.5 software (Agilent Technologies). Fluorescence ratios of the scanned images were calculated, and the raw array profiles were normalized versus total intensity and guanine-cytosine content of the region hybridized to the probe, using a LOESS-based method. Log ratios were segmented using the Bioconductor [20] DNAcopy package [21]. The Bioconductor NoWaves package [22] was used to remove wavy patterns in the arrays. Log ratios of every set of 12 adjacent probes were averaged to reduce the noise level. Segments were called gains or losses if their average exceeded 1 median absolute deviation of residuals above or below the median genome-wide log ratio.

### Whole-exome sequencing

When available, DNA from microdissected lesions and matched normal DNA were subjected to whole-exome capture with SureSelect Human All Exon v4 (Agilent Technologies) using a validated protocol [23, 24]. An average of 187 million 75-bp paired-end reads were generated on an Illumina HiSeq 2000 instrument (Illumina, San Diego, CA, USA) for each sample, equivalent to a median depth of 192 (range 94–369). Reads were aligned to the reference human genome GRCh37 using the Burrows-Wheeler Aligner (BWA v0.6.2) [25]. Local realignment and quality score recalibration were performed using the Genome Analysis Toolkit (GATK v3.1.1) [26]. Deduplication was performed using Picard (v1.92) [27]. Somatic single nucleotide variants were identified using MuTect (v.1.1.4) [28], and small somatic indels were defined using VarScan2 (v2.3.6) [29] and Strelka (v3.1.1) [26]. Variants found with >5 % global minor allele frequency in dbSNP (build 137) were disregarded.

### Statistical analysis

Correlations in the pattern of allelic gains and losses between lesions were evaluated using a method previously developed by our team [30, 31]. Regions of allelic gain and loss were estimated using a one-step version of the circular binary segmentation algorithm in which at most one region of gain or loss was identified on each chromosome arm [21]. The method involves examining individual concordant gains or losses carefully to assess the evidence that each concordant change could have originated from a clonal (i.e., identical) somatic event. The closeness of identified paired copy number changes provides the primary evidence for or against a clonal origin of the lesion pairs. The results are aggregated, and a measure characterizing the strength of evidence favoring clonality is calculated. This measure is then benchmarked against the distribution of the measure in pairs of lesions from different patients to obtain a *p* value. Comparisons are reported for pairs of lesions where both members of the pair were considered to have sufficient array quality. Arrays with no clear evidence of any allelic gains and losses or a high noise level relative to few called changes are excluded from the Results section but included in Additional file 1. We used two metrics: (1) percent gained or lost and (2) 75th  percentile of |height| of gains or losses (at least ten markers long) divided by the median absolute deviation of the residuals. The quality was considered to be sufficiently good if either the percent gained or lost was >10 % or if the |height| percentile was >1.75 median absolute deviation.

Statistical comparison of exome sequencing data involved the use of a likelihood ratio test specially adapted for clonality testing of this nature [32]. This test evaluates the possibility that observed mutation matches could occur by chance in independent tumors based on the marginal probabilities of individual point mutations, estimated using mutation frequencies observed in The Cancer Genome Atlas (TCGA) [33]. This results in a *p* value testing the hypothesis that the tumors are independent (i.e., a test for clonal relatedness), with smaller values indicating stronger evidence for clonality. Absence of matches suggests that the tumors are independent.

We also used the exome sequencing results to impute copy number profiles as a validation of the comparisons obtained from the comparative genomic hybridization arrays. We accomplished this by first selecting a set of loci in the target regions of exome sequencing such that consecutive loci were separated by 250 bases and the coverage depth was at least 25 for all of the normal samples. The copy number log ratio data represent the logarithm of the tumor to normal coverage depth at these loci. We used LOESS normalization to correct for G+C artifacts by regressing the log ratio on the G+C percentage of a 1-kilobase window centered at the locus. This gave us the log ratio for approximately 227,000 loci spanning the genome. Adjacent probes were averaged as before to reduce this to 14,603 markers, which we then analyzed using the same statistical methods as described above for the data obtained using comparative genomic hybridization. These copy number plots are displayed in Additional file 2.

We elected to “call” cases as clonal using a “believe the positive” rule, but with a strict significance level; that is, a case was designated as clonal if either the mutation profiling or the copy number comparison rejected the hypothesis that the tumors were independent with *p* < 0.01. If the smaller *p* value was in the 0.01–0.05 range, we called the case “equivocal.”

## Results

Using copy number arrays, we profiled a total of 125 tumors from 50 cases. Whole-exome sequencing was also performed for tumors for which sufficient DNA was available, including one additional case with five tumors not subjected to copy number profiling. The results of all comparisons meeting quality control criteria described above in the Methods section using either copy number profiling or exome sequencing or both are presented in Table 1. Details of all individual mutations identified are provided in Additional file 3.

**Table 1:** Results of all comparisons meeting quality control criteria

Case	Age (years)	Lesion pairs	Same quadrant?	Size of invasive lesion (cm)	Number of mutations (shared/total)^a^		Clonality test *p* values		Diagnosis	Shared mutations^b^	
					Tumor 1	Tumor 2	Mutations^a^	Copy number arrays^c^		*CDH1*	*PIK3CA* ^d^
LCIS-ILC											
13	50	LCIS1-ILC	No	1.6	16/3300	16/56	<0.001	0.002	Clonal	–	–
24	57	LCIS1-ILC	Yes	2.1	25/36	25/34	<0.001	<0.001	Clonal	√	√
		LCIS2-ILC	No		2/29	2/34	<0.001		Clonal	–	–
28	60	LCIS-ILC	Yes	0.12				0.93	Independent		
31	69	LCIS-ILC	Yes	1.5				0.31	Independent		
33	65	LCIS-ILC	Yes	2.1	66/81	66/1015	<0.001	<0.001	Clonal	–	–
35	72	LCIS-ILC	Yes	4.7				<0.001	Clonal		
38	73	LCIS2-ILC	Yes	Missing	23/527	23/615	<0.001	0.54	Clonal	–	–
42	52	LCIS-ILC	Yes	2.3	56/119	56/109	<0.001		Clonal	√	√
		LCIS2-ILC	Yes					0.01	Equivocal		
43	41	LCIS-ILC	Yes	1.9				0.001	Clonal		
45	55	LCIS-ILC	Yes	12.2				<0.001	Clonal		
46	51	LCIS-ILC	Yes	4.5	0/46	0/15	1.0		Independent		
		LCIS3-ILC	No		0/37	0/15	1.0		Independent		
47	51	LCIS1-ILC	No	1.5	7/29	7/25	<0.001	<0.001	Clonal	√	–
		LCIS2-ILC	No		7/22	7/25	<0.001	0.42	Clonal	√	–
48	37	LCIS1-ILC	Yes	6.0	20/33	20/40	<0.001	<0.001	Clonal	√	–
		LCIS2-ILC	No		1/22	1/40	0.03	0.10	Equivocal	–	–
52	50	LCIS-ILC	Yes	1.9				0.005	Clonal		
		LCIS2-ILC	Yes					0.05	Independent		
55	72	LCIS-ILC	Yes	1.3	6/31	6/36	<0.001	0.002	Clonal	–	√
68	48	LCIS1-ILC	No	1.4	0/44	0/33	1.0	0.58	Independent	–	–
69	56	LCIS-ILC	Yes	3.0	18/56	18/31	<0.001	0.001	Clonal	√	√
73	44	LCIS1-ILC	Yes	0.5	0/26	0/42	1.0		Independent		
75	58	LCIS1-ILC	Yes	3.5	15/46	15/39	<0.001		Clonal	√	√
LCIS-IDC											
26	54	LCIS-IDC	No	1.8	0/29	0/32	1.0		Independent		
47	51	LCIS1-IDC	No	1.0	1/29	1/30	0.02	0.35	Equivocal	–	–
		LCIS2-IDC	No		1/22	1/30	0.03	0.48	Equivocal	–	–
53	41	LCIS1-IDC	Yes	3.7	2/20	2/23	<0.001	0.40	Clonal	–	–
		LCIS2-IDC	Yes		1/15	1/23	0.02	0.62	Equivocal	–	–
74	61	LCIS1-IDC	Yes	0.75	0/32	0/34	1.0	0.50	Independent	–	–
		LCIS2-IDC	No		0/37	0/34	1.0	0.73	Independent	–	–
		LCIS3-IDC	No		3/42	3/34	<0.001		Clonal	–	–
75	58	LCIS2-IDC	Yes	1.8	0/22	0/29	1.0		Independent	–	–
LCIS-LCIS											
4	44	LCIS1-LCIS2	No	N/A	11/46	11/30	<0.001		Clonal	–	–
5	67	LCIS1-LCIS2	No	N/A				0.85	Independent		
8	47	LCIS1-LCIS2	No	N/A	9/34	9/25	<0.001		Clonal	–	–
17	48	LCIS1-LCIS2	No	N/A	13/26	13/27	<0.001		Clonal	√	–
19	43	LCIS1-LCIS2	No	N/A	12/24	12/185	<0.001		Clonal	–	–
24	57	LCIS1-LCIS2	No	N/A	0/36	0/28	1.0		Independent	–	–
38	73	LCIS1-LCIS2	Yes	N/A	23/174	23/527	<0.001		Clonal	–	–
46	51	LCIS-LCIS3	No	N/A	5/46	5/37	<0.001		Clonal	–	–
47	51	LCIS1-LCIS2	Yes	N/A	8/29	8/22	<0.001	0.59	Clonal	√	–
48	37	LCIS1-LCIS2	No	N/A	0/33	0/22	1.0	0.59	Independent	–	–
52	50	LCIS-LCIS2	Yes	N/A	13/28	13/34	<0.001	0.02	Clonal	√	√
53	41	LCIS1-LCIS2	Yes	N/A	6/21	6/17	<0.001	0.005	Clonal	√	–
59	47	LCIS-LCIS2	No	N/A	12/31	12/26	<0.001	0.10	Clonal	√	–
73	44	LCIS3-LCIS4	No	N/A	0/14	0/11	1.0		Independent		
74	61	LCIS1-LCIS2	No	N/A	0/32	0/37	1.0	0.02	Independent	–	–
		LCIS1-LCIS3	No	N/A	1/34	1/43	0.03		Equivocal	–	–
		LCIS2-LCIS3	No	N/A	24/37	24/42	<0.001		Clonal	√	–
		LCIS4-LCIS5	No	N/A	14/41	14/47	<0.001		Clonal	–	–
LCIS-DCIS											
04	44	LCIS1-DCIS1	Yes	N/A	15/46	15/703	<0.001	<0.001	Clonal	–	–
		LCIS2-DCIS1	No	N/A	10/30	10/703	<0.001		Clonal	–	–
		LCIS1-DCIS2	No	N/A	13/46	13/24	<0.001	<0.001	Clonal	–	–
		LCIS2-DCIS2	Yes	N/A	8/30	8/24	<0.001		Clonal	–	–
06	57	LCIS-DCIS	Yes	N/A	6/50	6/75	<0.001	0.81	Clonal	–	–
16	62	LCIS-DCIS	No	N/A	0/92	0/57	1.0		Independent	–	–
26	54	LCIS-DCIS	No	N/A	0/29	0/34	1.0	0.14	Independent	–	–
47	51	LCIS1-DCIS	Yes	N/A	3/29	3/23	<0.001	0.71	Clonal	–	–
		LCIS2-DCIS	Yes	N/A	0/22	0/23	1.0	0.03	Independent	–	–
59	47	LCIS-DCIS	Yes	N/A	6/31	6/31	<0.001	0.74	Clonal	–	–
		LCIS2-DCIS	No	N/A	2/26	2/31	0.003	0.67	Clonal	–	√
68	48	LCIS1-DCIS	Yes	N/A	8/44	8/33	<0.001	0.03	Clonal	–	–
75	58	LCIS2-DCIS	Yes	N/A	0/22	0/38	1.0		Independent	–	–

^a^A blank entry indicates that whole-exome sequencing was not performed

^b^In cases with shared mutations “√” represents the presence of a shared mutation in the designated gene, while a dash represents its absence

^c^Copy number comparisons using Agilent comparative genomic hybridization. A blank entry indicates that one or more of the copy number profiles did not meet our quality control criteria (except for case 75, for which copy number profiling was not performed)

^d^Note that only seven cases are listed with a *PIK3CA* match, versus eight in Table 2. The additional match occurred between the two ductal lesions in case 47

### Clonal relatedness of LCIS and invasive breast cancers

**Fig. 1:**
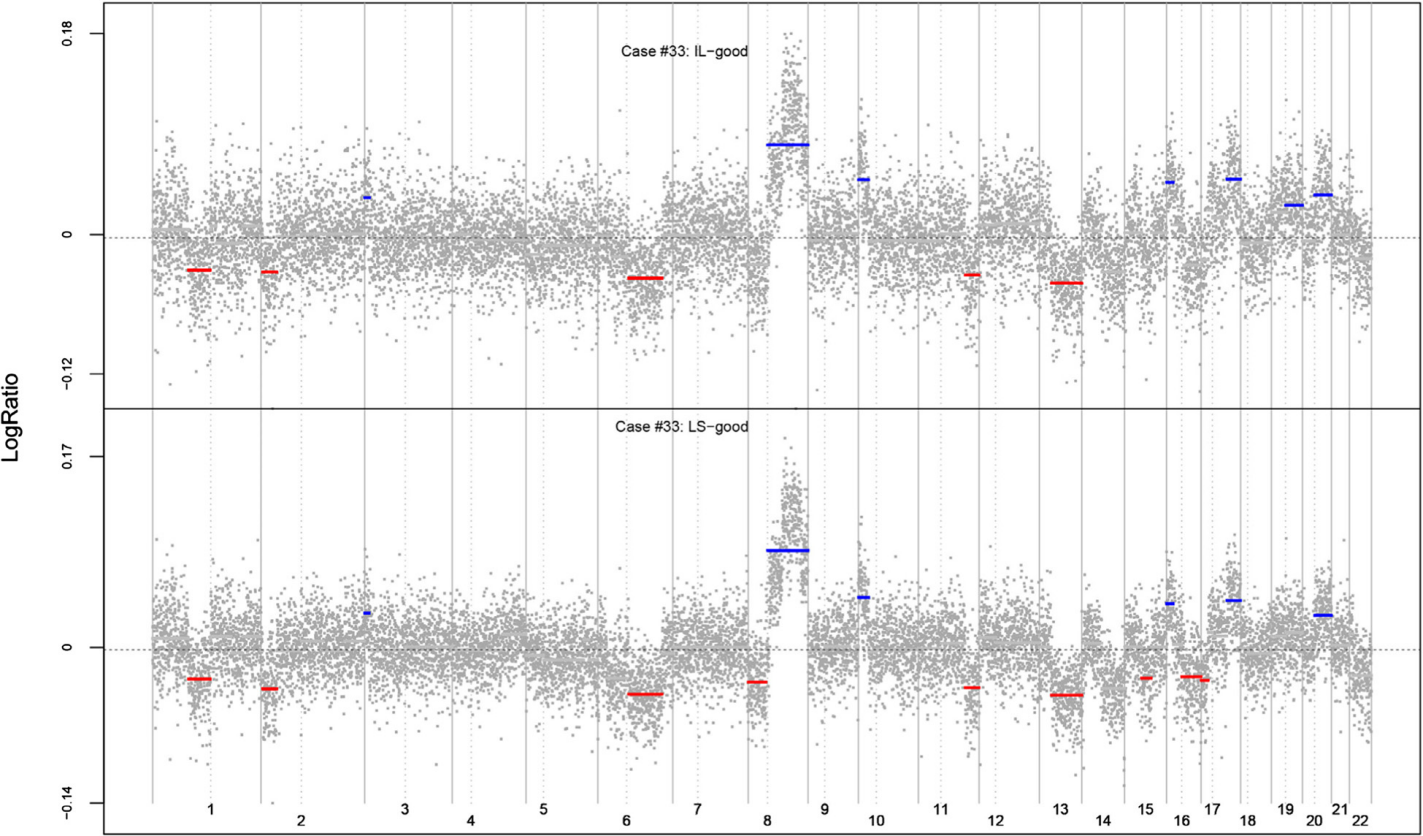
Copy number comparisons for case 33. Plots show similar copy number aberrations in both invasive lobular carcinoma (ILC) and lobular carcinoma in situ (LCIS) lesions for case 33. The *x*-axis is ordered by chromosome, and the *y*-axis is the log ratio, representing the allele count, with areas of gains and loss represented by *blue* and *red lines*, respectively. Similar gains and losses were observed in both ILC and LCIS lesions

**Fig. 2:**
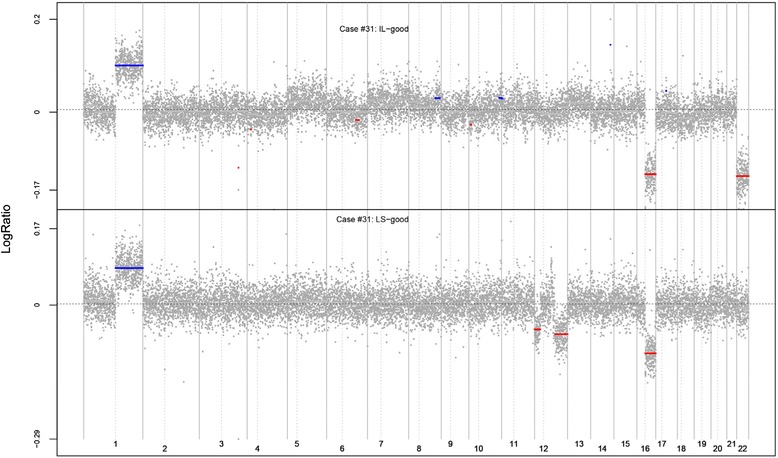
Copy number comparisons for case 31. Plots show distinct copy number changes for case 31, except for the ubiquitous 1q gains and 16q losses seen in most lobular carcinoma in situ (LCIS) lesions

The clonal relatedness of LCIS and ILC is strongly represented by both copy number and mutational analysis (Table 1). A total of 16 pairings from 14 of the 19 cases are definitively clonal on the basis of highly significant tests comparing either the copy number profiles or the mutational profiles, or both. A striking example is case 33. The copy number plots, reproduced in Fig. 1, show strongly similar patterns of gains and losses in the LCIS and ILC samples, and we identified 66 matching mutations in whole-exome sequencing (Table 1). Similar plots of all copy number comparisons, including those that did not meet quality control criteria, are provided in Additional file 1. Four pairs from three distinct cases had no matching mutations, suggesting independence, and three additional cases were judged to be independent on the basis of the copy number comparisons. The copy number plots for one of these cases (case 31) are shown in Fig. 2. There are matching whole arm gains in chromosome 1q and matching whole arm losses in chromosome 16q, but these allelic changes are emblematic of LCIS lesions generally, and the statistical method discounts the significance of these matches accordingly. The other gains and losses in the two lesions are discordant. In the remaining two comparisons, we judged the evidence not to be definitive (i.e., equivocal).

The results for pairings involving LCIS and IDC samples are shown in Table 1. In total, nine comparisons from five distinct cases were available for LCIS and one or more IDCs. Six pairings with copy number data met our quality control criteria, but these comparisons failed to provide evidence of clonality. However, shared mutational events were evident in five of nine pairwise comparisons, and for two of the five distinct cases, there was strongly significant evidence of clonal relatedness for at least one LCIS-IDC pairing. Yet, the number of matches in the various pairings ranged from one to three, substantially fewer than the number of matches typically observed in the LCIS-ILC comparisons.

### Clonal relatedness of distinct in situ lesions

Extensive sampling of mastectomy specimens allowed us to generate 18 pairs of anatomically distinct LCIS lesions from 15 patients (Table 1). The copy number plots were frequently of poor quality in one or the other of the specimens in the pair, but the whole-exome sequencing data provide strong evidence of clonal relatedness in the preponderance of cases examined (12 of 18 comparisons in 11 of 15 distinct cases), including several cases where the lesions were harvested from anatomically distinct quadrants of the breast. Similarly, in the 13 comparisons from 8 distinct cases with both LCIS and DCIS lesions, we identified strong evidence of clonal relatedness in 9 comparisons from 5 cases (Table 1), again with evidence of clonal relatedness in lesions harvested from different quadrants of the breast.

### Further observations from exome profiling

Exome sequencing permits the derivation of copy number arrays, serving as a validation of the results from the Agilent comparative genomic hybridization approach for those cases with sequencing available. The copy number segmentation plots are provided in Additional file 2, and these can be compared with the corresponding copy number plots obtained from comparative genomic hybridization, which are available in Additional file 1. The table in Additional file 4 compares the *p* values obtained from these two methods side by side. Although there is clearly considerable variation in the actual *p* values observed, the results are broadly consistent.

The results from the cases with exome sequencing also provide some novel insights into the typical mutational profiles of the distinct lesions and the roles that individual genes may play. We tabulated the frequencies at which mutations were observed in individual genes among the 20 cases with tumors sequenced, and the results are summarized in Table 2. A mutation was designated as “clonal” if it was observed in two or more tumors from the patient. A mutation was designated as “private” if it was observed in at most one tumor from the patient. The relative frequencies of clonal and private mutations are displayed in Table 2, where the genes are ranked on the basis of overall mutation frequency. This table was constructed after excluding the 5 tumors that were hypermutated with >200 mutations (see Additional file 3) as well as an outlier gene (*FSIP2*) that exhibited 13 nonfunctional mutations in 2 cases. The table shows clearly that the most common driver mutations, *CDH1* (E-cadherin) and *PIK3CA*, have high percentages of clonal mutations, but that the relative frequency rapidly drops to the overall average of <20 %. *CBFB* also exhibits a high relative frequency of clonal mutations. Clearly the preponderance of “clonal” mutations occur in a very broad spectrum of genes largely unrelated to the genes’ propensity to harbor somatic mutations. It is also noteworthy that all but one of the clonal mutations in *CDH1* and *PIK3CA* occurred in pairs involving lobular lesions (see Table 1 for the specific cases in which clonal mutations occurred in these genes). Indeed, for all 16 patients with mutations in *CDH1*, the mutations occurred in lobular lesions.

**Table 2:** Mutation frequencies^a^

Gene	Mutations per gene^b^ (20 cases)					Percent functional mutations^c^			
	Mutations	Cases^d^	Clonal	Private	Percent clonal	Clonal		Private	
*CDH1*	24	16	11	13	54 %	11/11	100 %	13/13	100 %
*PIK3CA*	13	11	9	4		9/9^e^		4/4	
*SPRR3*	10	9	1	9	30 %	1/1	55 %	1/9	70 %
*CBFB*	10	8	7	3		6/6		3/3	
*GATA3*	9	6	2	7		2/2		6/7	
*NBPF1*	8	8	2	6		2/2		4/6	
*MUC12*	7	5	1	6		0/1		4/6	
*KMT2C*	6	6	1	5		1/1		4/5	
*MUC4*	6	5	2	4		0/2		3/4	
*TCEAL3*	6	4	5	1		0/5		0/1	
*MAP3K1*	6	4	3	3		3/3		3/3	
*BCLAF1*	5	5	0	5		0/5		5/5	
*HMCN1*	5	5	0	5		0/0		3/5	
*TMPRSS13*	5	4	1	4		1/1		4/4	
*REXO1L1*	4	4	0	4	13 %	0/0	75 %	0/4	79 %
*TDG*	4	4	0	4		0/0		3/4	
*DDX11*	4	4	1	3		1/1		2/3	
*ABCC5*	4	4	0	4		0/0		3/4	
*MACF1*	4	4	0	4		0/0		4/4	
*AGGF1*	4	4	1	3		1/1		3/3	
*OR13C2*	4	2	0	4		0/0		4/4	
*NASP*	4	2	0	4		0/0		4/4	
*SERPINA3*	4	2	0	4		0/0		4/4	
28 genes	3	3	14	70		10/14		50/70	
159 genes	2	2	69	249	16 %	50/69	72 %	164/249	66 %
1477 genes	1	1	224	1253		158/224	71 %	854/1253	68 %

^a^This table includes only the 20 cases with exome sequencing results available. Data from five tumors that were hypermutated are excluded: case 4 (DCIS1), case 13 (LCIS), case 33 (ILC), case 38 (LCIS), and case 38 (ILC)

^b^If a mutation was observed in two or more tumors from the case, it was designated a “clonal” mutation. If it was detected in a single tumor from the case, it was designated “private.” Note that the sum of clonal and private mutations can exceed the number of cases, since more than one distinct mutational locus can occur in the same patient or even in the same tumor

^c^The category “functional” excludes synonymous mutations

^d^This represents the number of cases in which one or more tumors possess mutations in the designated gene

^e^We note that two of the nine clonal *PIK3CA* mutations occurred in pairs of ductal tumors

All of the 37 mutations observed in either of the two most frequently mutated genes, *CDH1* and *PIK3CA*, were functional (nonsynonymous, truncating, or nonsense as opposed to synonymous). This compares with a baseline relative frequency of functional mutations of 69 % across all genes, a frequency essentially unrelated to the overall mutation frequencies of the genes after excluding these two genes (Table 2). Interestingly, we see no evidence that clonal mutations are more likely than private mutations to be functional. The overall mutational burdens of the distinct tumor types were also similar. The median numbers of mutations per lesion were 33 for LCIS, 38 for ILC, 29 for IDC, and 33 for DCIS, numbers that are not significantly different.

## Discussion

Lobular carcinoma in situ is a noninvasive neoplastic lesion of the breast characterized by distention of the lobules and terminal ductal lobular units by a proliferation of atypical but monomorphic dyshesive cells [1, 34]. It is most frequently diagnosed in women aged 40–55 years [2] as an incidental finding in benign breast biopsies, reported in 0.5–4.0 % of cases [15, 35, 36], but the true prevalence of LCIS in the general population is likely to be higher. Although LCIS is typically perceived as a marker of increased breast cancer risk [37], emerging evidence of genotypic similarities between coexisting LCIS and invasive lobular breast cancer suggests that a subset of LCIS may carry a higher risk for progression to invasive disease, exhibiting true precursor potential [8–14]. Using a combination of copy number profiling and whole-exome sequencing in this study, we have demonstrated strong clonal relationships between multiple foci of LCIS and DCIS as well as LCIS and ILC, confirming that LCIS is commonly the clonal precursor of ILC, with distinct foci of LCIS frequently sharing a clonal origin. Furthermore, the mutational data exhibit large numbers of mutations in both the LCIS and DCIS samples, with absolute numbers similar to the mutational burdens of invasive cancers. The dominant influence of mutations in *CDH1* and *PIK3CA* reflects the recent results from TCGA showing that these two genes harbor by far the most frequently recurring mutations in lobular breast cancer [38].

The clinical characteristics of LCIS that have long supported its role as a risk factor for the subsequent development of breast cancer include a cumulative long-term risk that is generally conferred equally to both breasts, averaging 1–2 % per year, and the observation that not all breast cancers developing after a diagnosis of LCIS have lobular histology [39, 40]. However, in some studies, researchers have reported a higher rate of breast cancer in the ipsilateral breast [41, 42], and in these studies the majority of the cancers reported have lobular histology, supporting a precursor role for LCIS. This clinical observation, in parallel with Surveillance, Epidemiology, and End Results data demonstrating an increasing incidence of both LCIS and ILC from the late 1980s to the mid-1990s among women 50 years of age and older [43], has generated renewed interest in the debate over the clinical significance of LCIS.

Previous investigations provided suggestive evidence about the clonal relationships between LCIS and related breast tumors, especially ILC, using a variety of genetic techniques [8–14]. In our present study, by using whole-exome sequencing in selected cases, we provide exceptionally convincing evidence of clonal relatedness in multiple cases. Since whole-exome sequencing allowed us to detect precise mutational matches, and since the specific locations of the vast majority of somatic mutations are exceptionally rare, the occurrence of more than one match is extremely unlikely to occur by chance, unless the matches occur at one of the very few mutational hot spots. We observed many cases of tumor pairs with multiple matches, providing overwhelming evidence of clonality in these cases. Our results indicate very strong clonal relationships not only between LCIS and invasive lobular cancers but also among anatomically distinct in situ lesions, including LCIS and DCIS lesions, as has been suggested in previous studies [14, 44]. We also saw evidence of clonality between LCIS and IDC, though the numbers of matching mutations were small and the evidence for clonality was not supported by the copy number comparisons.

We do acknowledge that mutation calling artifacts can occur due to germline mutations that are not recognized as such or due to misalignment in a region that is difficult to sequence. Therefore, caution must be taken in interpreting results in pairs with very few shared mutations. We further caution that our LCIS cases should not be considered to be representative of typical LCIS encountered in women without invasive cancers. In this study, we ascertained the LCIS specimens from mastectomies conducted to remove a diagnosed invasive cancer. Consequently, it is probable that our study overrepresents the LCIS lesions that progress. Furthermore, we evaluated the mutational profiles at the time of breast cancer diagnosis. It is plausible that the mutational burdens would be lower in LCIS tumors found earlier in the disease process.

For many cases in our study, we used both copy number profiling and mutational testing to evaluate the clonal relatedness of the tumors. Typically, though not always, the results from these two methods are congruent. However, in several cases, mutational profiling demonstrated strong evidence of clonality, while copy number profiling did not. We used a “believe the positive” rule in these cases, since the presence of multiple mutational matches is extraordinarily unlikely to occur by chance and thus provides strong evidence of clonal relatedness. There were no examples where the copy number profiles were strongly clonal while the mutational data were not. This may be because the copy number approach is less sensitive in detecting somatic events due to noise in the data, even though we excluded many cases for excessive noise. It is also possible that the early events in breast tumorigenesis are more typically mutational events and that the copy number gains and losses tend to occur later. Indeed, it is possible that cases we called “independent” based solely on nonsignificant copy number comparisons without exome data could be false-negatives for this reason.

Our broader exploratory analyses of the mutational spectra observed in the subset of cases with exome sequencing produced some interesting findings. The most frequently mutated gene is the E-cadherin gene (*CDH1*), known to be silenced in most LCIS tumors, through either allelic losses of chromosome 16q or the presence of a mutation [45]. Our results showed no ductal lesions harboring a mutation in *CDH1*, confirming the diagnostic contrast of this gene in ductal versus lobular in situ lesions [46]. In the most commonly mutated genes, the frequencies of “clonal” mutations relative to “private” mutations were notably higher than the collective relative frequency for the very large number of genes that occurred infrequently. Since the clonal mutations must have occurred early in tumor development, this confirms the importance of these common mutations as driver mutations. The two genes with the highest frequencies of occurrence, *CDH1* and *PIK3CA*, were in this category*.* However, after excluding these genes, there was very little remaining correlation between the overall prevalence of gene mutations and the relative frequency of clonal mutations, suggesting that many mutations that occurred in the originating clonal cell may actually be passenger mutations. This notion is further buttressed by the fact that the relative frequencies of functional to nonfunctional mutations did not appear to be different for clonal versus private mutations. There appeared to be little overall difference in the mutational burden of in situ versus invasive cancers. All four groups of tumors had a similar mutational burden overall.

Taken together, our observational and molecular data support the contention that LCIS is not only a risk indicator but also a nonobligate precursor of invasive breast cancer. In particular, the presence of concordant gene copy number changes and identical mutations in matched LCIS and ILC from the same patients, as demonstrated here, supports the premise that the majority of LCIS lesions found in association with ILC are in fact true precursor lesions. With increasing numbers of women presenting with LCIS, a better understanding of the malignant potential and factors that may alter the risk of breast cancer development are paramount to appropriately counseling women with this lesion.

## Conclusions

LCIS has traditionally been considered to be a marker of invasive breast cancer risk rather than a precursor lesion. Our study provides definitive evidence that LCIS is frequently a precursor lesion. Furthermore, the mutational spectra of the LCIS lesions in our study were broadly indistinguishable from those of the invasive carcinomas. These results suggest that LCIS lesions have the hallmarks of invasive cancers. Our present findings provide important insights for clinicians who counsel women with LCIS regarding breast cancer prevention options.

## Abbreviations

Cy, cyanine; DCIS, ductal carcinoma in situ; dUTP, 2'-deoxyuridine 5'-triphosphate; IDC, invasive ductal carcinoma; IL, invasive lobular; ILC, invasive lobular carcinoma; LCIS, lobular carcinoma in situ; LS, lobular carcinoma in situ; N/A, not applicable; TCGA, The Cancer Genome Atlas

## Additional files

Additional file 1: Separate plots are provided for copy number comparisons of all pairs of lesions ascertained in the study, including those comparisons not presented in the main text because one or other of the tumors in the pair was considered to have insufficient quality. We used two quality metrics: percent gained or lost; 75th percentile of |height| of gains or losses (at least ten markers long) divided by the median absolute deviation of the residuals. The quality was considered to be sufficiently good if either the percent gained or lost was >10 % or if the |height| percentile was >1.75 median absolute deviation. The figures display the log ratios for each marker ordered across the genome, side by side for each tumor in the pair. The *blue lines* indicate regions of allelic gain, and the *red lines* indicate regions of allelic loss, as determined by the segmentation algorithm used [21]. (PDF 19100 kb) Additional file 2: Separate plots are provided for copy number comparisons imputed from the exome sequencing platforms for all cases where good-quality data were obtained from use of both this method and the method based on comparative genomic hybridization. These plots can be compared with the corresponding plots obtained using comparative genomic hybridization in Additional file 1. For most tumors, the copy number patterns derived from the two methods are very similar. *p* Values are compared in the table in Additional file 4. (PDF 7994 kb) Additional file 3: Details of all individual mutations detected by exome sequencing. (XLSX 756 kb) Additional file 4: This table compares the *p* values obtained from the clonality tests based on copy number profiling derived using comparative genomic hybridization and the exome arrays. Although there is clearly considerable variation in the actual *p* values observed, there is consistency in identifying strong clonality signals (*p* < 0.01), except for case 48 (LCIS2-ILC). (DOCX 28 kb)
