# Supplementary material for: Clonal relationships between lobular carcinoma in situ and other breast malignancies
Source: Breast Cancer Res. 2016 Jun 23;18:66. doi: 10.1186/s13058-016-0727-z (PMC4918003; doi:10.1186/s13058-016-0727-z)

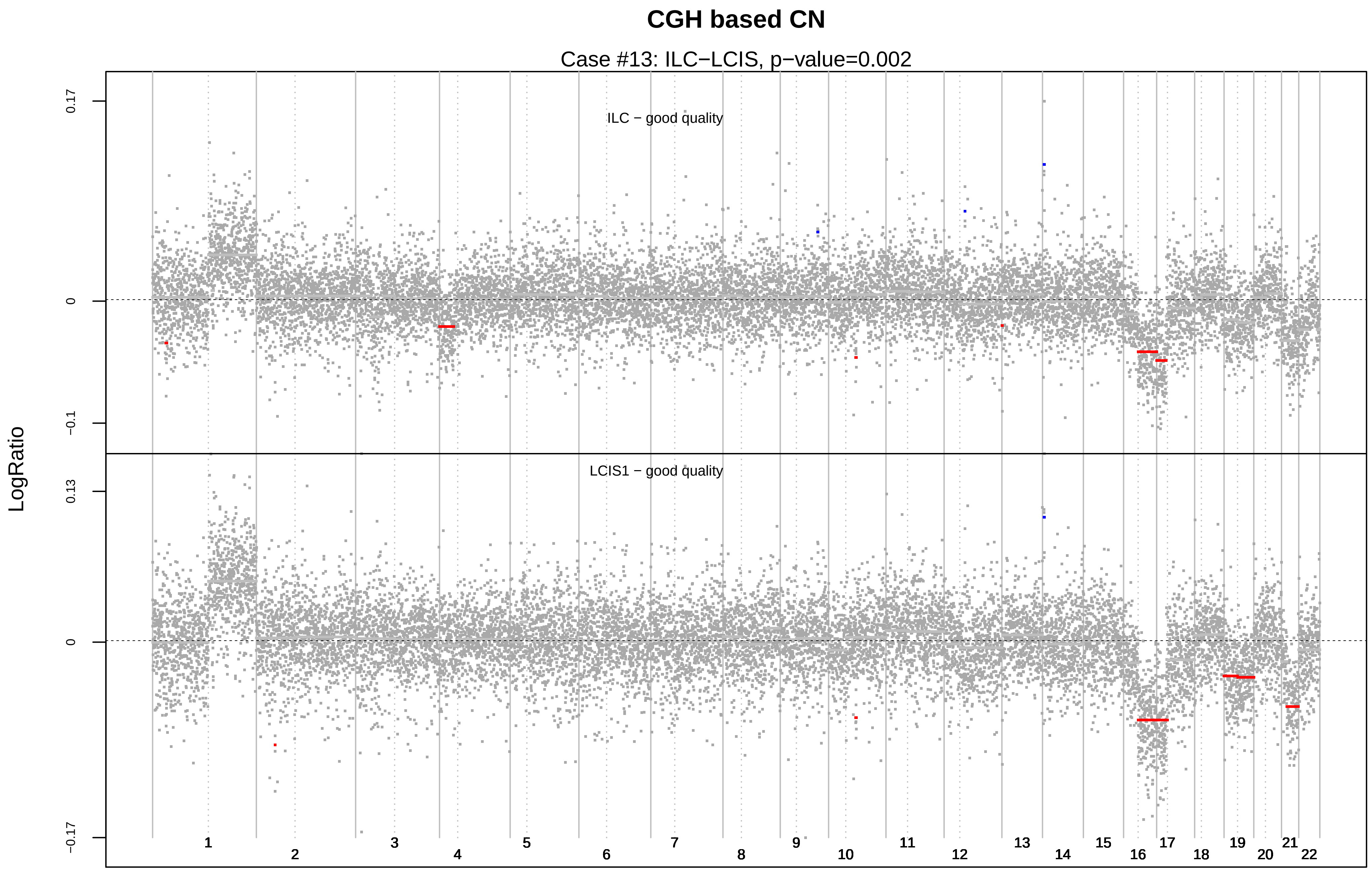

CGH based CN
Case #13: ILC−LCIS, p−value=0.002
ILC − good quality
LCIS1 − good quality
LogRatio
0.17
0
−0.1
0.13
0
−0.17
1
2
3
4
5
6
7
8
9
10
11
12
13
14
15
16
17
18
19
20
21
22

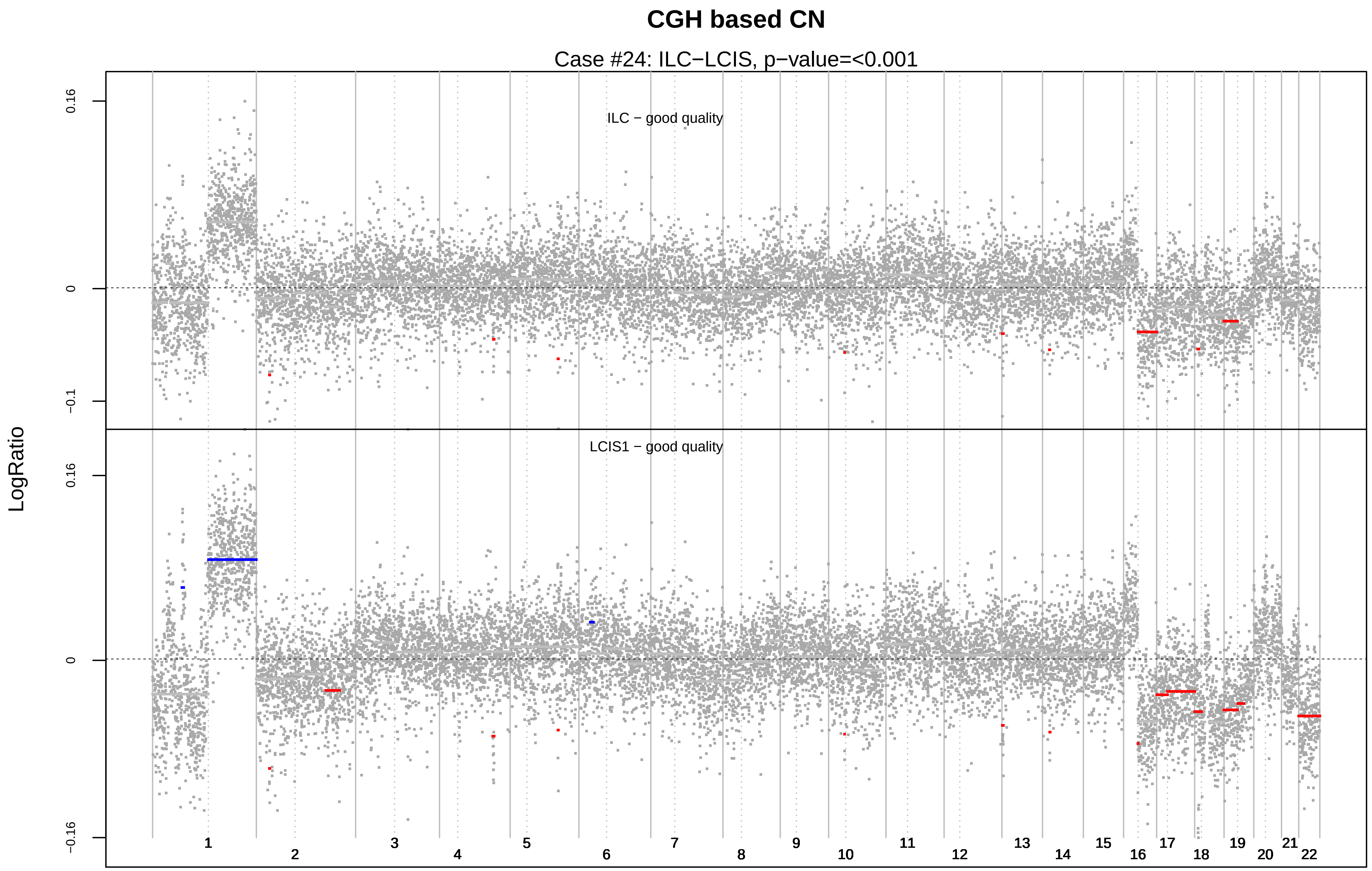
CGH based CN
Case #24: ILC−LCIS, p−value=<0.001
ILC − good quality
LCIS1 − good quality
LogRatio
0.16
0
−0.1
−0.16
1
2
3
4
5
6
7
8
9
10
11
12
13
14
15
16
17
18
19
20
21
22

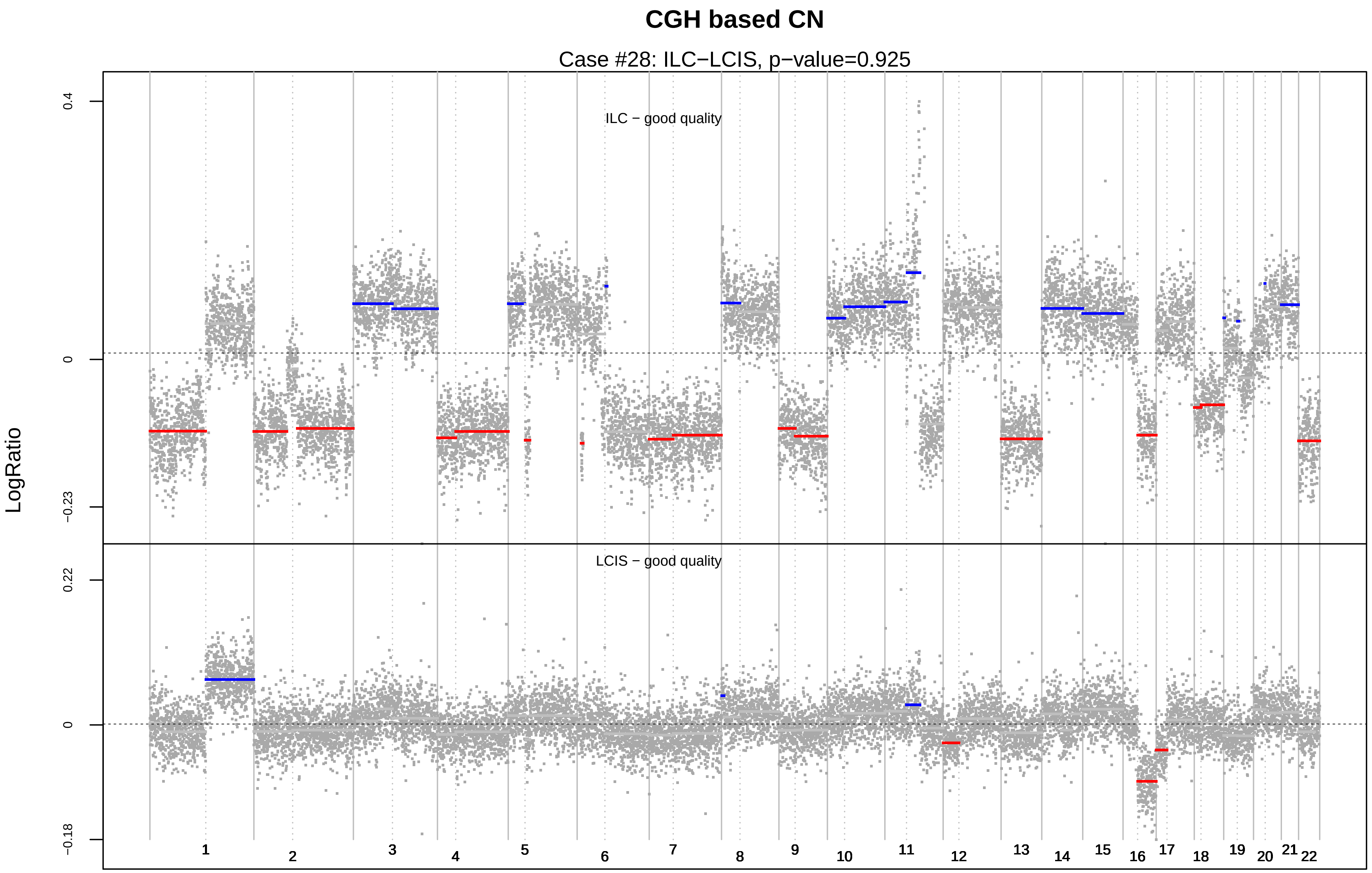
CGH based CN
Case #28: ILC−LCIS, p−value=0.925
ILC − good quality
LCIS − good quality
LogRatio
0.4
0
−0.23
0.22
0
−0.18
1
2
3
4
5
6
7
8
9
10
11
12
13
14
15
16
17
18
19
20
21
22

CGH based CN

Case #31: ILC−LCIS, p−value=0.312

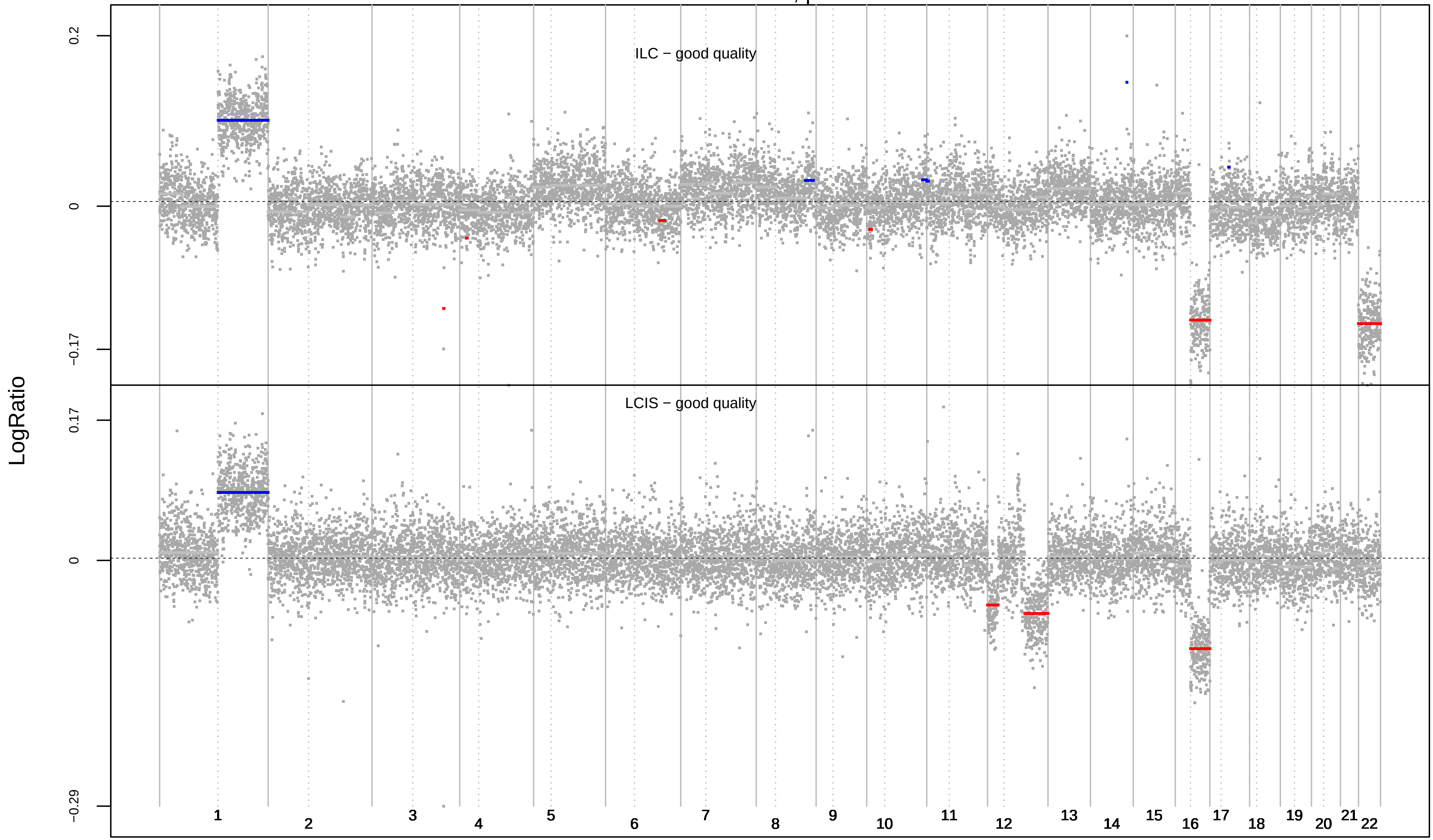

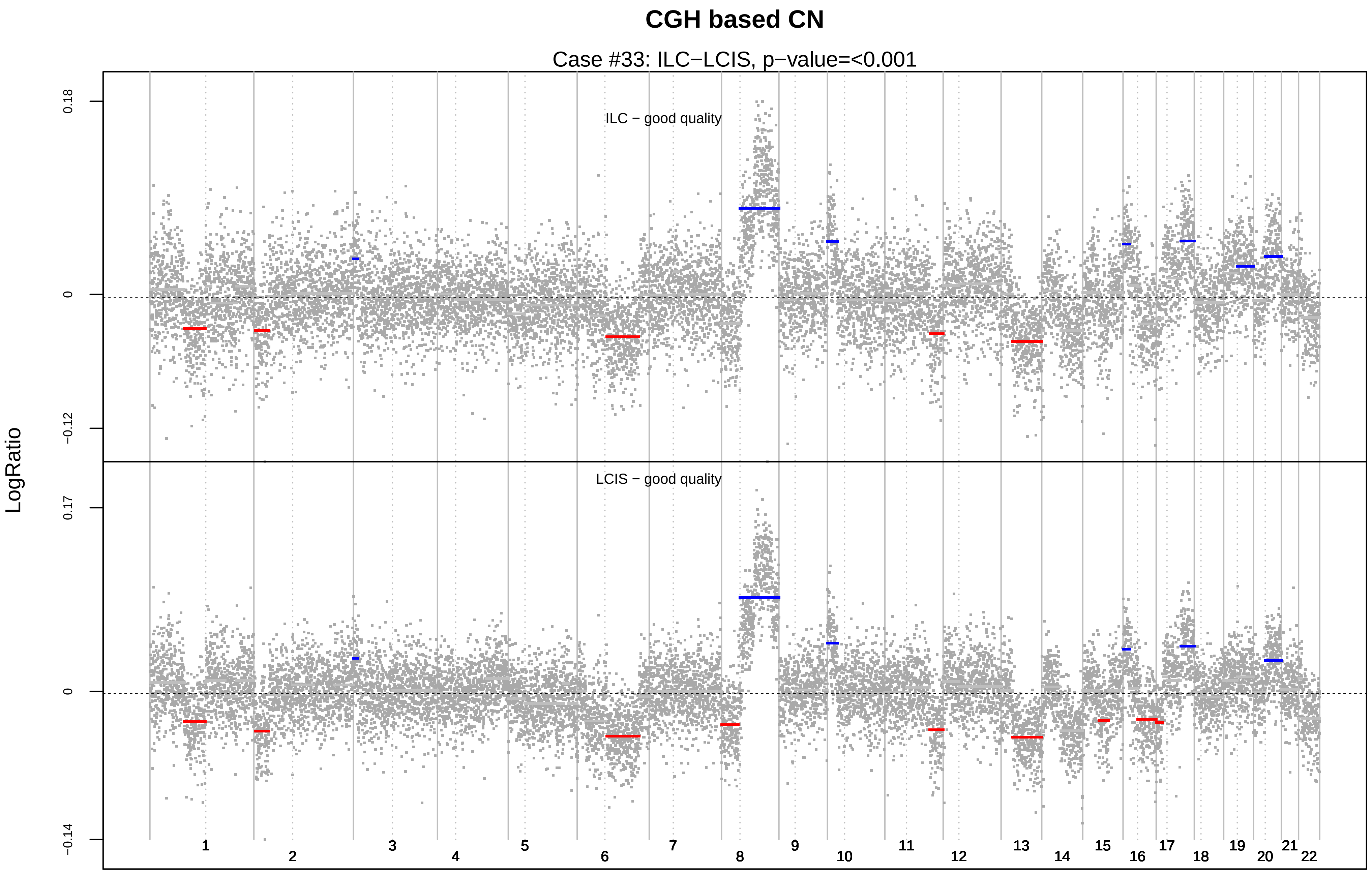
CGH based CN
Case #33: ILC−LCIS, p−value=<0.001
ILC − good quality
LCIS − good quality
LogRatio
0.18
0
−0.12
0.17
0
−0.14
1 2 3 4 5 6 7 8 9 10 11 12 13 14 15 16 17 18 19 20 21 22

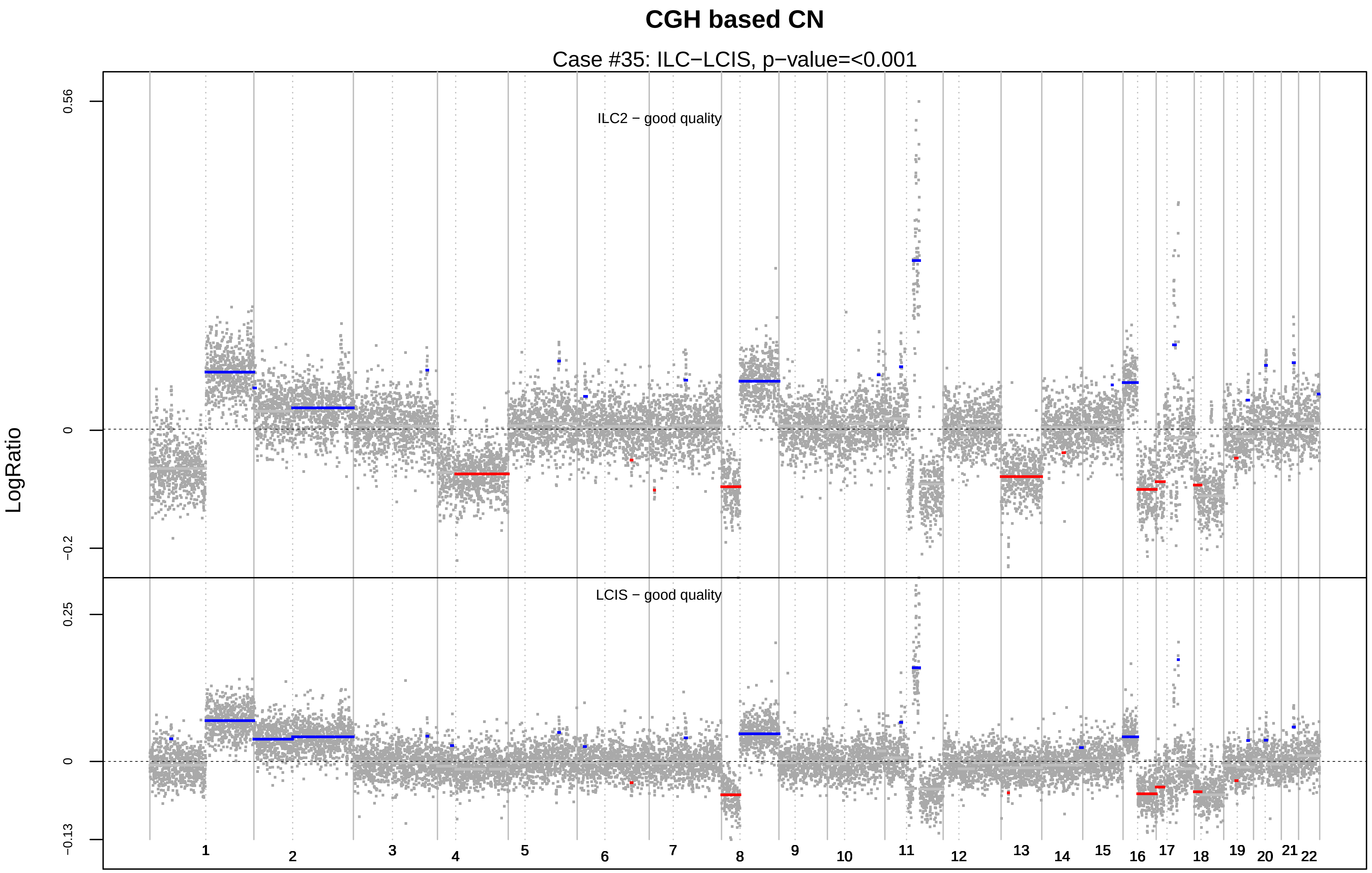

CGH based CN
Case #35: ILC−LCIS, p−value=<0.001
ILC2 − good quality
LCIS − good quality
LogRatio
0.56
0
−0.2
0.25
0
−0.13
1
2
3
4
5
6
7
8
9
10
11
12
13
14
15
16
17
18
19
20
21
22

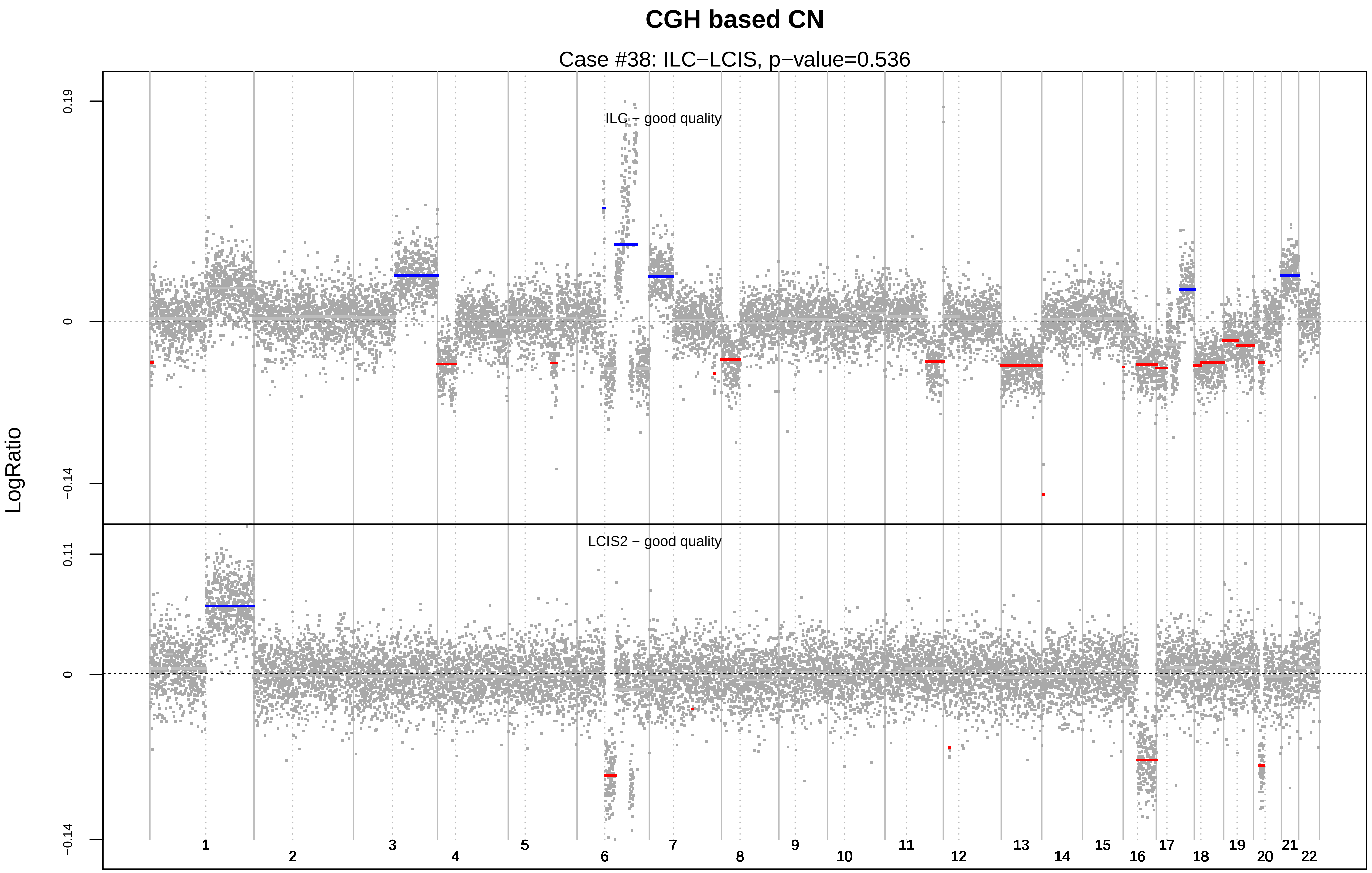

CGH based CN
Case #38: ILC−LCIS, p−value=0.536
LogRatio
0.19
0
−0.14
ILC − good quality
0.11
0
−0.14
LCIS2 − good quality
1 2 3 4 5 6 7 8 9 10 11 12 13 14 15 16 17 18 19 20 21 22

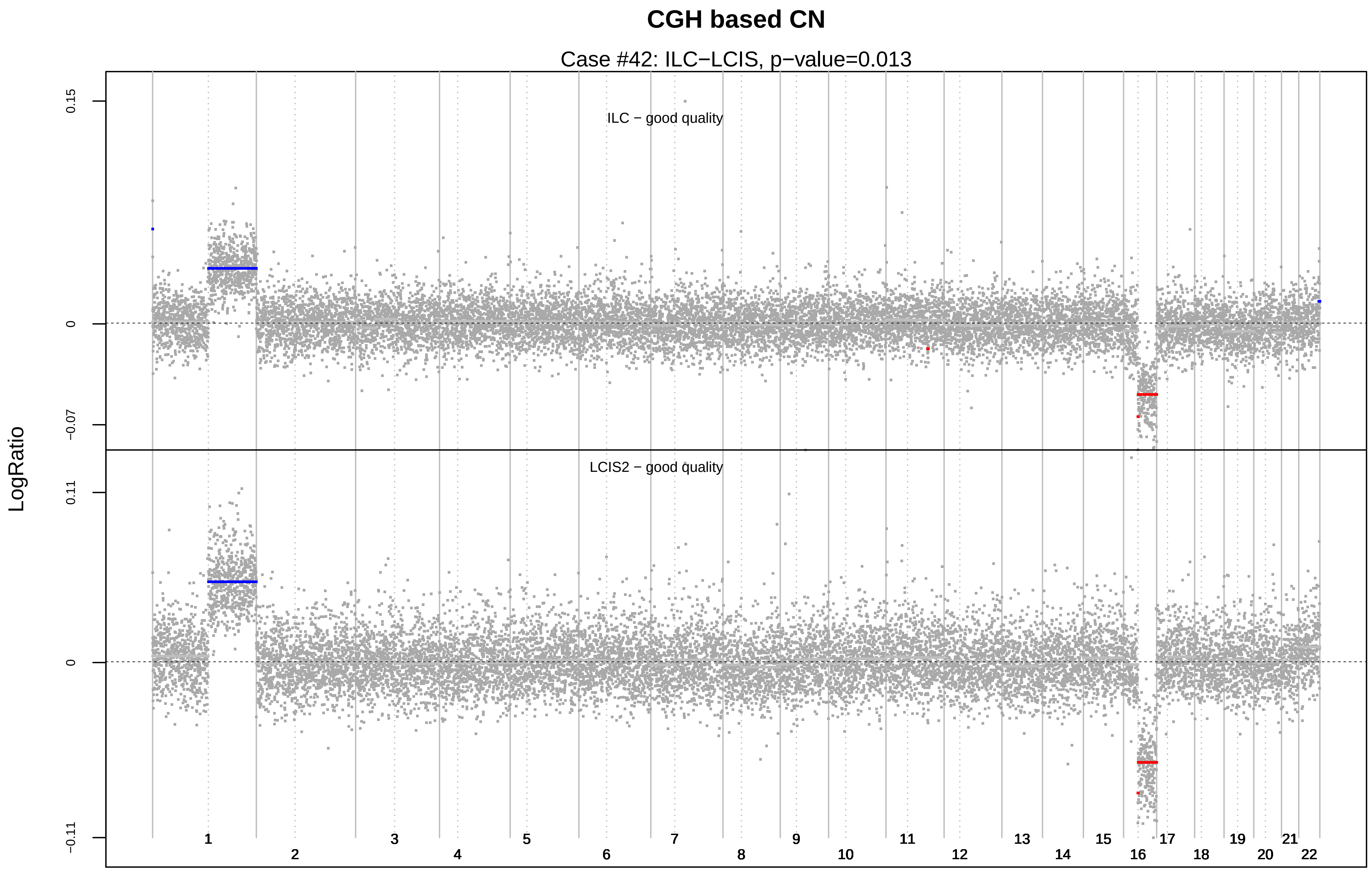
CGH based CN
Case #42: ILC−LCIS, p−value=0.013
ILC − good quality
LCIS2 − good quality
LogRatio
0.15
0
−0.07
0.11
0
−0.11
1
2
3
4
5
6
7
8
9
10
11
12
13
14
15
16
17
18
19
20
21
22

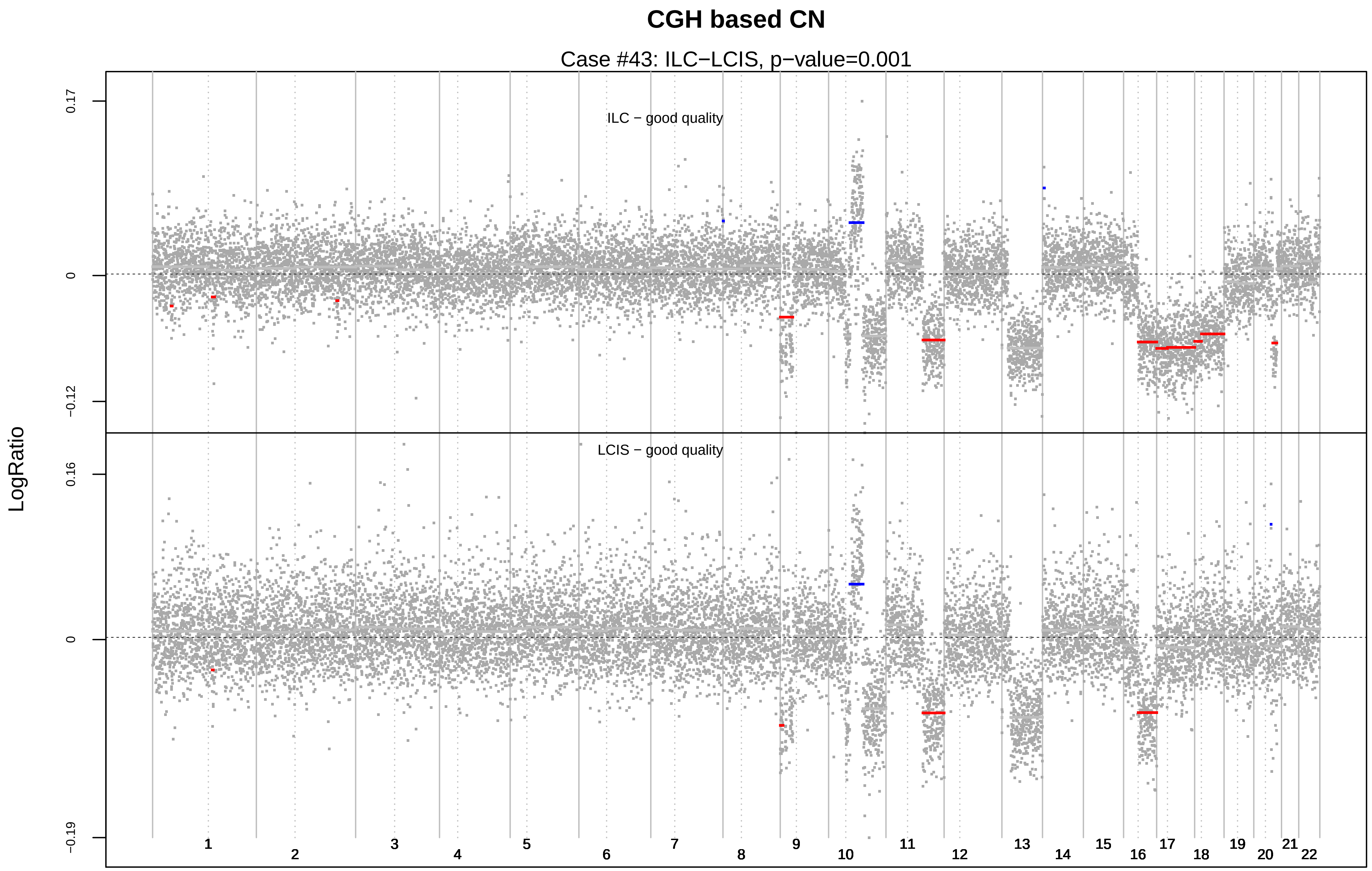

CGH based CN
Case #43: ILC−LCIS, p−value=0.001
ILC − good quality
LCIS − good quality
LogRatio
0.17
0
−0.12
0.16
0
−0.19
1
2
3
4
5
6
7
8
9
10
11
12
13
14
15
16
17
18
19
20
21
22

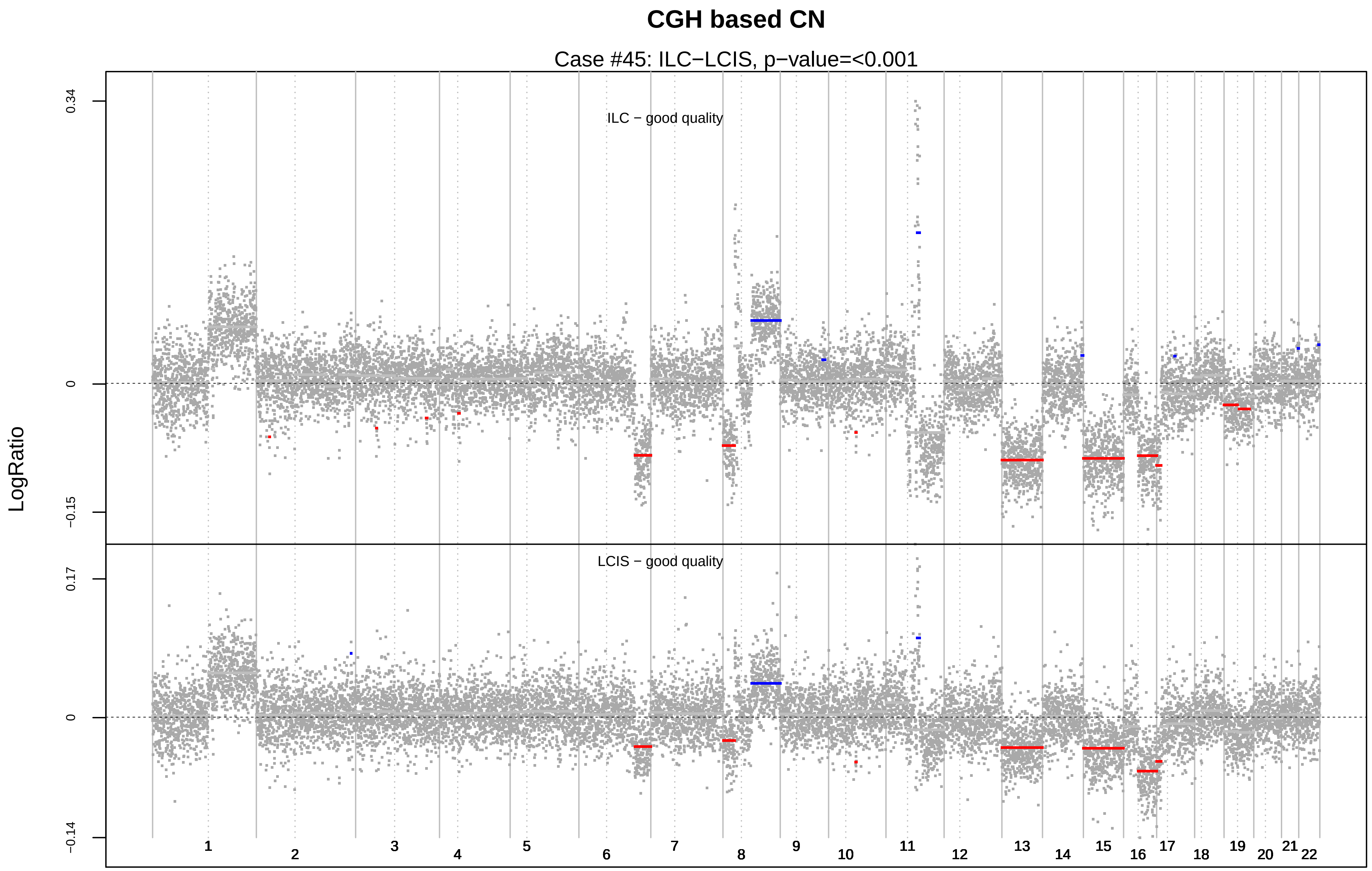

CGH based CN
Case #45: ILC−LCIS, p−value=<0.001
ILC − good quality
LCIS − good quality
LogRatio
0.34
0
−0.15
0.17
0
−0.14
1
2
3
4
5
6
7
8
9
10
11
12
13
14
15
16
17
18
19
20
21
22

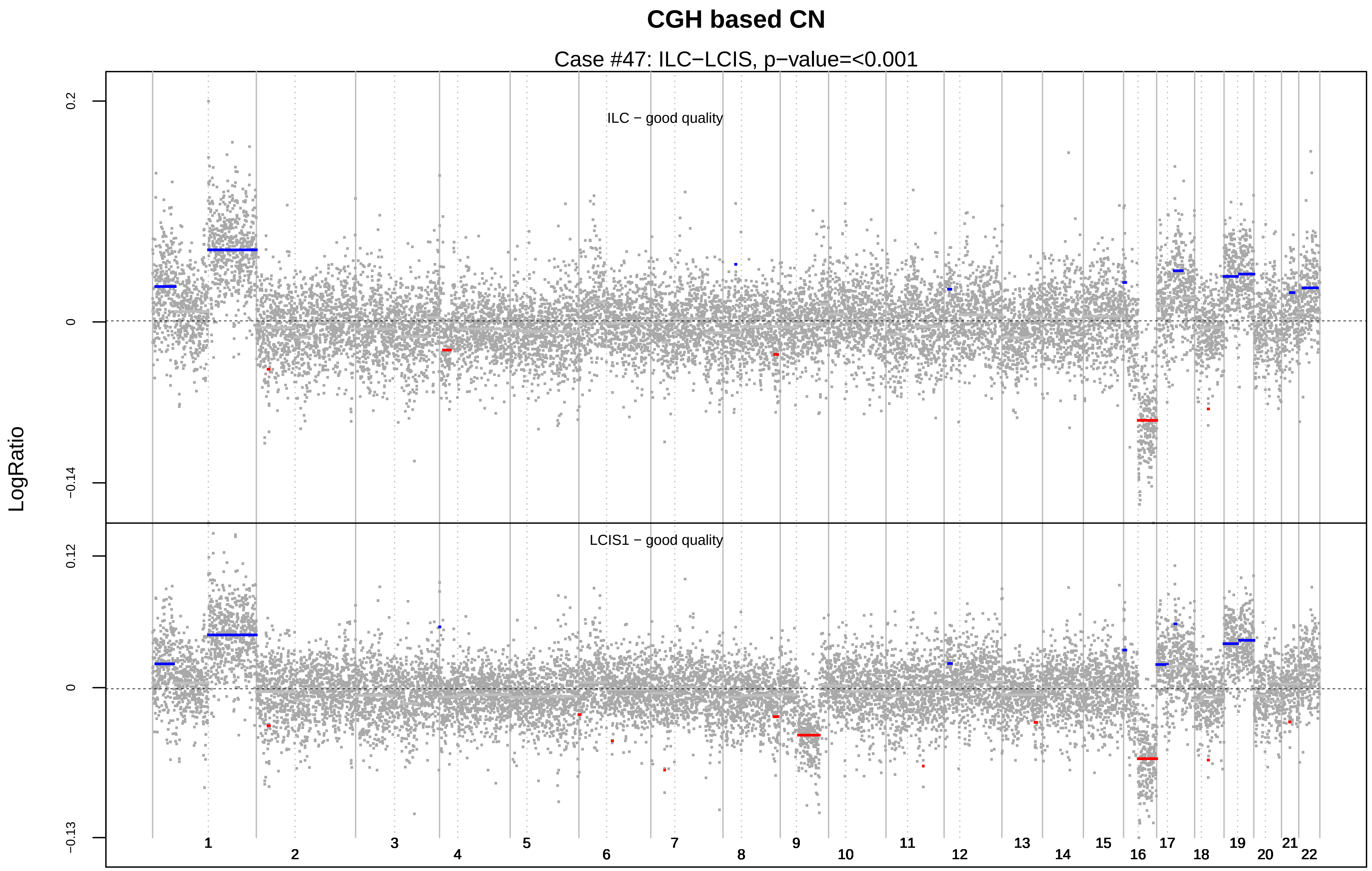
CGH based CN
Case #47: ILC−LCIS, p−value=<0.001
ILC − good quality
LCIS1 − good quality
LogRatio
0.2
0
−0.14
0.12
0
−0.13
1
2
3
4
5
6
7
8
9
10
11
12
13
14
15
16
17
18
19
20
21
22

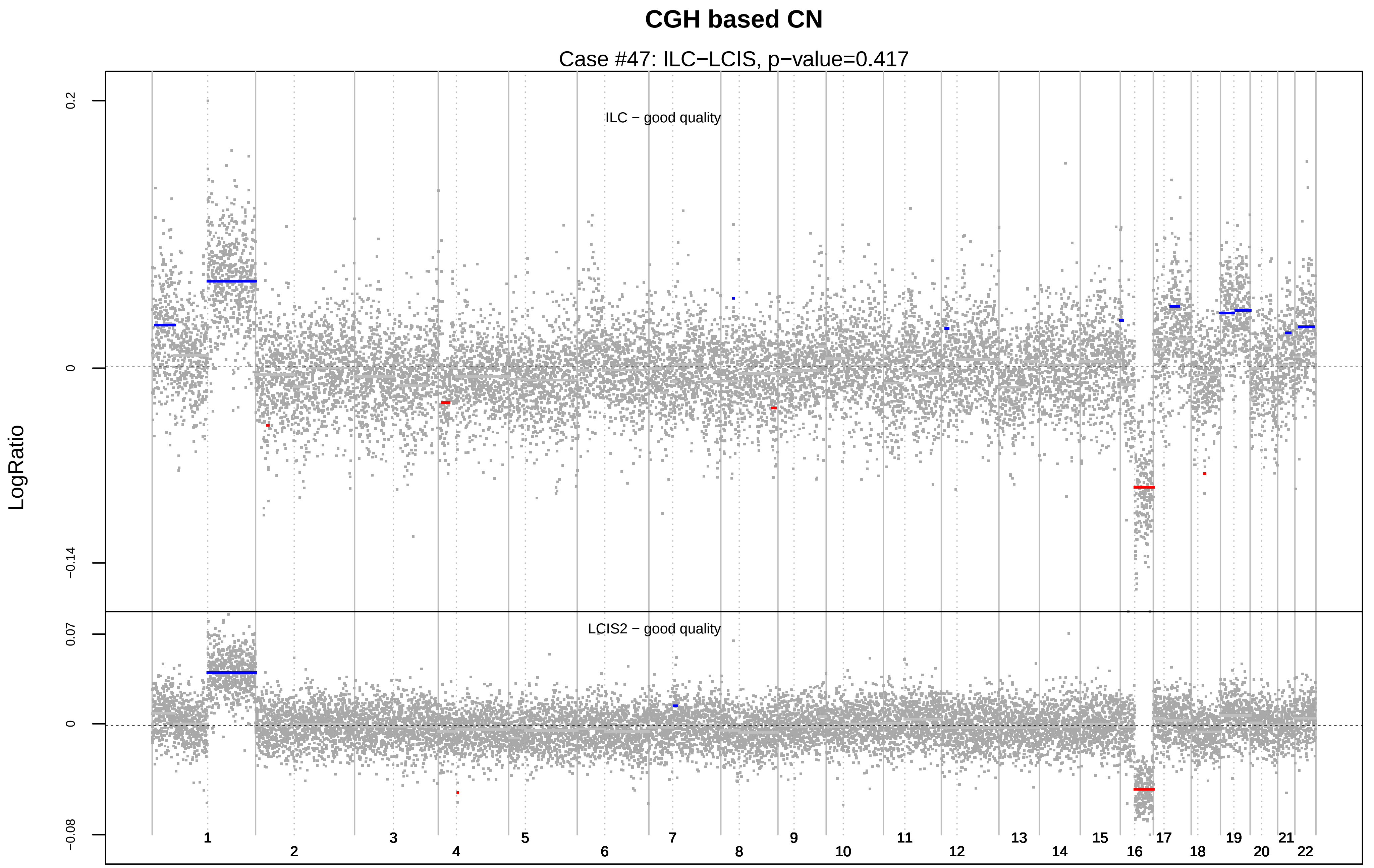
CGH based CN
Case #47: ILC−LCIS, p−value=0.417
ILC − good quality
LCIS2 − good quality
LogRatio
0.2
0
−0.14
0.07
0
−0.08
1
2
3
4
5
6
7
8
9
10
11
12
13
14
15
16
17
18
19
20
21
22

CGH based CN

Case #48: ILC−LCIS, p−value=<0.001

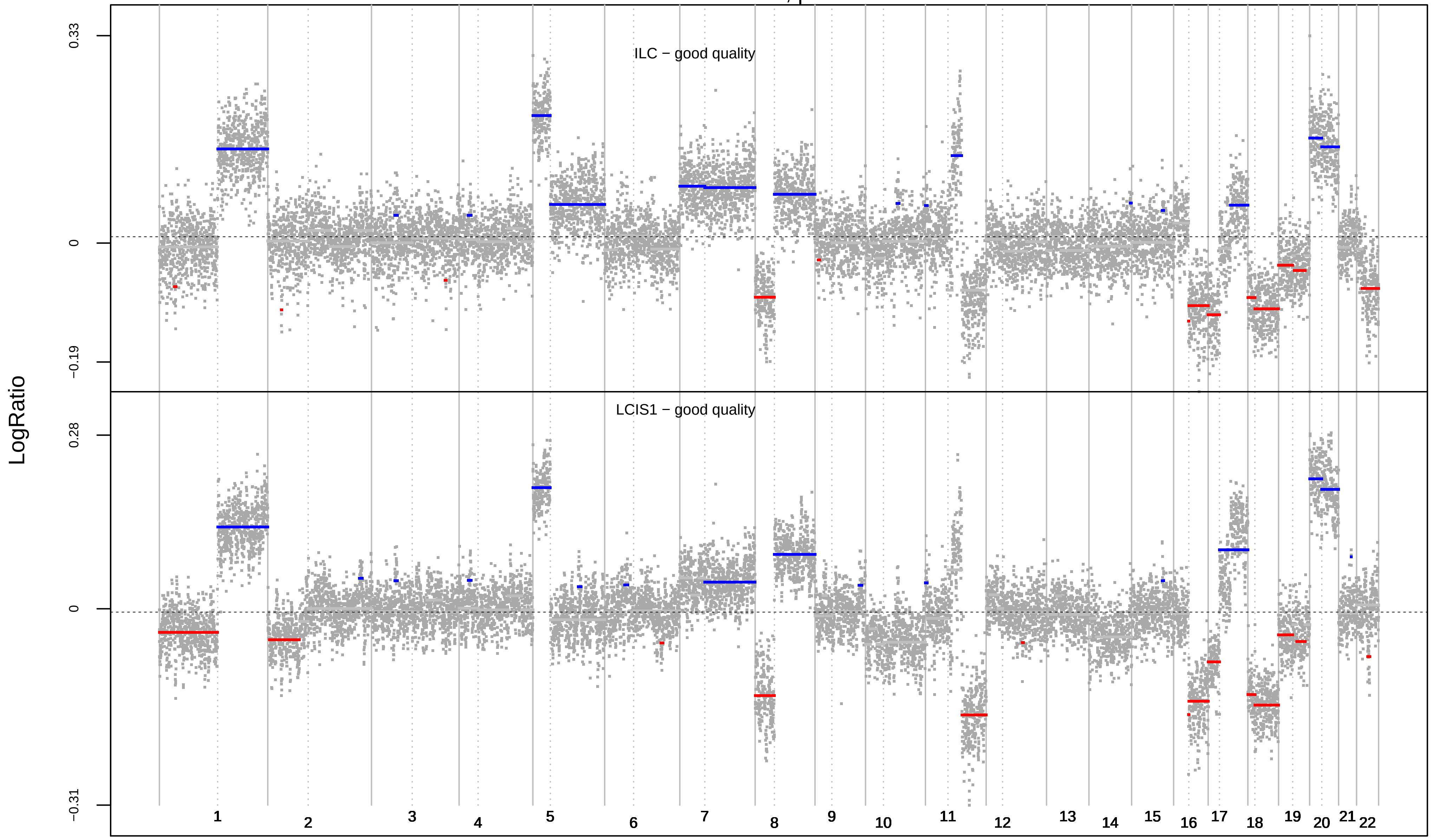

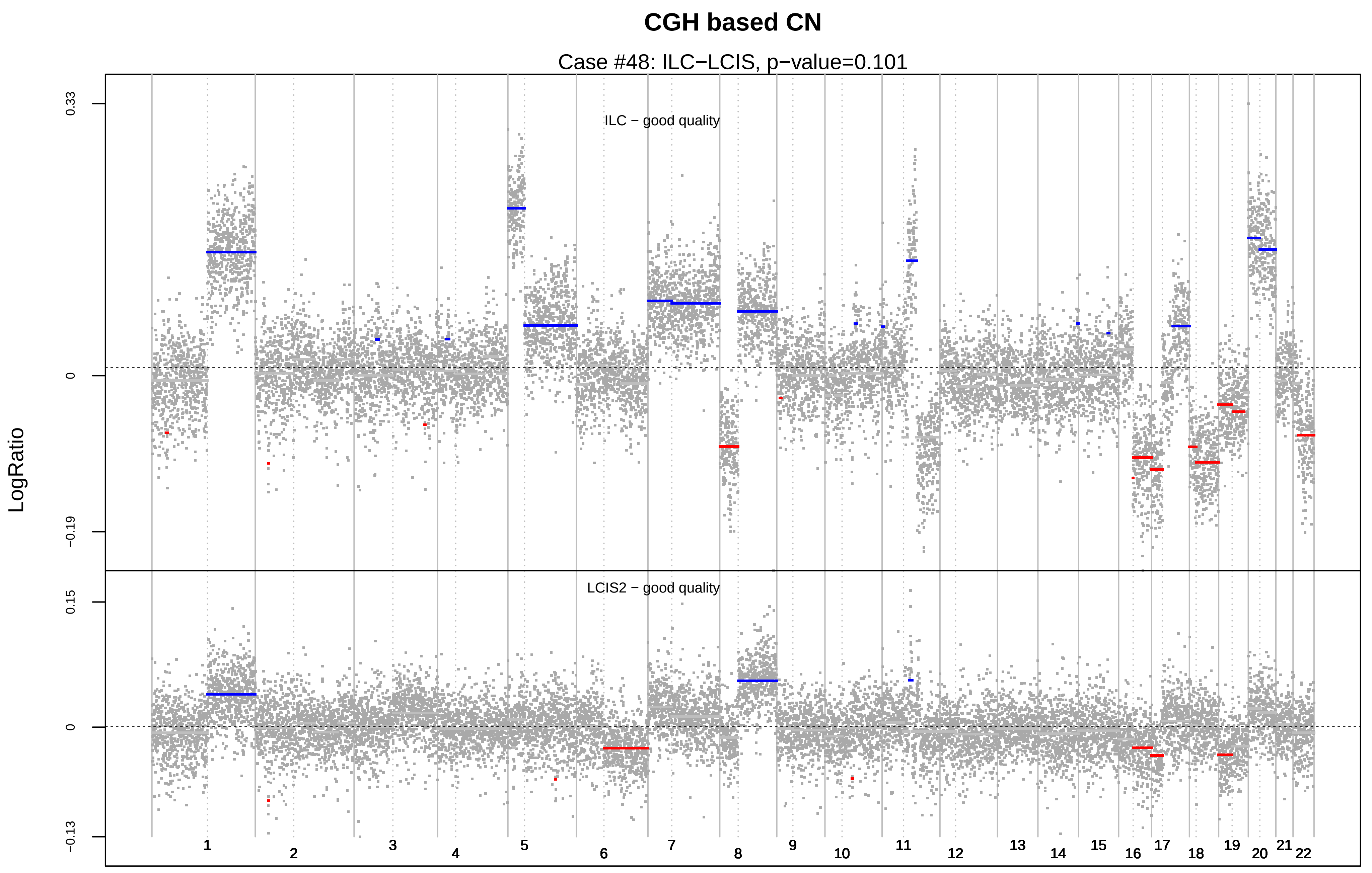
CGH based CN
Case #48: ILC−LCIS, p−value=0.101
ILC − good quality
LCIS2 − good quality
LogRatio
0.33
0
−0.19
0.15
0
−0.13
1
2
3
4
5
6
7
8
9
10
11
12
13
14
15
16
17
18
19
20
21
22

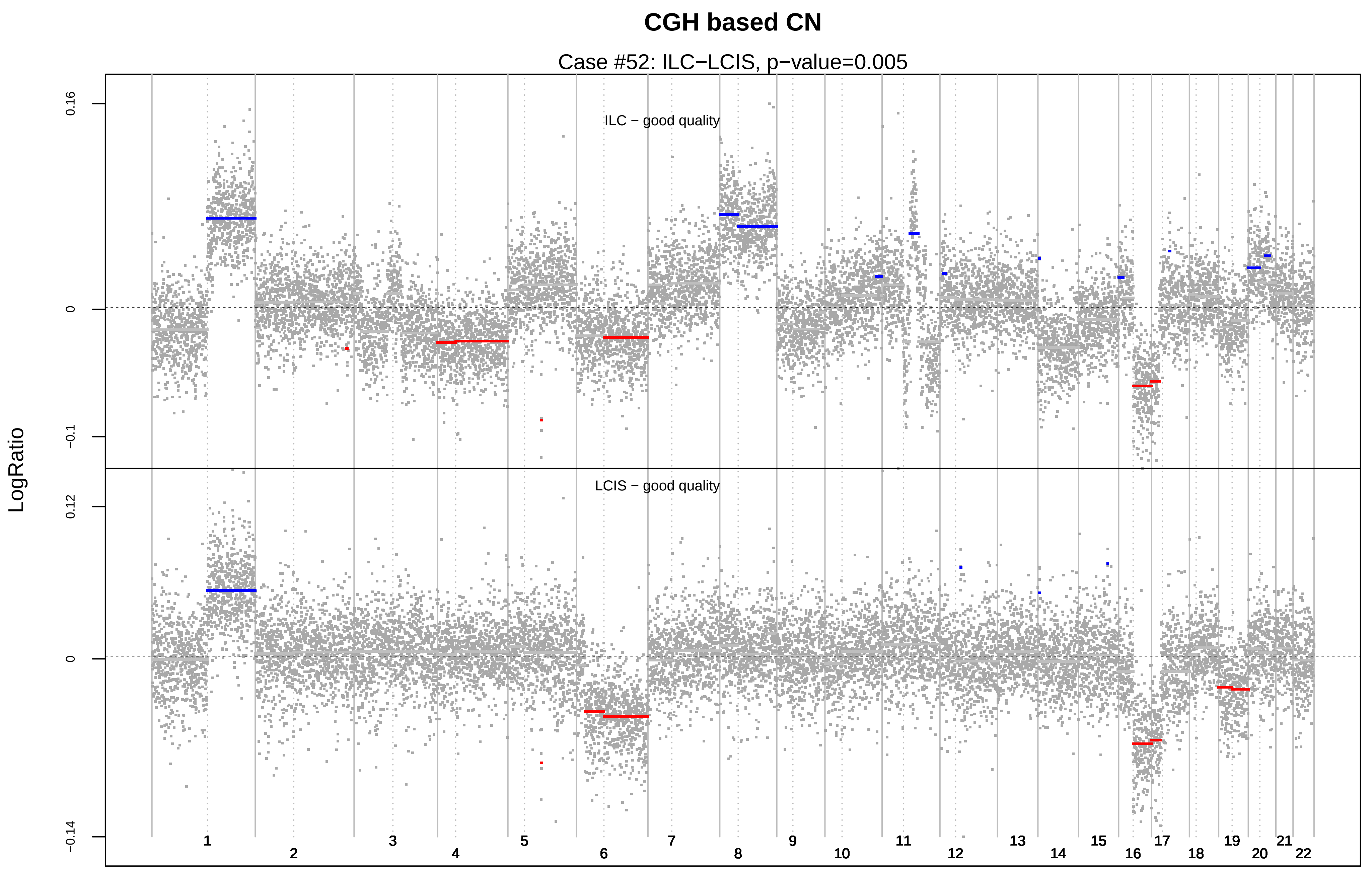
CGH based CN
Case #52: ILC−LCIS, p−value=0.005
LogRatio
ILC − good quality
LCIS − good quality
0.16
0
−0.1
0.12
0
−0.14
1
2
3
4
5
6
7
8
9
10
11
12
13
14
15
16
17
18
19
20
21
22

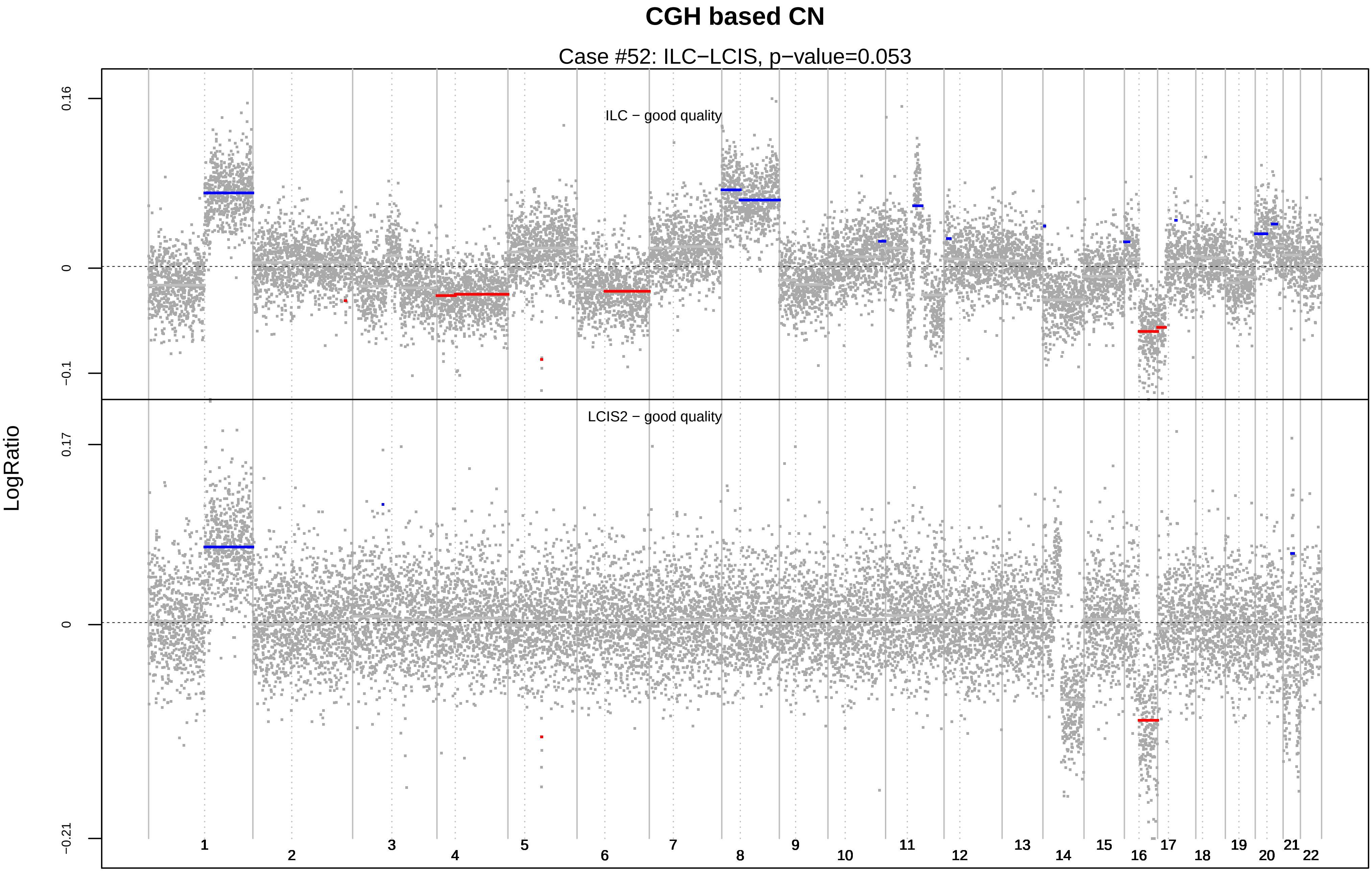

CGH based CN
Case #52: ILC−LCIS, p−value=0.053
ILC − good quality
LCIS2 − good quality
LogRatio
0.16
0
−0.1
0.17
0
−0.21
1
2
3
4
5
6
7
8
9
10
11
12
13
14
15
16
17
18
19
20
21
22

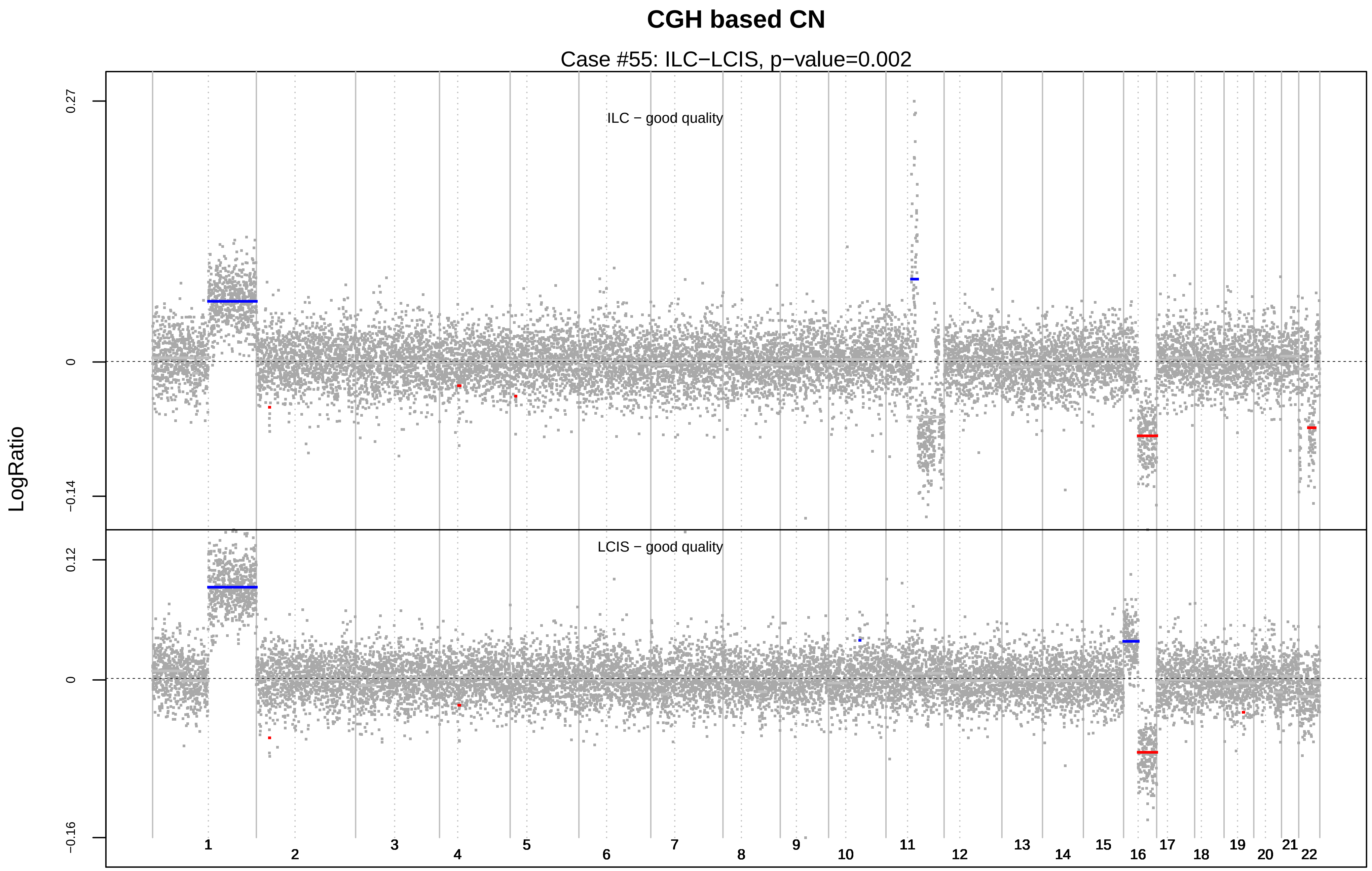
CGH based CN
Case #55: ILC−LCIS, p−value=0.002
ILC − good quality
LCIS − good quality
LogRatio
0.27
0
−0.14
0.12
0
−0.16
1
2
3
4
5
6
7
8
9
10
11
12
13
14
15
16
17
18
19
20
21
22

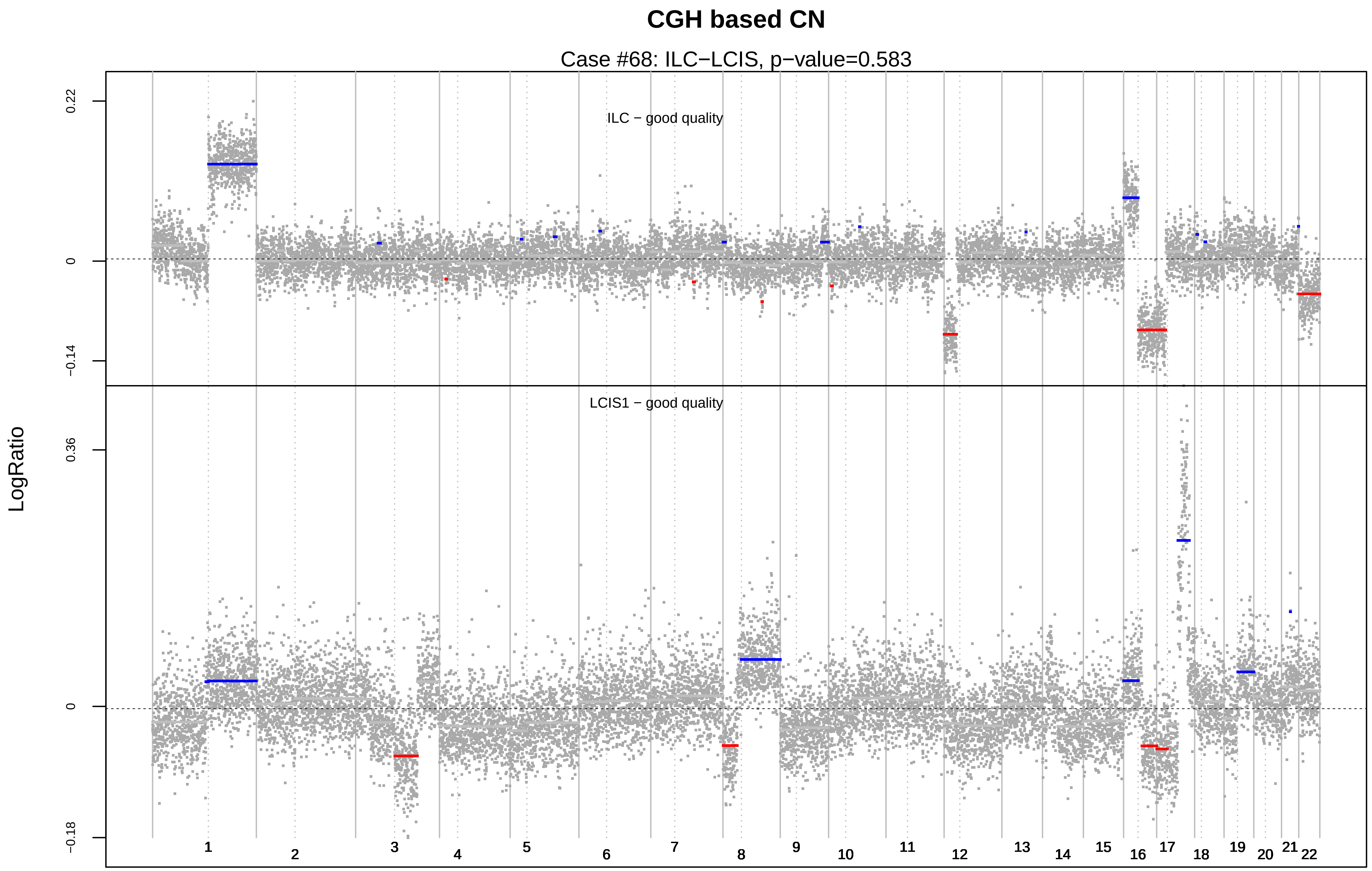

CGH based CN
Case #68: ILC−LCIS, p−value=0.583
ILC − good quality
LCIS1 − good quality
LogRatio
0.22
0
−0.14
0.36
0
−0.18
1
2
3
4
5
6
7
8
9
10
11
12
13
14
15
16
17
18
19
20
21
22

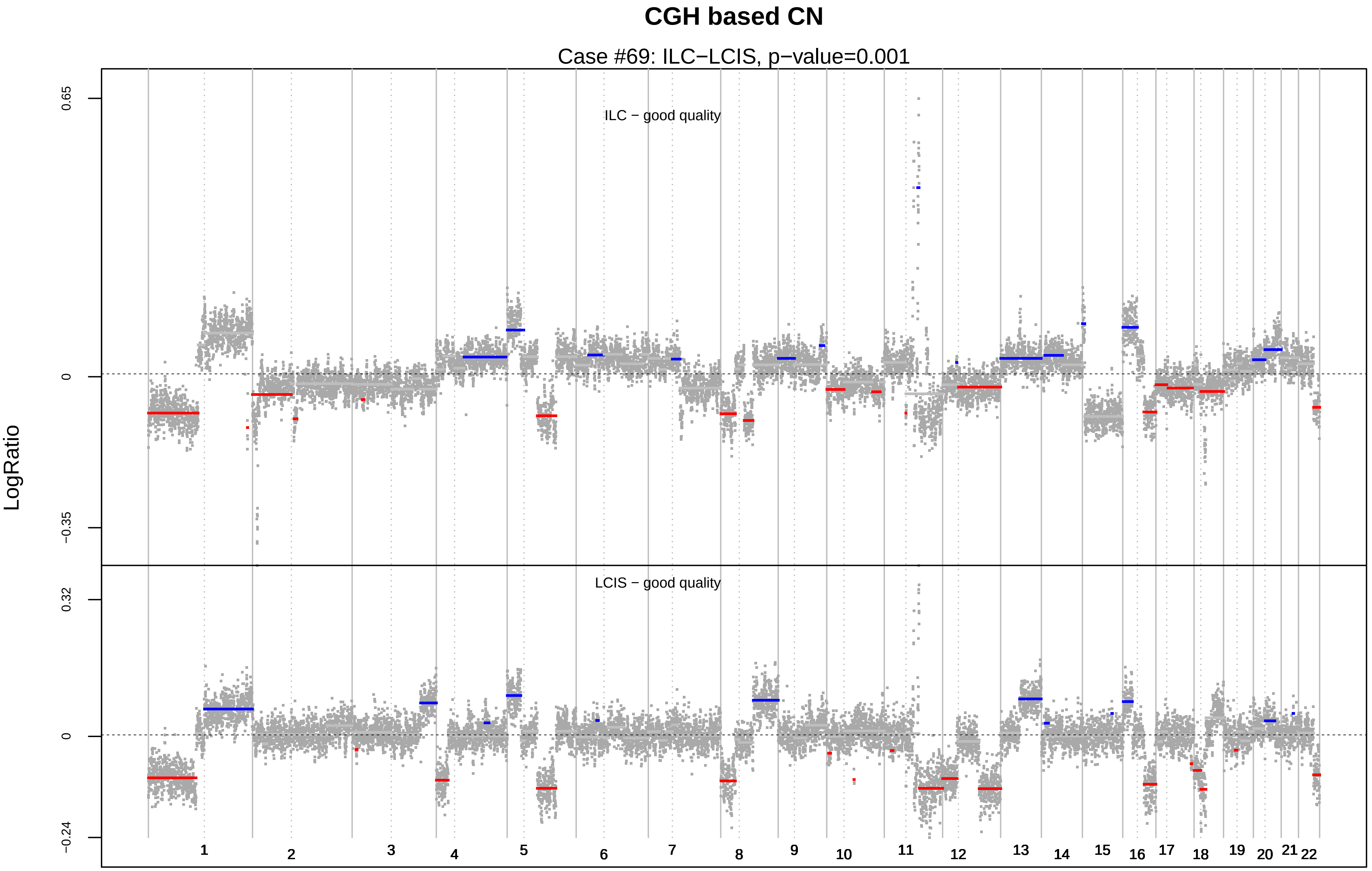

CGH based CN
Case #69: ILC−LCIS, p−value=0.001
ILC − good quality
LCIS − good quality
LogRatio
0.65
0
−0.35
0.32
0
−0.24
1
2
3
4
5
6
7
8
9
10
11
12
13
14
15
16
17
18
19
20
21
22

CGH based CN

Case #47: IDC−LCIS, p−value=0.345

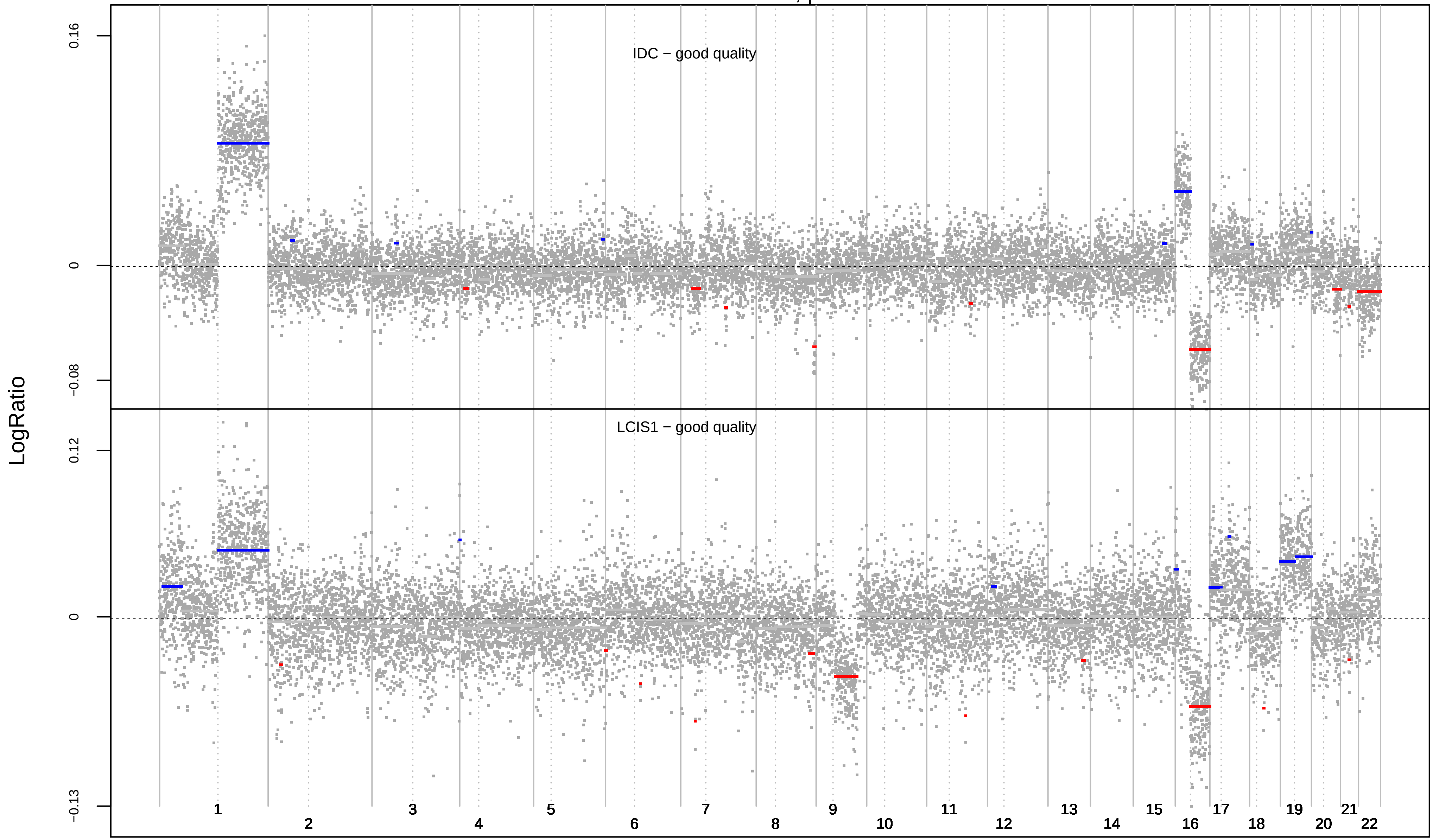

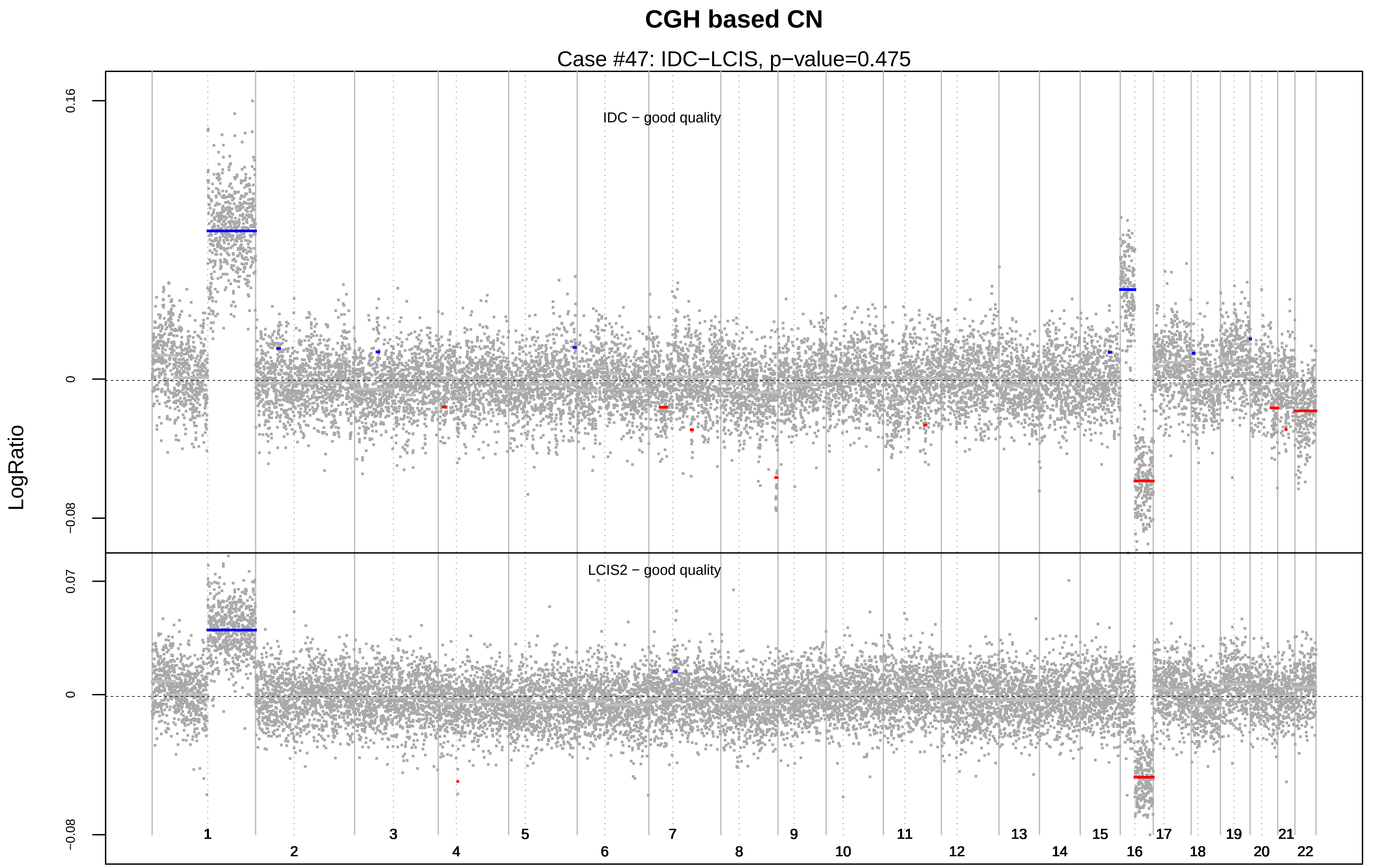
CGH based CN
Case #47: IDC−LCIS, p−value=0.475
IDC − good quality
LCIS2 − good quality
LogRatio
0.16
0
−0.08
0.07
0
−0.08
1
2
3
4
5
6
7
8
9
10
11
12
13
14
15
16
17
18
19
20
21
22

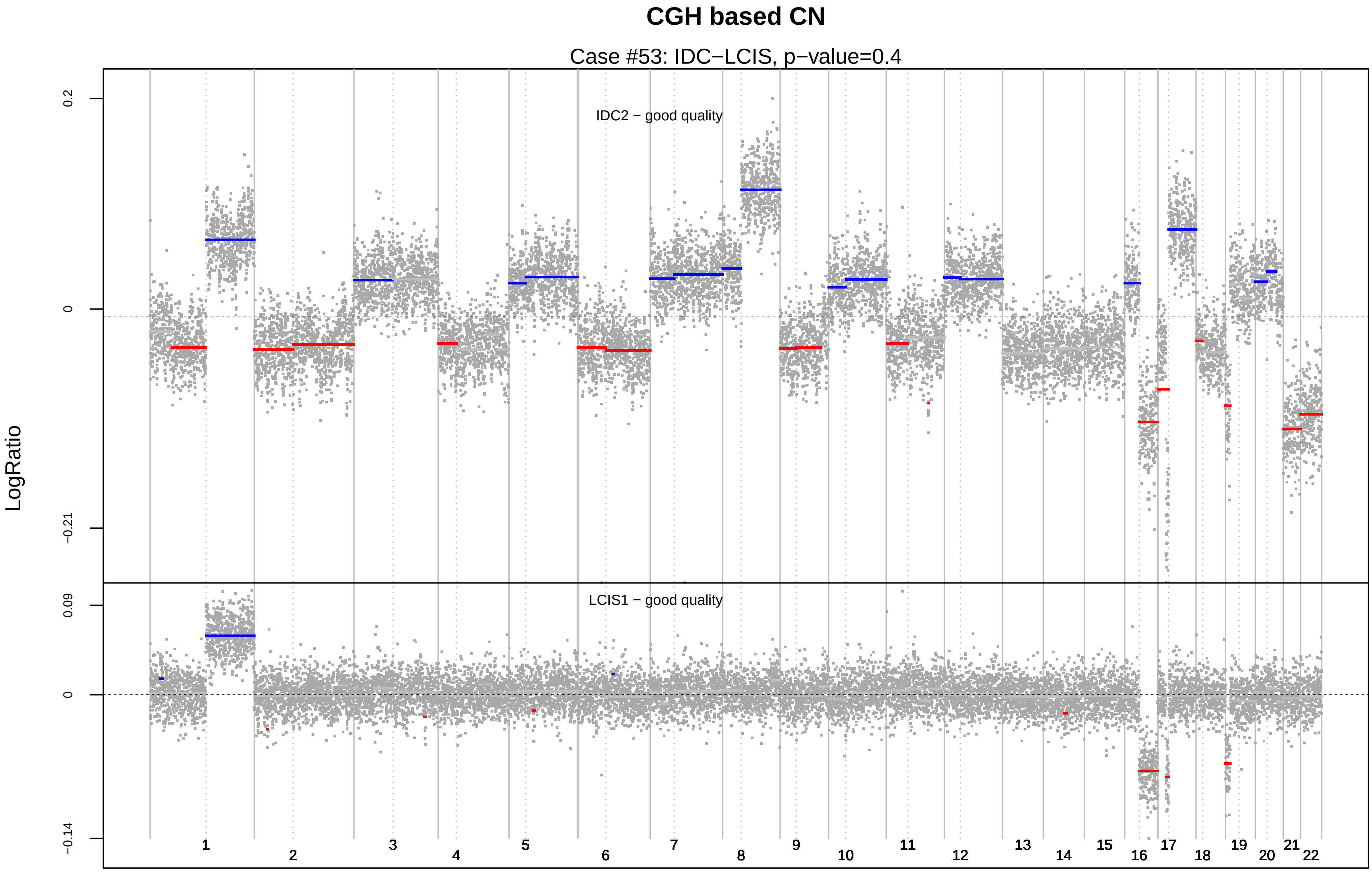

CGH based CN
Case #53: IDC−LCIS, p−value=0.4
IDC2 − good quality
LCIS1 − good quality
LogRatio
0.2
0
−0.21
0.09
0
−0.14
1
2
3
4
5
6
7
8
9
10
11
12
13
14
15
16
17
18
19
20
21
22

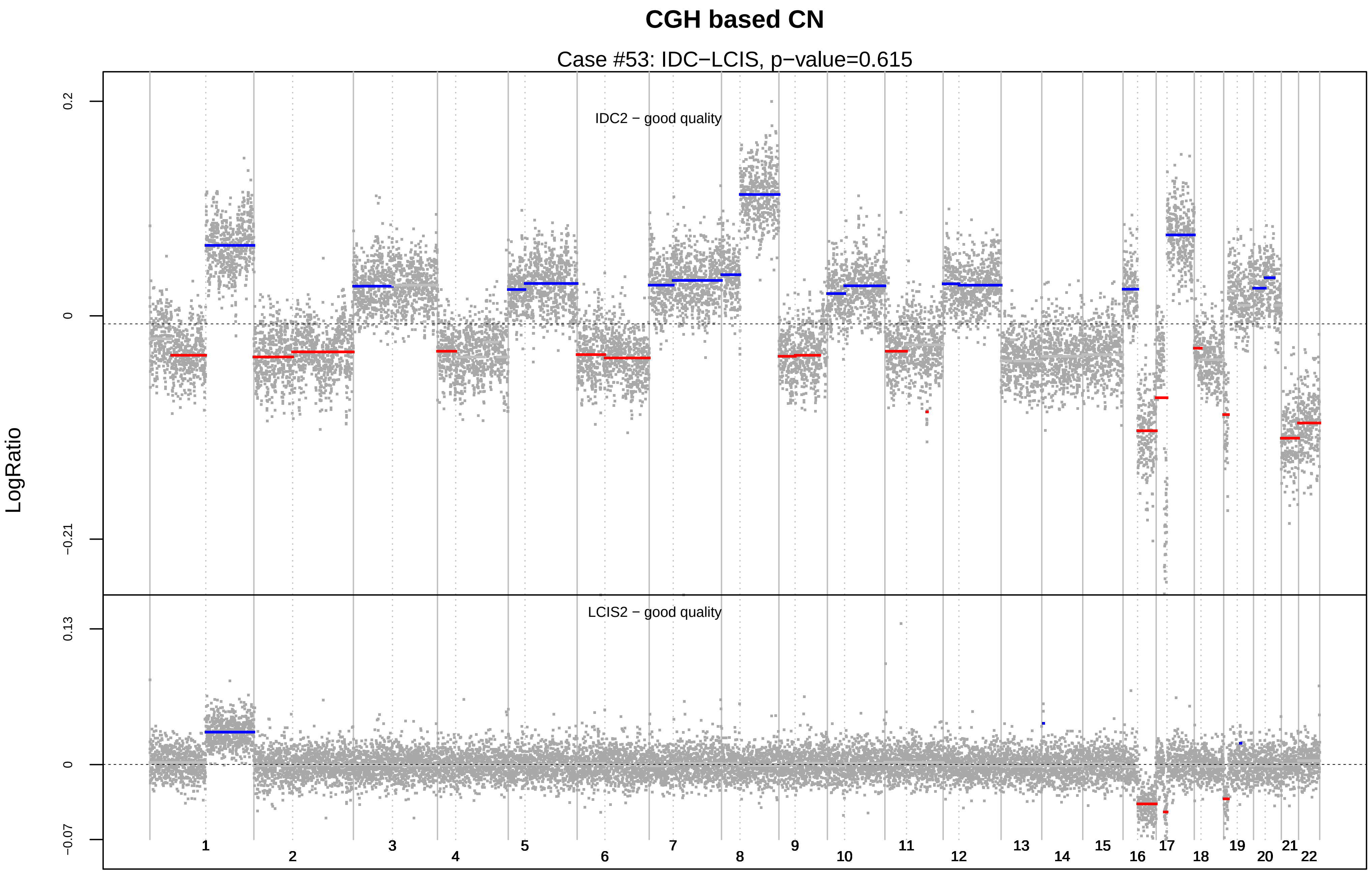

CGH based CN
Case #53: IDC−LCIS, p−value=0.615
IDC2 − good quality
LCIS2 − good quality
LogRatio
0.2
0
−0.21
0.13
0
−0.07
1
2
3
4
5
6
7
8
9
10
11
12
13
14
15
16
17
18
19
20
21
22

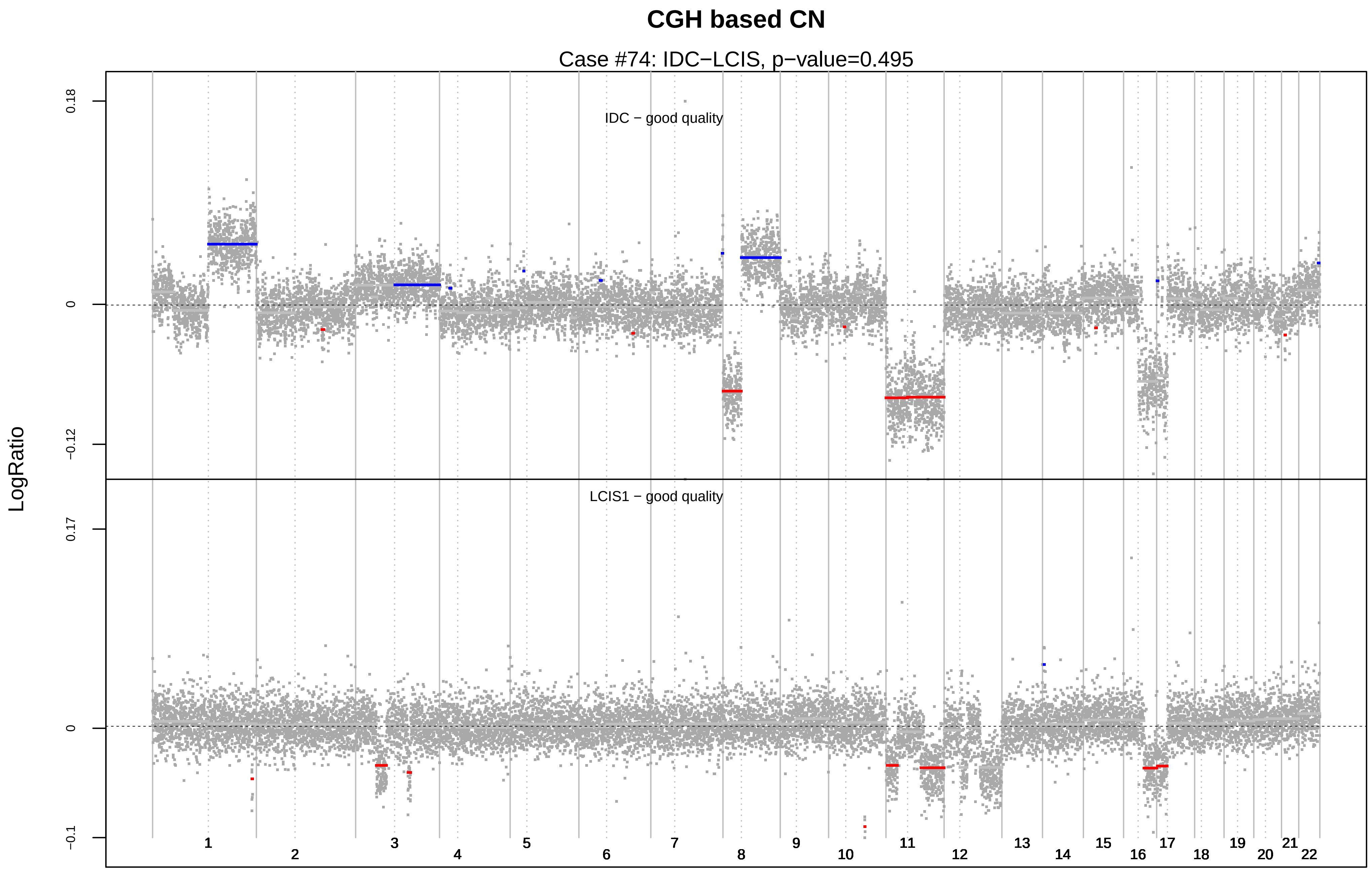
CGH based CN
Case #74: IDC−LCIS, p−value=0.495
IDC − good quality
LCIS1 − good quality
LogRatio
0.18
0
−0.12
0.17
0
−0.1
1
2
3
4
5
6
7
8
9
10
11
12
13
14
15
16
17
18
19
20
21
22

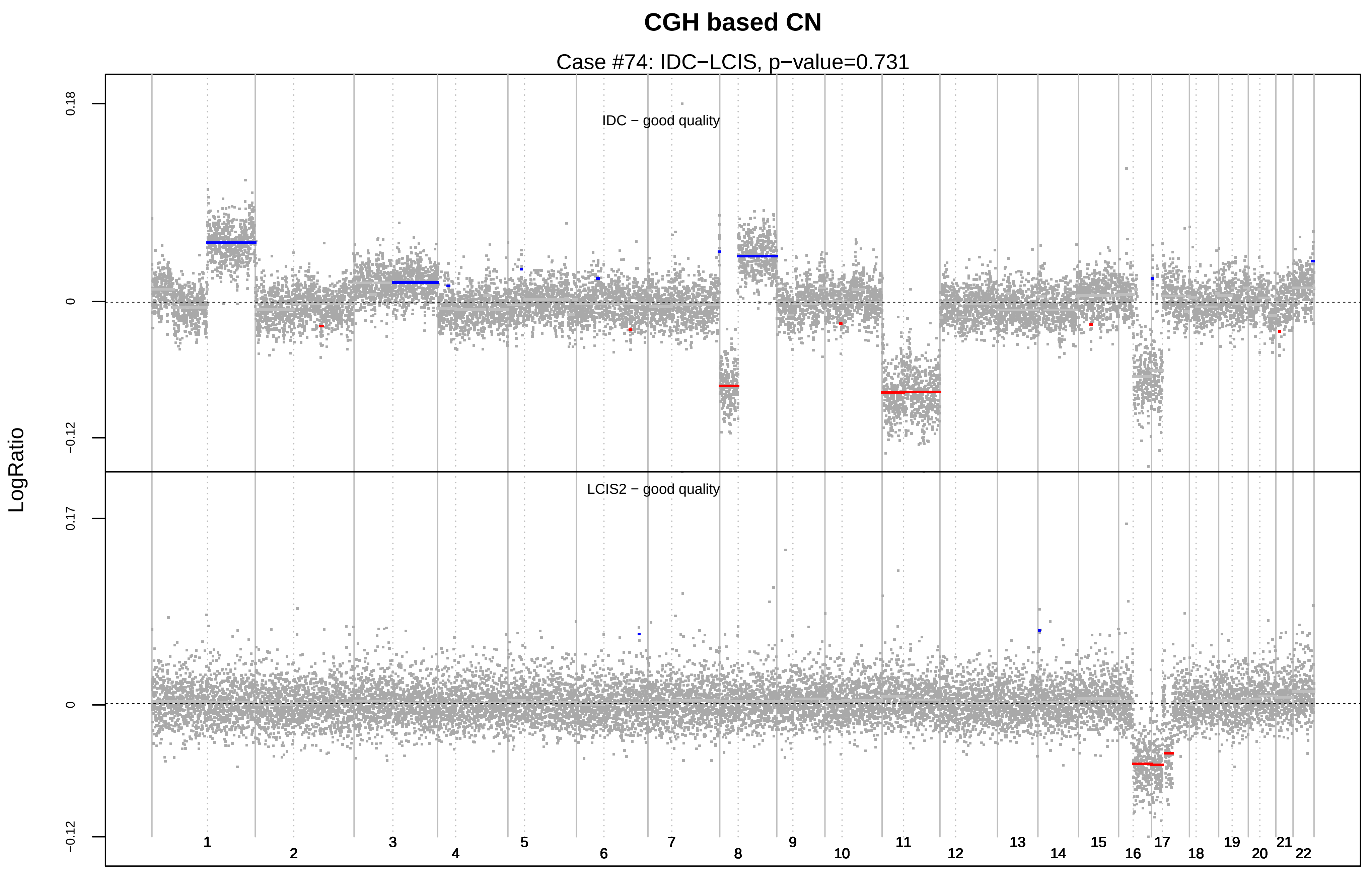
CGH based CN
Case #74: IDC−LCIS, p−value=0.731
IDC − good quality
LCIS2 − good quality
LogRatio
0.18
0
−0.12
0.17
0
−0.12
1
2
3
4
5
6
7
8
9
10
11
12
13
14
15
16
17
18
19
20
21
22

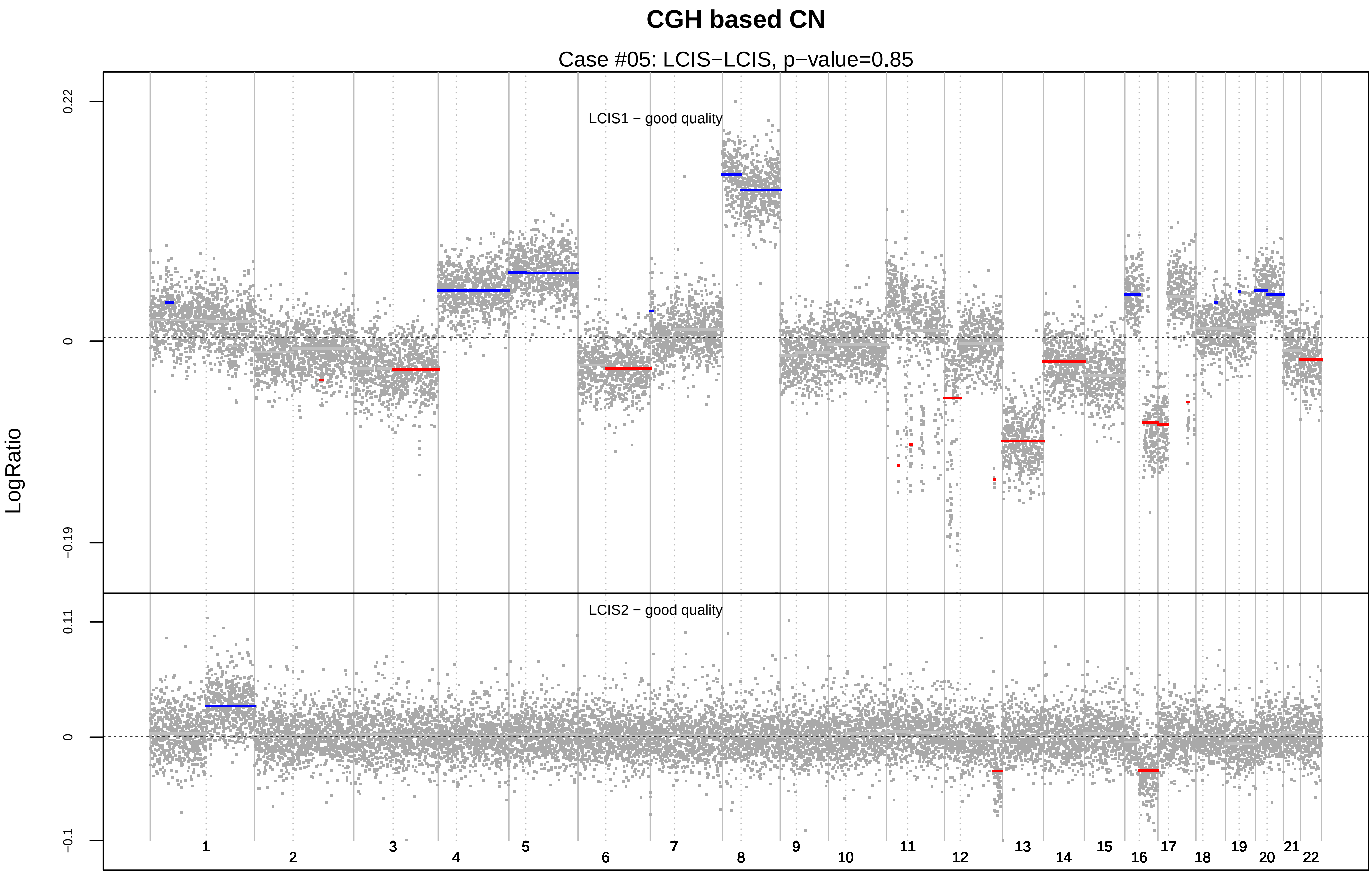
CGH based CN
Case #05: LCIS−LCIS, p−value=0.85
LCIS1 − good quality
LCIS2 − good quality
LogRatio
0.22
0
−0.19
0.11
0
−0.1
1
2
3
4
5
6
7
8
9
10
11
12
13
14
15
16
17
18
19
20
21
22

CGH based CN

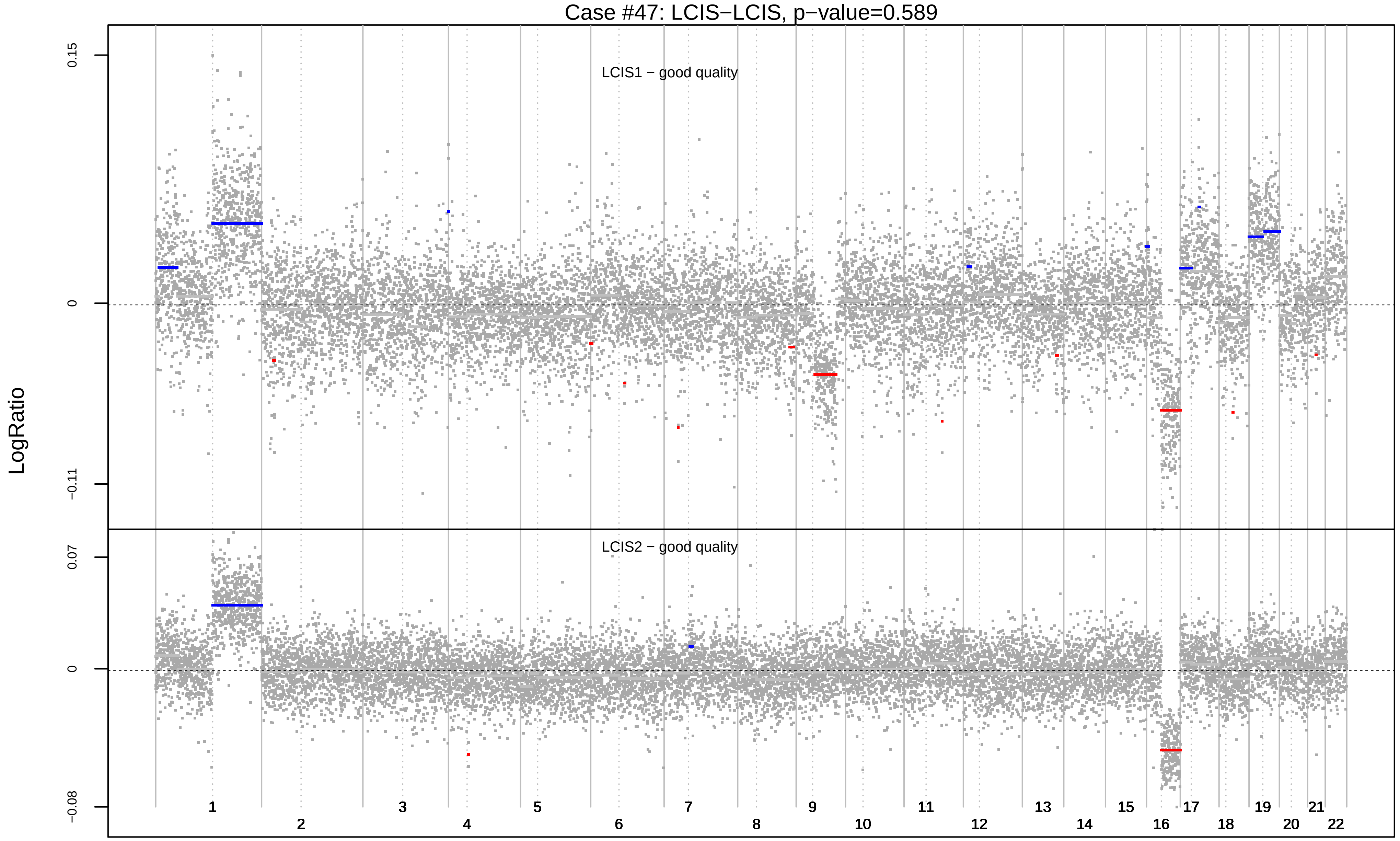

Case #47: LCIS−LCIS, p−value=0.589
LCIS1 − good quality
LCIS2 − good quality
LogRatio
0.15
0
−0.11
0.07
0
−0.08
1
2
3
4
5
6
7
8
9
10
11
12
13
14
15
16
17
18
19
20
21
22

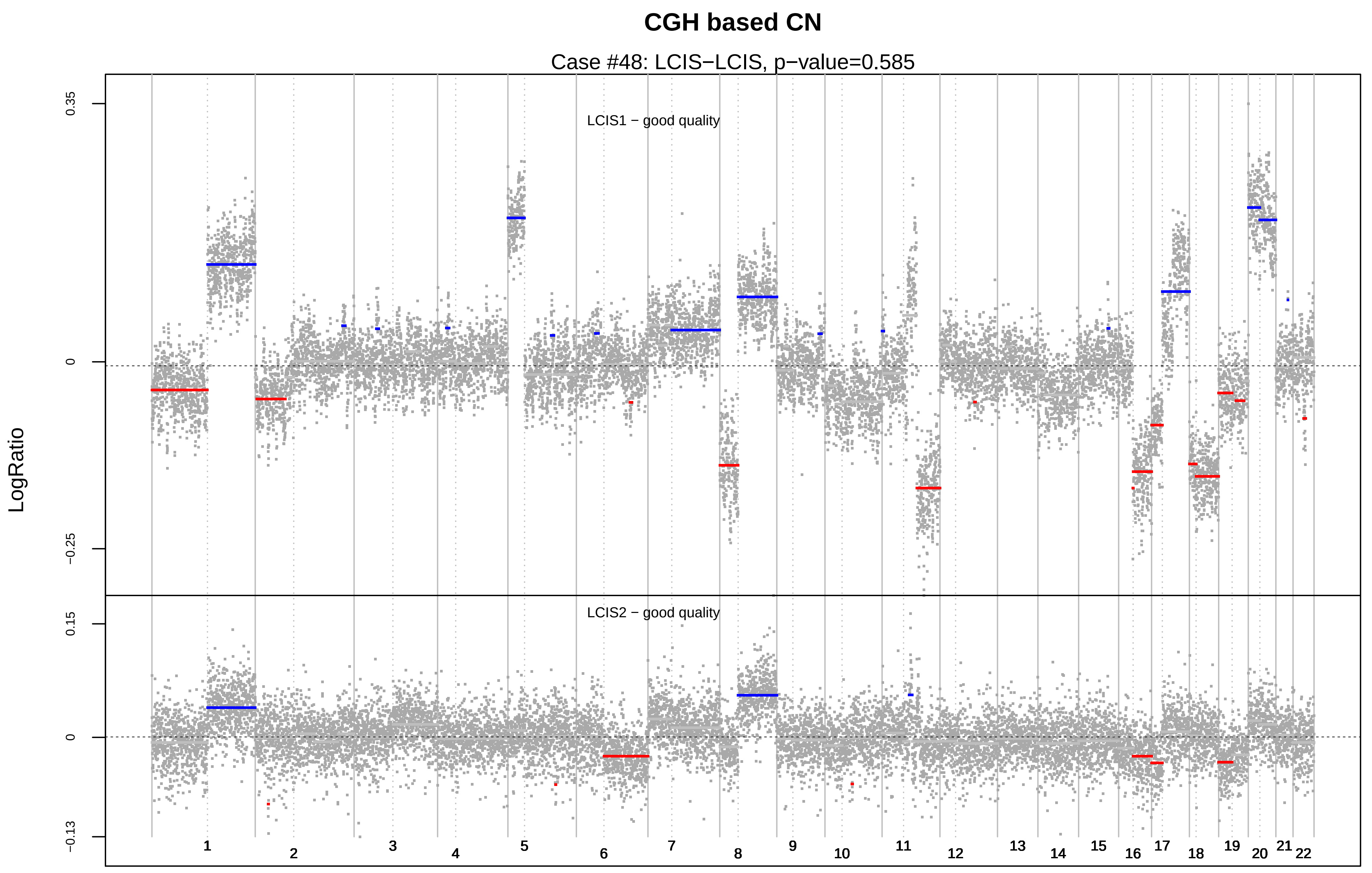
CGH based CN
Case #48: LCIS−LCIS, p−value=0.585
LCIS1 − good quality
LCIS2 − good quality
LogRatio
0.35
0
−0.25
0.15
0
−0.13
1
2
3
4
5
6
7
8
9
10
11
12
13
14
15
16
17
18
19
20
21
22

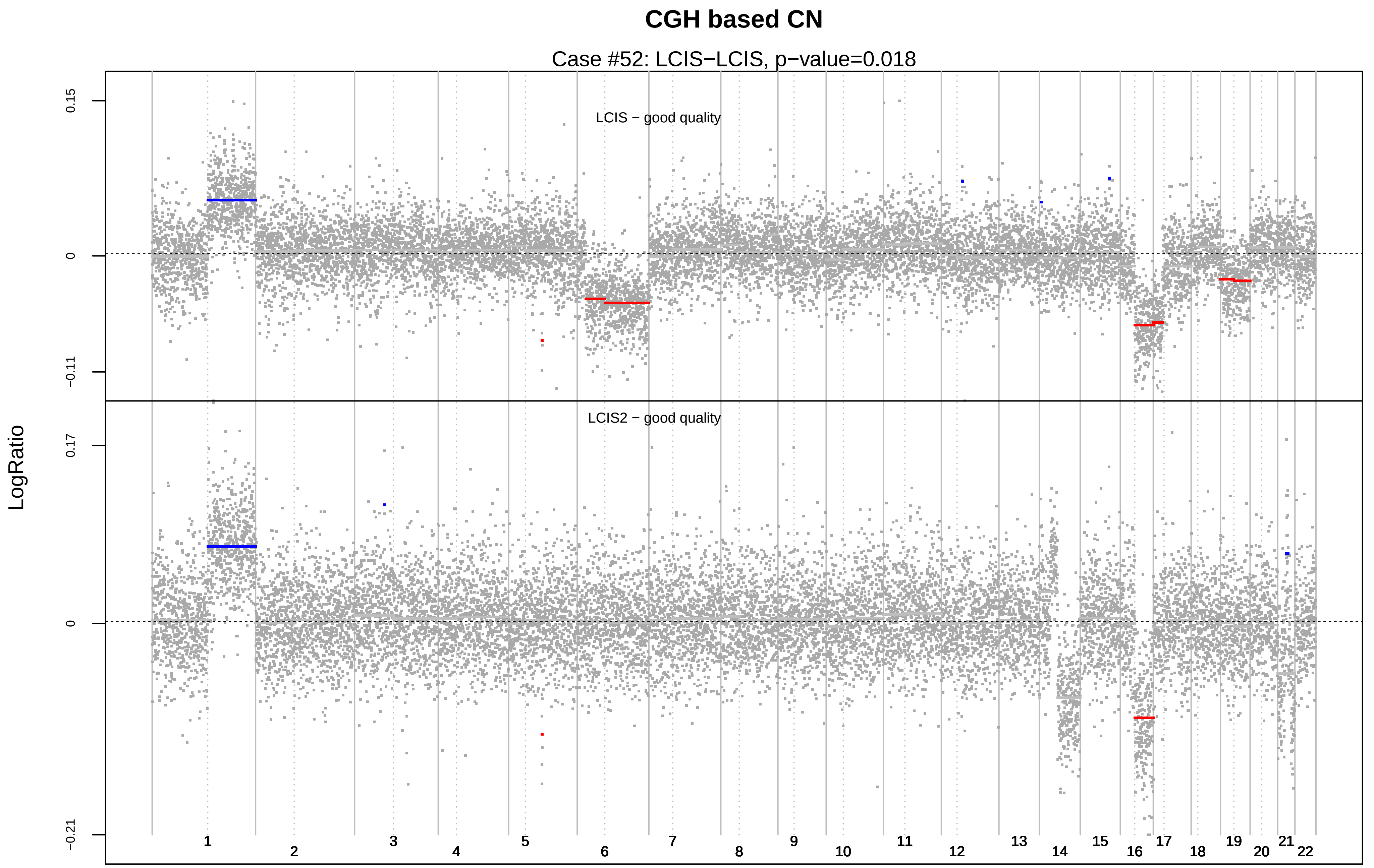
CGH based CN
Case #52: LCIS−LCIS, p−value=0.018
LCIS − good quality
LCIS2 − good quality
LogRatio
0.15
0
−0.11
0.17
0
−0.21
1
2
3
4
5
6
7
8
9
10
11
12
13
14
15
16
17
18
19
20
21
22

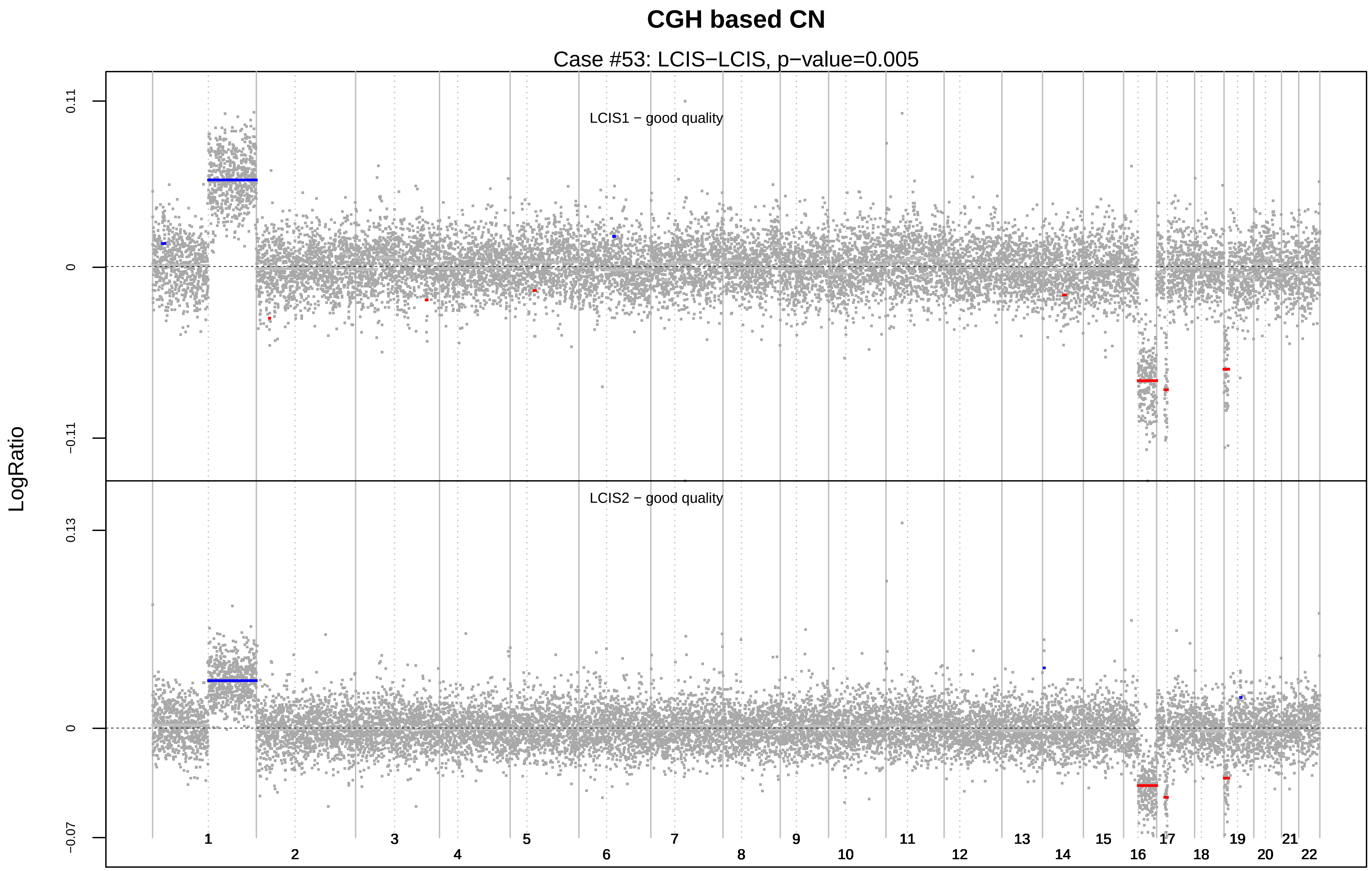
CGH based CN
Case #53: LCIS−LCIS, p−value=0.005
LCIS1 − good quality
LCIS2 − good quality
LogRatio
0.11
0
−0.11
0.13
0
−0.07
1
2
3
4
5
6
7
8
9
10
11
12
13
14
15
16
17
18
19
20
21
22

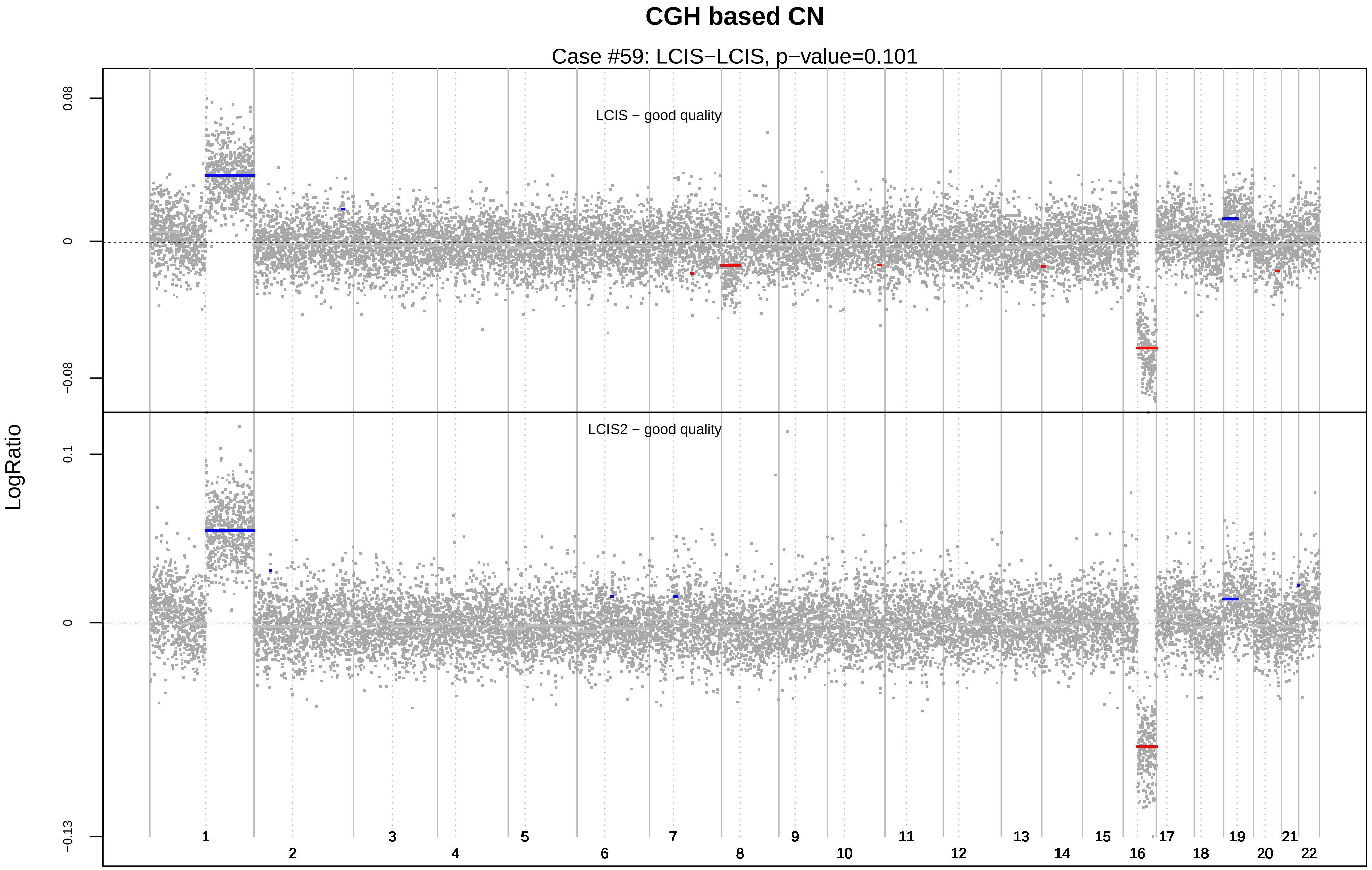

CGH based CN
Case #59: LCIS−LCIS, p−value=0.101
LCIS − good quality
LCIS2 − good quality
LogRatio
0.08
0
−0.08
0.1
0
−0.13
1
2
3
4
5
6
7
8
9
10
11
12
13
14
15
16
17
18
19
20
21
22

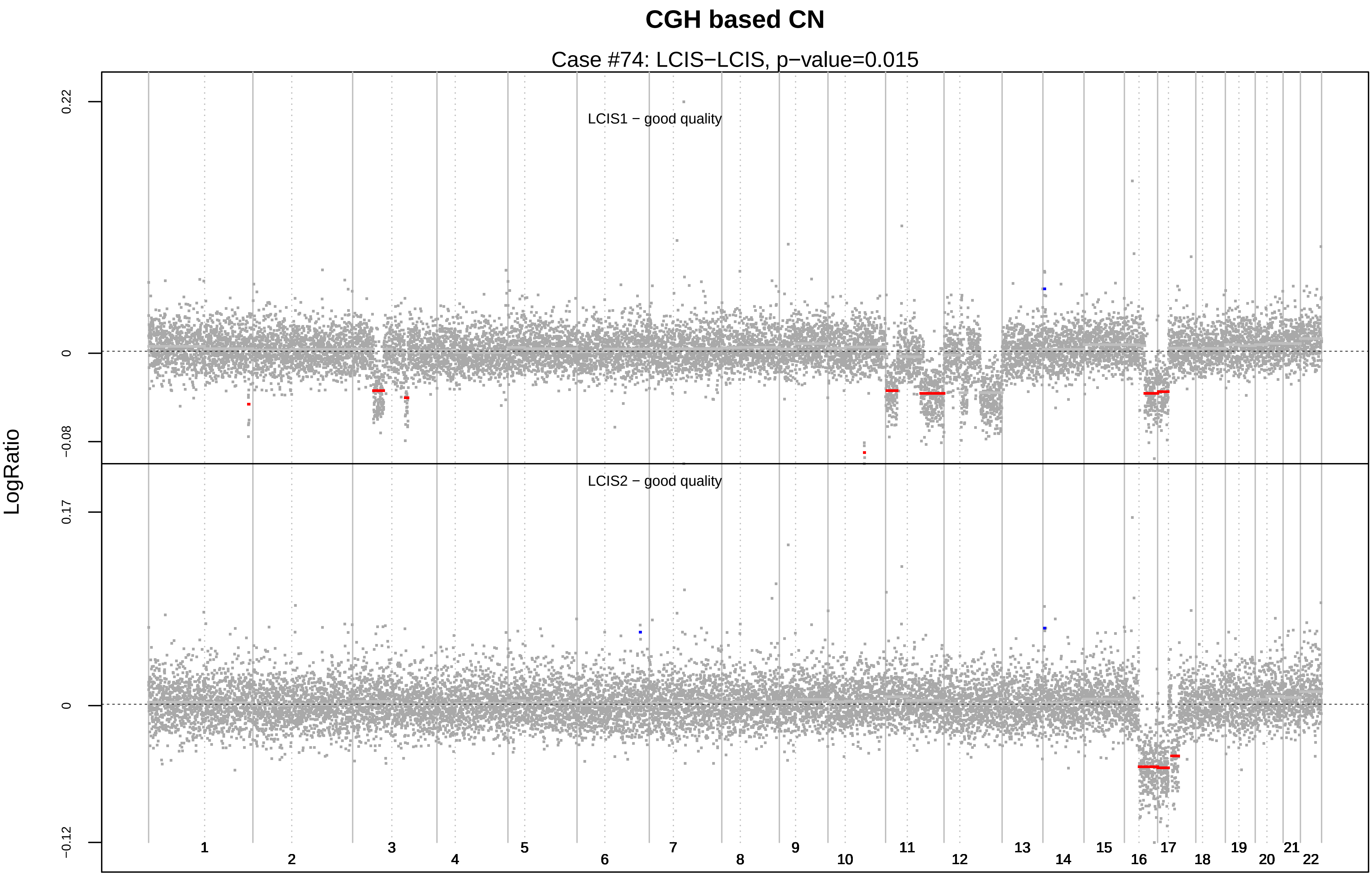
CGH based CN
Case #74: LCIS−LCIS, p−value=0.015
LCIS1 − good quality
LCIS2 − good quality
LogRatio
0.22
0
−0.08
0.17
0
−0.12
1
2
3
4
5
6
7
8
9
10
11
12
13
14
15
16
17
18
19
20
21
22

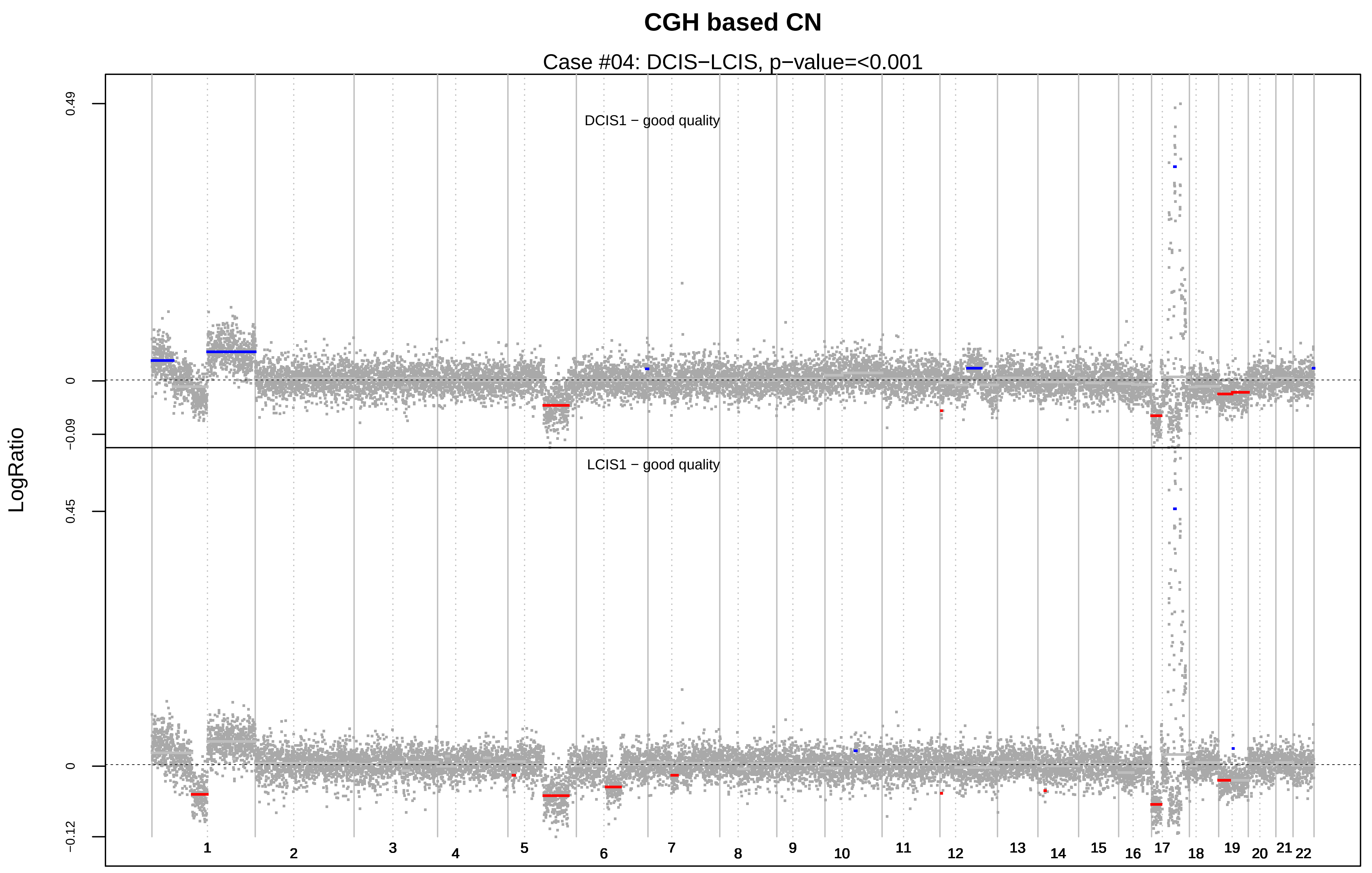

CGH based CN
Case #04: DCIS−LCIS, p−value=<0.001
DCIS1 − good quality
LCIS1 − good quality
LogRatio
0.49
0
−0.09
0.45
0
−0.12
1
2
3
4
5
6
7
8
9
10
11
12
13
14
15
16
17
18
19
20
21
22

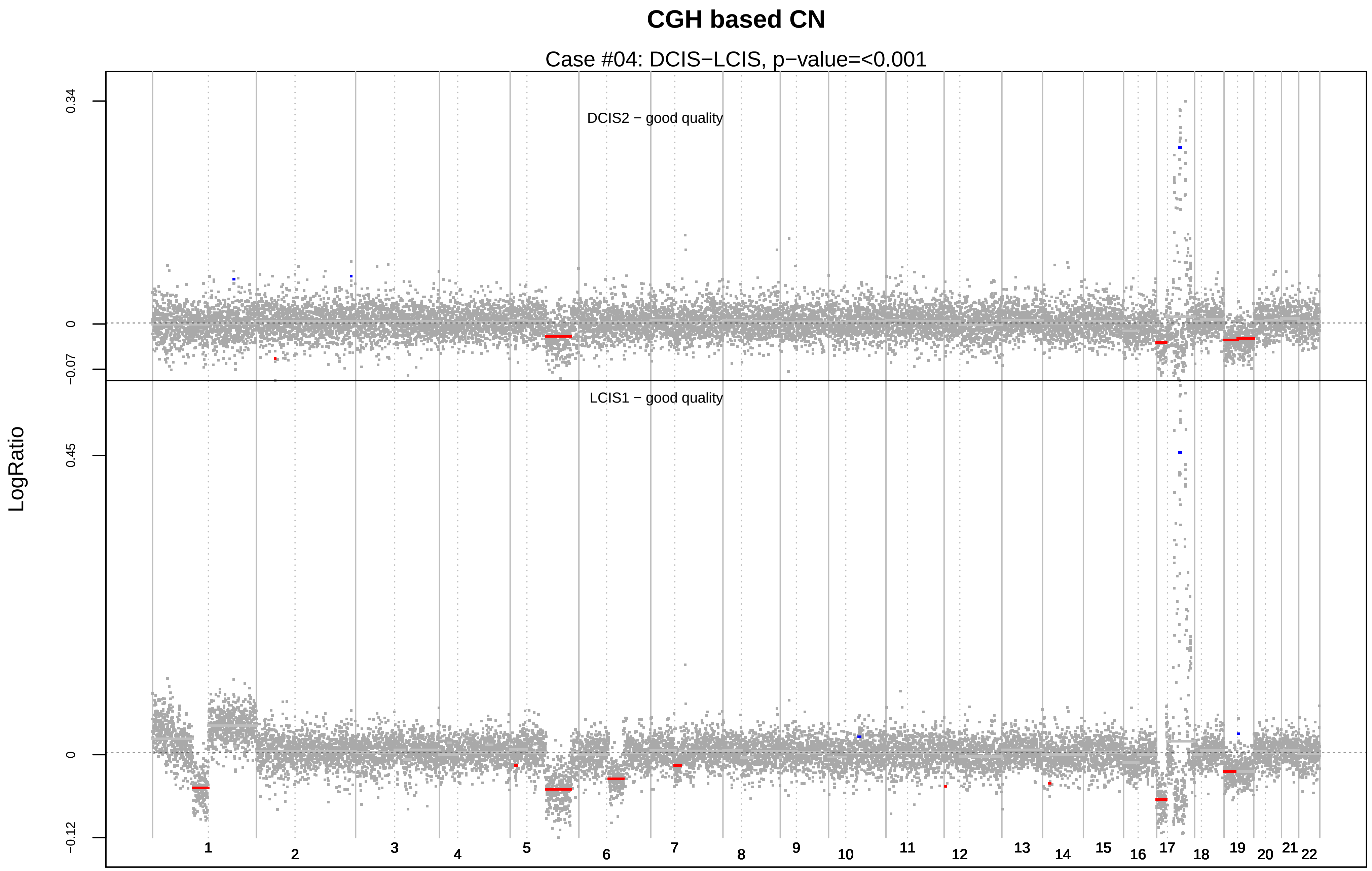

CGH based CN
Case #04: DCIS−LCIS, p−value=<0.001
DCIS2 − good quality
LCIS1 − good quality
LogRatio
0.34
0
−0.07
0.45
0
−0.12
1
2
3
4
5
6
7
8
9
10
11
12
13
14
15
16
17
18
19
20
21
22

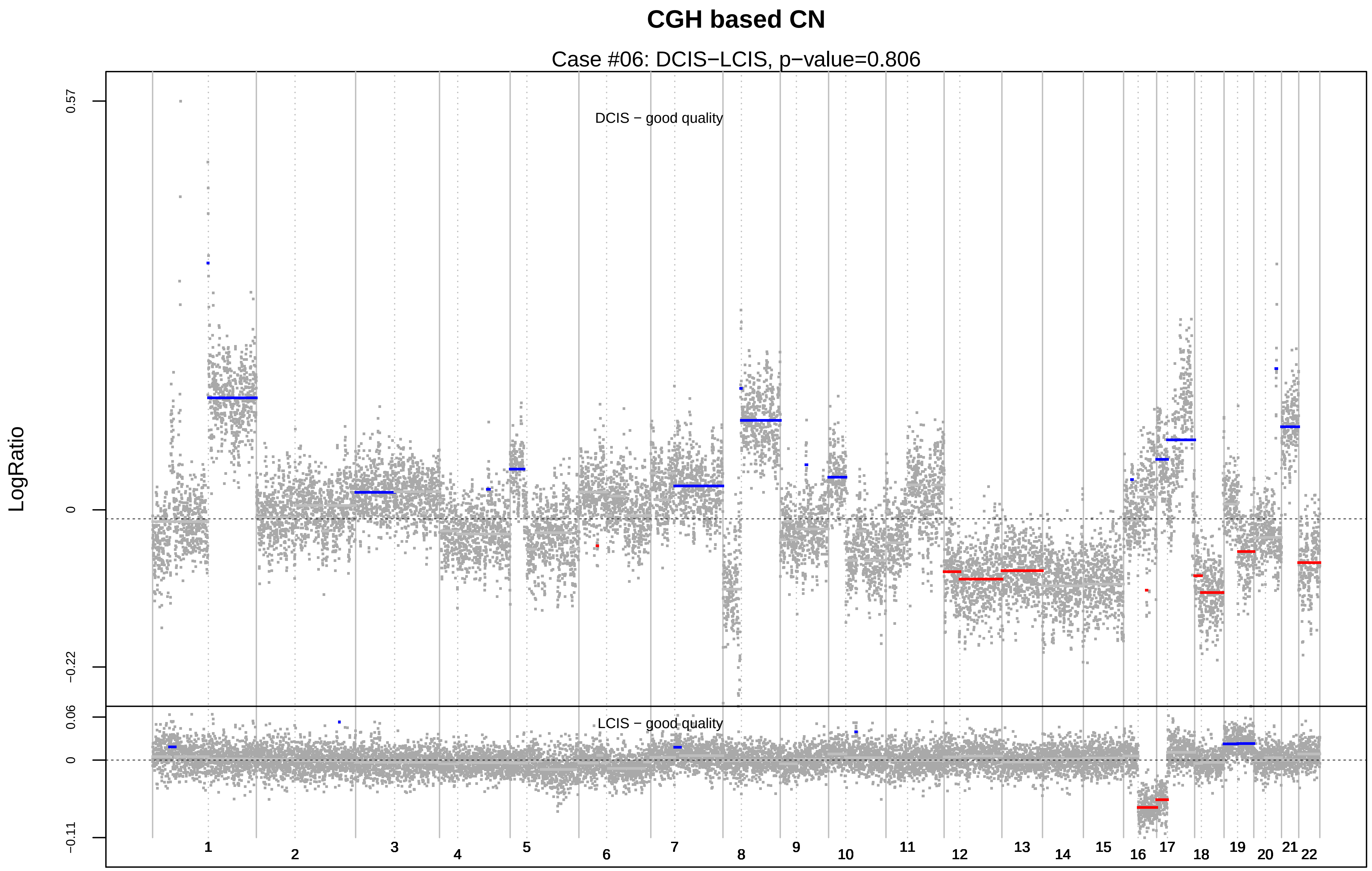
CGH based CN
Case #06: DCIS−LCIS, p−value=0.806
DCIS − good quality
LCIS − good quality
LogRatio
0.57
0
−0.22
0.06
0
−0.11
1
2
3
4
5
6
7
8
9
10
11
12
13
14
15
16
17
18
19
20
21
22

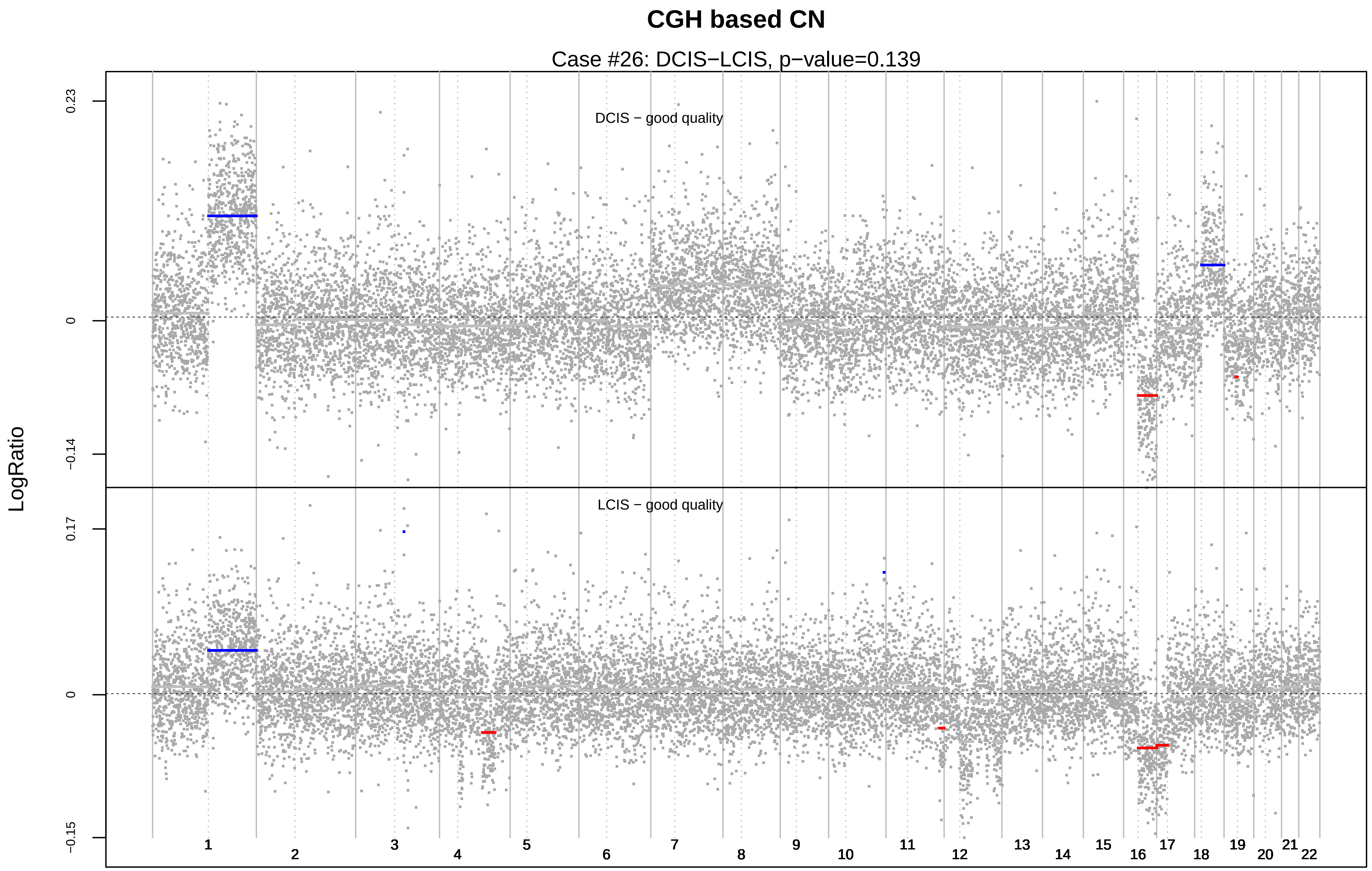

CGH based CN
Case #26: DCIS−LCIS, p−value=0.139
DCIS − good quality
LCIS − good quality
LogRatio
0.23
0
−0.14
0.17
0
−0.15
1
2
3
4
5
6
7
8
9
10
11
12
13
14
15
16
17
18
19
20
21
22

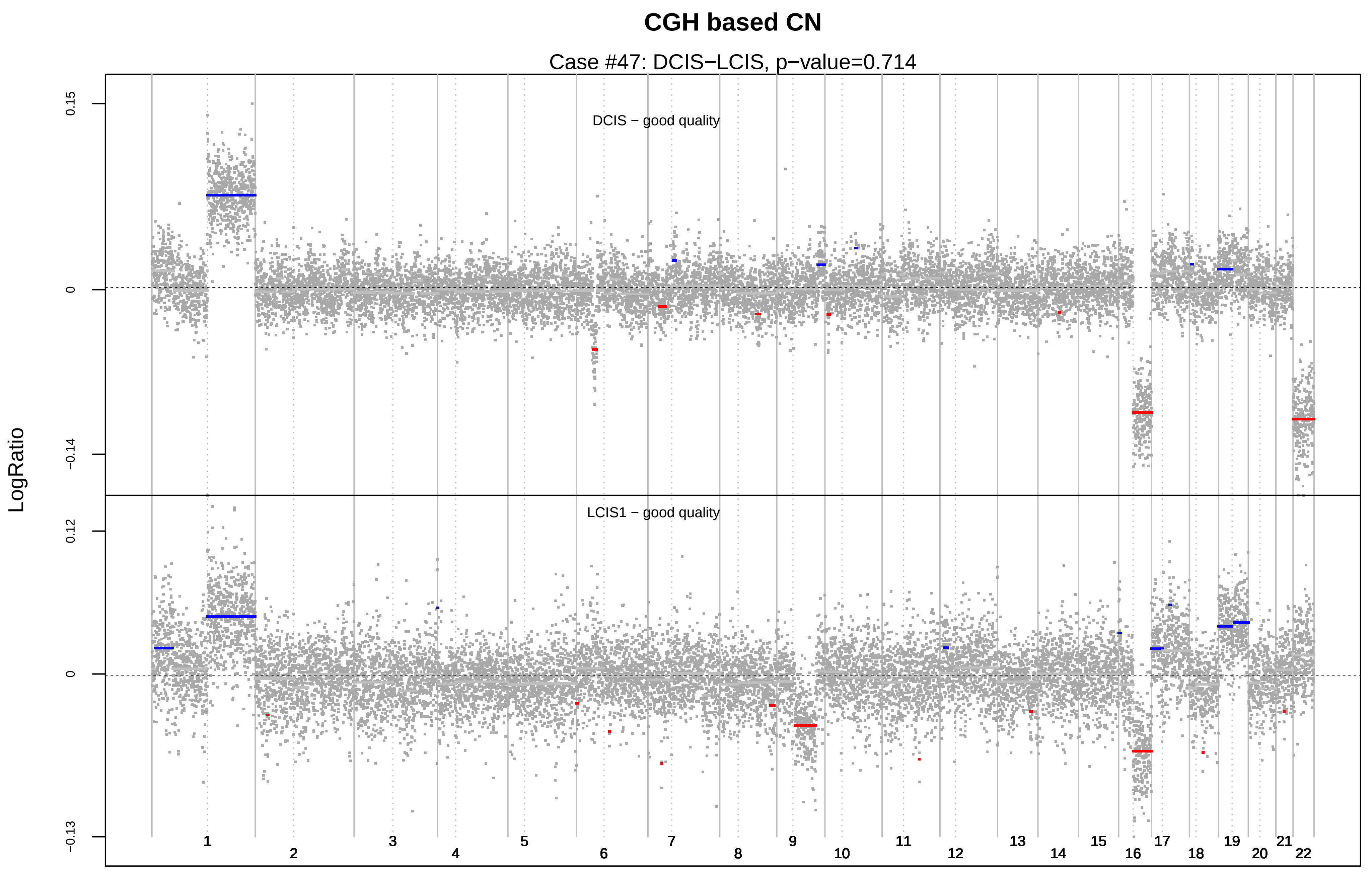
CGH based CN
Case #47: DCIS−LCIS, p−value=0.714
DCIS − good quality
LCIS1 − good quality
LogRatio
0.15
0
−0.14
0.12
0
−0.13
1
2
3
4
5
6
7
8
9
10
11
12
13
14
15
16
17
18
19
20
21
22

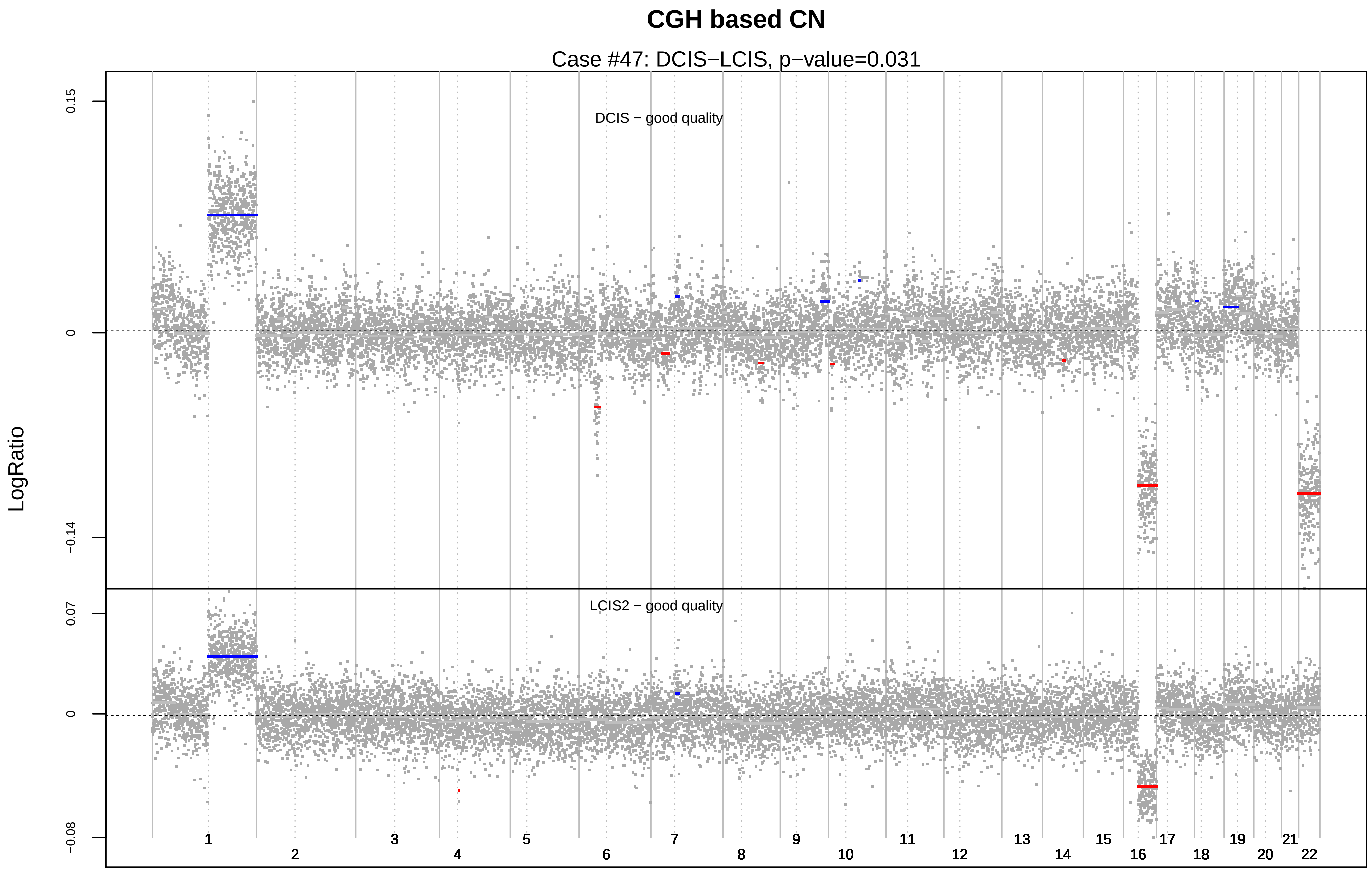

CGH based CN
Case #47: DCIS−LCIS, p−value=0.031
DCIS − good quality
LCIS2 − good quality
LogRatio
0.15
0
−0.14
0.07
0
−0.08
1
2
3
4
5
6
7
8
9
10
11
12
13
14
15
16
17
18
19
20
21
22

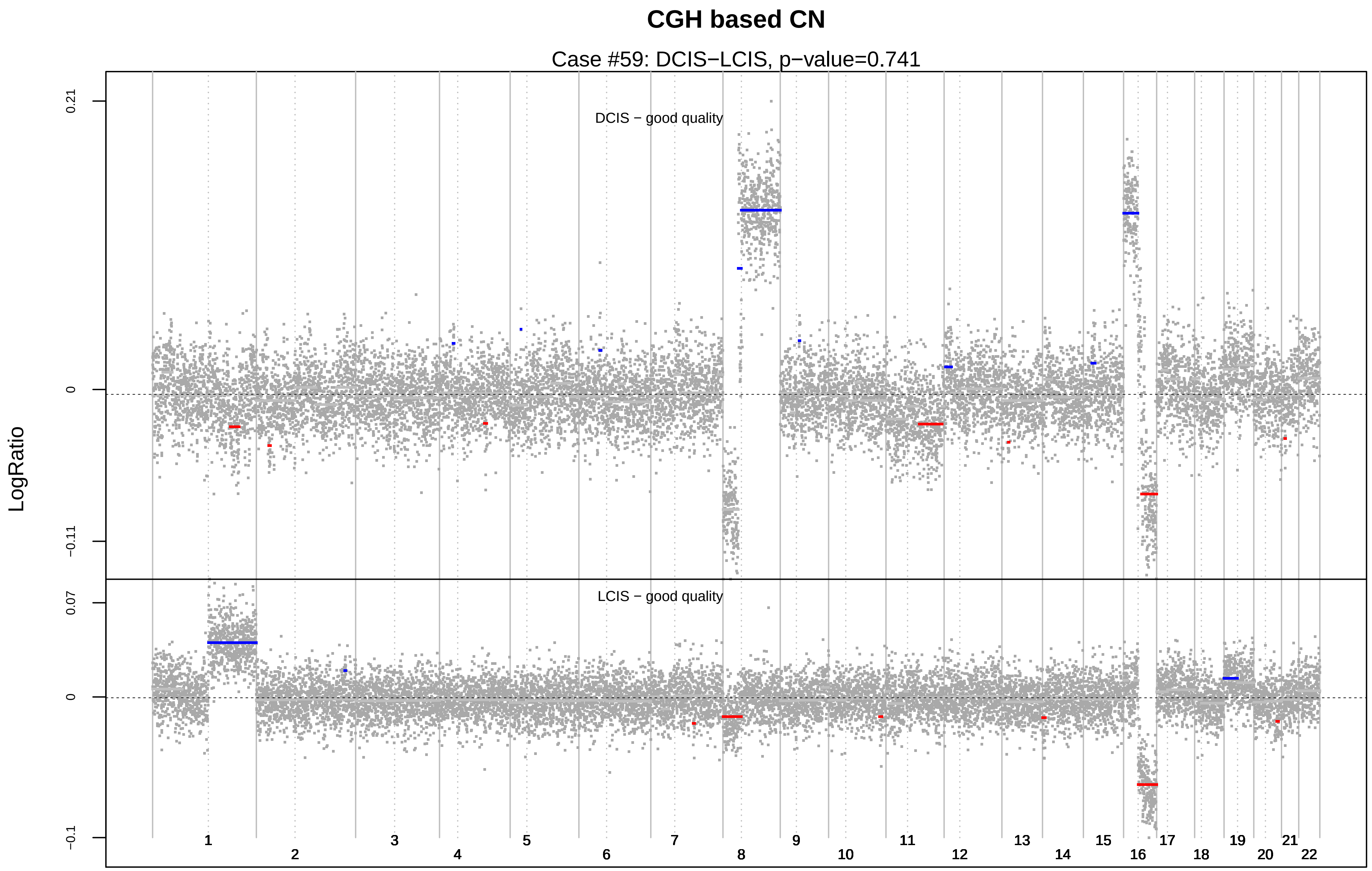

CGH based CN
Case #59: DCIS−LCIS, p−value=0.741
DCIS − good quality
LCIS − good quality
LogRatio
0.21
0
−0.11
0.07
0
−0.1
1 2 3 4 5 6 7 8 9 10 11 12 13 14 15 16 17 18 19 20 21 22

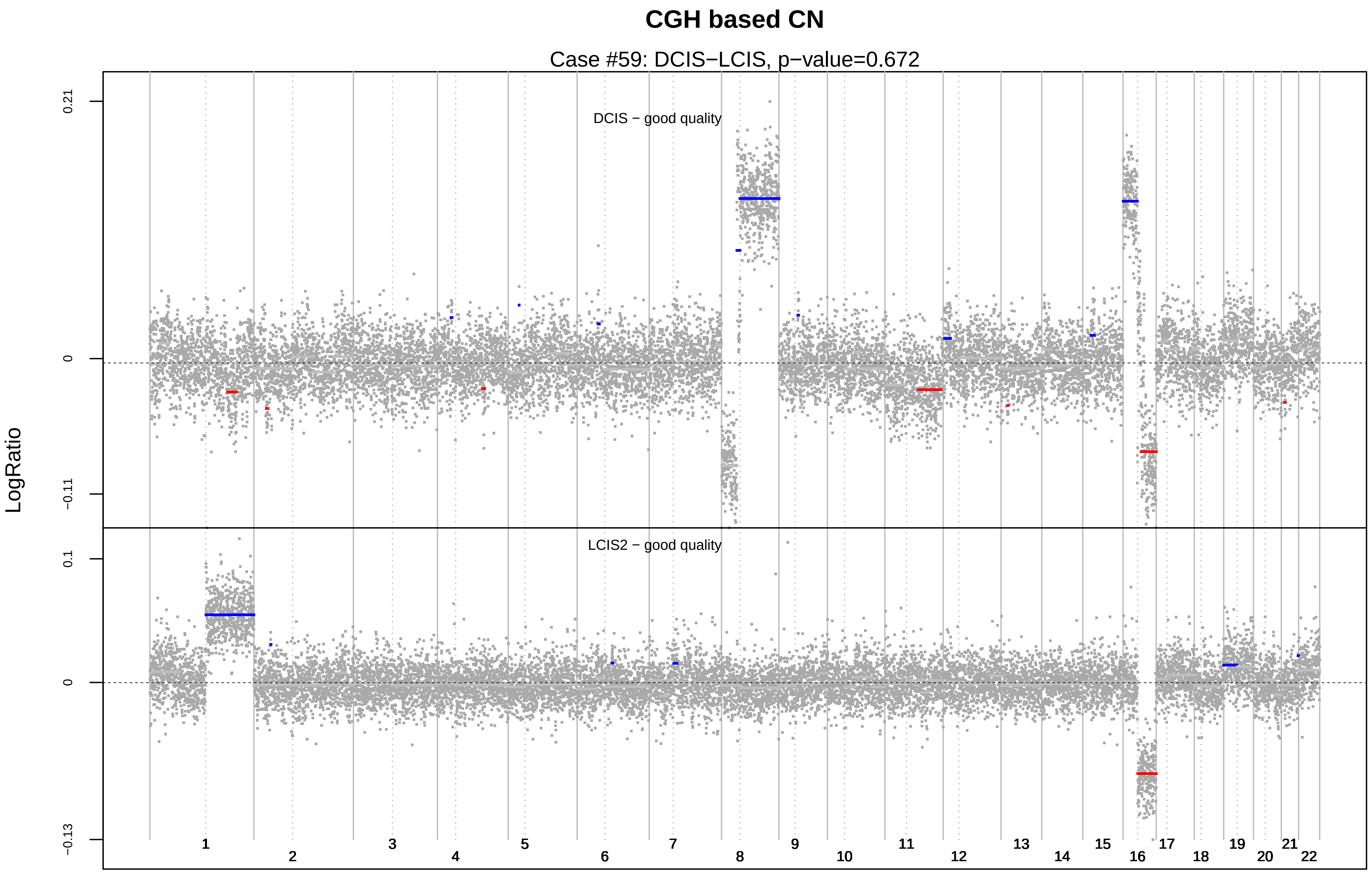

CGH based CN
Case #59: DCIS−LCIS, p−value=0.672
DCIS − good quality
LCIS2 − good quality
LogRatio
0.21
0
−0.11
0.1
0
−0.13
1 2 3 4 5 6 7 8 9 10 11 12 13 14 15 16 17 18 19 20 21 22

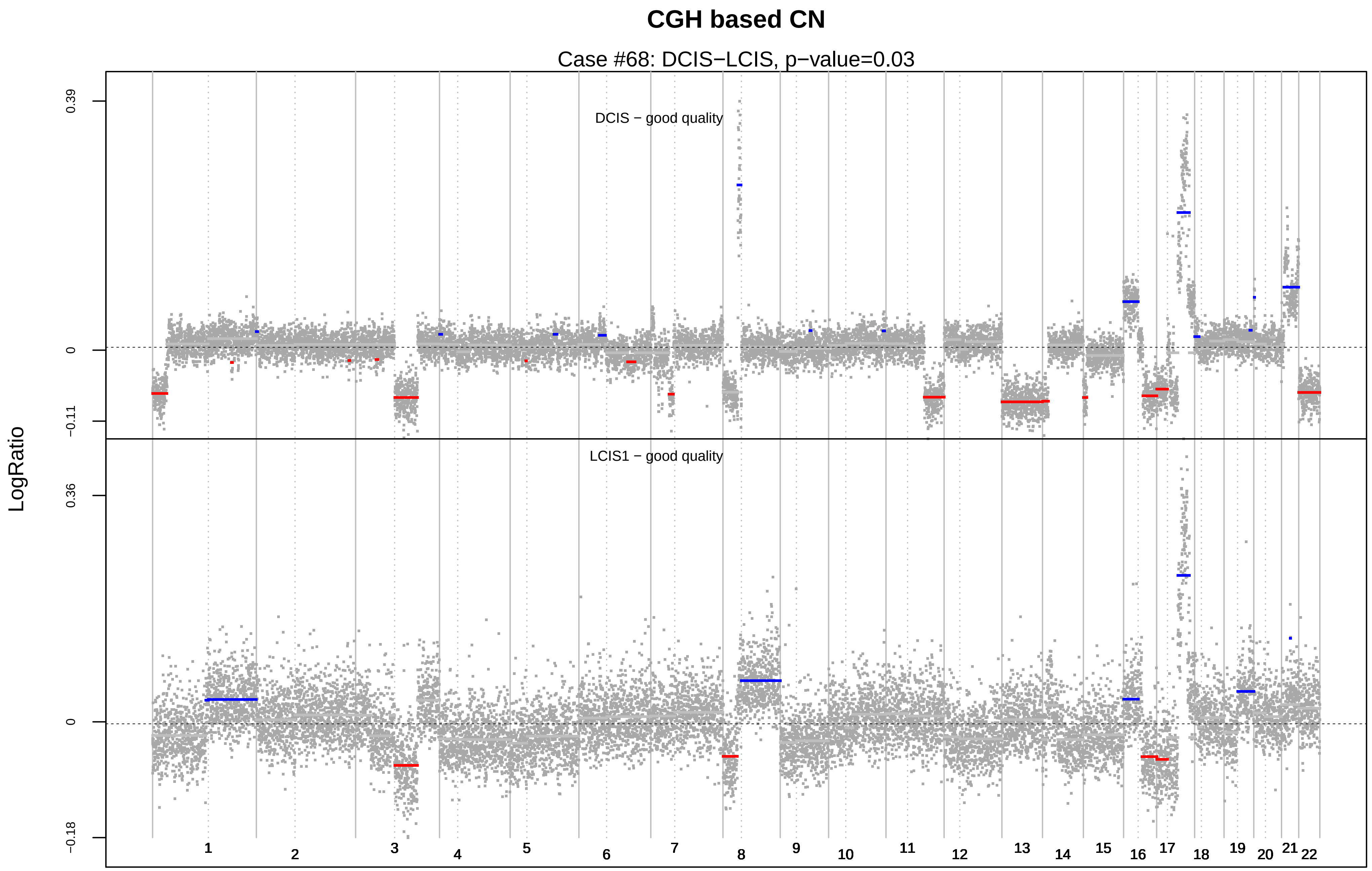
CGH based CN
Case #68: DCIS−LCIS, p−value=0.03
DCIS − good quality
LCIS1 − good quality
LogRatio
0.39
0
−0.11
0.36
0
−0.18
1
2
3
4
5
6
7
8
9
10
11
12
13
14
15
16
17
18
19
20
21
22

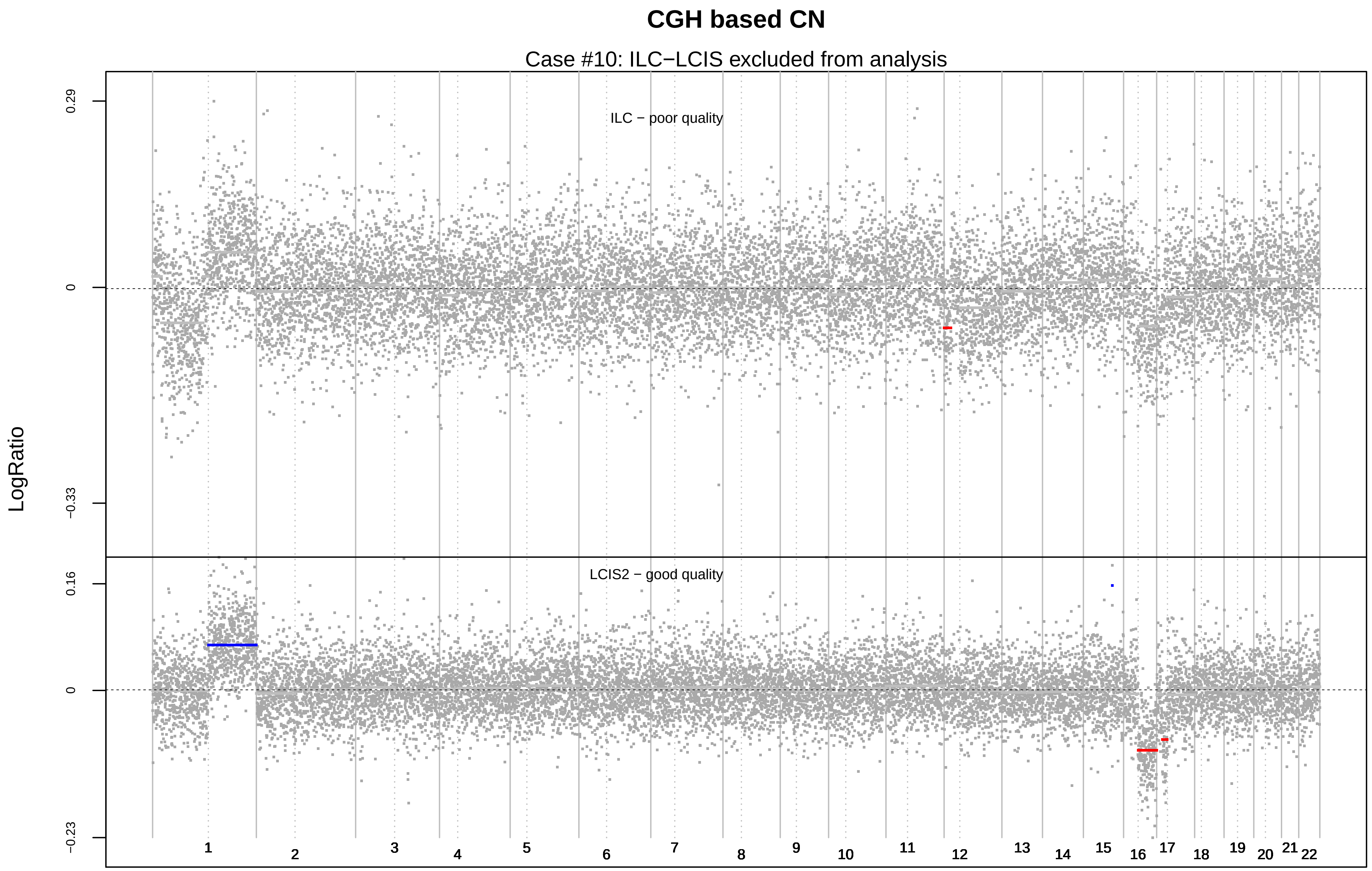

CGH based CN
Case #10: ILC−LCIS excluded from analysis
ILC − poor quality
LCIS2 − good quality
LogRatio
0.29
0
−0.33
0.16
0
−0.23
1
2
3
4
5
6
7
8
9
10
11
12
13
14
15
16
17
18
19
20
21
22

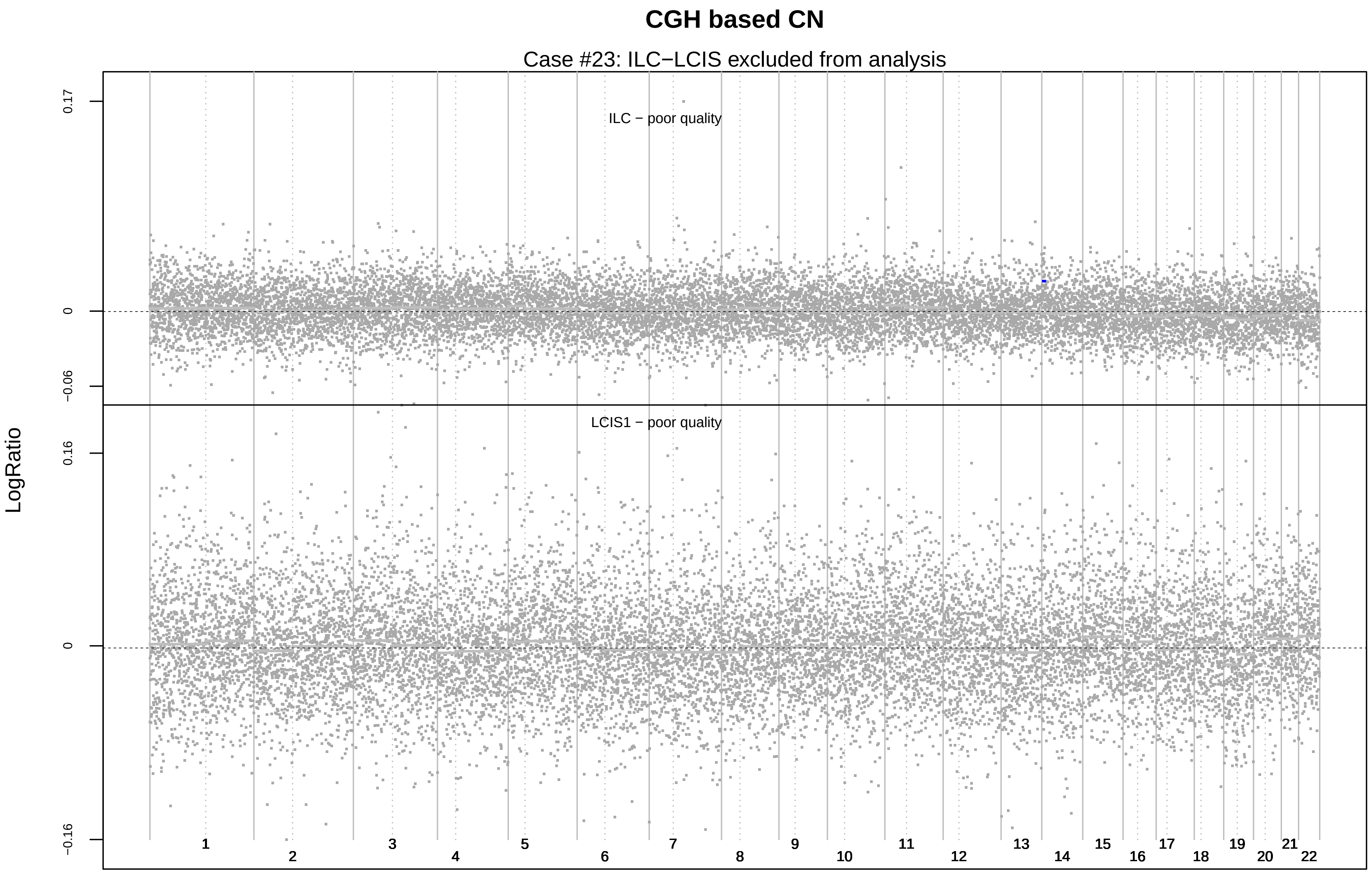

CGH based CN
Case #23: ILC−LCIS excluded from analysis
ILC − poor quality
LCIS1 − poor quality
LogRatio
0.17
0
−0.06
0.16
0
−0.16
1 2 3 4 5 6 7 8 9 10 11 12 13 14 15 16 17 18 19 20 21 22

CGH based CN

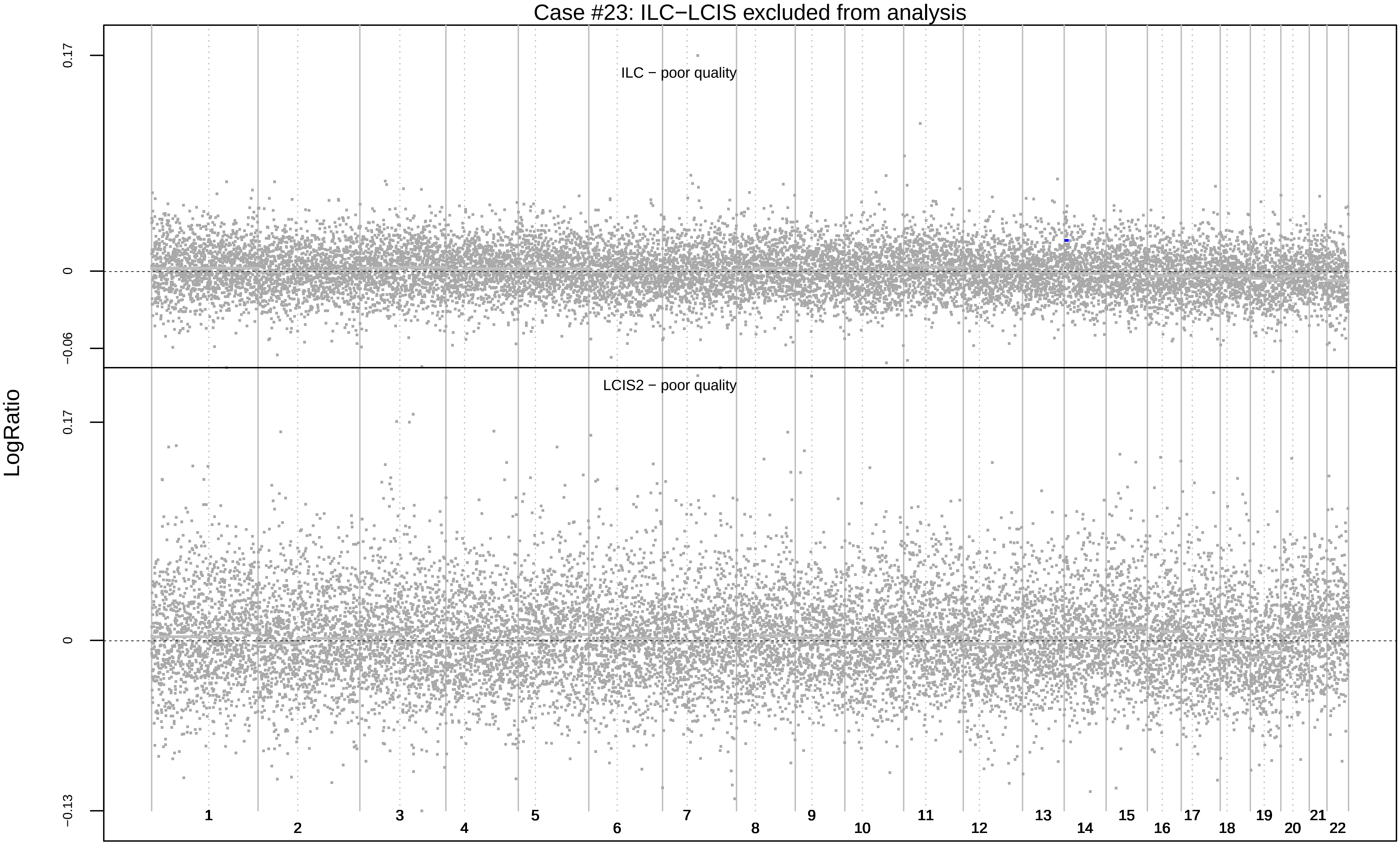

Case #23: ILC−LCIS excluded from analysis
ILC − poor quality
LCIS2 − poor quality
LogRatio
0.17
0
−0.06
0.17
0
−0.13
1
2
3
4
5
6
7
8
9
10
11
12
13
14
15
16
17
18
19
20
21
22

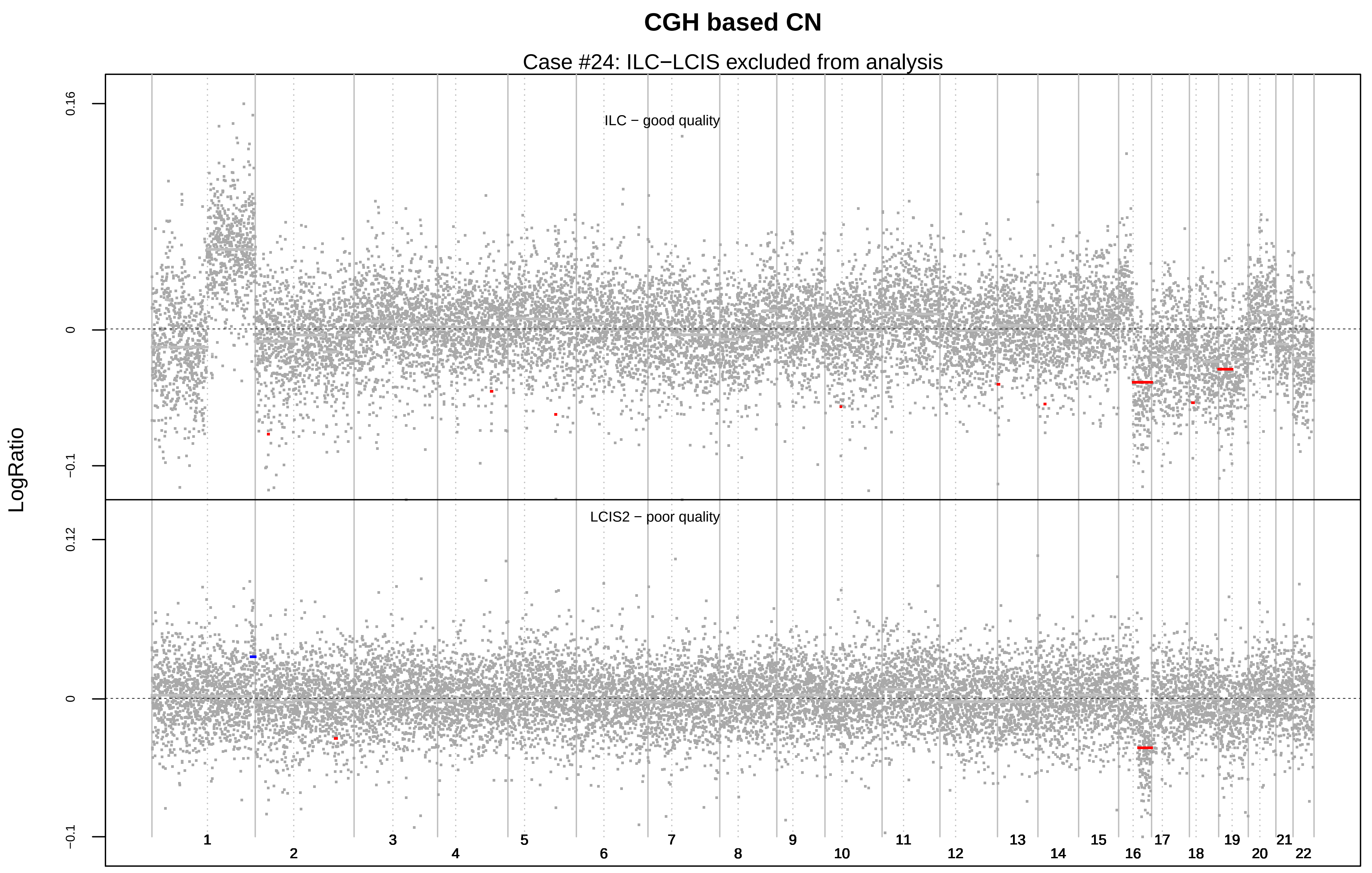
CGH based CN
Case #24: ILC−LCIS excluded from analysis
ILC − good quality
LCIS2 − poor quality
LogRatio
0.16
0
−0.1
0.12
0
−0.1
1
2
3
4
5
6
7
8
9
10
11
12
13
14
15
16
17
18
19
20
21
22

CGH based CN

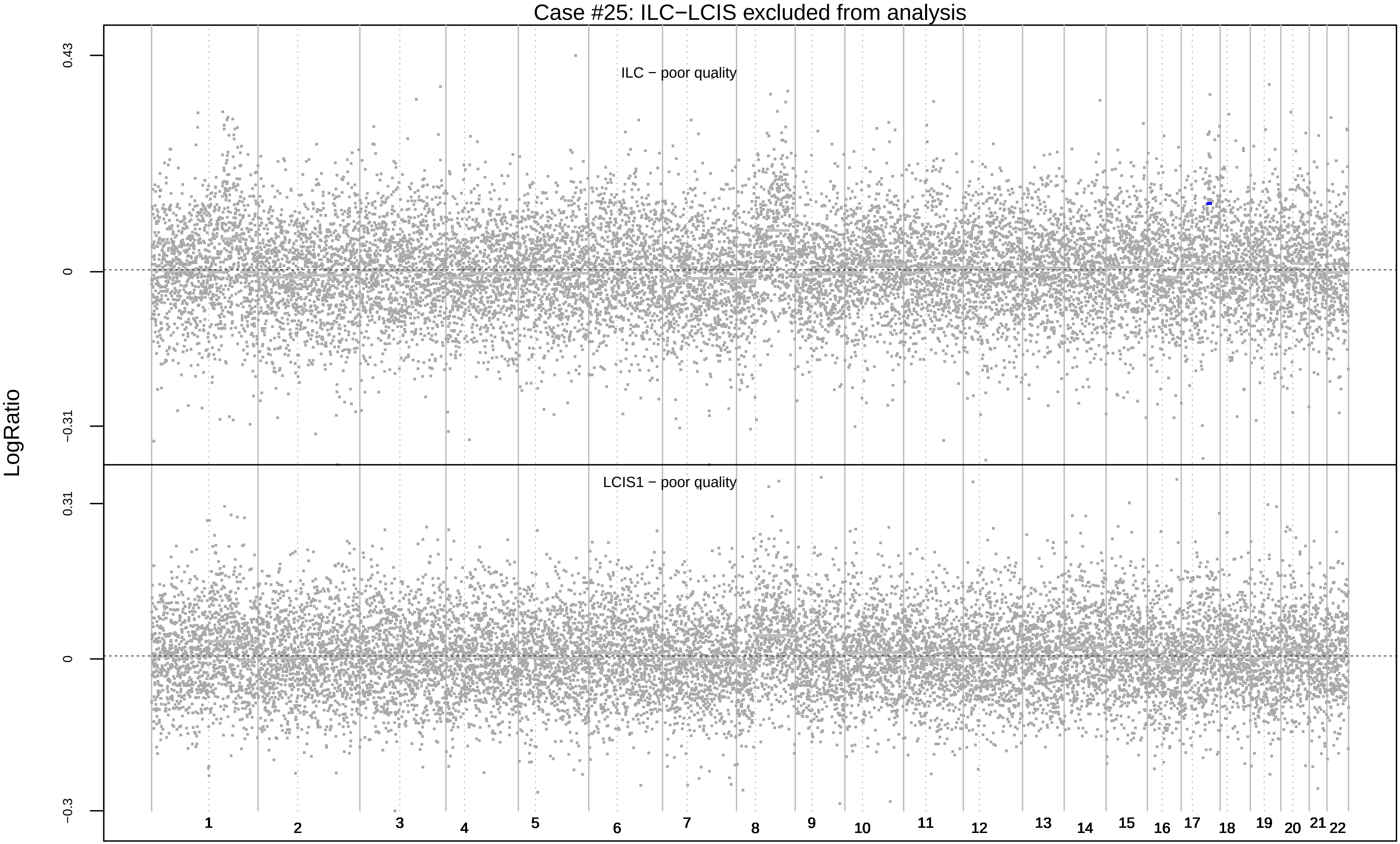

Case #25: ILC−LCIS excluded from analysis
ILC − poor quality
LCIS1 − poor quality
LogRatio
0.43
0
−0.31
0.31
0
−0.3
1
2
3
4
5
6
7
8
9
10
11
12
13
14
15
16
17
18
19
20
21
22

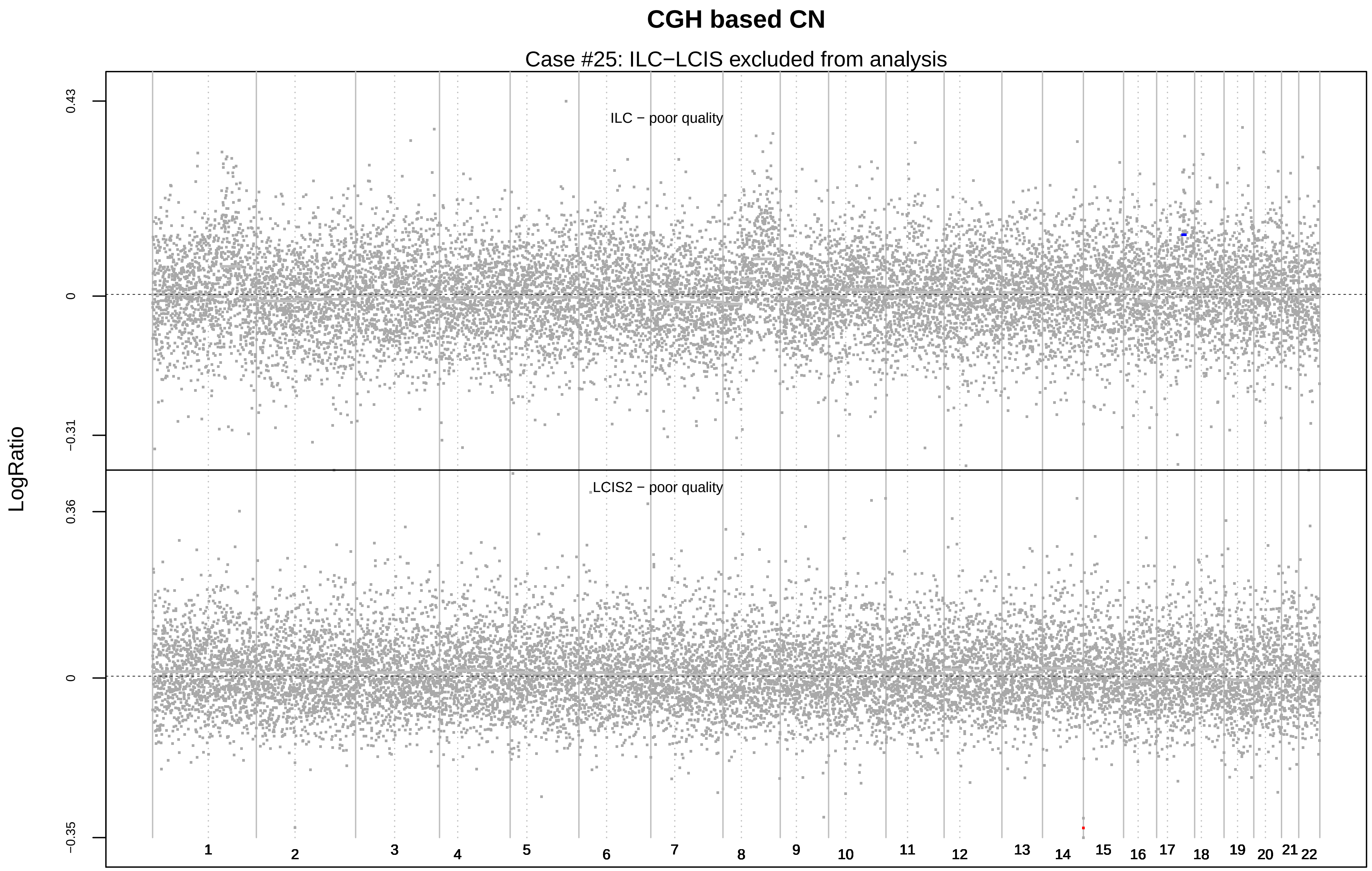
CGH based CN
Case #25: ILC−LCIS excluded from analysis
ILC − poor quality
LCIS2 − poor quality
LogRatio
0.43
0
−0.31
0.36
0
−0.35
1
2
3
4
5
6
7
8
9
10
11
12
13
14
15
16
17
18
19
20
21
22

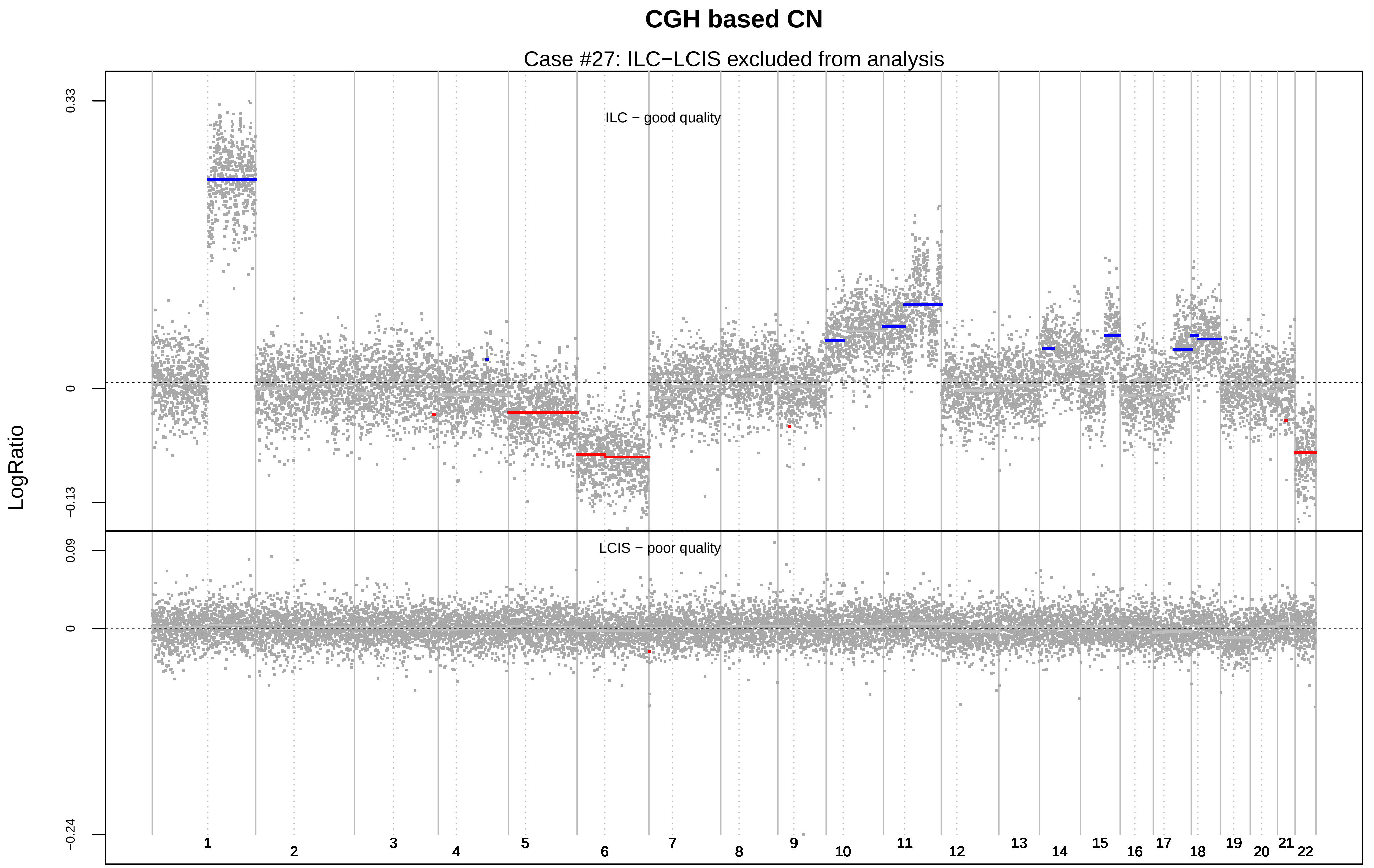

CGH based CN
Case #27: ILC−LCIS excluded from analysis
ILC − good quality
LCIS − poor quality
LogRatio
0.33
0
−0.13
0.09
0
−0.24
1
2
3
4
5
6
7
8
9
10
11
12
13
14
15
16
17
18
19
20
21
22

CGH based CN

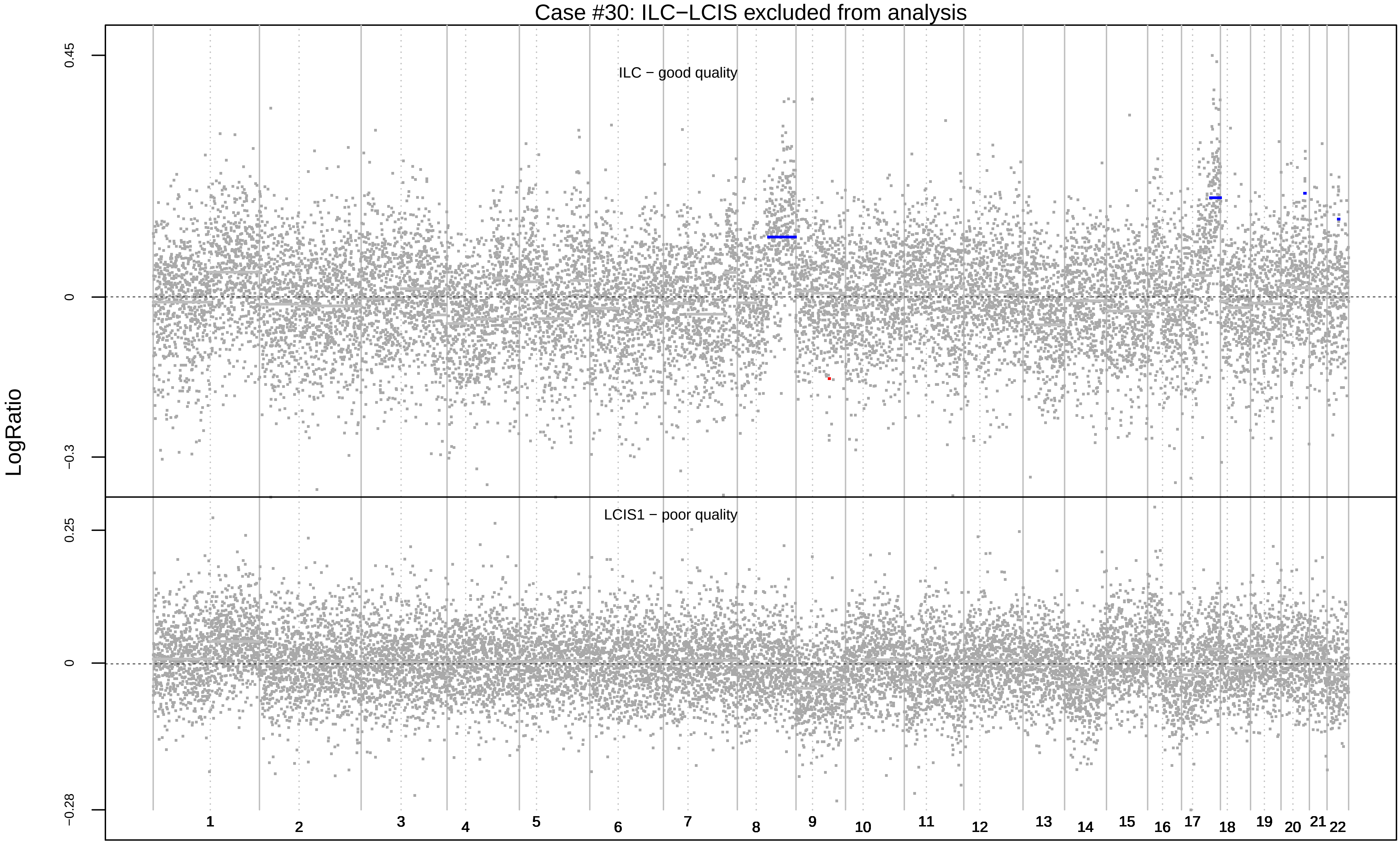
Case #30: ILC−LCIS excluded from analysis
LogRatio
ILC − good quality
0.45
0
−0.3
LCIS1 − poor quality
0.25
0
−0.28
1
2
3
4
5
6
7
8
9
10
11
12
13
14
15
16
17
18
19
20
21
22

CGH based CN

Case #30: ILC−LCIS excluded from analysis

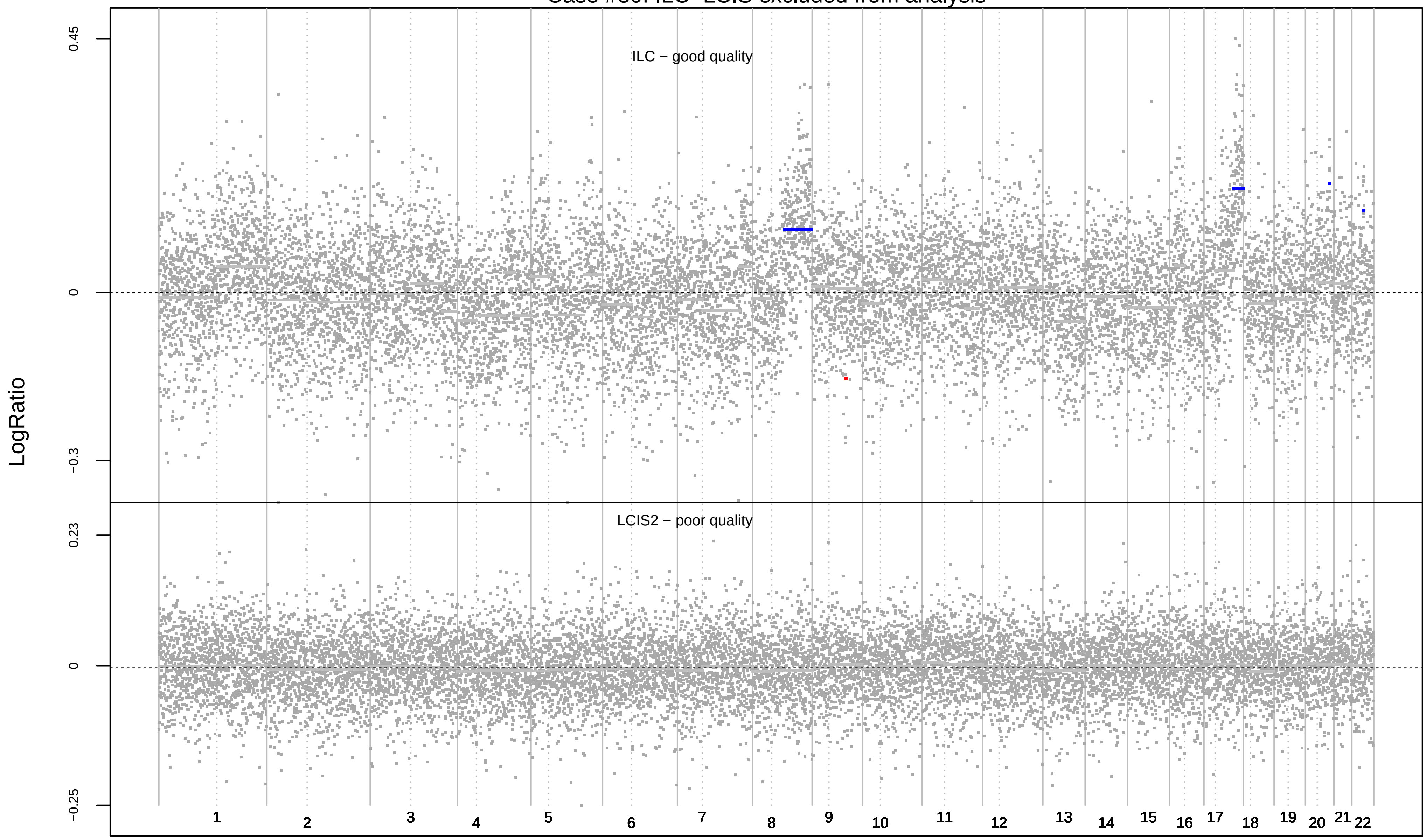

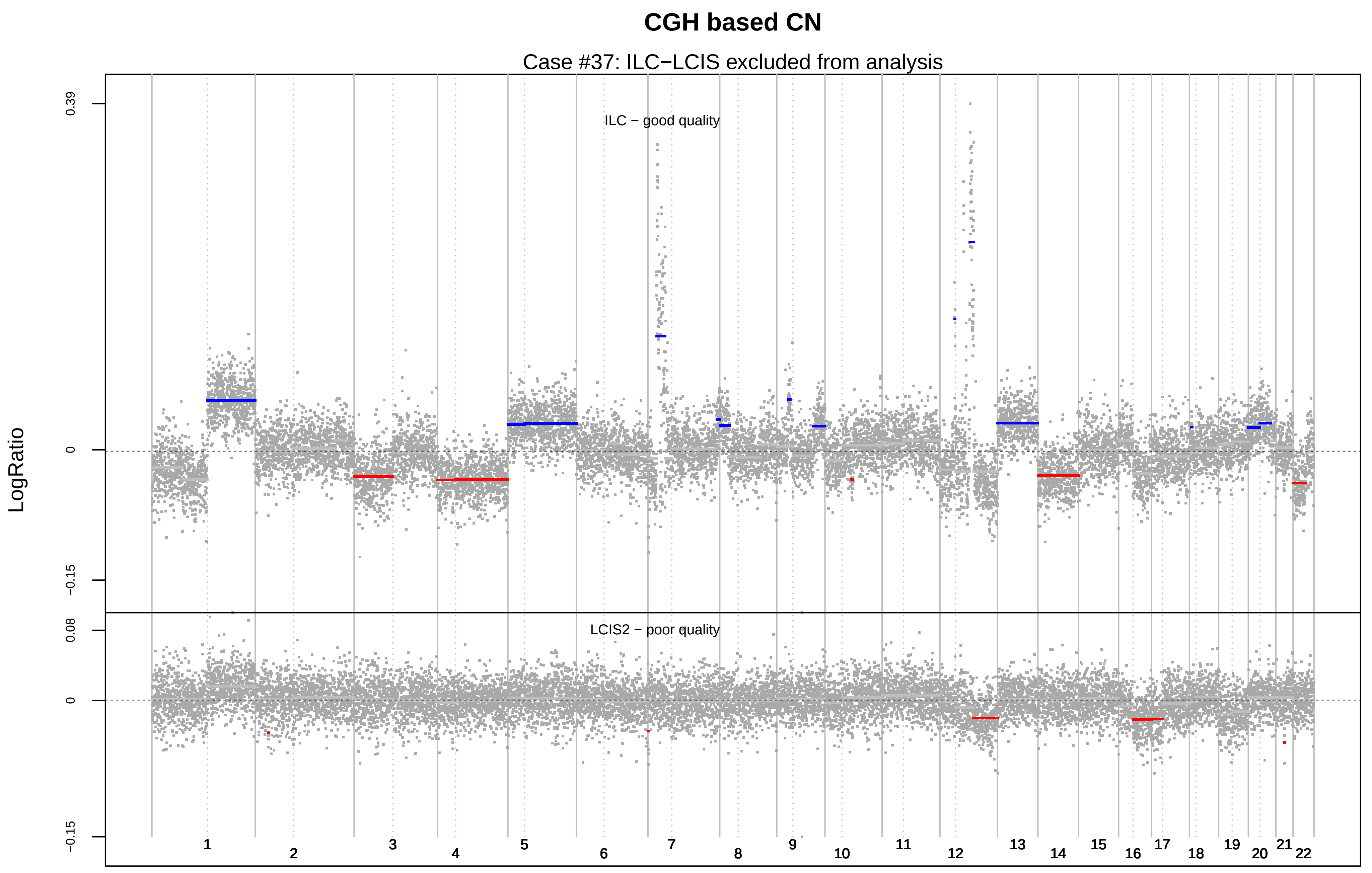

CGH based CN
Case #37: ILC−LCIS excluded from analysis
ILC − good quality
LCIS2 − poor quality
LogRatio
0.39
0
−0.15
0.08
0
−0.15
1
2
3
4
5
6
7
8
9
10
11
12
13
14
15
16
17
18
19
20
21
22

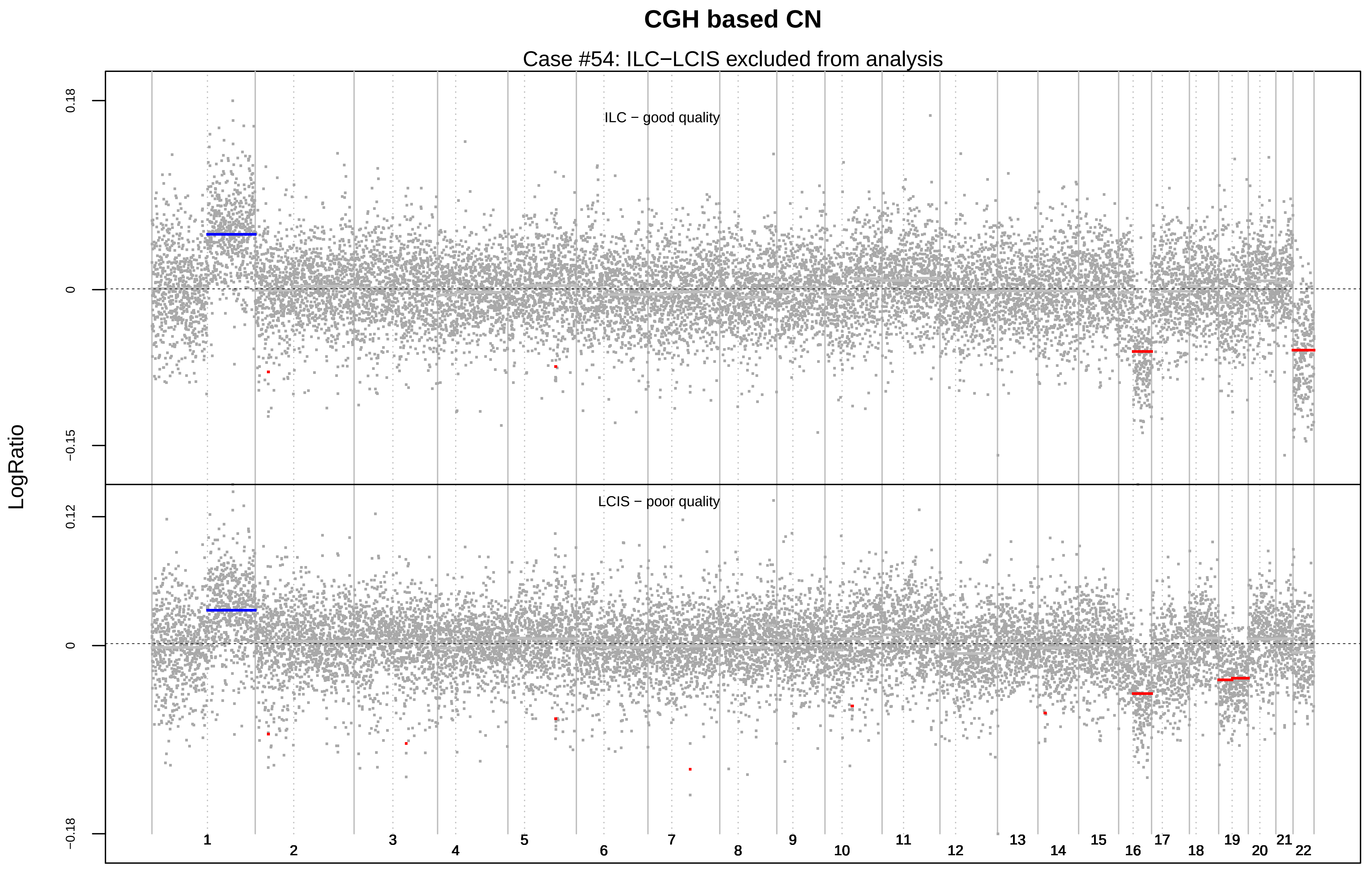

CGH based CN
Case #54: ILC−LCIS excluded from analysis
ILC − good quality
LCIS − poor quality
LogRatio
0.18
0
−0.15
0.12
0
−0.18
1
2
3
4
5
6
7
8
9
10
11
12
13
14
15
16
17
18
19
20
21
22

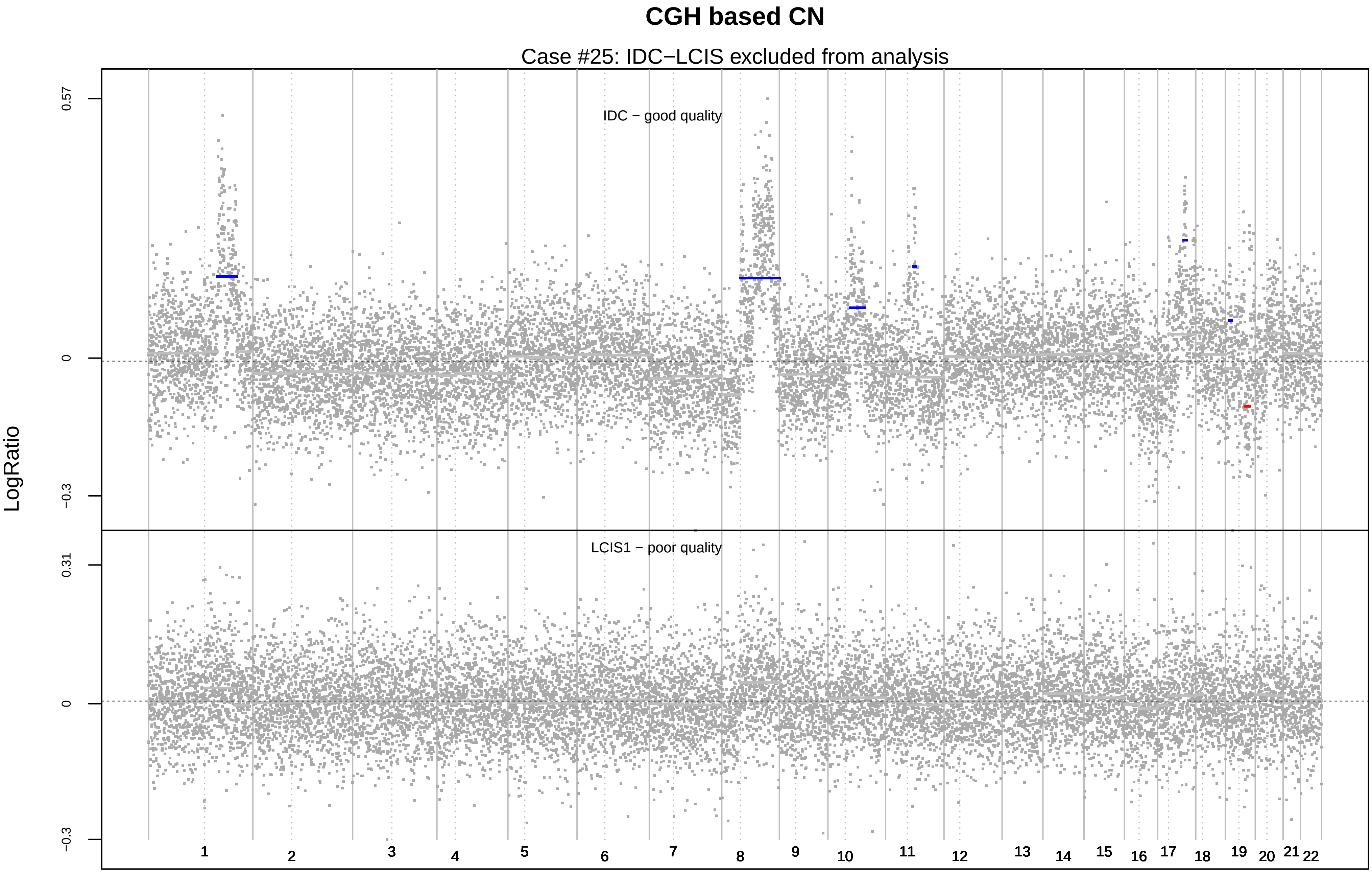
CGH based CN
Case #25: IDC−LCIS excluded from analysis
IDC − good quality
LCIS1 − poor quality
LogRatio
0.57
0
−0.3
0.31
0
−0.3
1
2
3
4
5
6
7
8
9
10
11
12
13
14
15
16
17
18
19
20
21
22

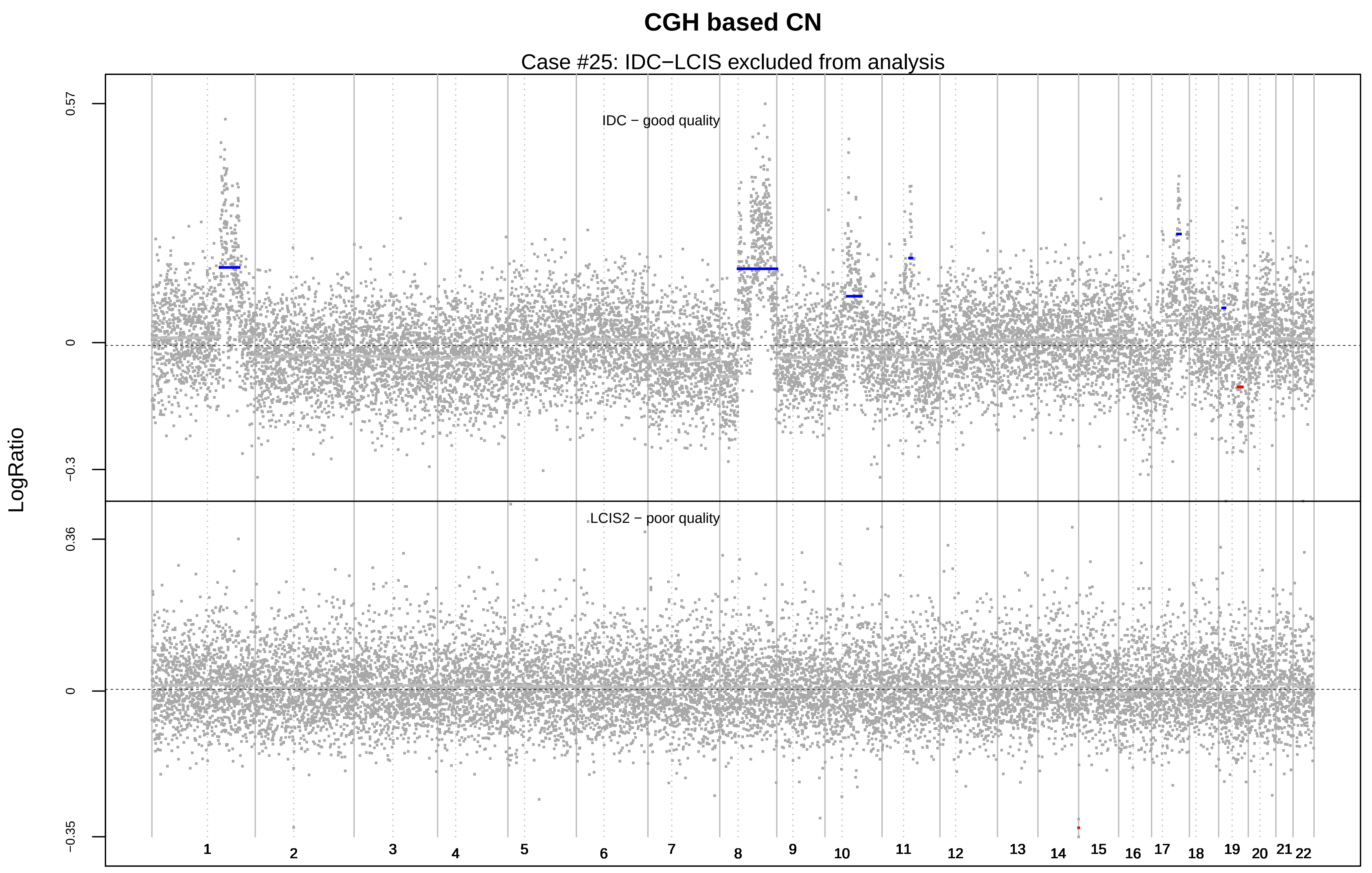

CGH based CN
Case #25: IDC−LCIS excluded from analysis
IDC − good quality
LCIS2 − poor quality
LogRatio
0.57
0
−0.3
0.36
0
−0.35
1
2
3
4
5
6
7
8
9
10
11
12
13
14
15
16
17
18
19
20
21
22

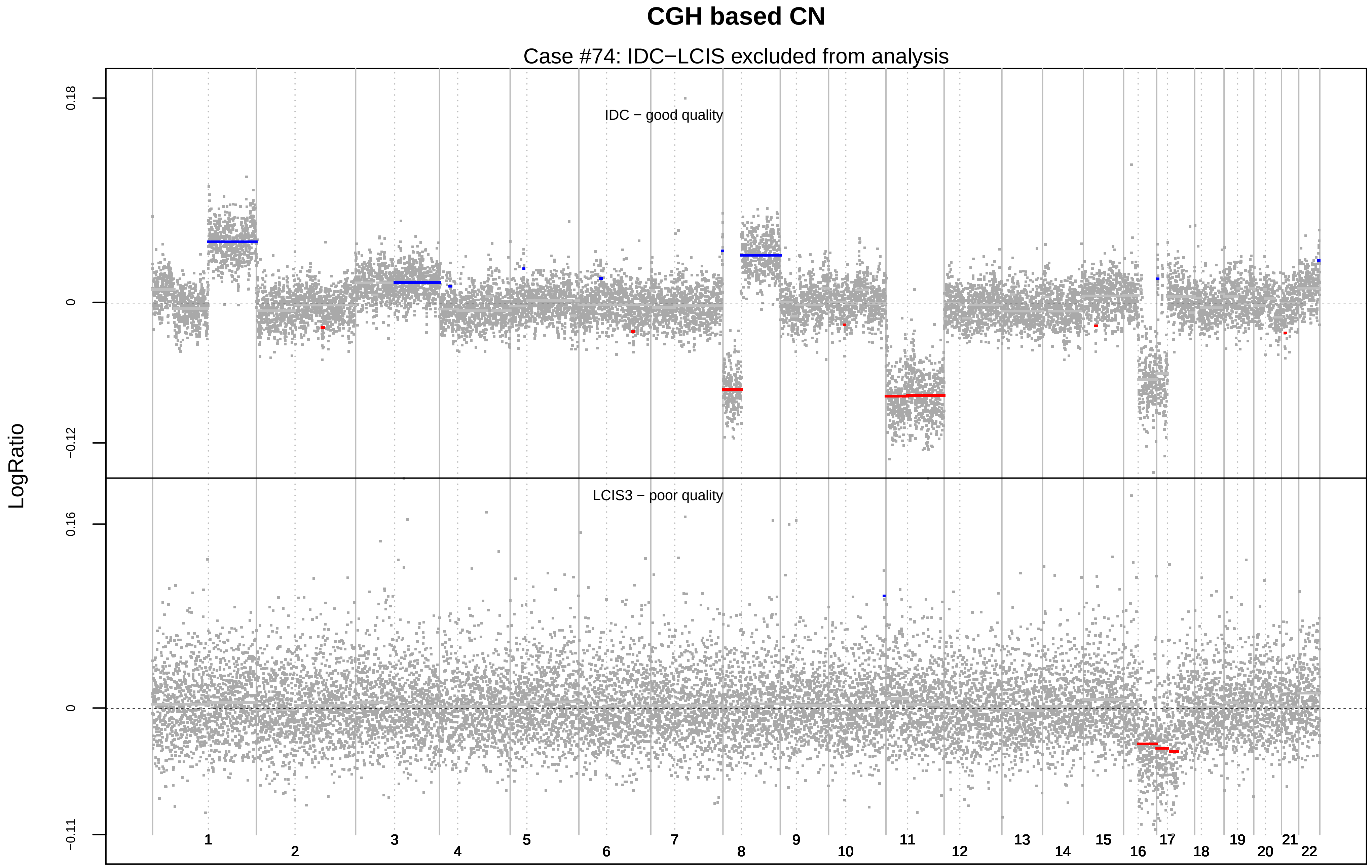
CGH based CN
Case #74: IDC−LCIS excluded from analysis
IDC − good quality
LCIS3 − poor quality
LogRatio
0.18
0
−0.12
0.16
0
−0.11
1
2
3
4
5
6
7
8
9
10
11
12
13
14
15
16
17
18
19
20
21
22

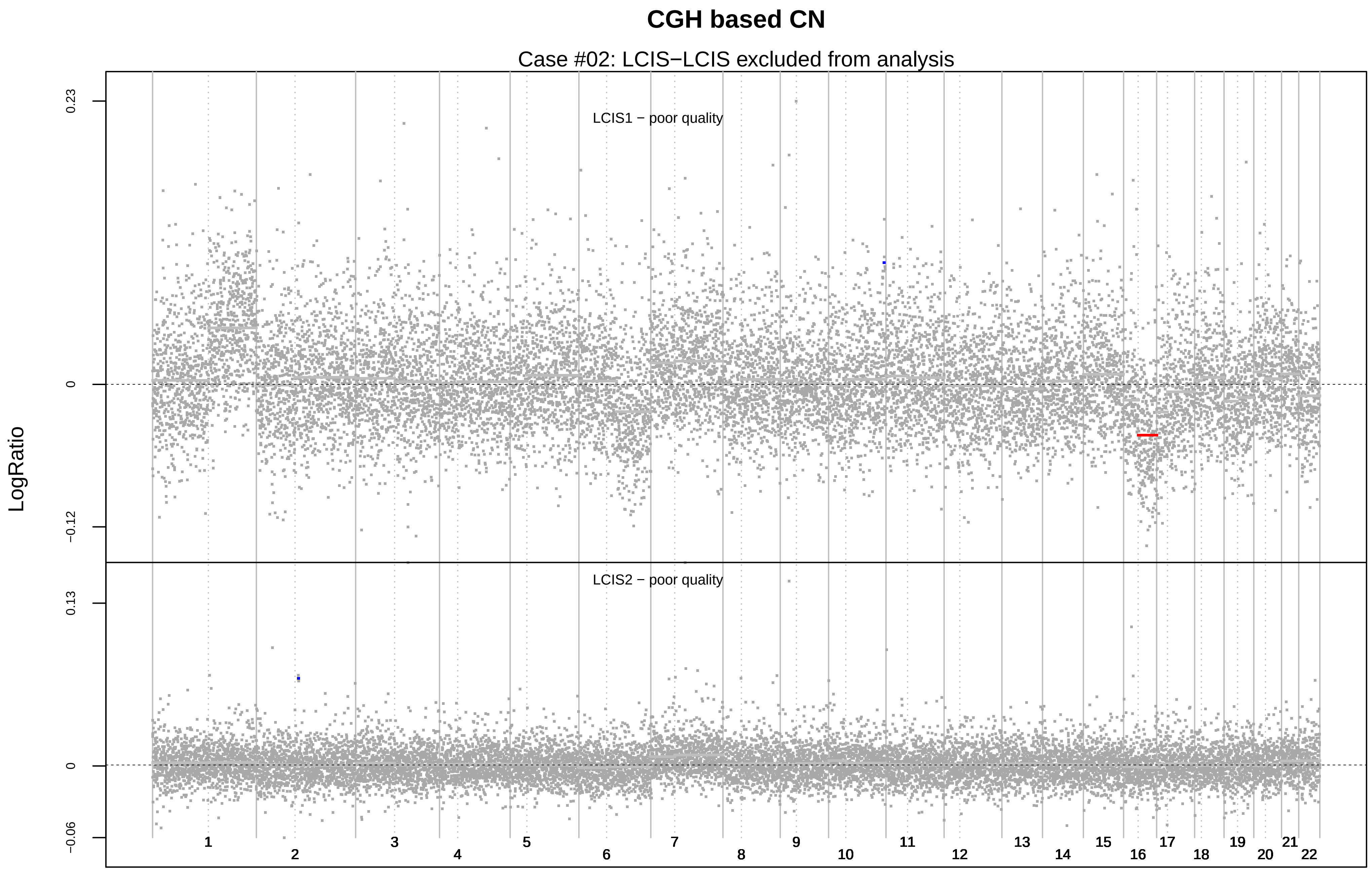

CGH based CN
Case #02: LCIS−LCIS excluded from analysis
LCIS1 − poor quality
LCIS2 − poor quality
LogRatio
0.23
0
−0.12
0.13
0
−0.06
1 2 3 4 5 6 7 8 9 10 11 12 13 14 15 16 17 18 19 20 21 22

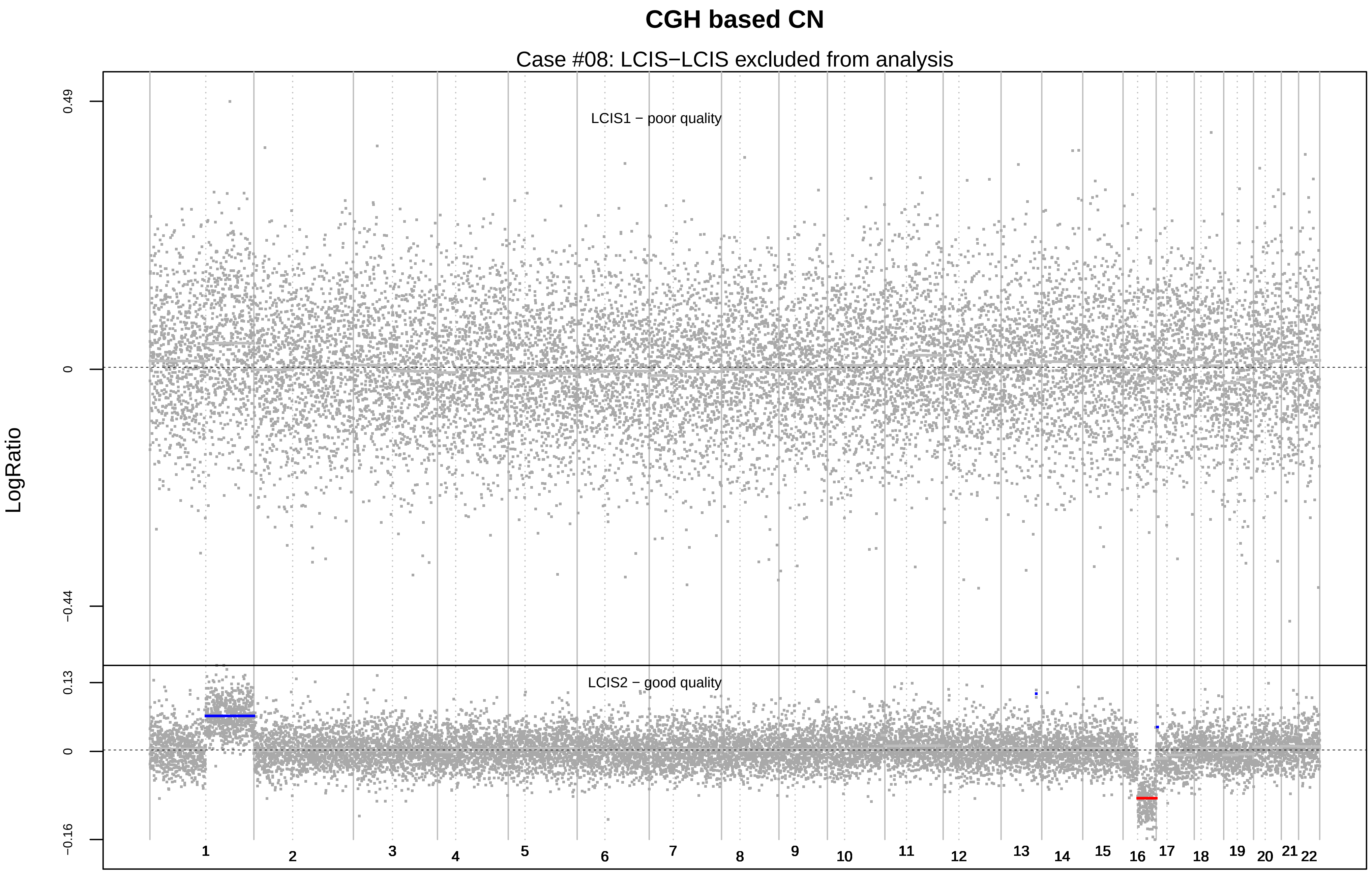

CGH based CN
Case #08: LCIS−LCIS excluded from analysis
LogRatio
LCIS1 − poor quality
LCIS2 − good quality
0.49
0
−0.44
0.13
0
−0.16
1
2
3
4
5
6
7
8
9
10
11
12
13
14
15
16
17
18
19
20
21
22

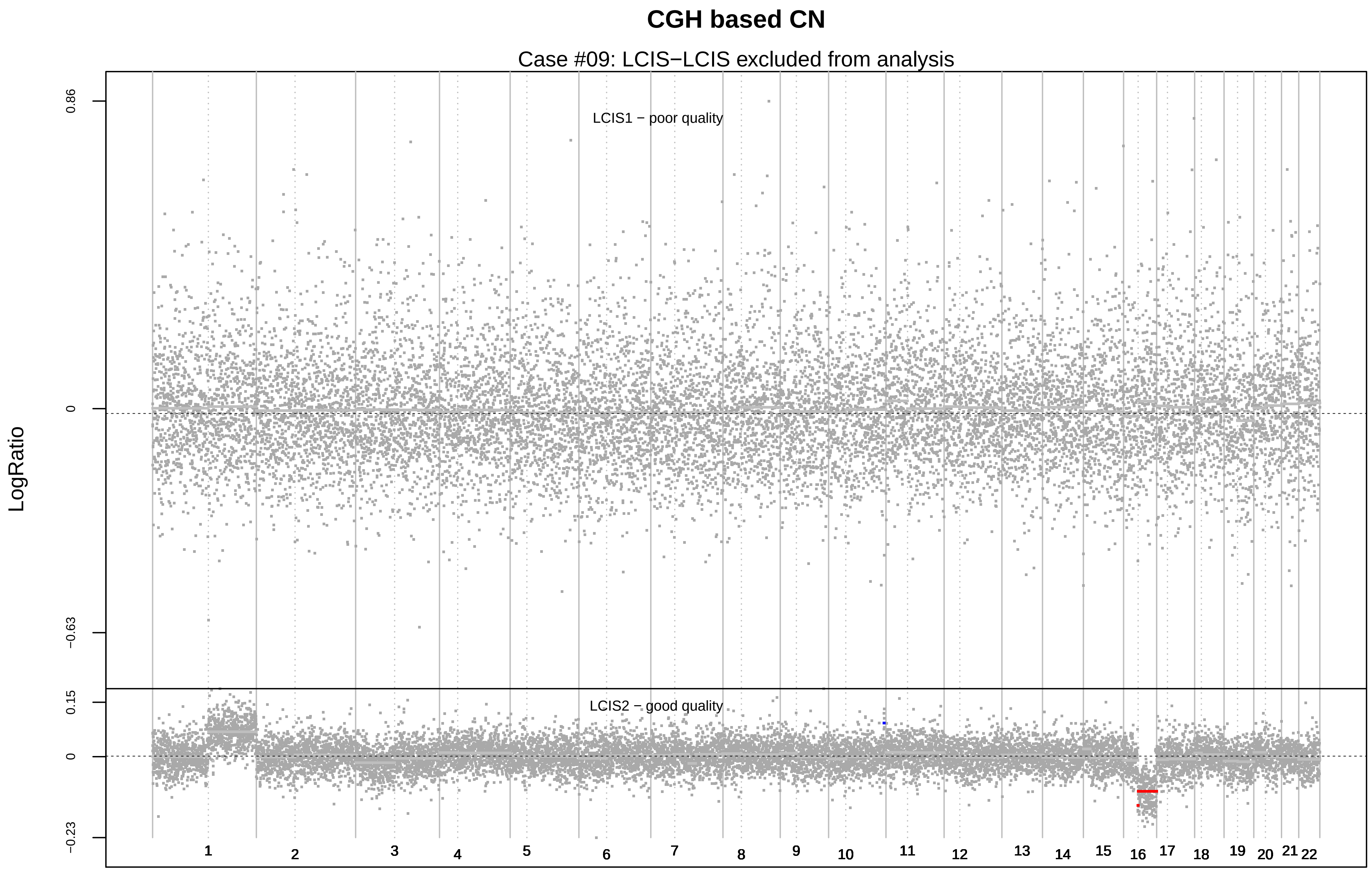

CGH based CN
Case #09: LCIS−LCIS excluded from analysis
LCIS1 − poor quality
LCIS2 − good quality
LogRatio
0.86
0
−0.63
0.15
0
−0.23
1
2
3
4
5
6
7
8
9
10
11
12
13
14
15
16
17
18
19
20
21
22

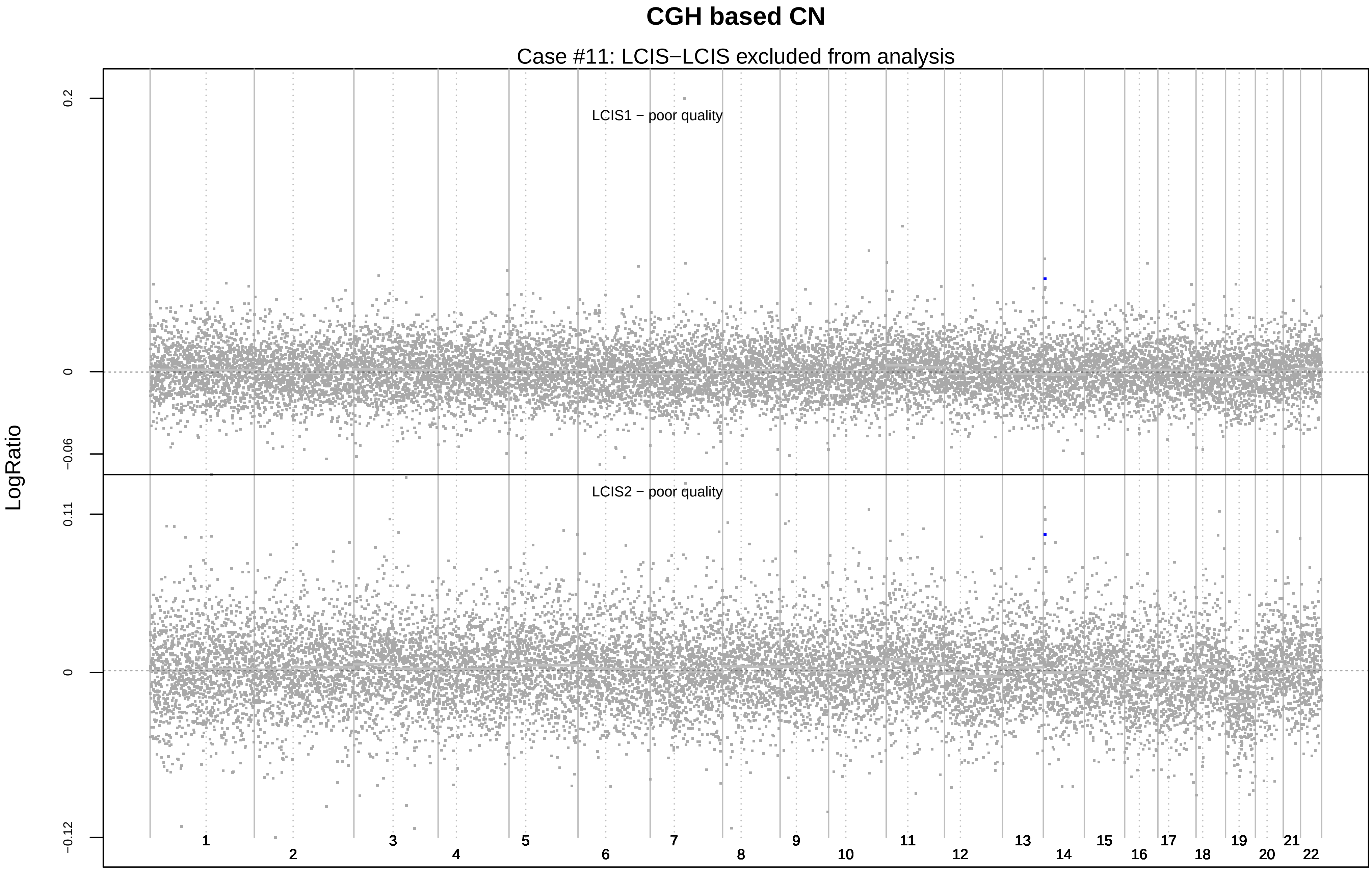
CGH based CN
Case #11: LCIS−LCIS excluded from analysis
LCIS1 − poor quality
LCIS2 − poor quality
LogRatio
0.2
0
−0.06
0.11
0
−0.12
1
2
3
4
5
6
7
8
9
10
11
12
13
14
15
16
17
18
19
20
21
22

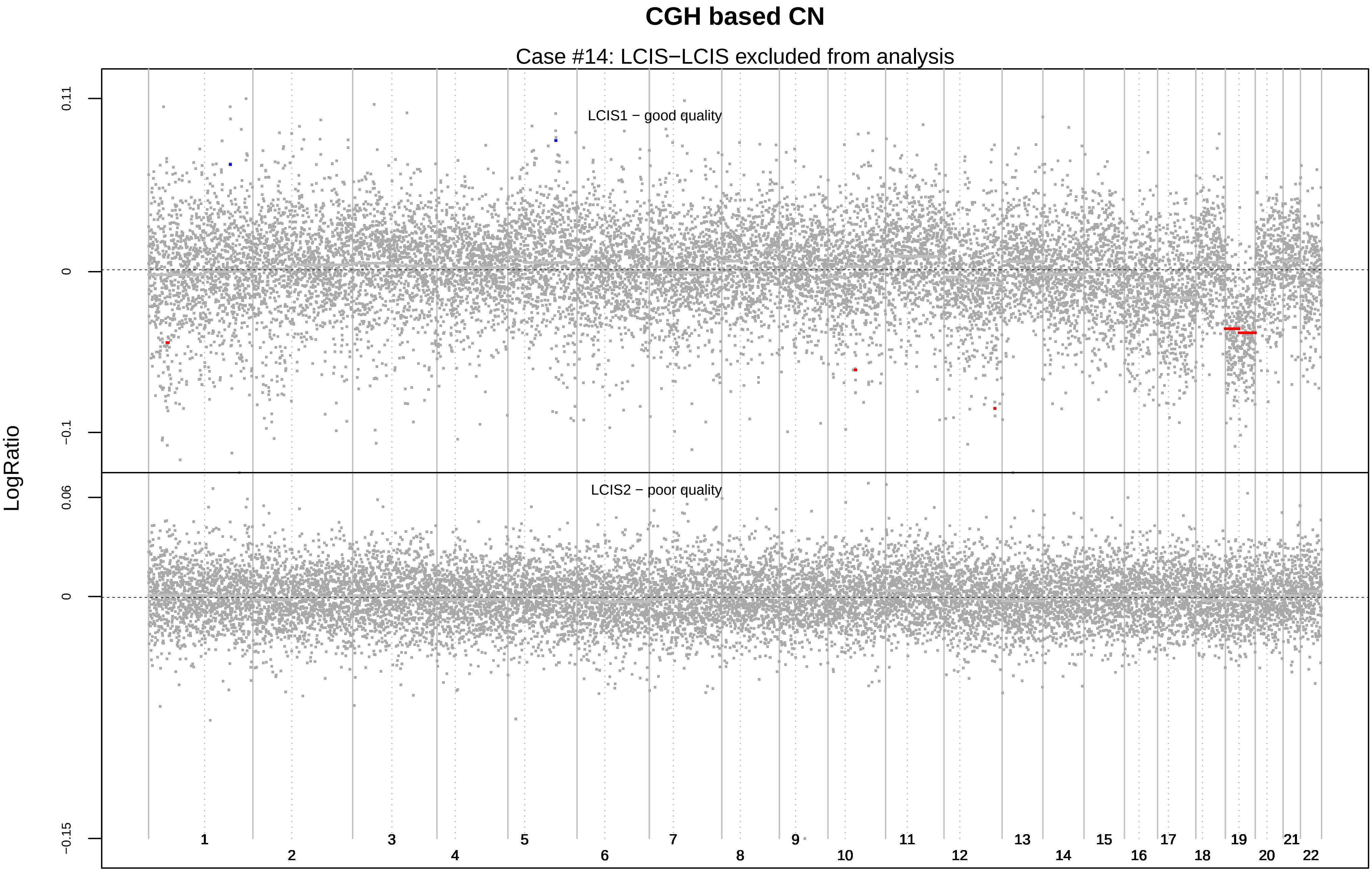

CGH based CN
Case #14: LCIS−LCIS excluded from analysis
LogRatio
LCIS1 − good quality
LCIS2 − poor quality
0.11
0
−0.1
0.06
0
−0.15
1
2
3
4
5
6
7
8
9
10
11
12
13
14
15
16
17
18
19
20
21
22

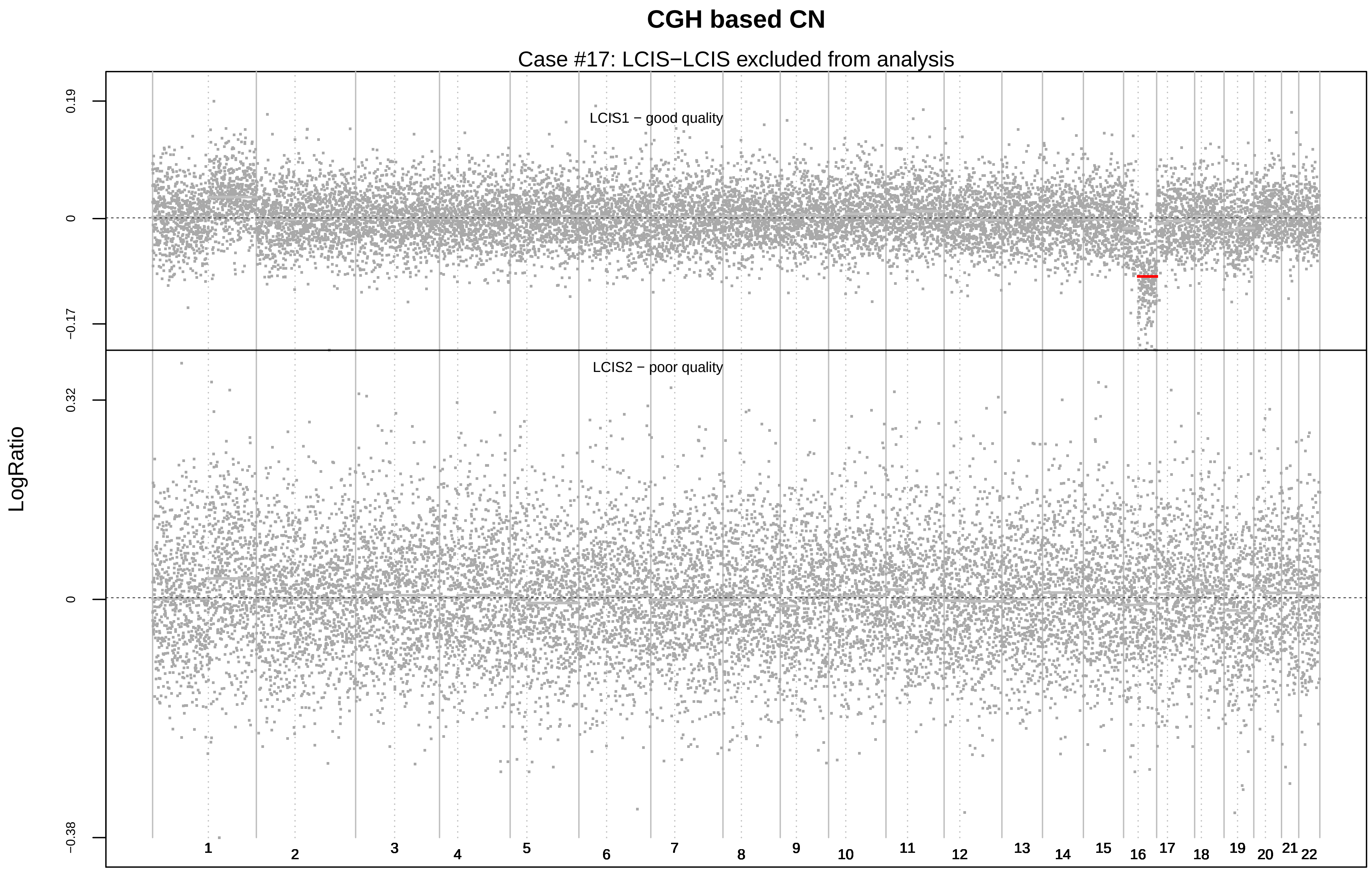

CGH based CN
Case #17: LCIS−LCIS excluded from analysis
LCIS1 − good quality
LCIS2 − poor quality
LogRatio
0.19
0
−0.17
0.32
0
−0.38
1
2
3
4
5
6
7
8
9
10
11
12
13
14
15
16
17
18
19
20
21
22

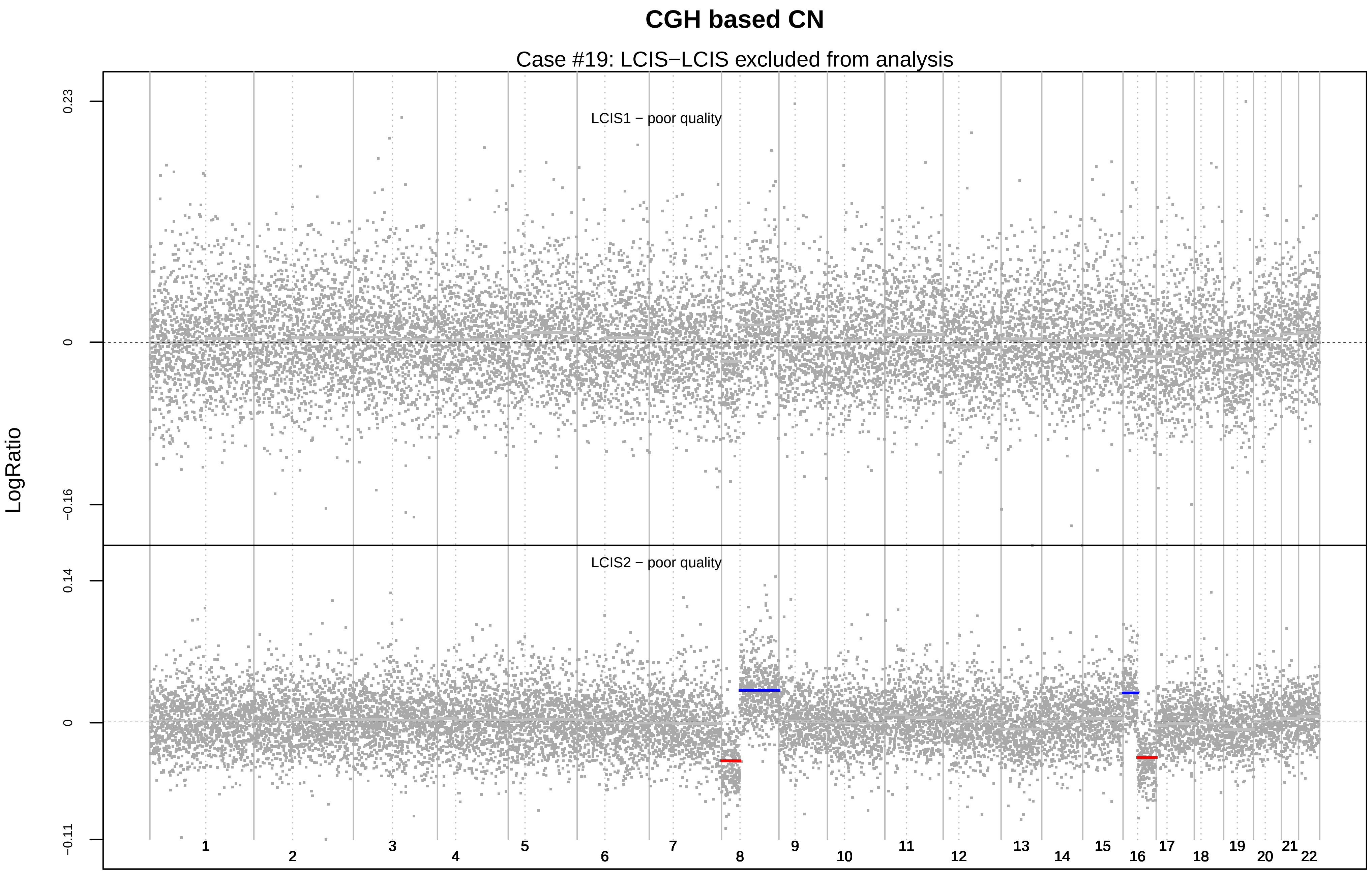

CGH based CN
Case #19: LCIS−LCIS excluded from analysis
LCIS1 − poor quality
LCIS2 − poor quality
LogRatio
0.23
0
−0.16
0.14
0
−0.11
1
2
3
4
5
6
7
8
9
10
11
12
13
14
15
16
17
18
19
20
21
22

CGH based CN

Case #23: LCIS−LCIS excluded from analysis

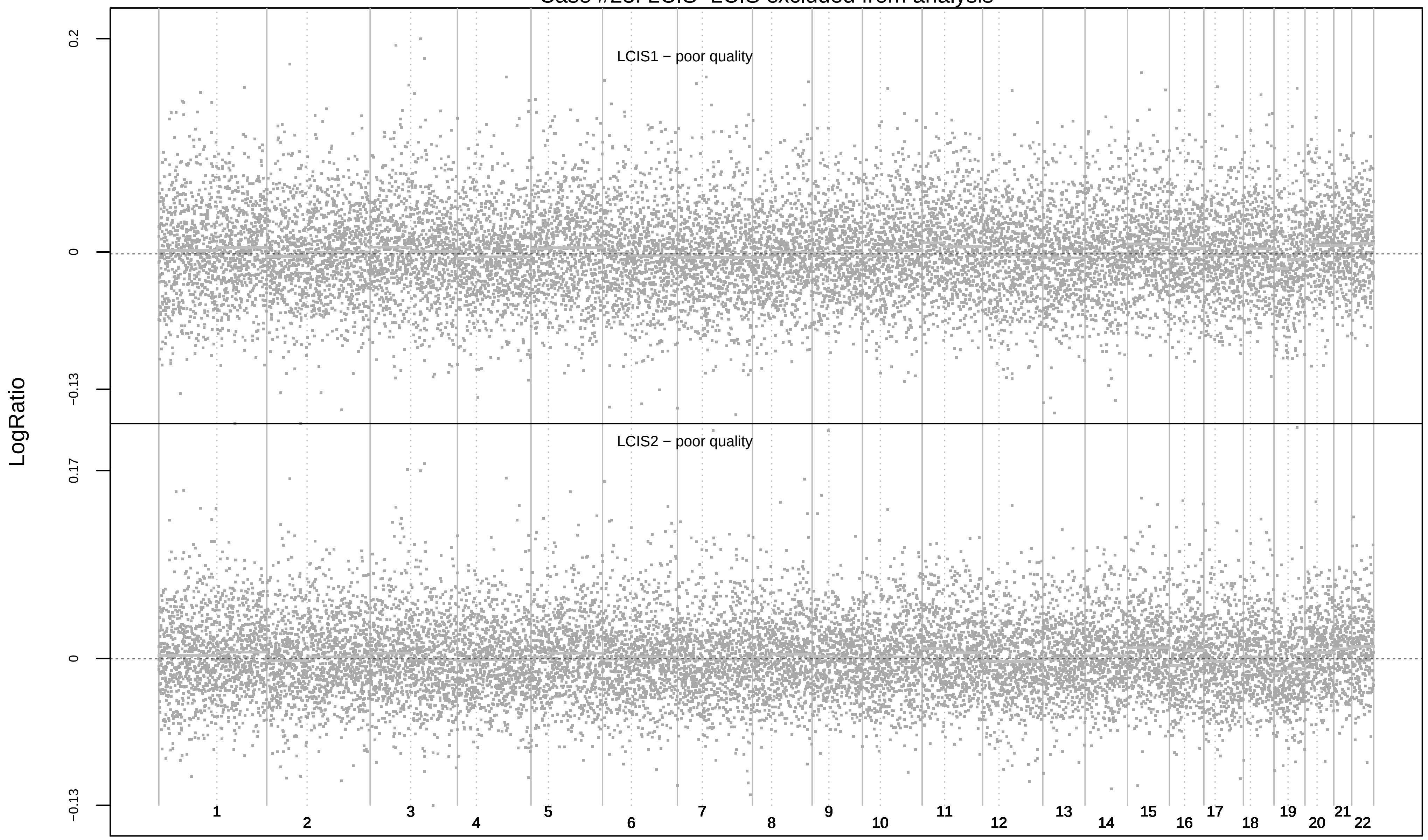

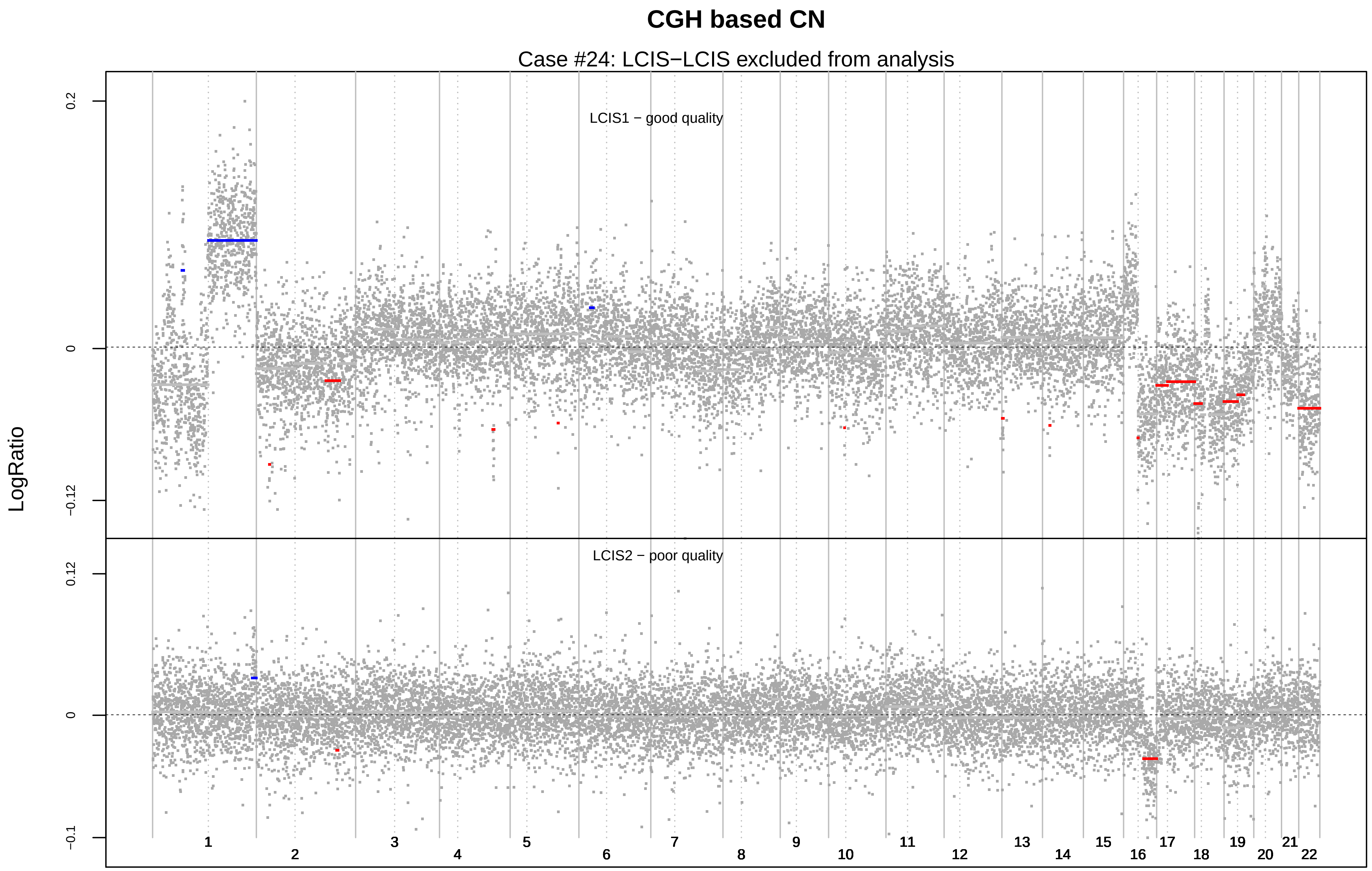
CGH based CN
Case #24: LCIS−LCIS excluded from analysis
LCIS1 − good quality
LCIS2 − poor quality
LogRatio
0.2
0
−0.12
0.12
0
−0.1
1
2
3
4
5
6
7
8
9
10
11
12
13
14
15
16
17
18
19
20
21
22

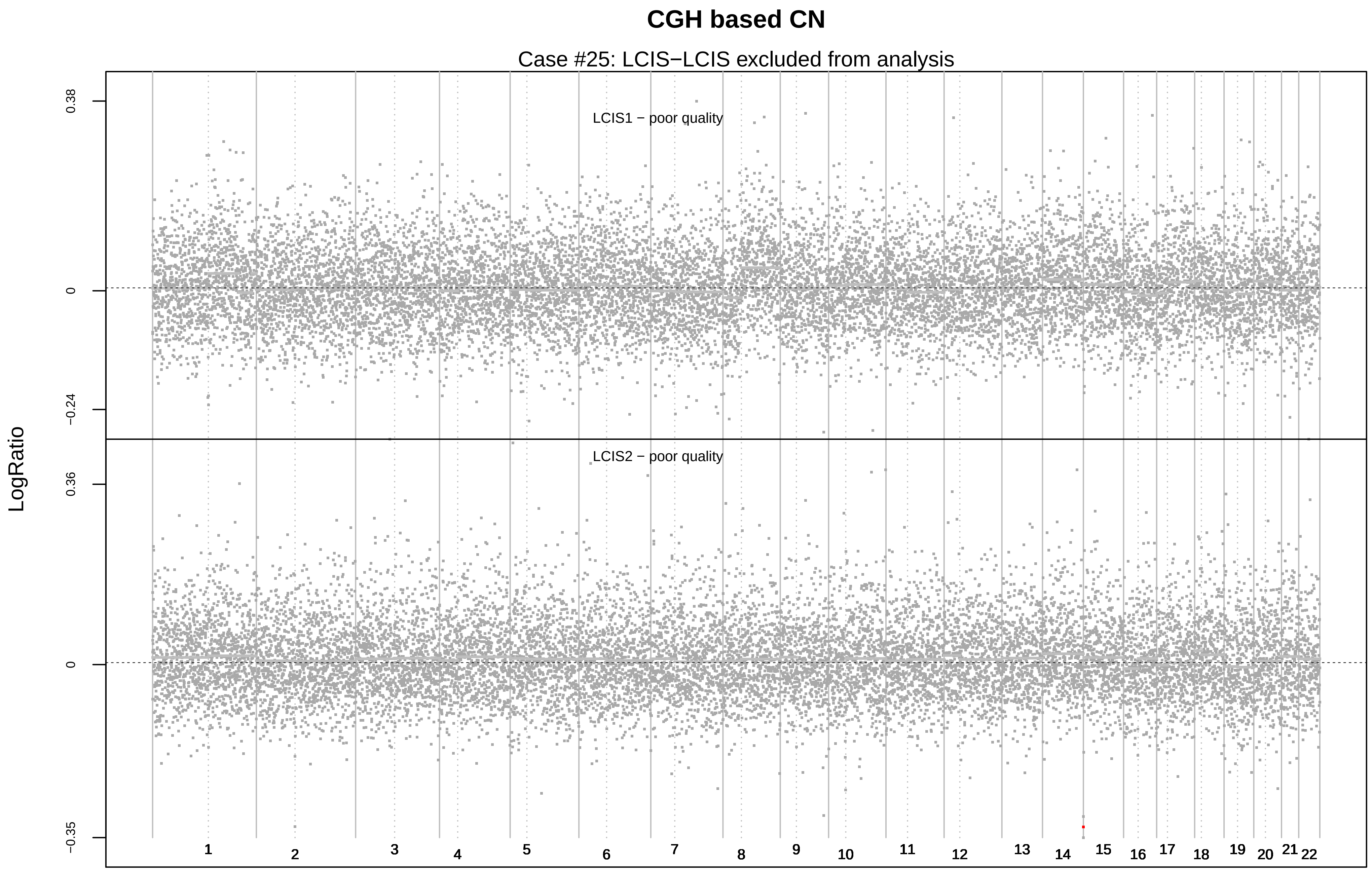
CGH based CN
Case #25: LCIS−LCIS excluded from analysis
LCIS1 − poor quality
LCIS2 − poor quality
LogRatio
0.38
0
−0.24
0.36
0
−0.35
1
2
3
4
5
6
7
8
9
10
11
12
13
14
15
16
17
18
19
20
21
22

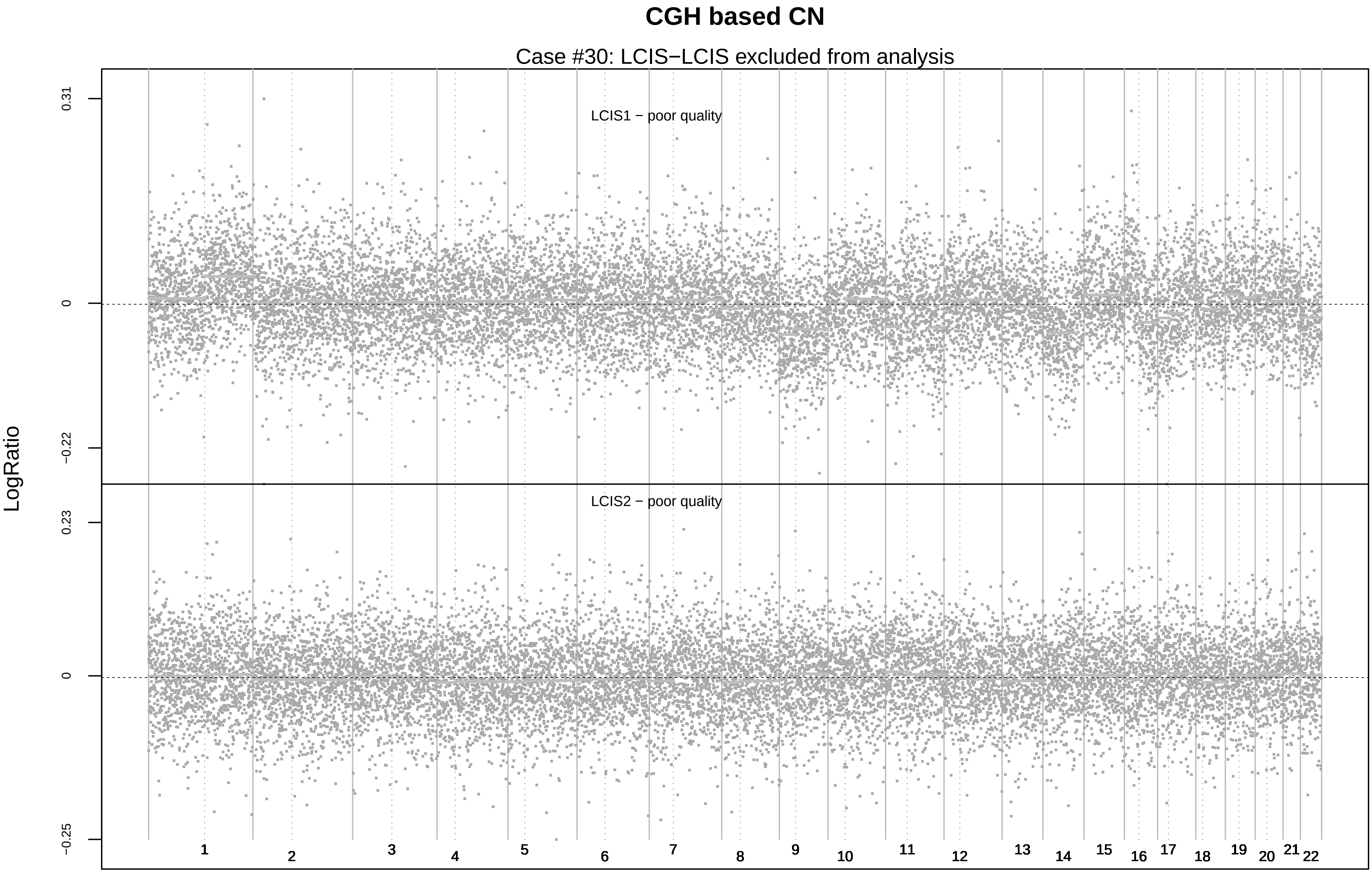
CGH based CN
Case #30: LCIS−LCIS excluded from analysis
LogRatio
LCIS1 − poor quality
0.31
0
−0.22
LCIS2 − poor quality
0.23
0
−0.25
1
2
3
4
5
6
7
8
9
10
11
12
13
14
15
16
17
18
19
20
21
22

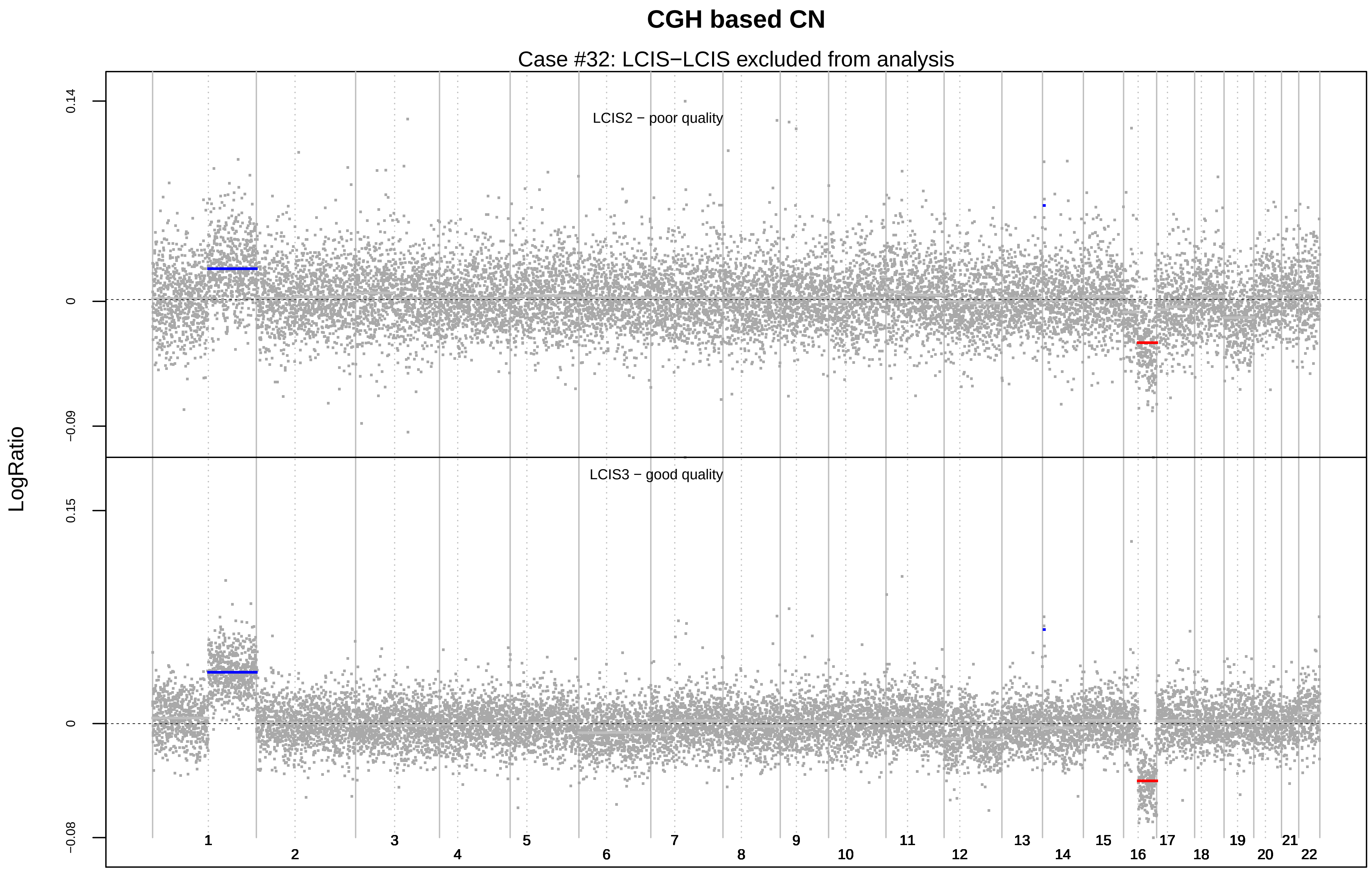

CGH based CN
Case #32: LCIS−LCIS excluded from analysis
LCIS2 − poor quality
LCIS3 − good quality
LogRatio
0.14
0
−0.09
0.15
0
−0.08
1
2
3
4
5
6
7
8
9
10
11
12
13
14
15
16
17
18
19
20
21
22

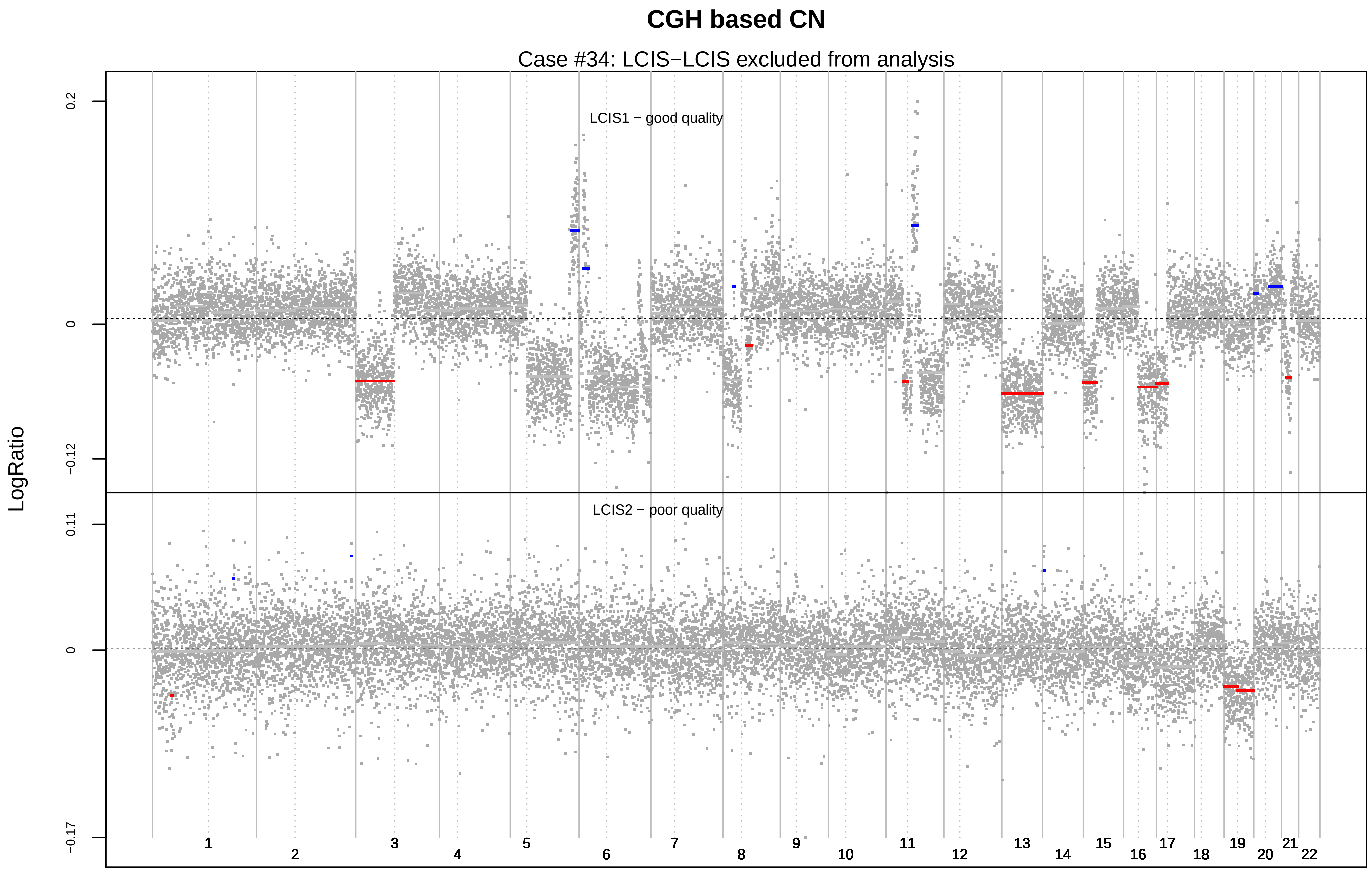

CGH based CN
Case #34: LCIS−LCIS excluded from analysis
LCIS1 − good quality
LCIS2 − poor quality
LogRatio
0.2
0
−0.12
0.11
0
−0.17
1
2
3
4
5
6
7
8
9
10
11
12
13
14
15
16
17
18
19
20
21
22

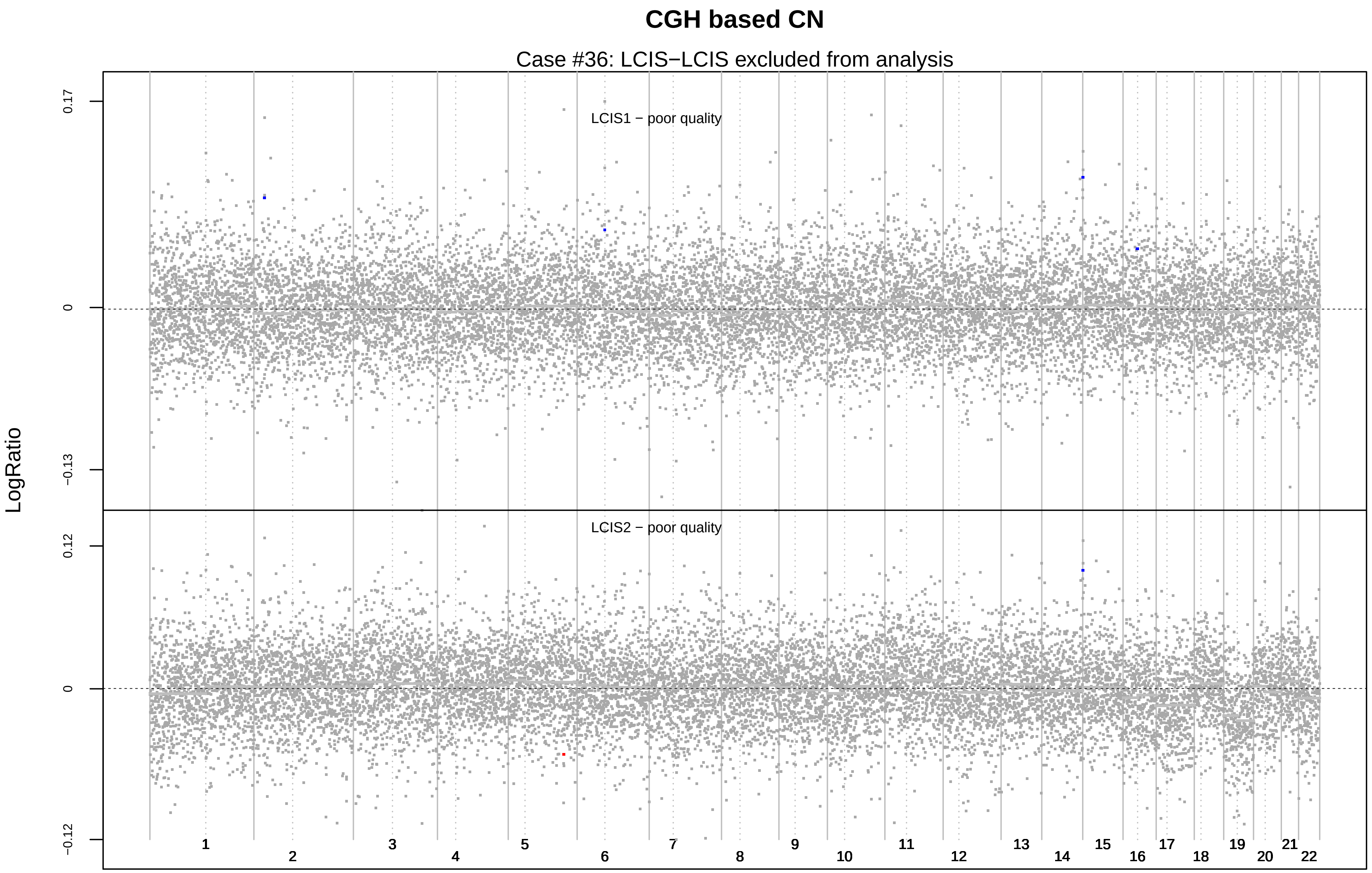

CGH based CN
Case #36: LCIS−LCIS excluded from analysis
LCIS1 − poor quality
LCIS2 − poor quality
LogRatio
0.17
0
−0.13
0.12
0
−0.12
1
2
3
4
5
6
7
8
9
10
11
12
13
14
15
16
17
18
19
20
21
22

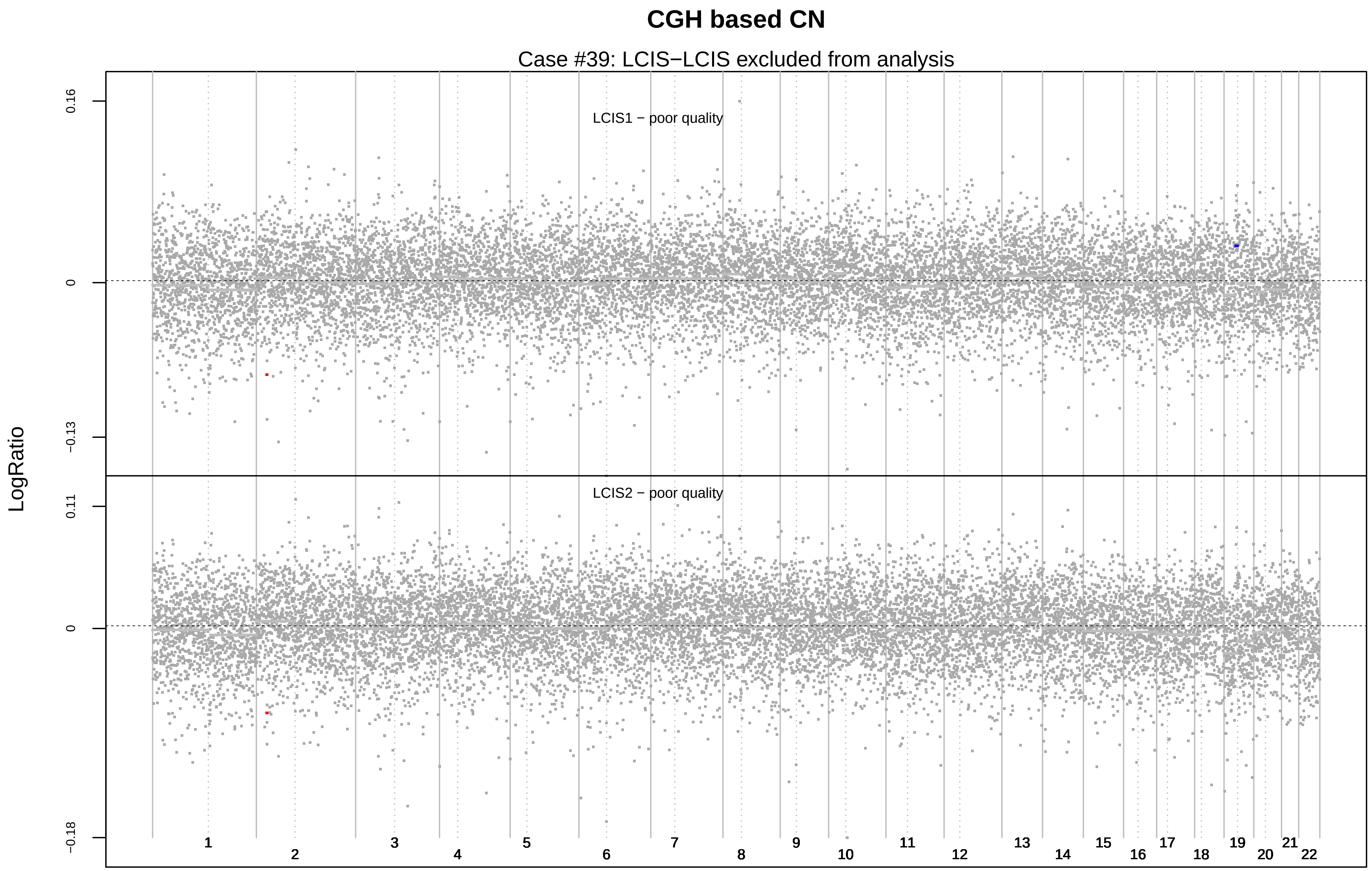

CGH based CN
Case #39: LCIS−LCIS excluded from analysis
LCIS1 − poor quality
LCIS2 − poor quality
LogRatio
0.16
0
−0.13
0.11
0
−0.18
1
2
3
4
5
6
7
8
9
10
11
12
13
14
15
16
17
18
19
20
21
22

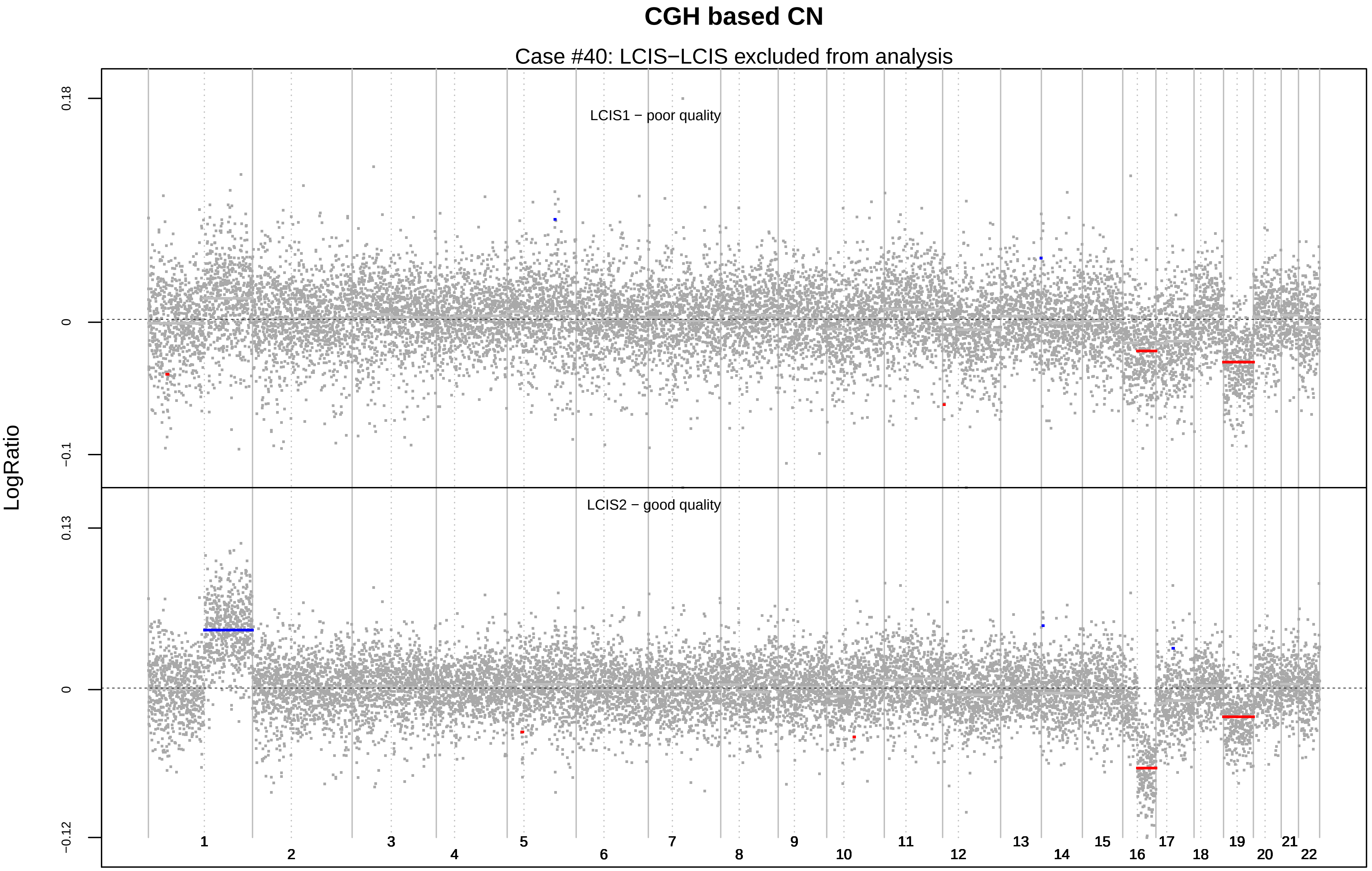
CGH based CN
Case #40: LCIS−LCIS excluded from analysis
LCIS1 − poor quality
LCIS2 − good quality
LogRatio
0.18
0
−0.1
0.13
0
−0.12
1
2
3
4
5
6
7
8
9
10
11
12
13
14
15
16
17
18
19
20
21
22

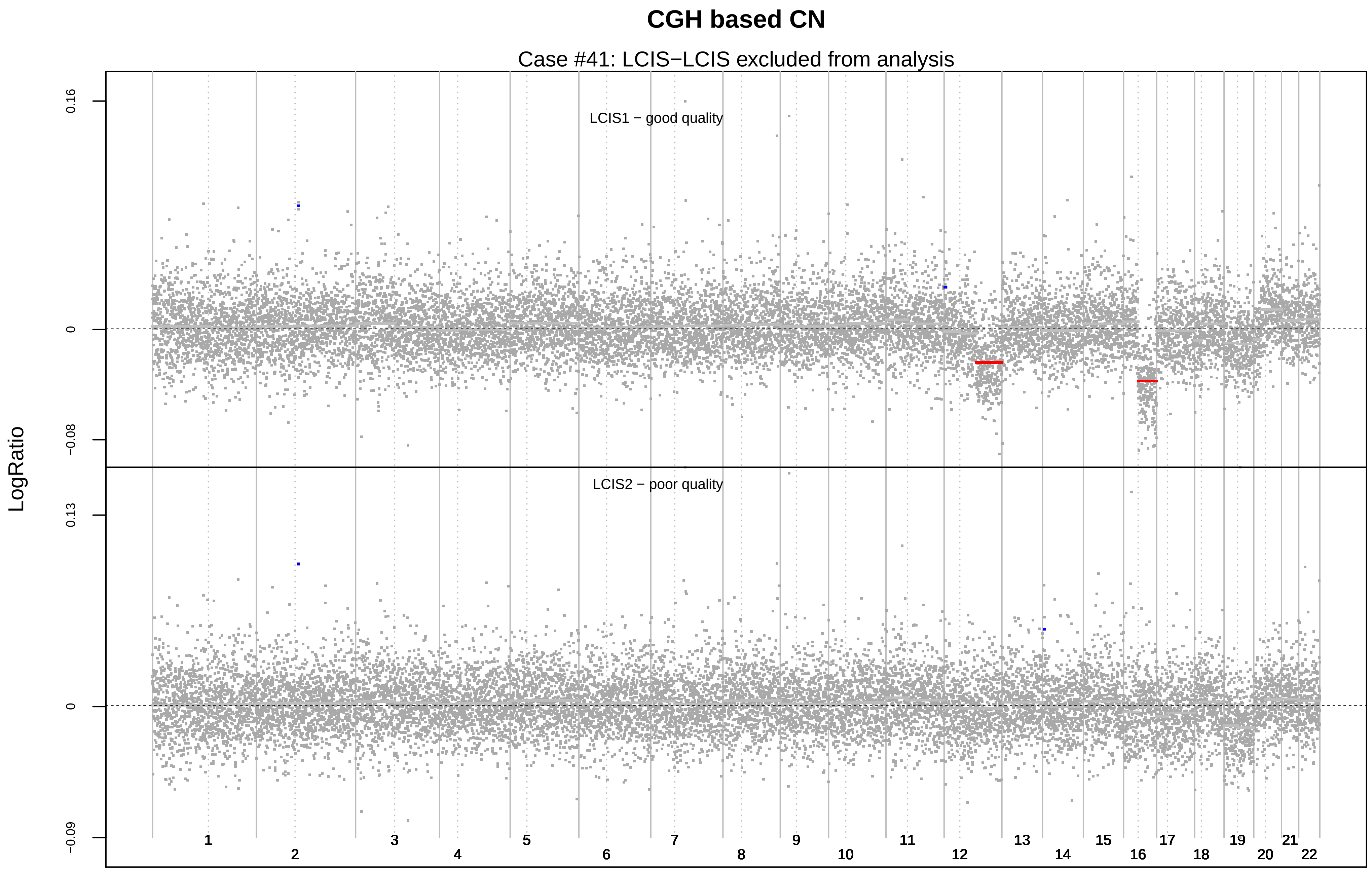

CGH based CN
Case #41: LCIS−LCIS excluded from analysis
LCIS1 − good quality
LCIS2 − poor quality
LogRatio
0.16
0
−0.08
0.13
0
−0.09
1
2
3
4
5
6
7
8
9
10
11
12
13
14
15
16
17
18
19
20
21
22

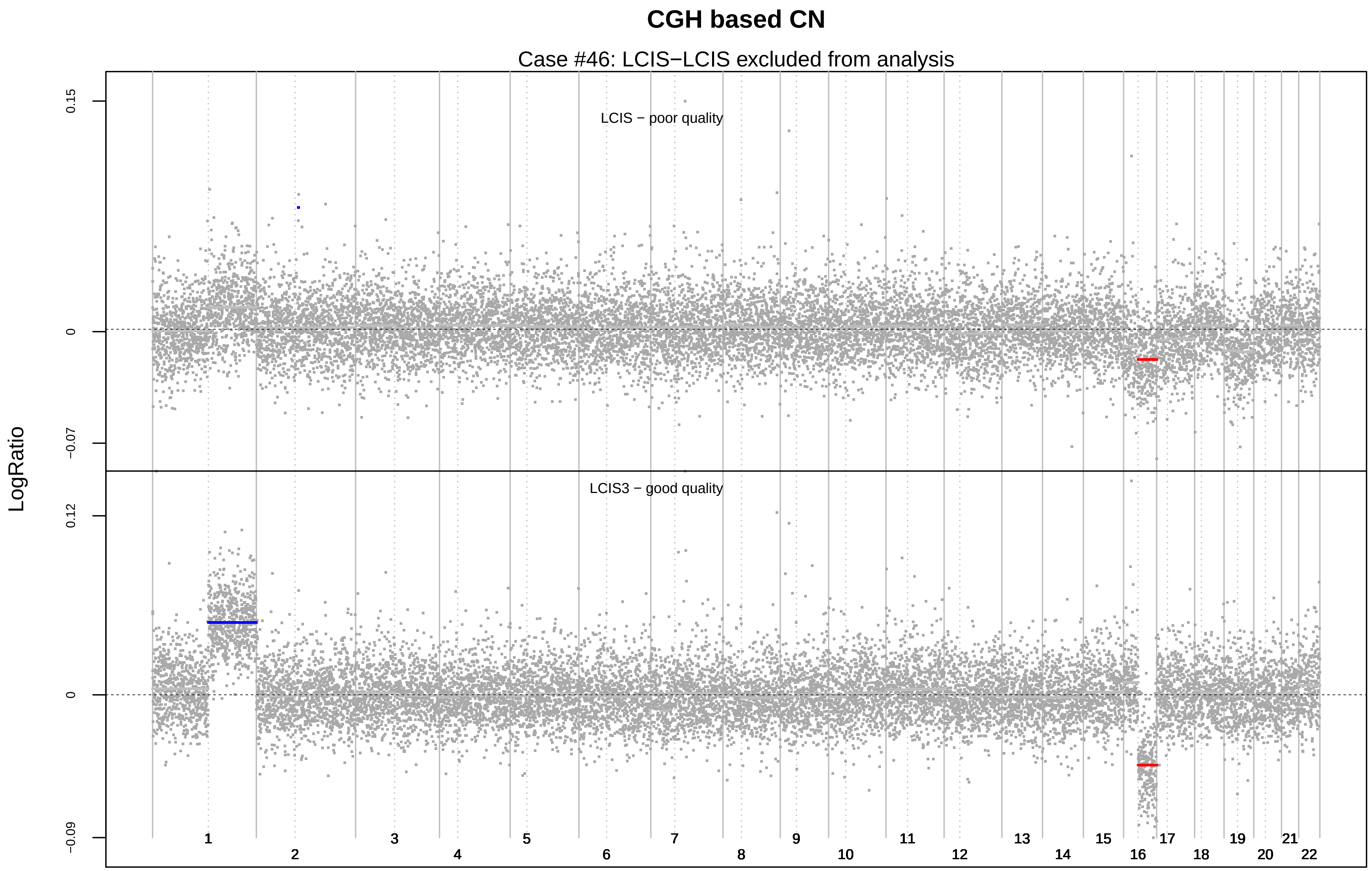
CGH based CN
Case #46: LCIS−LCIS excluded from analysis
LCIS − poor quality
LCIS3 − good quality
LogRatio
0.15
0
−0.07
0.12
0
−0.09
1
2
3
4
5
6
7
8
9
10
11
12
13
14
15
16
17
18
19
20
21
22

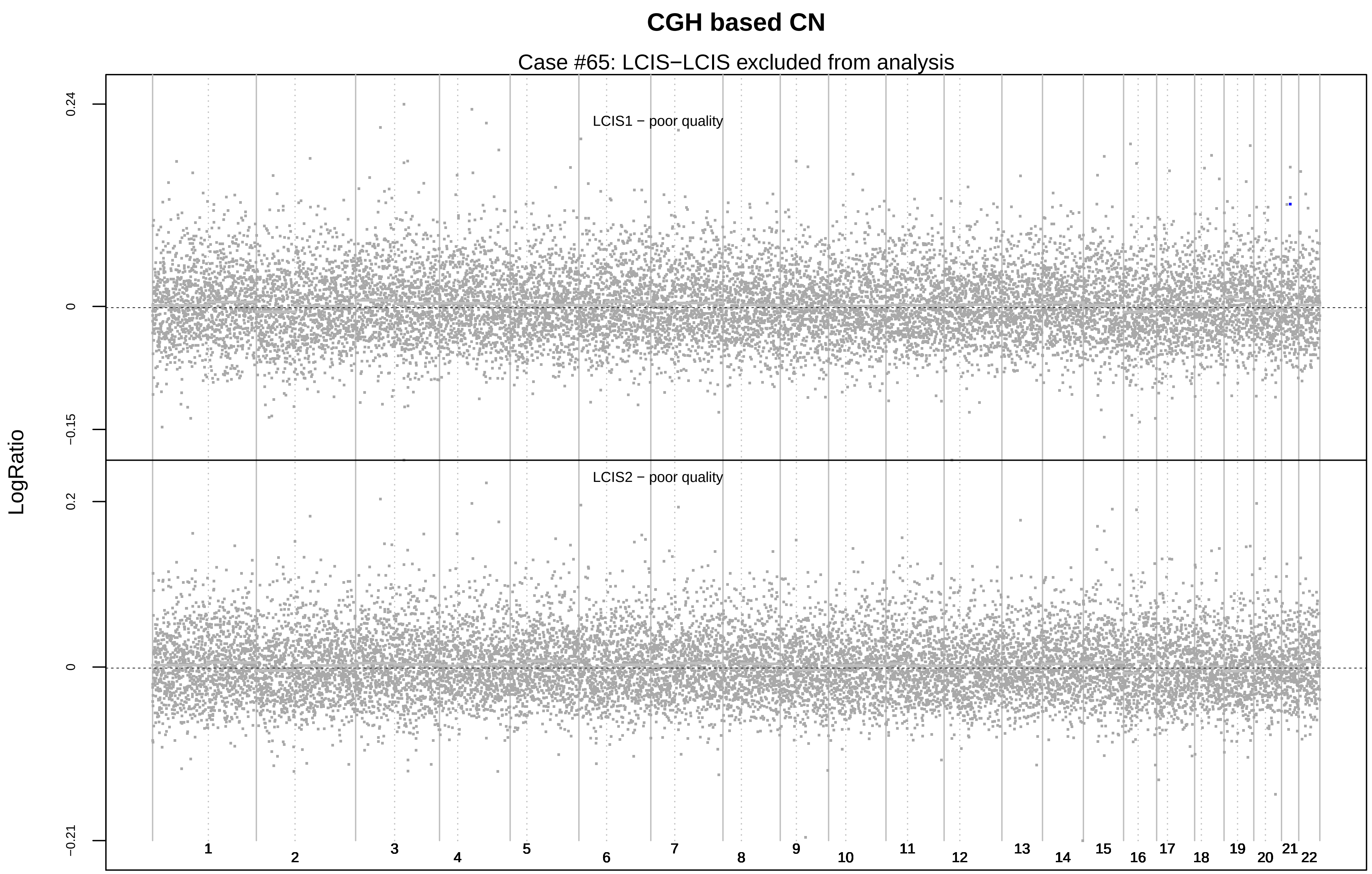

CGH based CN
Case #65: LCIS−LCIS excluded from analysis
LCIS1 − poor quality
LCIS2 − poor quality
LogRatio
0.24
0
−0.15
0.2
0
−0.21
1
2
3
4
5
6
7
8
9
10
11
12
13
14
15
16
17
18
19
20
21
22

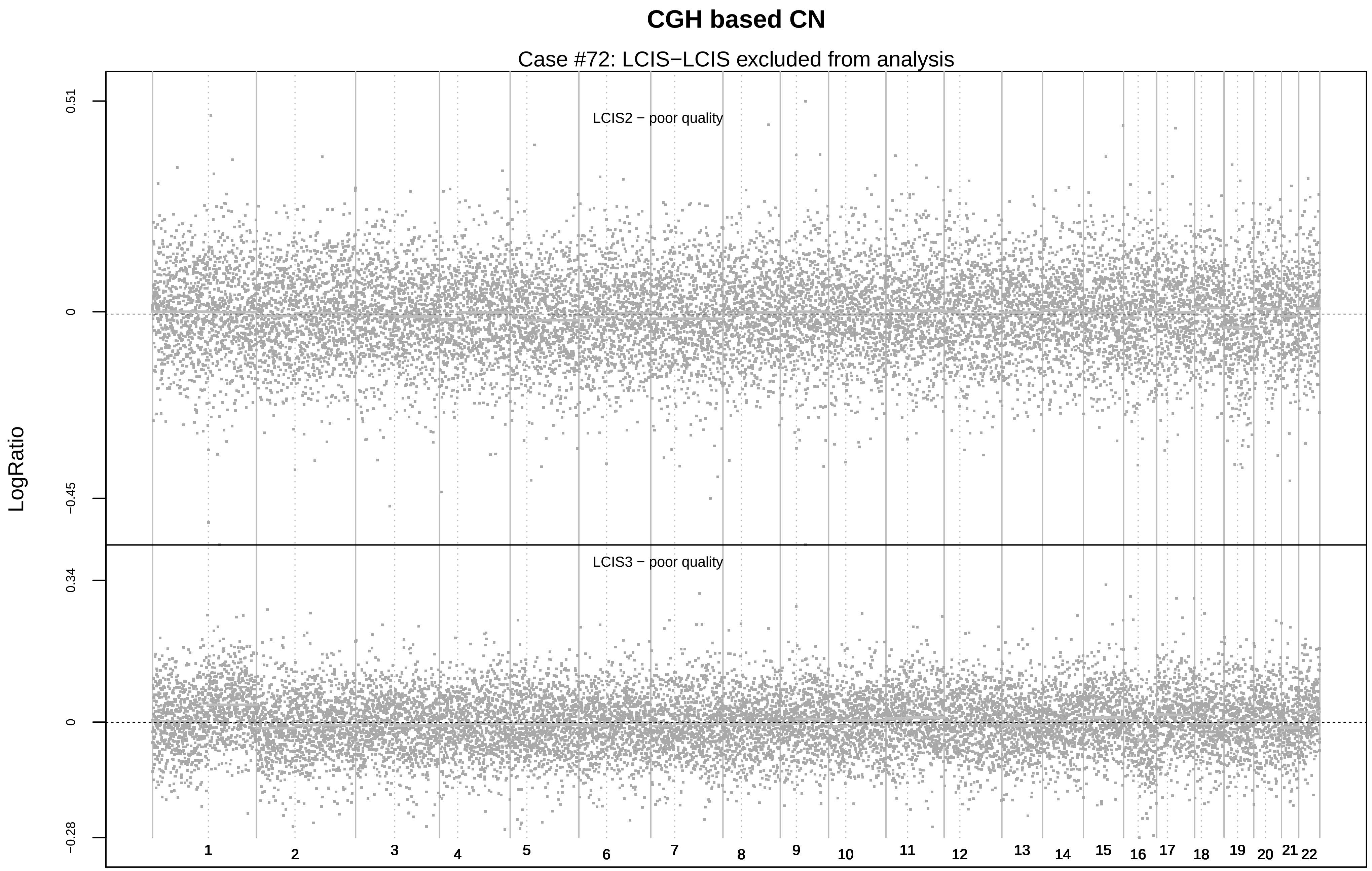
CGH based CN
Case #72: LCIS−LCIS excluded from analysis
LCIS2 − poor quality
LCIS3 − poor quality
LogRatio
0.51
0
−0.45
0.34
0
−0.28
1
2
3
4
5
6
7
8
9
10
11
12
13
14
15
16
17
18
19
20
21
22

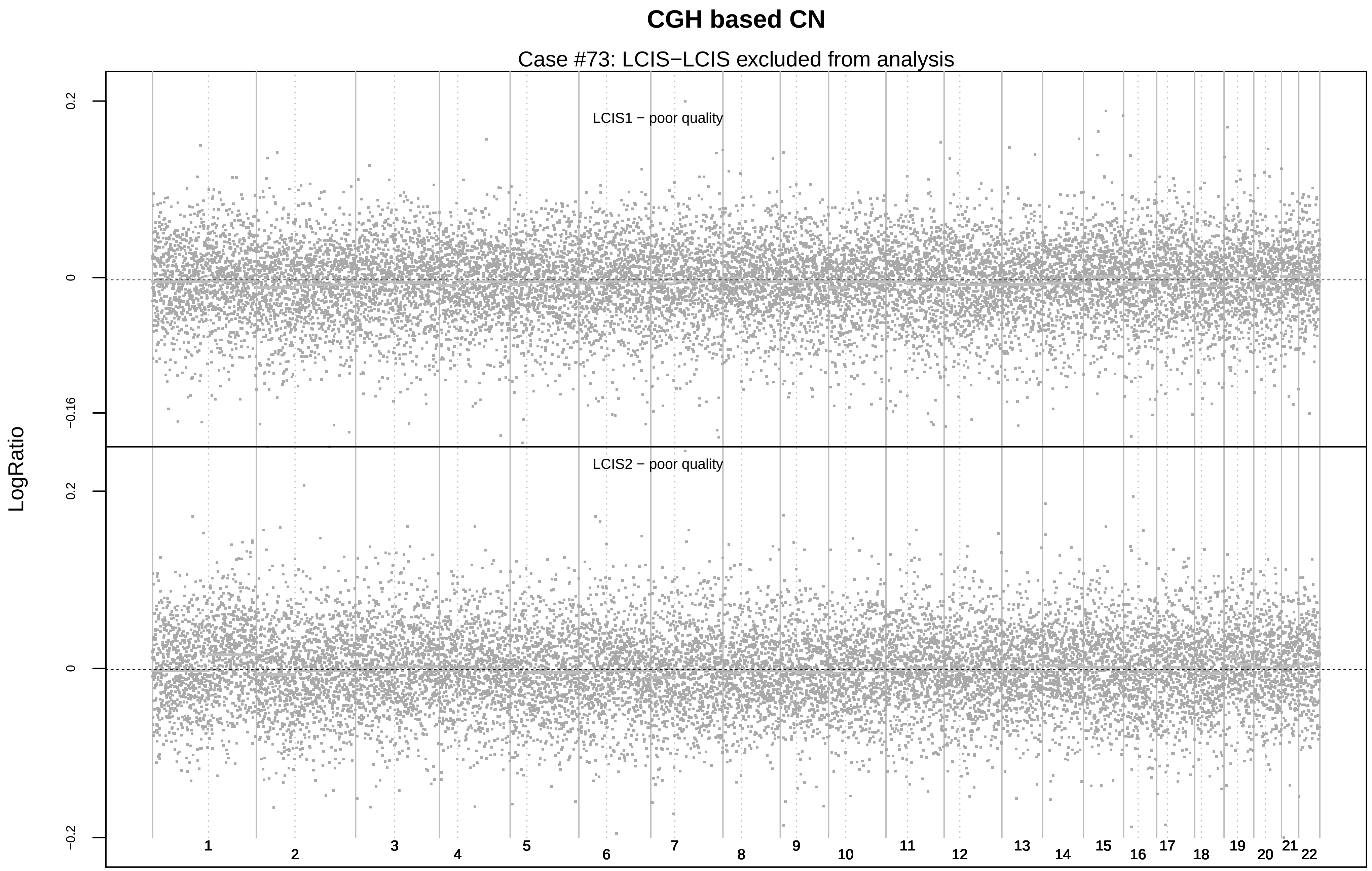

CGH based CN
Case #73: LCIS−LCIS excluded from analysis
LCIS1 − poor quality
LCIS2 − poor quality
LogRatio
0.2
0
−0.16
0.2
0
−0.2
1
2
3
4
5
6
7
8
9
10
11
12
13
14
15
16
17
18
19
20
21
22

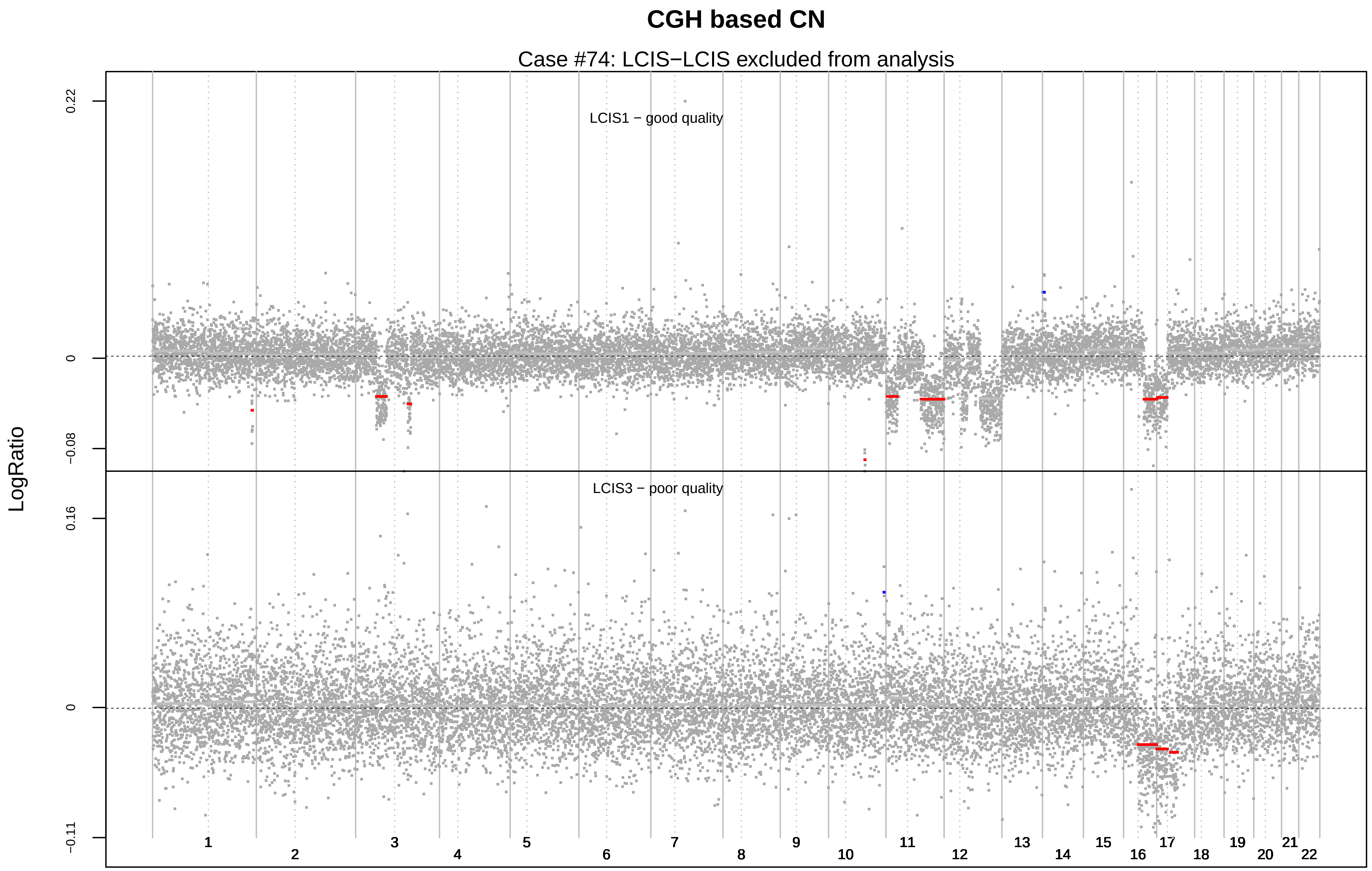

CGH based CN
Case #74: LCIS−LCIS excluded from analysis
LCIS1 − good quality
LCIS3 − poor quality
LogRatio
0.22
0
−0.08
0.16
0
−0.11
1
2
3
4
5
6
7
8
9
10
11
12
13
14
15
16
17
18
19
20
21
22

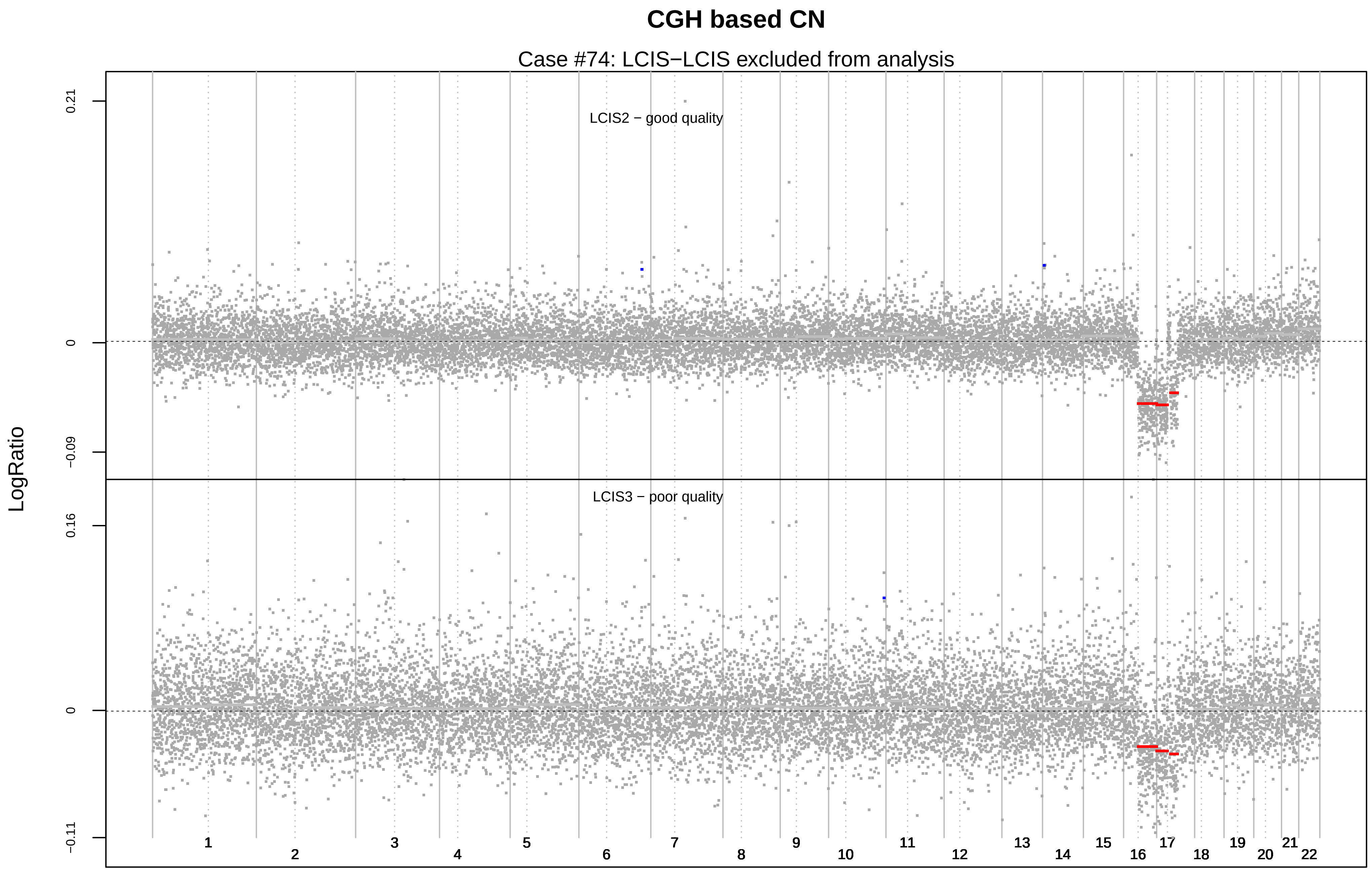
CGH based CN
Case #74: LCIS−LCIS excluded from analysis
LCIS2 − good quality
LCIS3 − poor quality
LogRatio
0.21
0
−0.09
0.16
0
−0.11
1 2 3 4 5 6 7 8 9 10 11 12 13 14 15 16 17 18 19 20 21 22

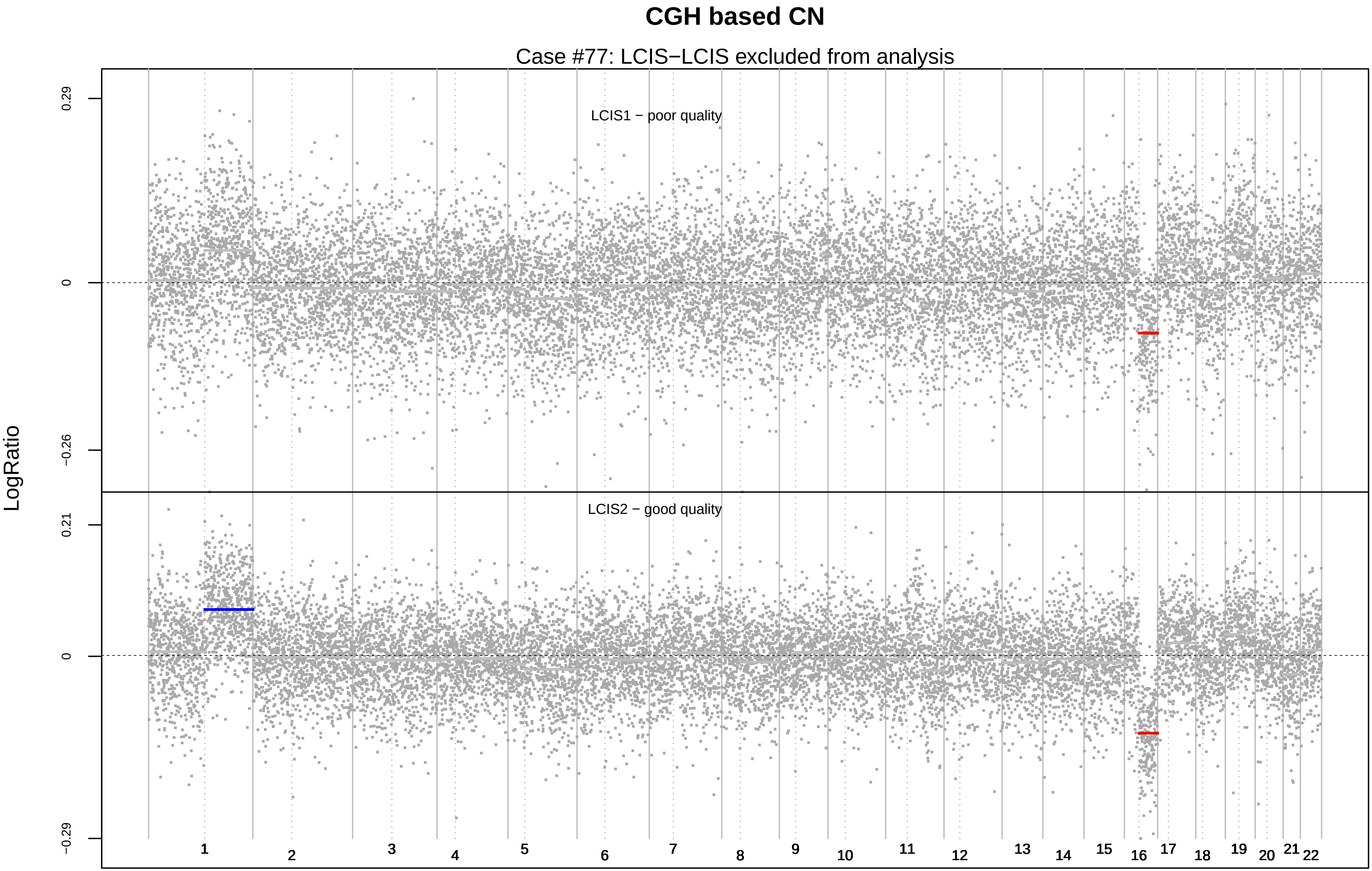

CGH based CN
Case #77: LCIS−LCIS excluded from analysis
LCIS1 − poor quality
LCIS2 − good quality
LogRatio
0.29
0
−0.26
0.21
0
−0.29
1
2
3
4
5
6
7
8
9
10
11
12
13
14
15
16
17
18
19
20
21
22

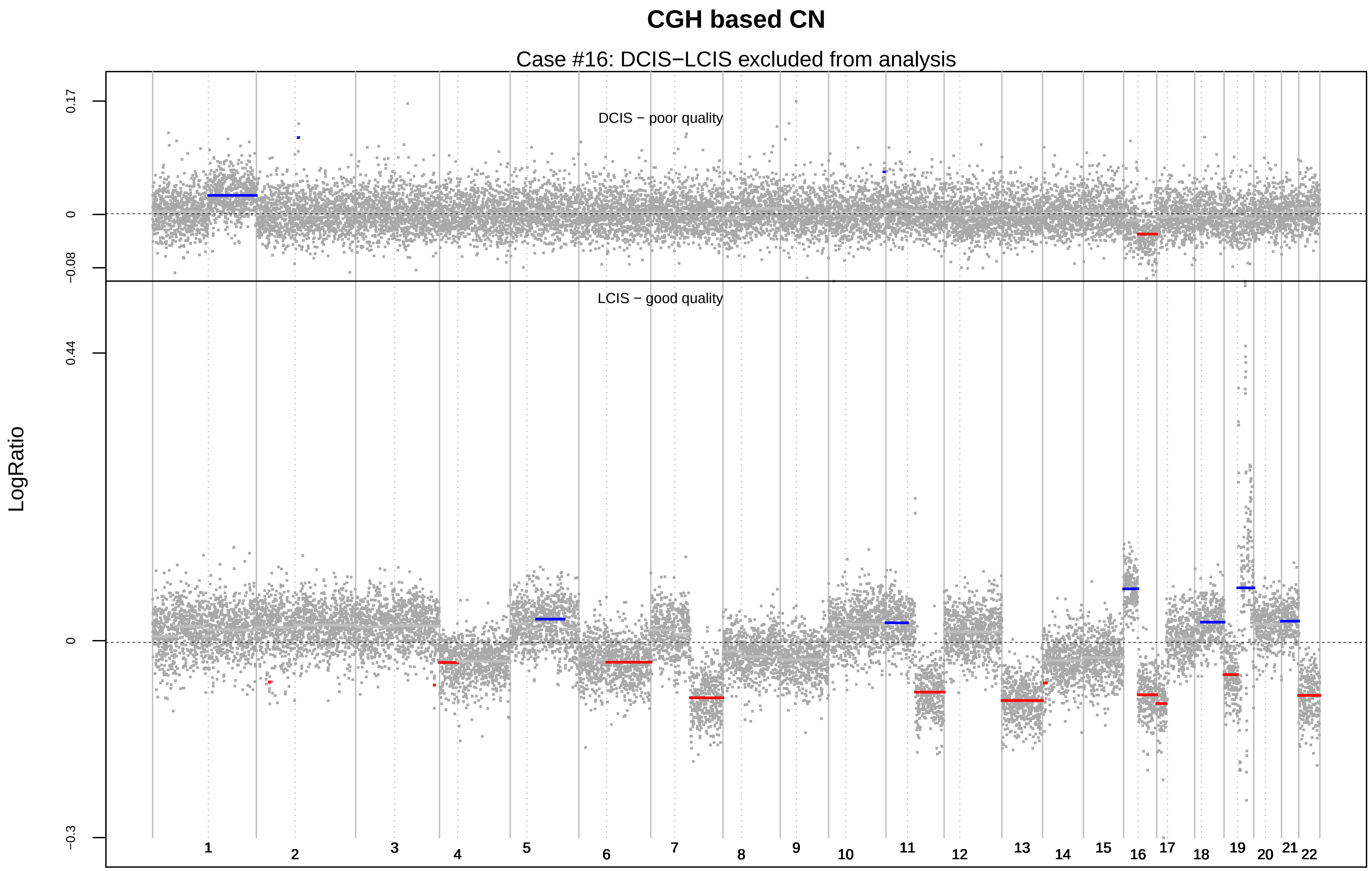
CGH based CN
Case #16: DCIS−LCIS excluded from analysis
DCIS − poor quality
LCIS − good quality
LogRatio
0.17
0
−0.08
0.44
0
−0.3
1
2
3
4
5
6
7
8
9
10
11
12
13
14
15
16
17
18
19
20
21
22

CGH based CN

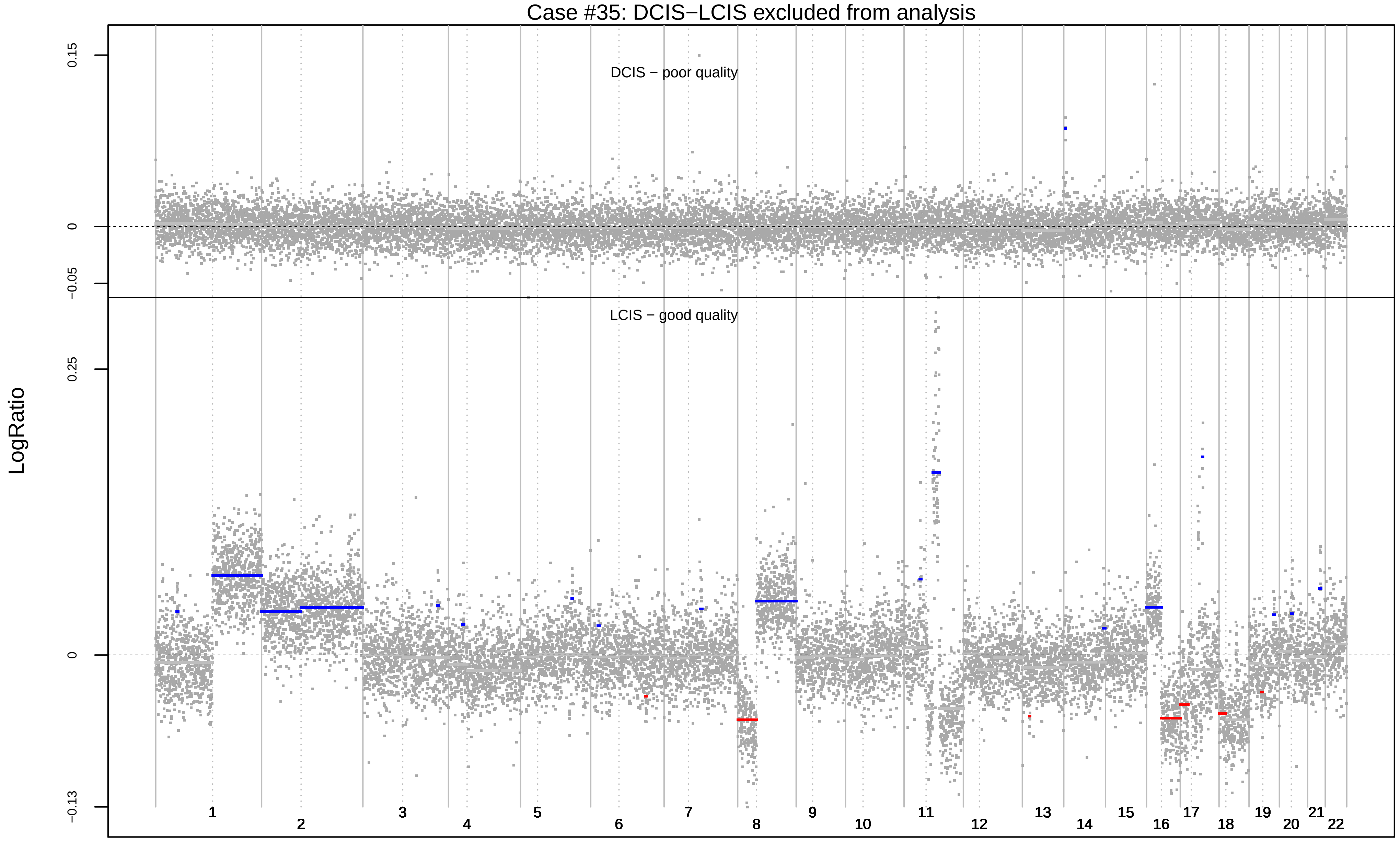
Case #35: DCIS−LCIS excluded from analysis
DCIS − poor quality
LCIS − good quality
LogRatio
0.15
0
−0.05
0.25
0
−0.13
1
2
3
4
5
6
7
8
9
10
11
12
13
14
15
16
17
18
19
20
21
22

CGH based CN

Case #57: DCIS−LCIS excluded from analysis

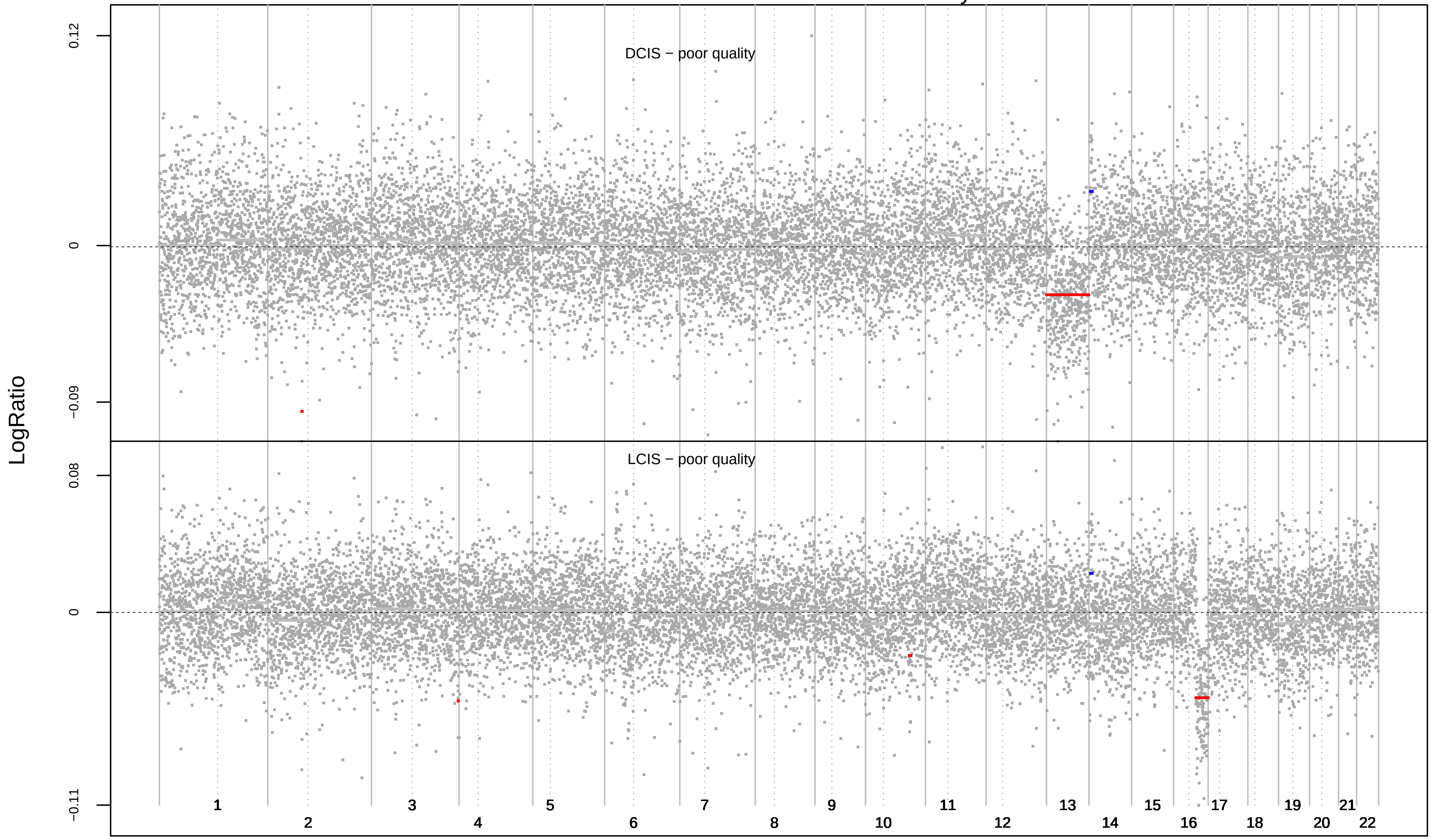

Supplement: Additional file 1: — Separate plots are provided for copy number comparisons of all pairs of lesions ascertained in the study, including those comparisons not presented in the main text because one or other of the tumors in the pair was considered to have insufficient quality. We used two quality metrics: percent gained or lost; 75th percentile of |height| of gains or losses (at least ten markers long) divided by the median absolute deviation of the residuals. The quality was considered to be sufficiently good if either the percent gained or lost was >10 % or if the |height| percentile was >1.75 median absolute deviation. The figures display the log ratios for each marker ordered across the genome, side by side for each tumor in the pair. The blue lines indicate regions of allelic gain, and the red lines indicate regions of allelic loss, as determined by the segmentation algorithm used [21]. (PDF 19100 kb) [file 13058_2016_727_MOESM1_ESM.pdf]
