# Supplementary material for: Clonal relationships between lobular carcinoma in situ and other breast malignancies
Source: Breast Cancer Res. 2016 Jun 23;18:66. doi: 10.1186/s13058-016-0727-z (PMC4918003; doi:10.1186/s13058-016-0727-z)
Supplement: Additional file 2: — Separate plots are provided for copy number comparisons imputed from the exome sequencing platforms for all cases where good-quality data were obtained from use of both this method and the method based on comparative genomic hybridization. These plots can be compared with the corresponding plots obtained using comparative genomic hybridization in Additional file 1. For most tumors, the copy number patterns derived from the two methods are very similar. p Values are compared in the table in Additional file 4. (PDF 7994 kb) [file 13058_2016_727_MOESM2_ESM.pdf]

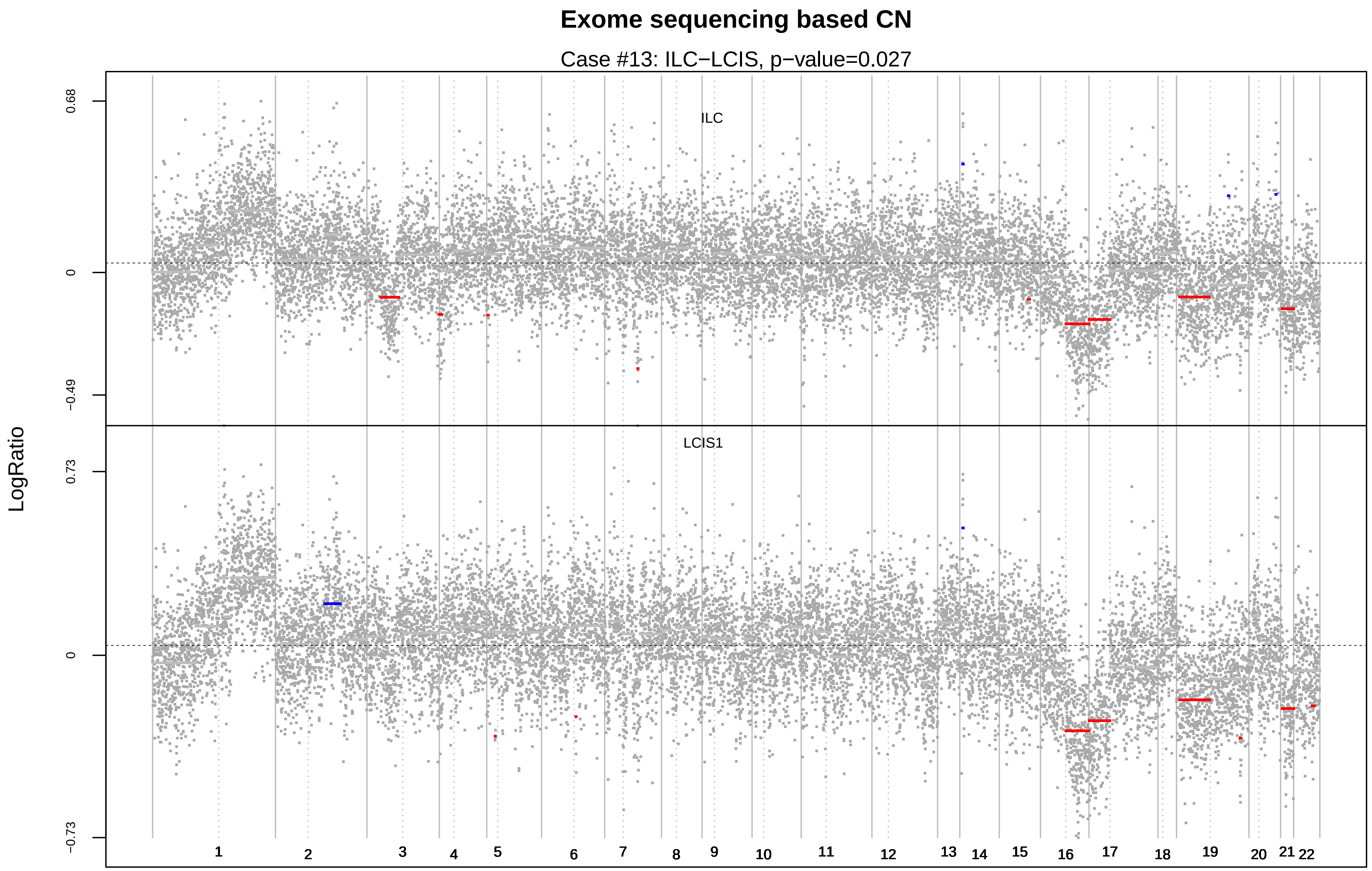

Exome sequencing based CN
Case #13: ILC−LCIS, p−value=0.027
LogRatio
ILC
LCIS1
0.68
0
−0.49
0.73
0
−0.73
1 2 3 4 5 6 7 8 9 10 11 12 13 14 15 16 17 18 19 20 21 22

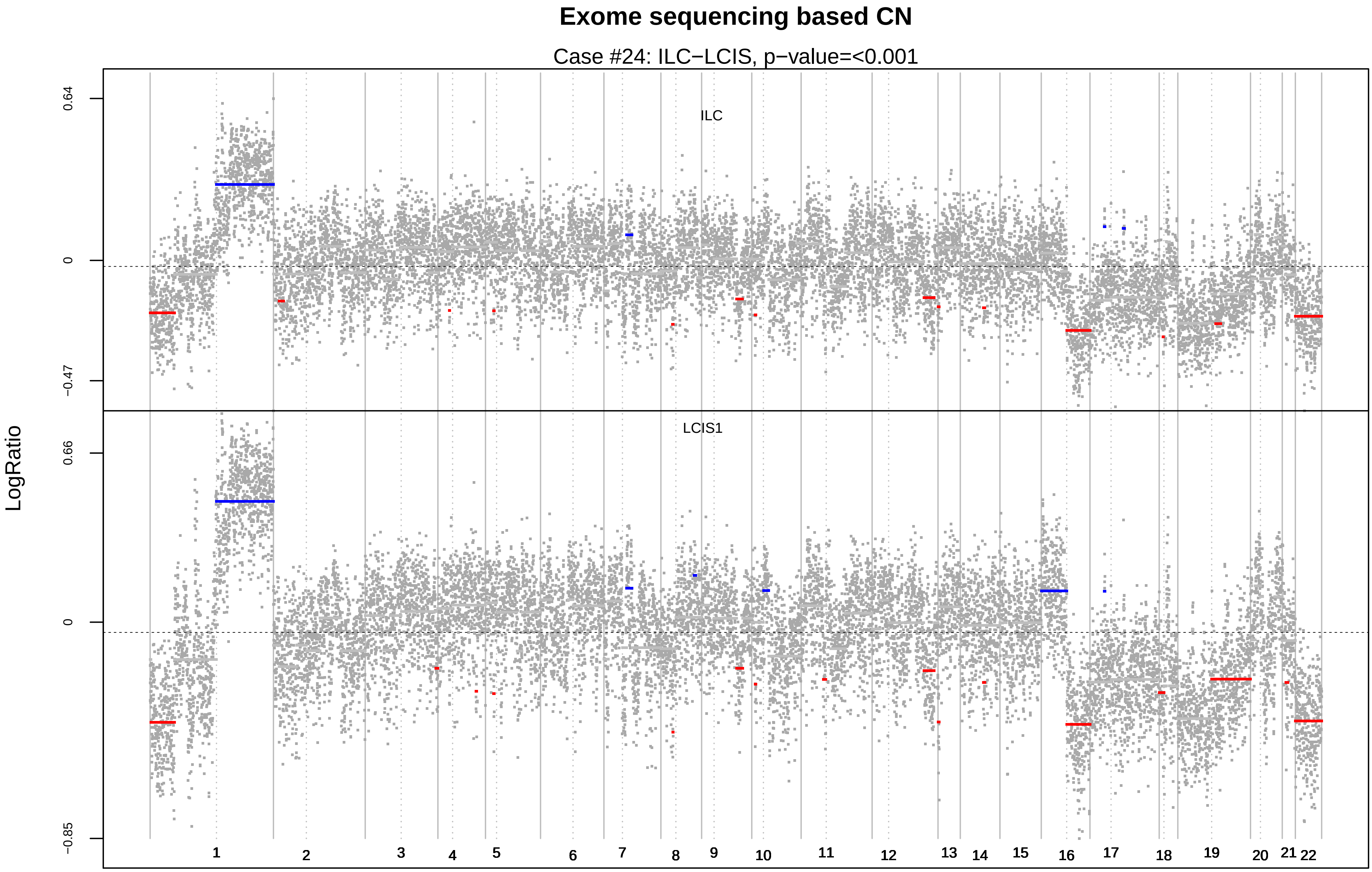

Exome sequencing based CN
Case #24: ILC−LCIS, p−value=<0.001
ILC
LCIS1
LogRatio
0.64
0
−0.47
0.66
0
−0.85
1
2
3
4
5
6
7
8
9
10
11
12
13
14
15
16
17
18
19
20
21
22

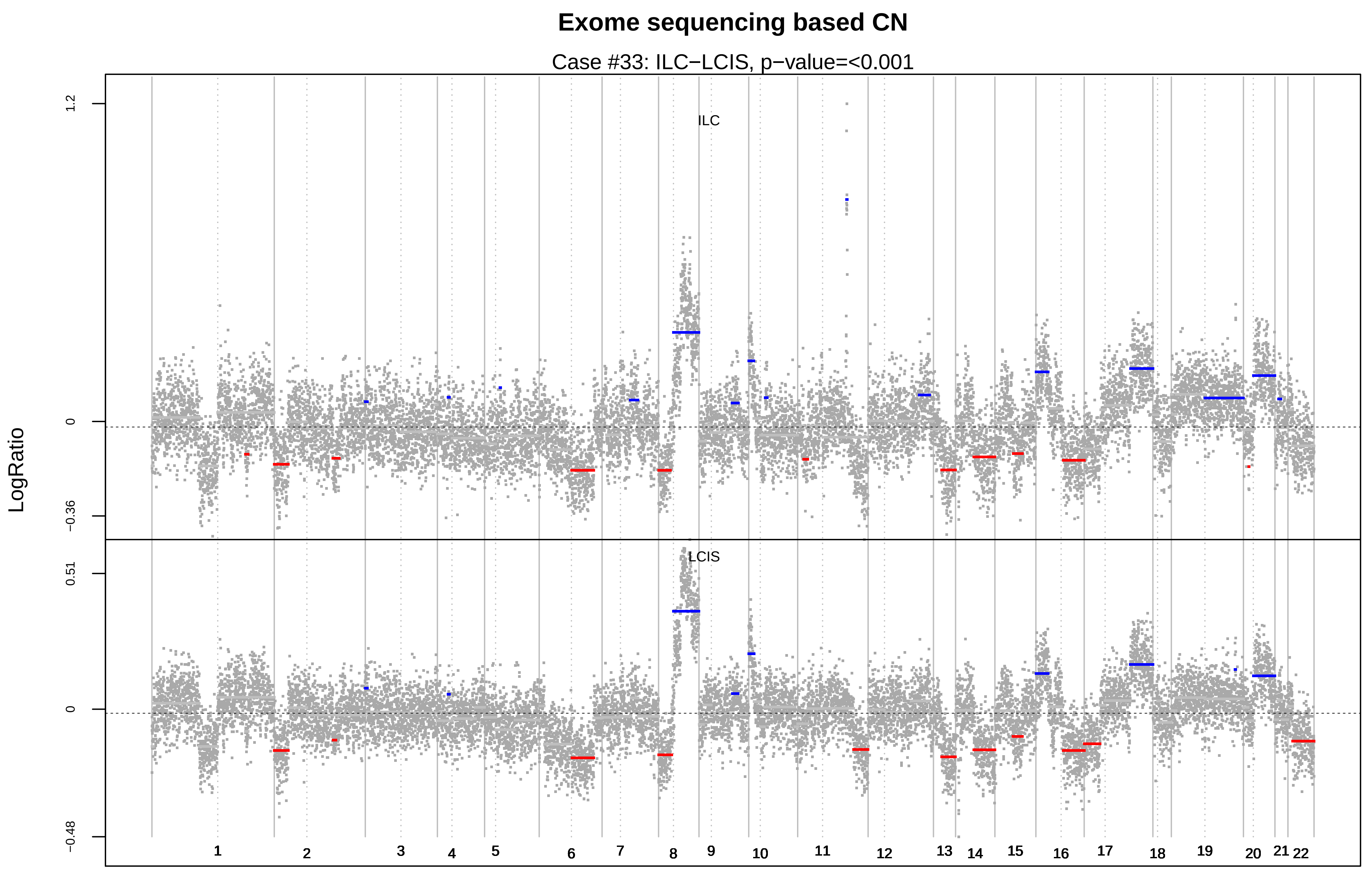

Exome sequencing based CN
Case #33: ILC−LCIS, p−value=<0.001
ILC
LCIS
LogRatio
1.2
0
−0.36
0.51
0
−0.48
1
2
3
4
5
6
7
8
9
10
11
12
13
14
15
16
17
18
19
20
21
22

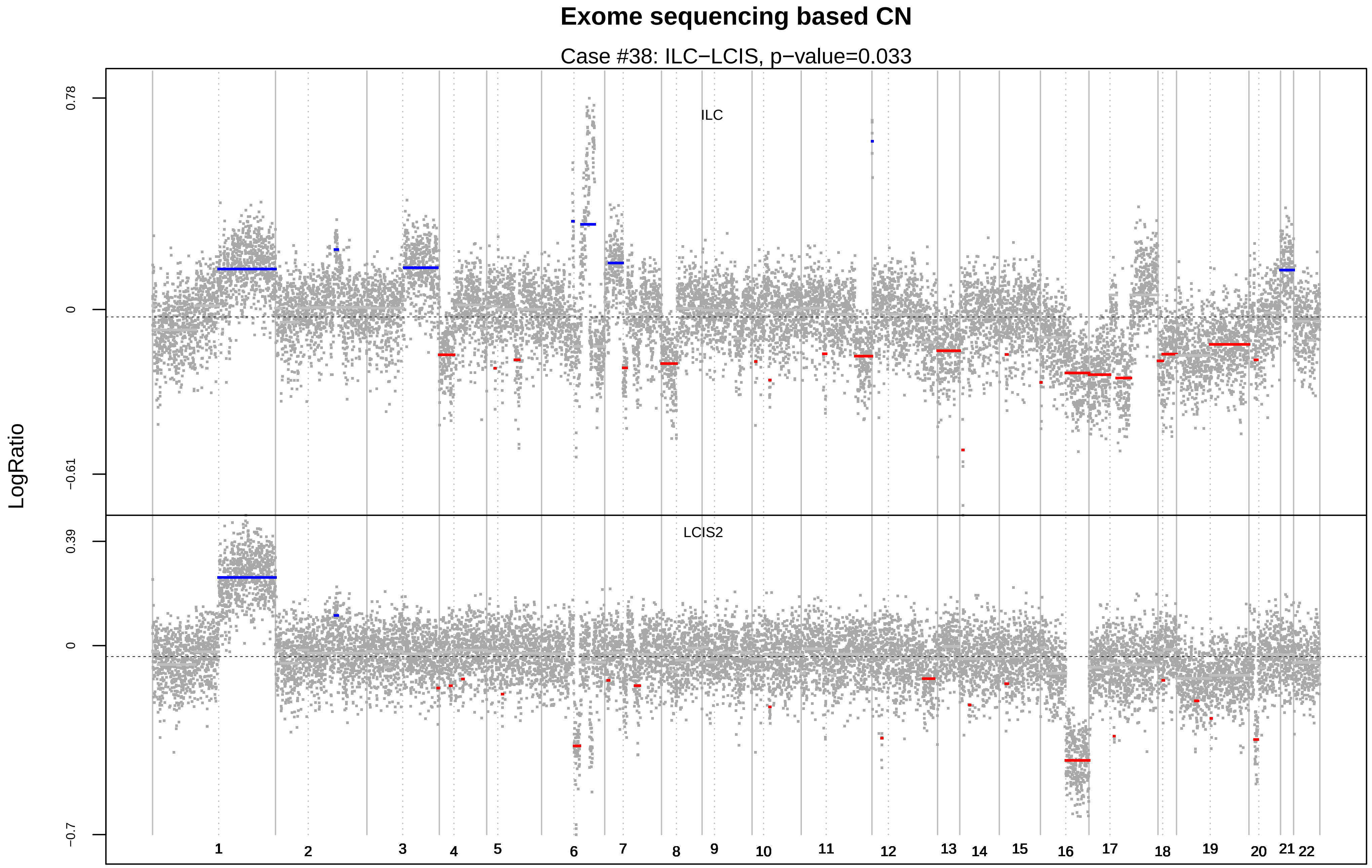

Exome sequencing based CN
Case #38: ILC−LCIS, p−value=0.033
LogRatio
ILC
LCIS2
0.78
0
−0.61
0.39
0
−0.7
1
2
3
4
5
6
7
8
9
10
11
12
13
14
15
16
17
18
19
20
21
22

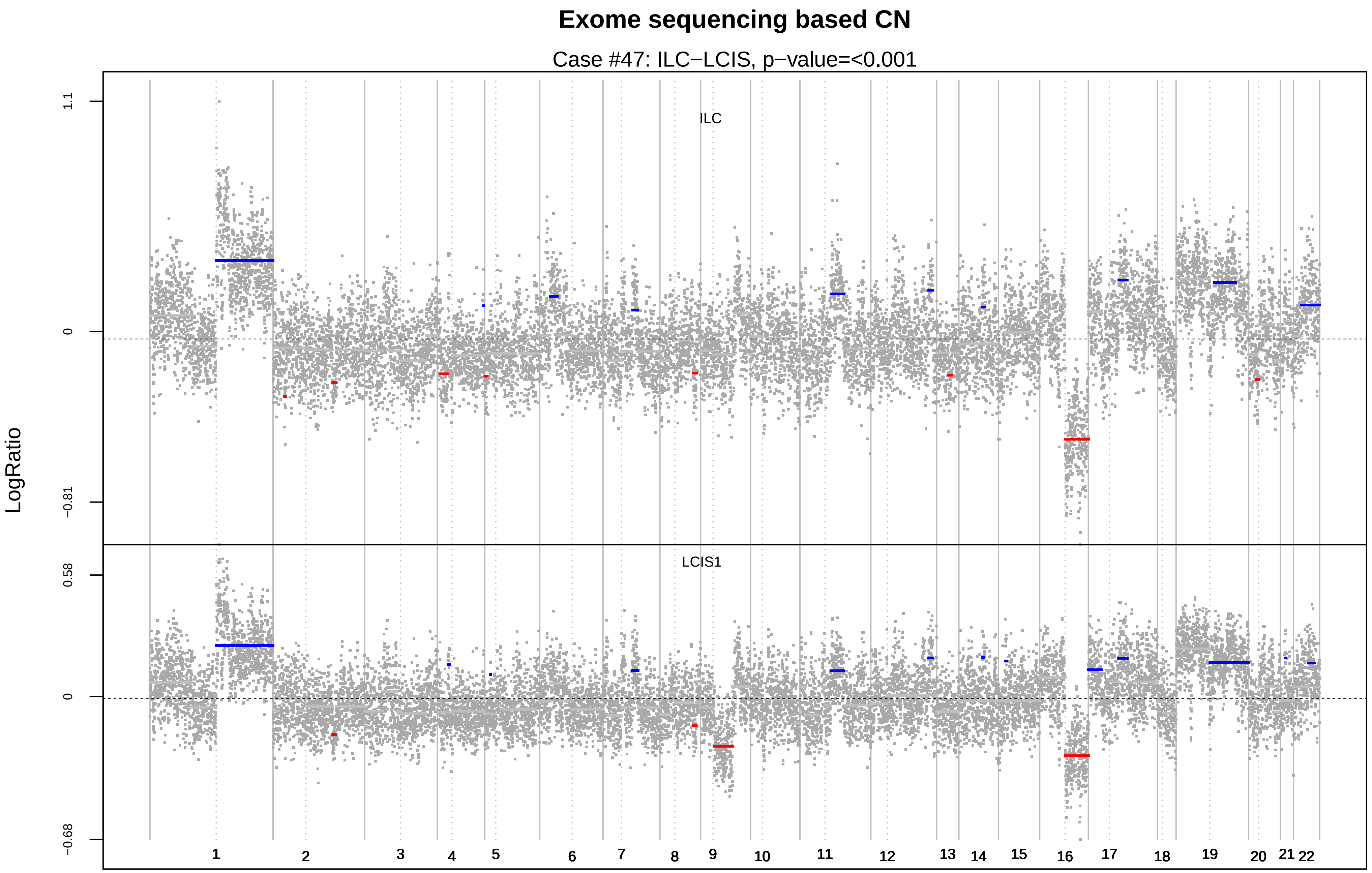

Exome sequencing based CN
Case #47: ILC−LCIS, p−value=<0.001
LogRatio
ILC
LCIS1
1.1
0
−0.81
0.58
0
−0.68
1
2
3
4
5
6
7
8
9
10
11
12
13
14
15
16
17
18
19
20
21
22

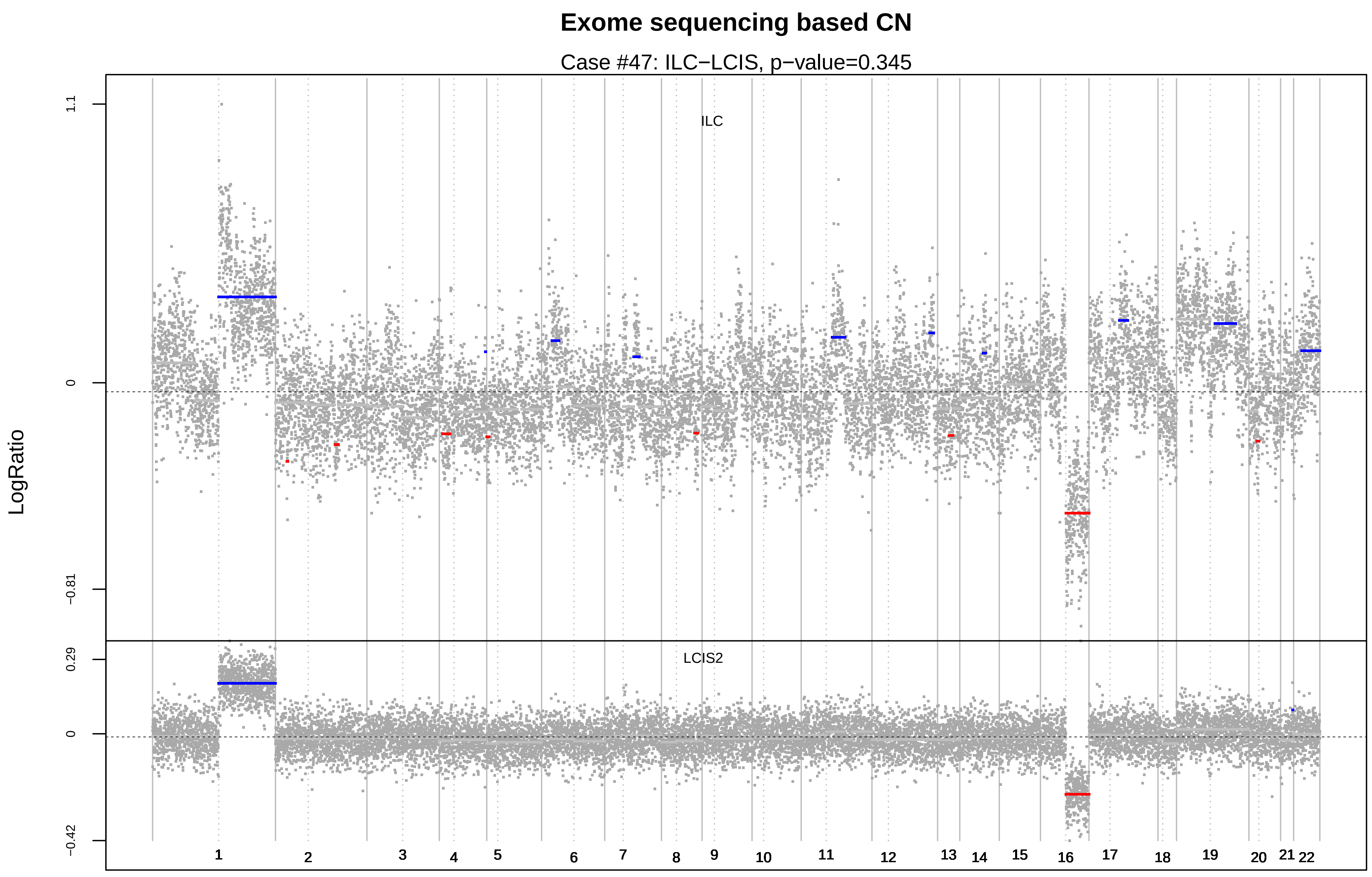

Exome sequencing based CN
Case #47: ILC−LCIS, p−value=0.345
LogRatio
ILC
LCIS2
1.1
0
−0.81
0.29
0
−0.42
1
2
3
4
5
6
7
8
9
10
11
12
13
14
15
16
17
18
19
20
21
22

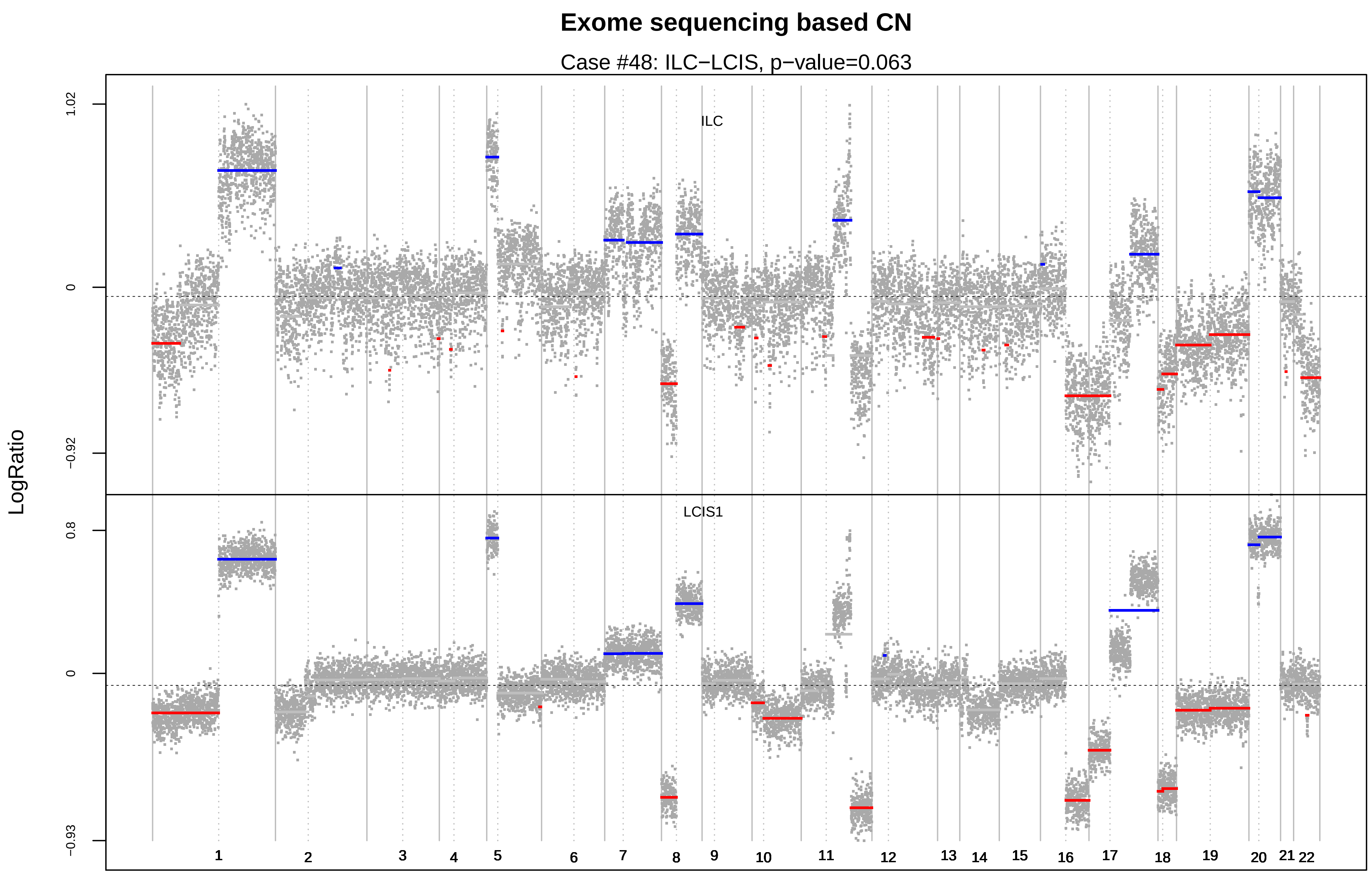

Exome sequencing based CN
Case #48: ILC−LCIS, p−value=0.063
LogRatio
1.02
0
−0.92
ILC
0.8
0
−0.93
LCIS1
1
2
3
4
5
6
7
8
9
10
11
12
13
14
15
16
17
18
19
20
21
22

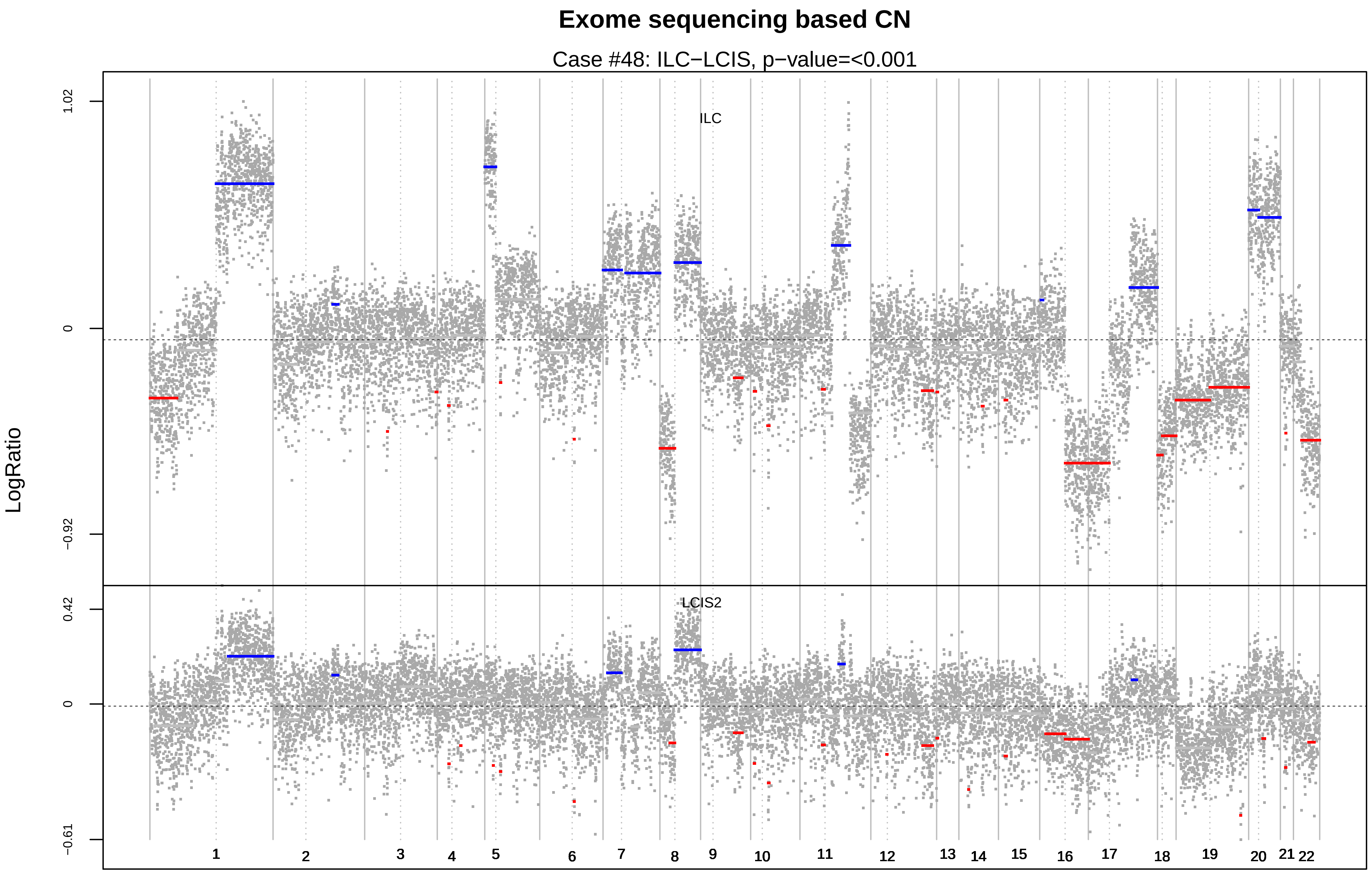

Exome sequencing based CN
Case #48: ILC−LCIS, p−value=<0.001
LogRatio
ILC
LCIS2
1.02
0
−0.92
0.42
0
−0.61
1
2
3
4
5
6
7
8
9
10
11
12
13
14
15
16
17
18
19
20
21
22

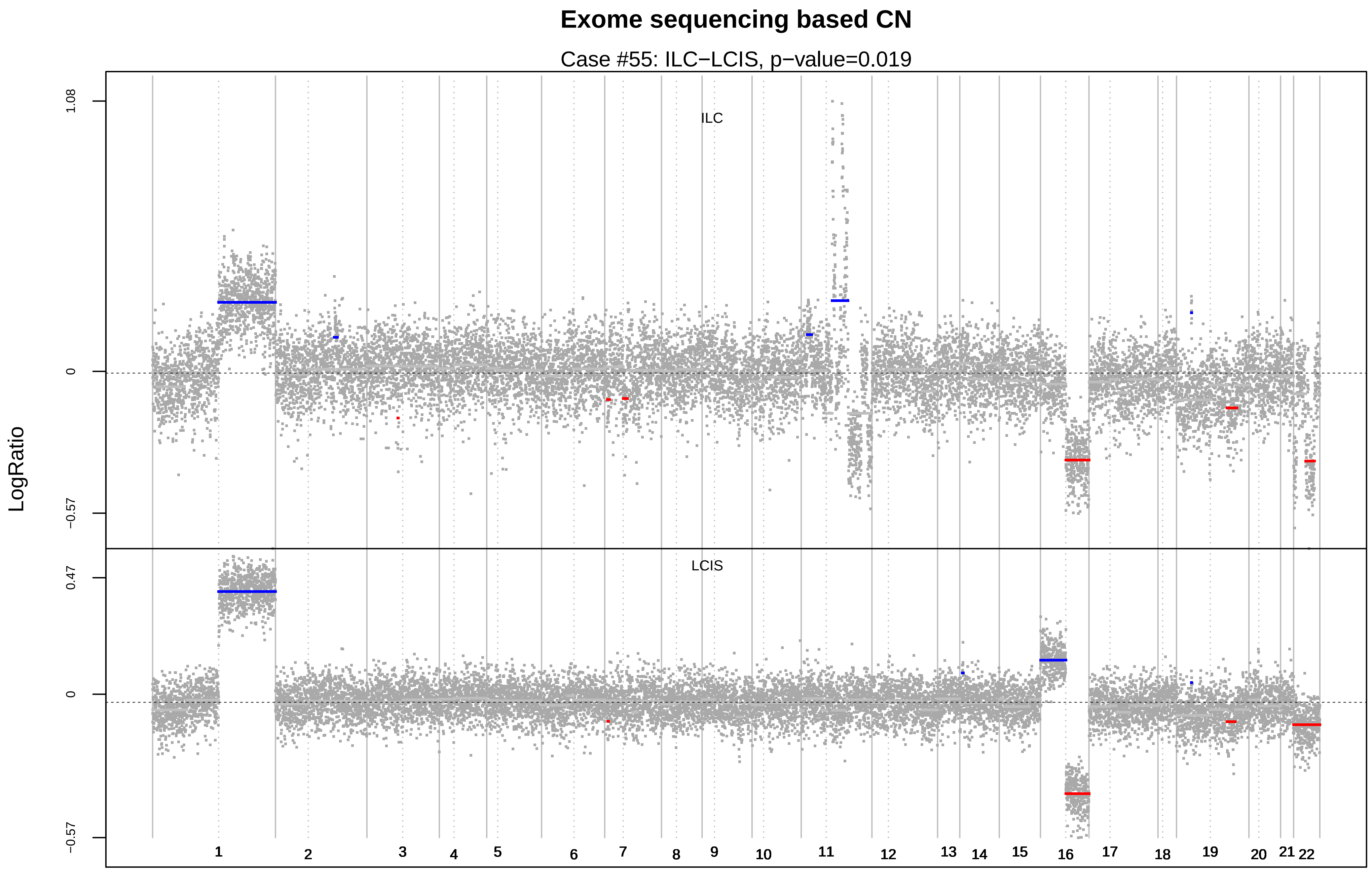
Exome sequencing based CN
Case #55: ILC−LCIS, p−value=0.019
LogRatio
ILC
LCIS
1.08
0
−0.57
0.47
0
−0.57
1
2
3
4
5
6
7
8
9
10
11
12
13
14
15
16
17
18
19
20
21
22

Exome sequencing based CN

Case #68: ILC−LCIS, p−value=0.286

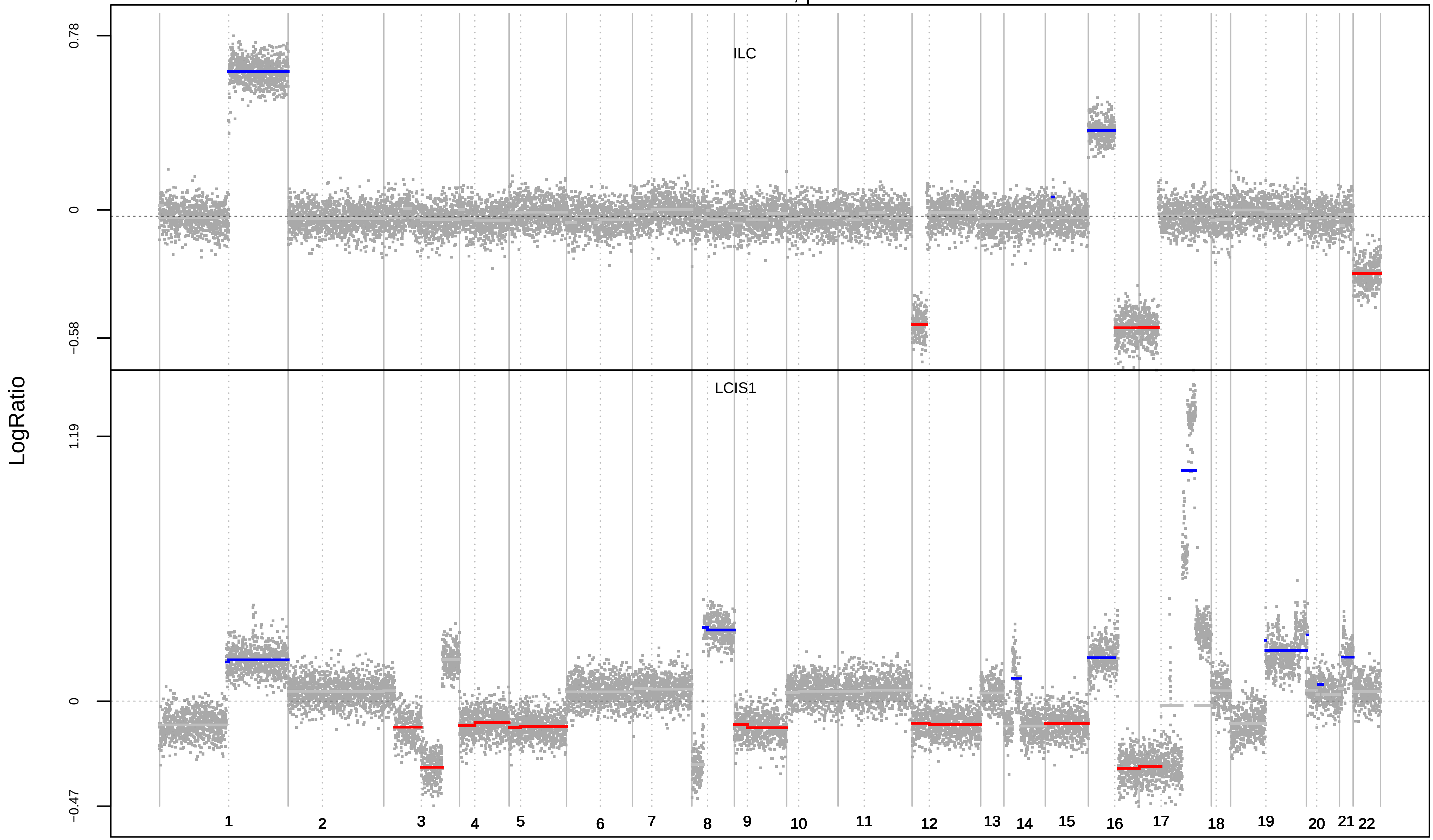

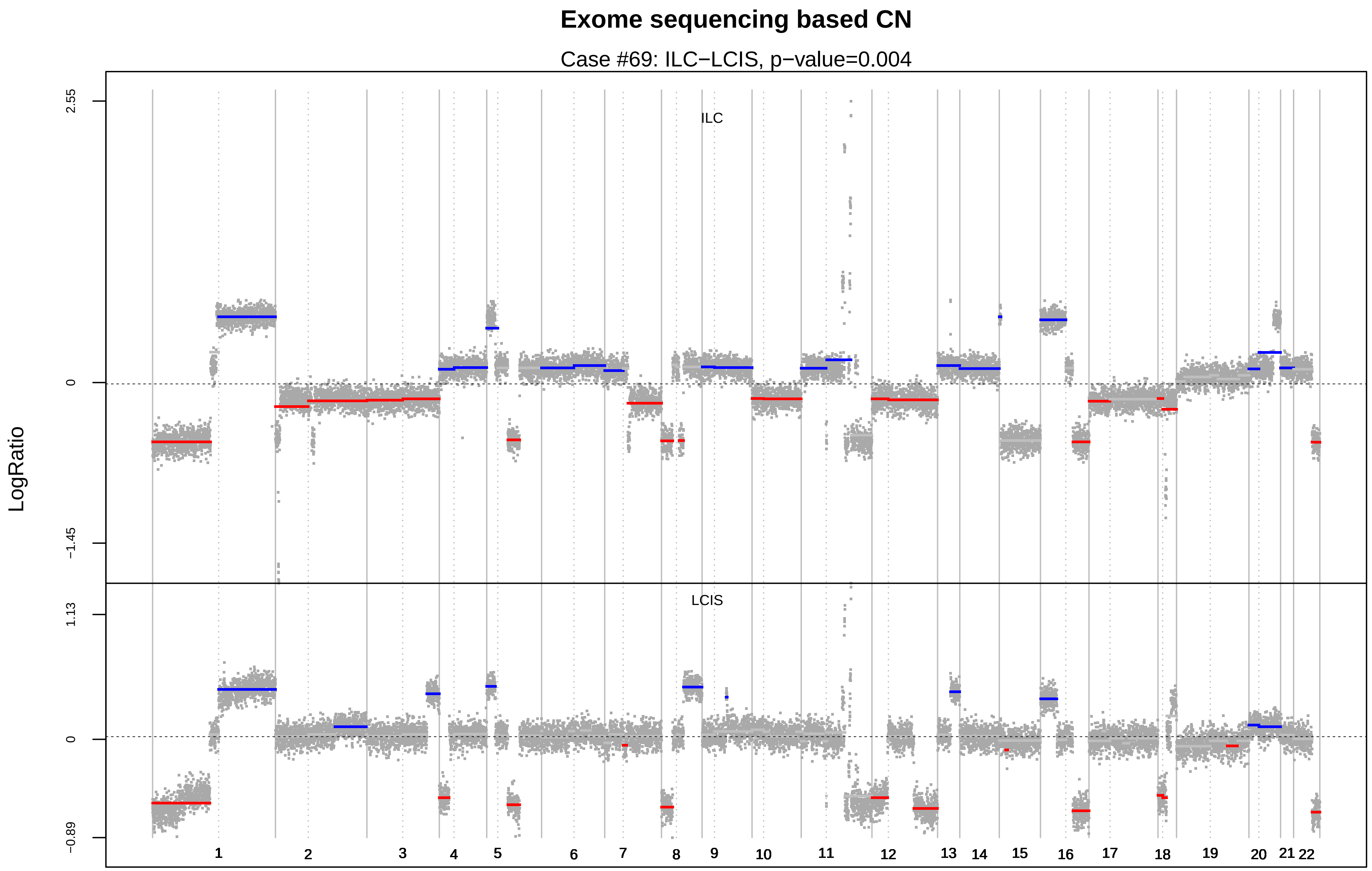

Exome sequencing based CN
Case #69: ILC−LCIS, p−value=0.004
LogRatio
2.55
0
−1.45
ILC
1.13
0
−0.89
LCIS
1
2
3
4
5
6
7
8
9
10
11
12
13
14
15
16
17
18
19
20
21
22

Exome sequencing based CN

Case #47: IDC−LCIS, p−value=0.215

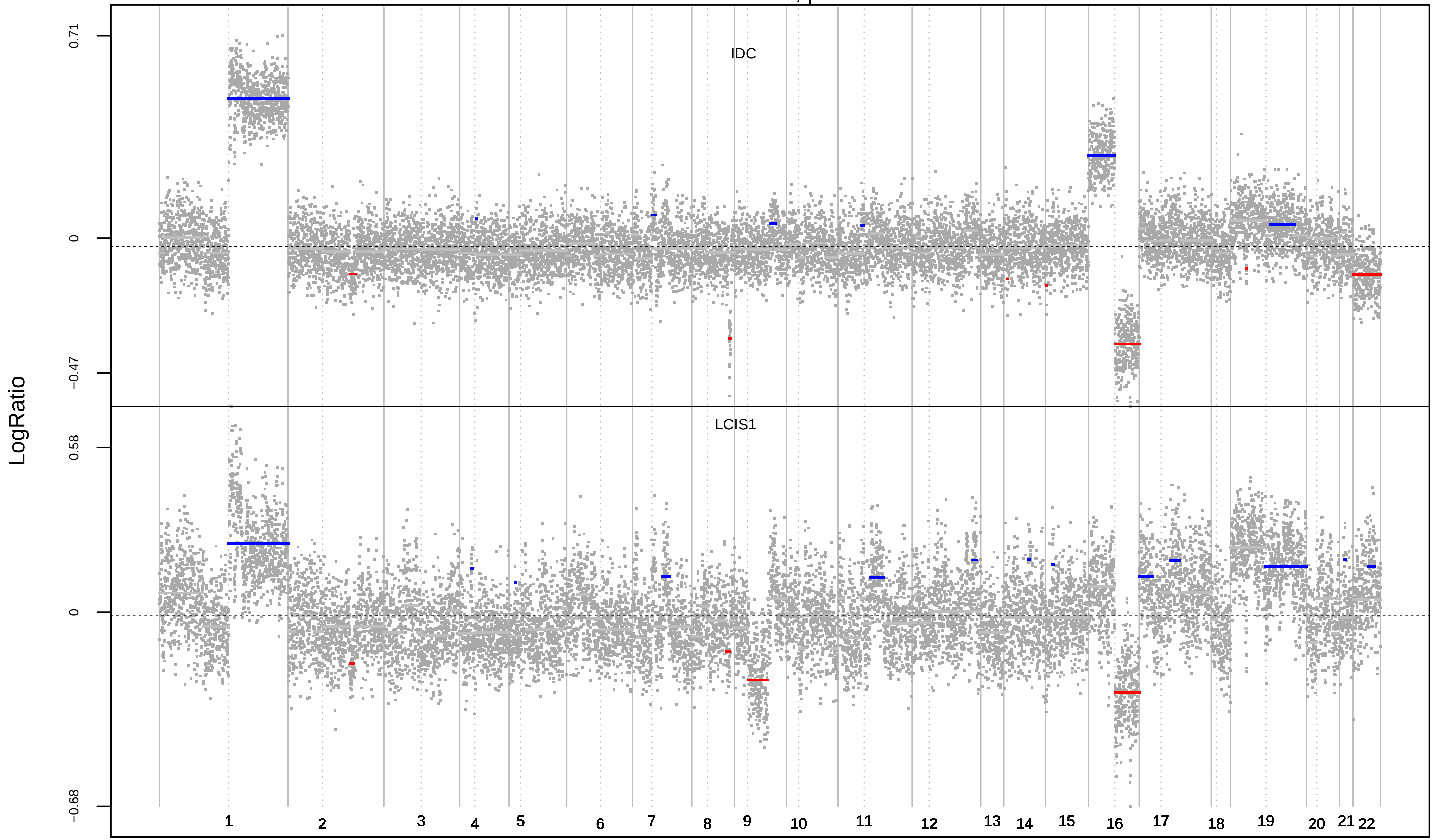

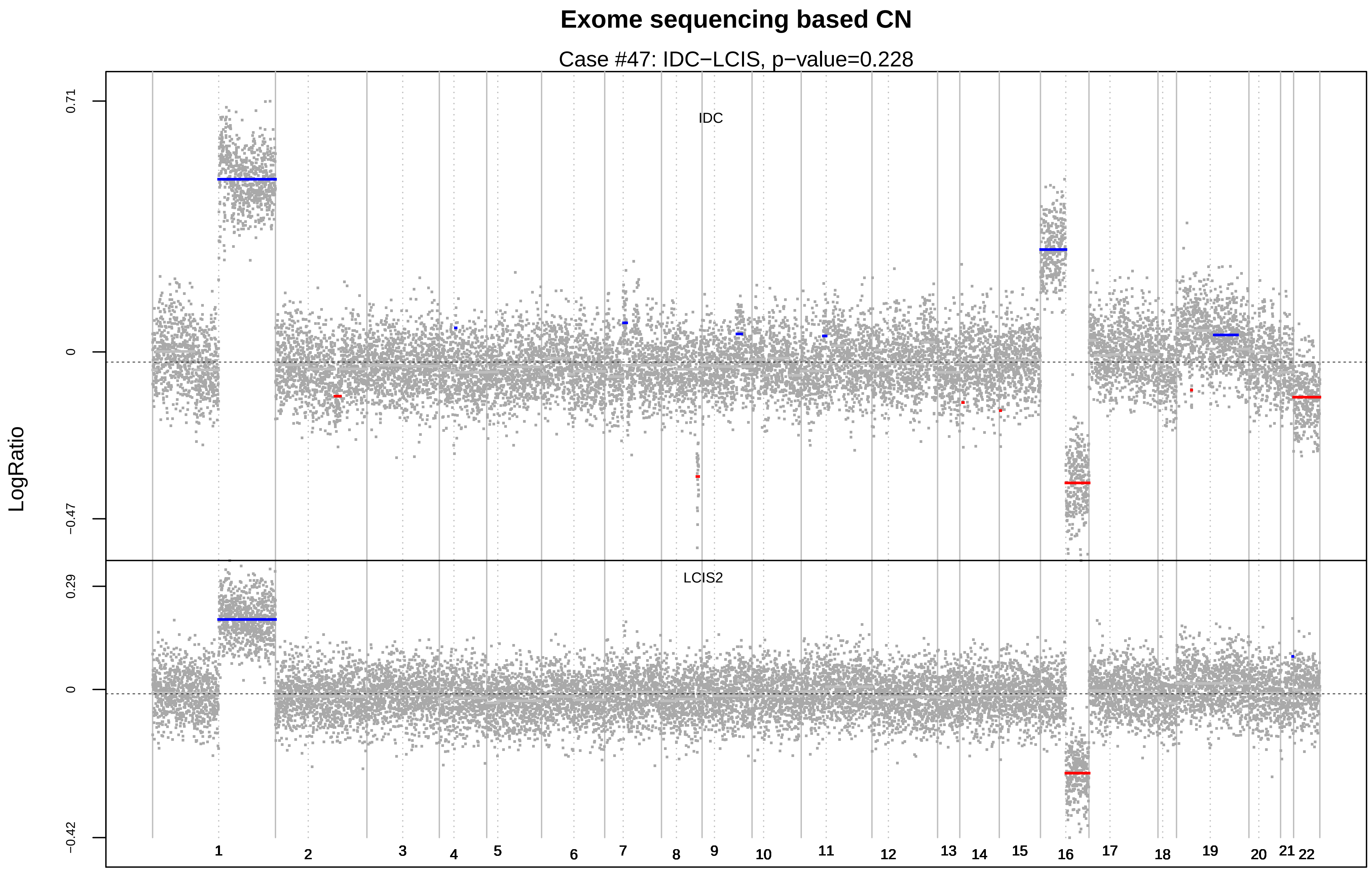

Exome sequencing based CN
Case #47: IDC−LCIS, p−value=0.228
IDC
LCIS2
LogRatio
0.71
0
−0.47
0.29
0
−0.42
1
2
3
4
5
6
7
8
9
10
11
12
13
14
15
16
17
18
19
20
21
22

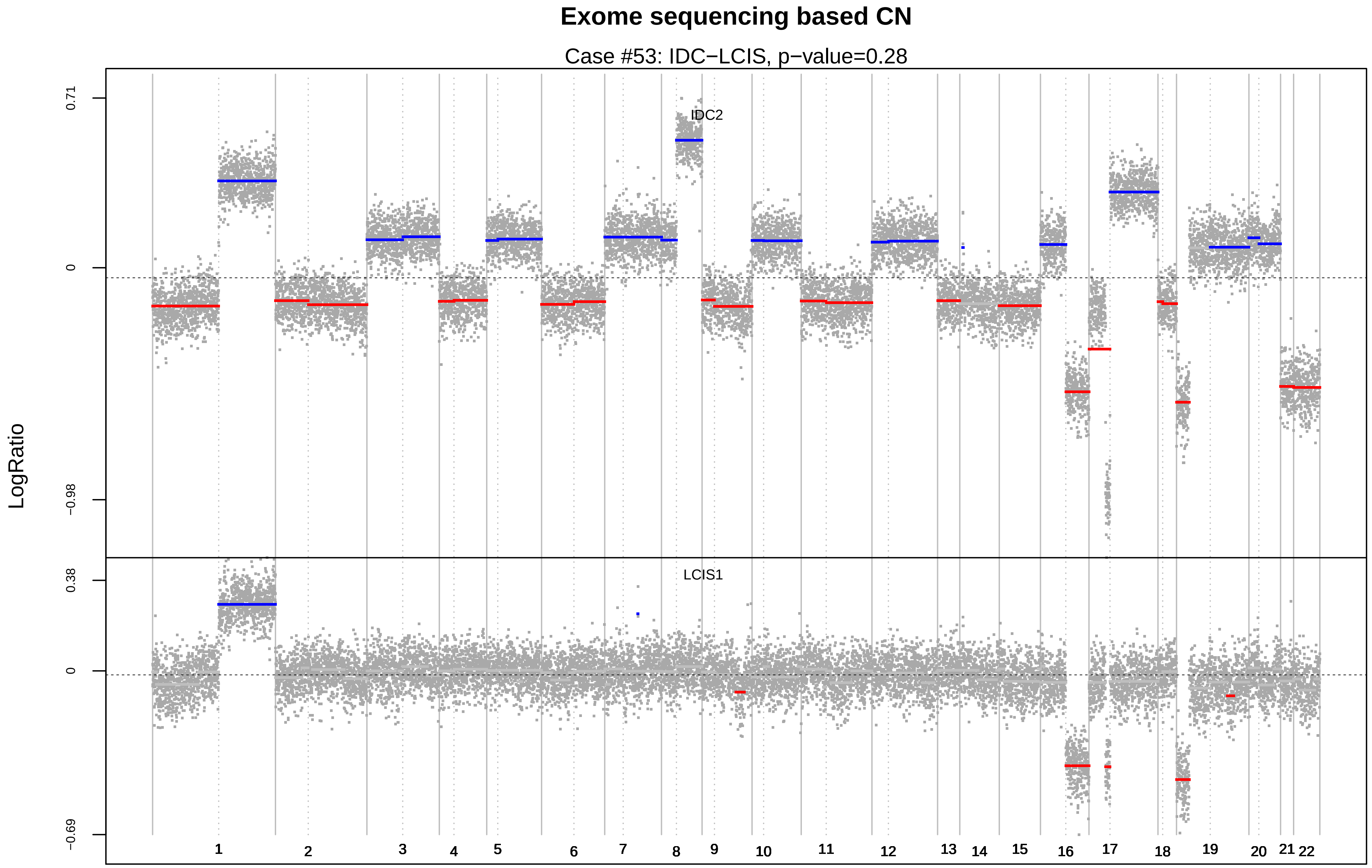
Exome sequencing based CN
Case #53: IDC−LCIS, p−value=0.28
LogRatio
0.71
0
−0.98
IDC2
0.38
0
−0.69
LCIS1
1
2
3
4
5
6
7
8
9
10
11
12
13
14
15
16
17
18
19
20
21
22

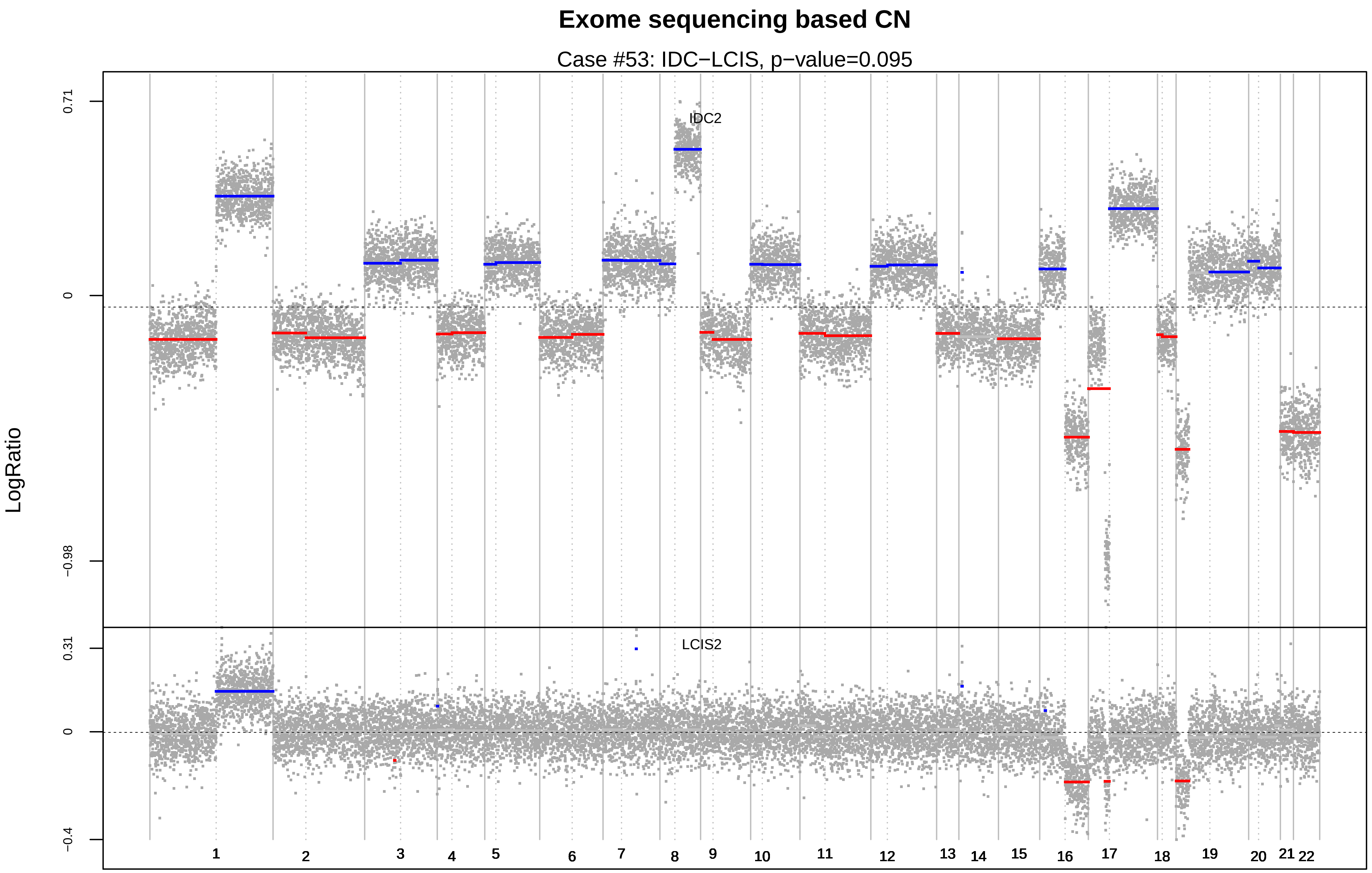

Exome sequencing based CN
Case #53: IDC−LCIS, p−value=0.095
LogRatio
0.71
0
−0.98
IDC2
0.31
0
−0.4
LCIS2
1
2
3
4
5
6
7
8
9
10
11
12
13
14
15
16
17
18
19
20
21
22

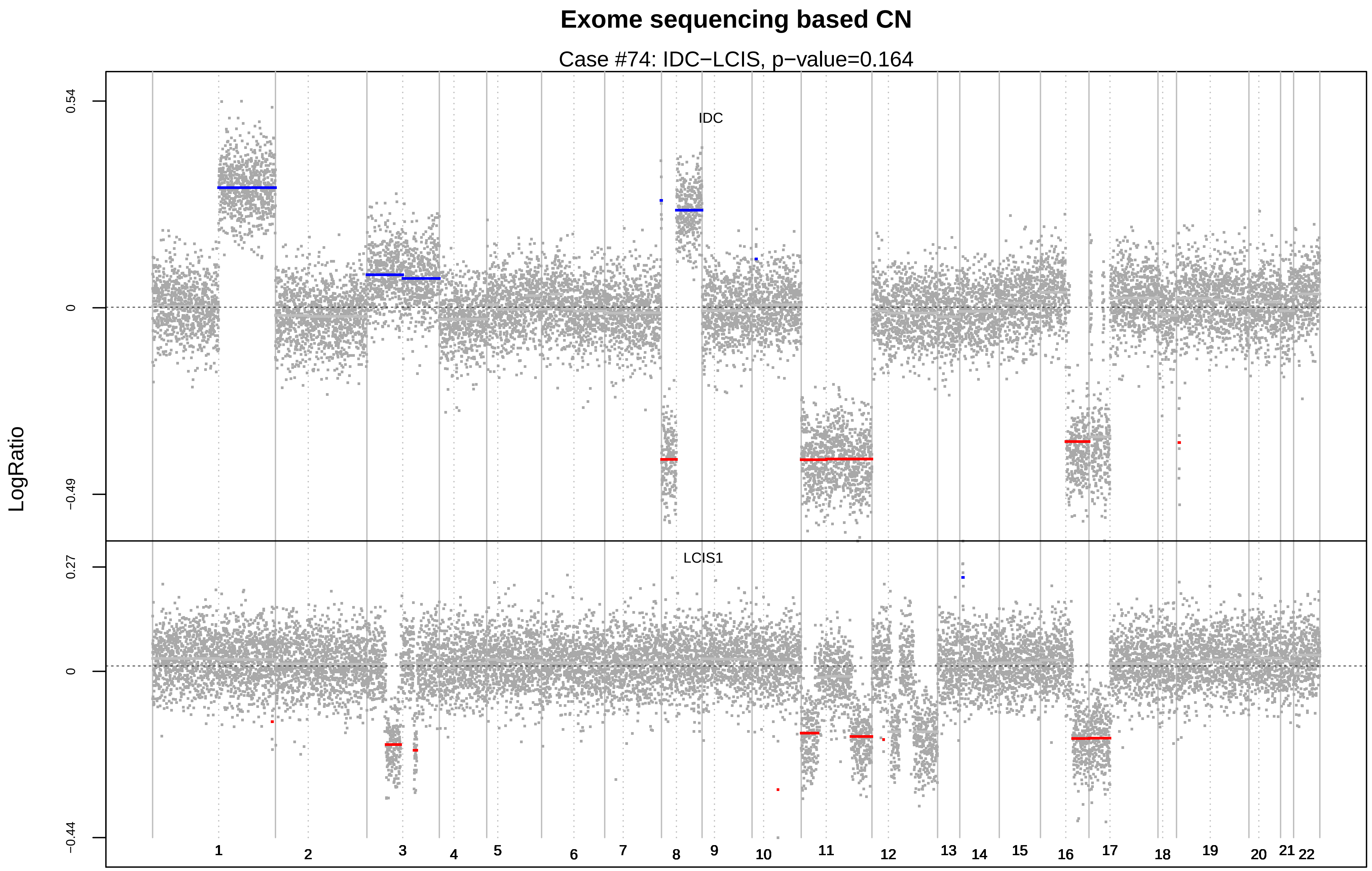
Exome sequencing based CN
Case #74: IDC−LCIS, p−value=0.164
IDC
LCIS1
LogRatio
0.54
0
−0.49
0.27
0
−0.44
1
2
3
4
5
6
7
8
9
10
11
12
13
14
15
16
17
18
19
20
21
22

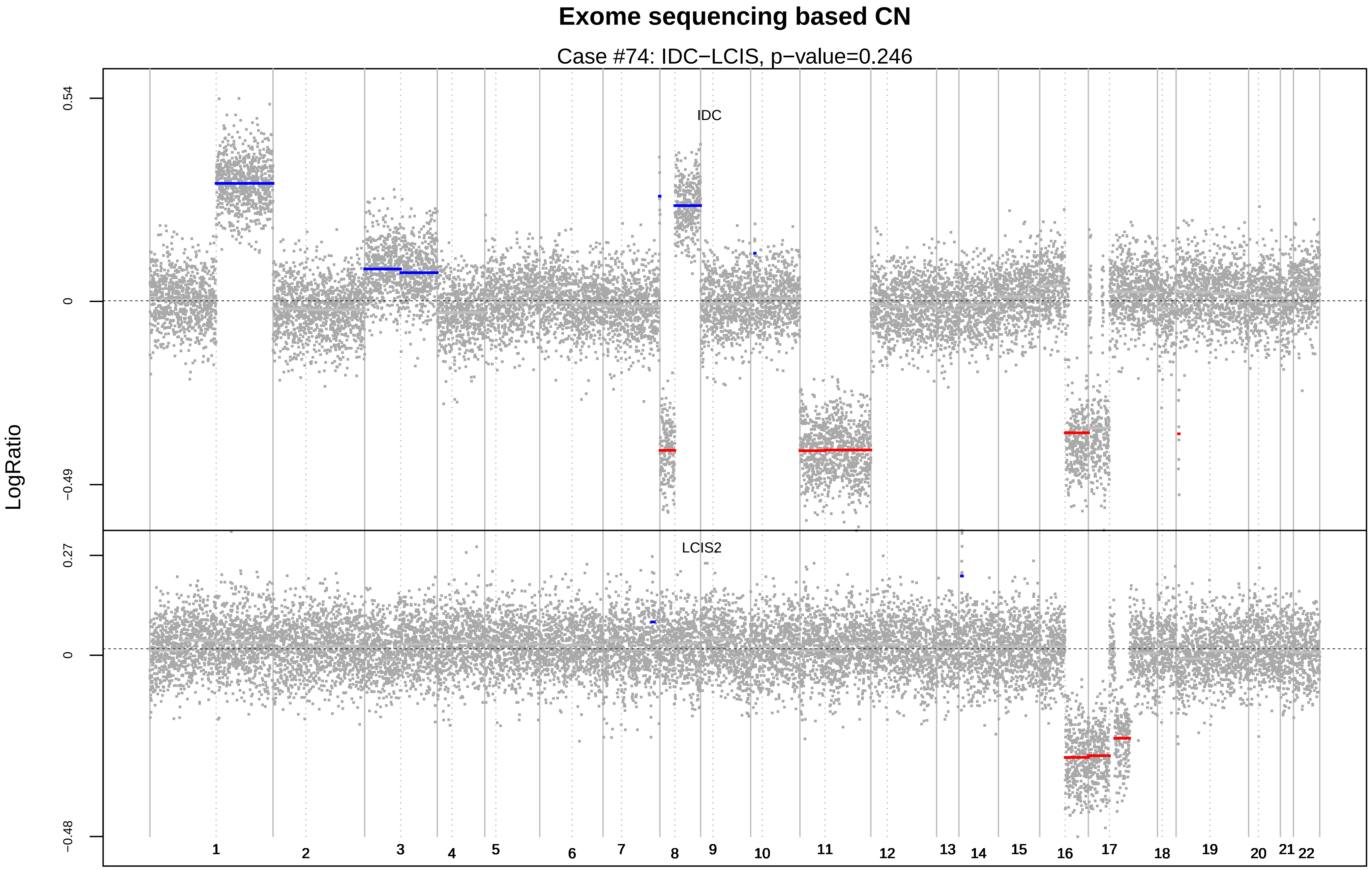
Exome sequencing based CN
Case #74: IDC−LCIS, p−value=0.246
LogRatio
0.54
0
−0.49
IDC
0.27
0
−0.48
LCIS2
1
2
3
4
5
6
7
8
9
10
11
12
13
14
15
16
17
18
19
20
21
22

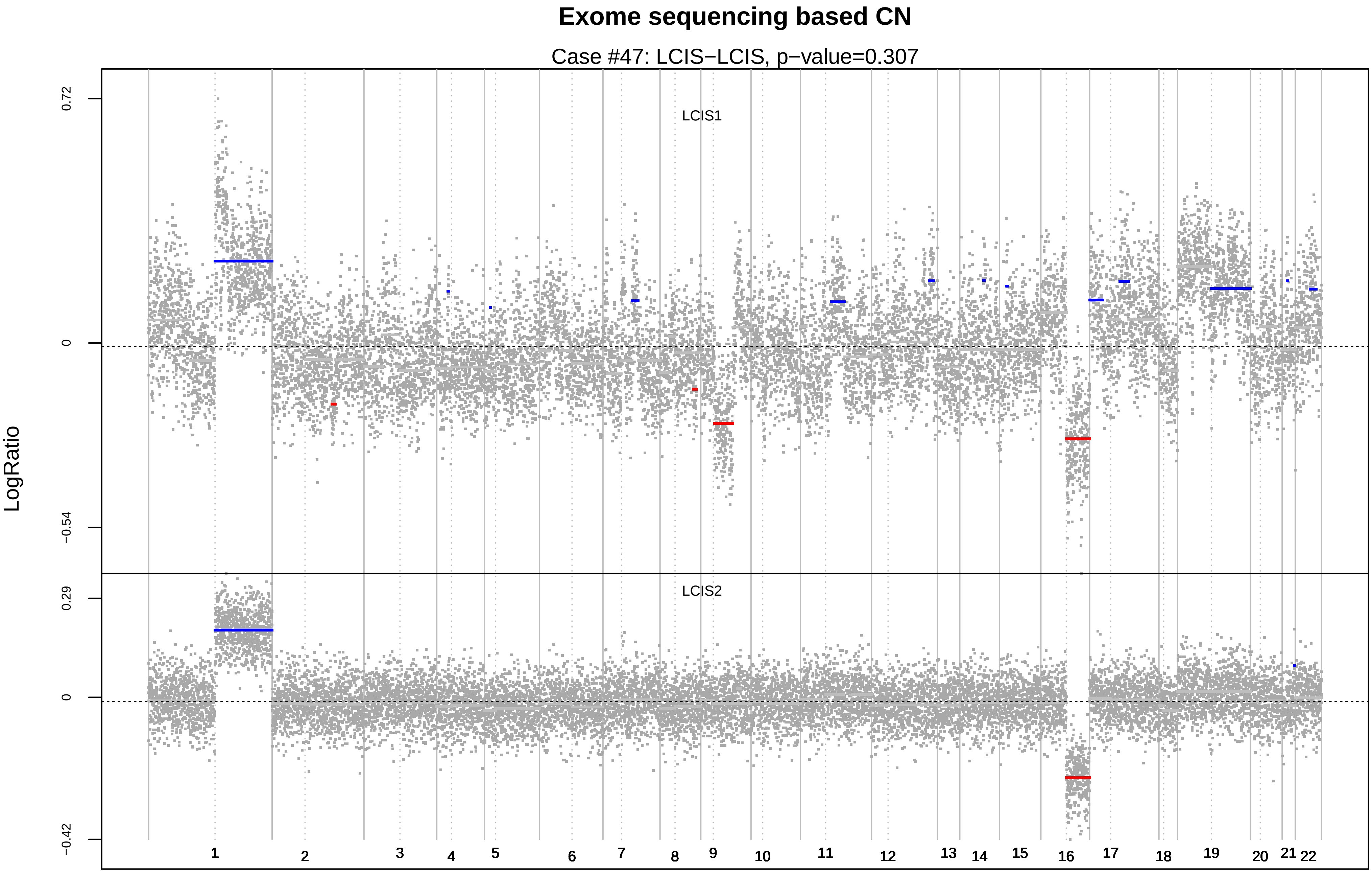

Exome sequencing based CN
Case #47: LCIS−LCIS, p−value=0.307
LogRatio
LCIS1
LCIS2
0.72
0
−0.54
0.29
0
−0.42
1
2
3
4
5
6
7
8
9
10
11
12
13
14
15
16
17
18
19
20
21
22

Exome sequencing based CN

Case #48: LCIS−LCIS, p−value=0.376

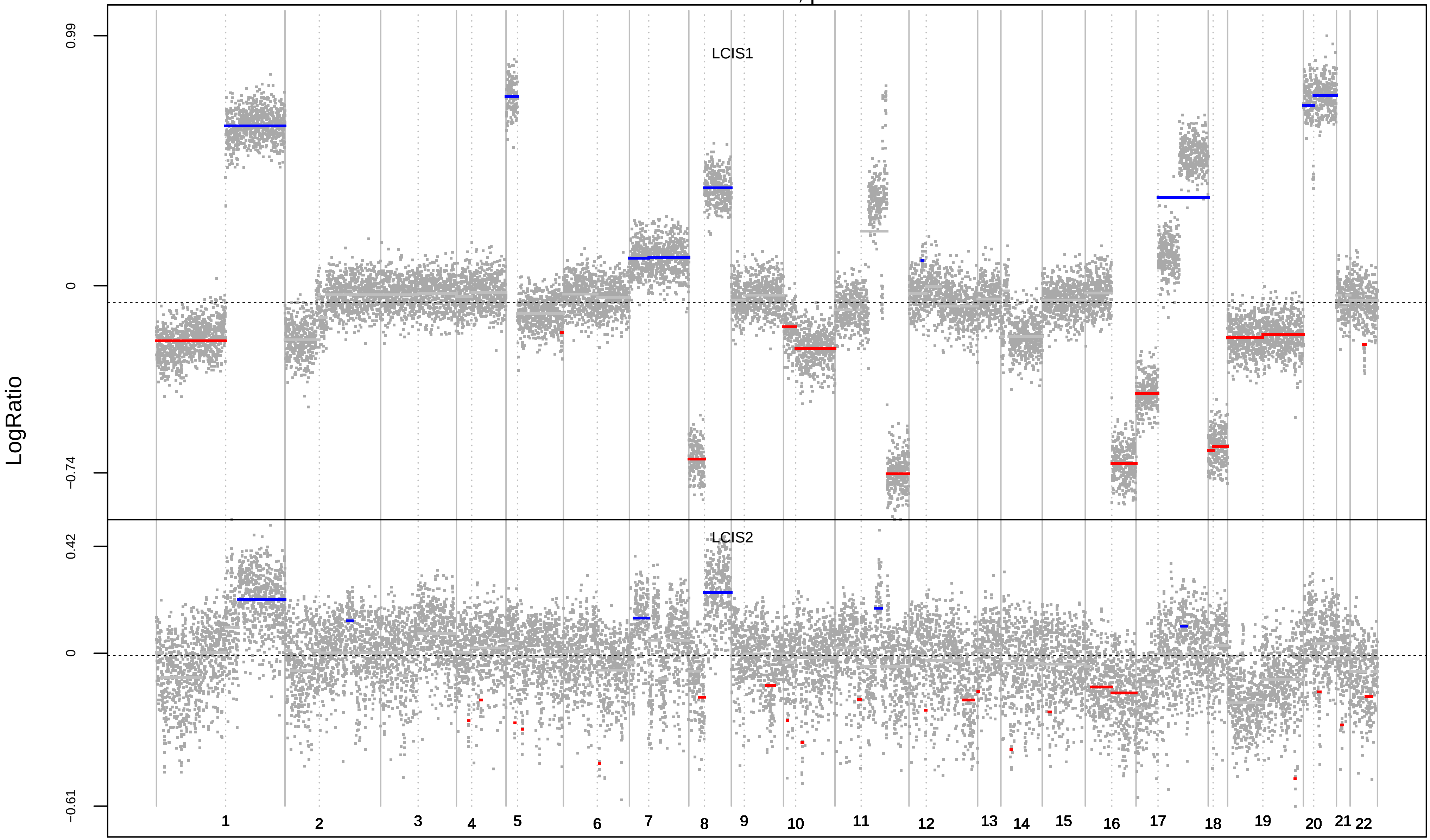

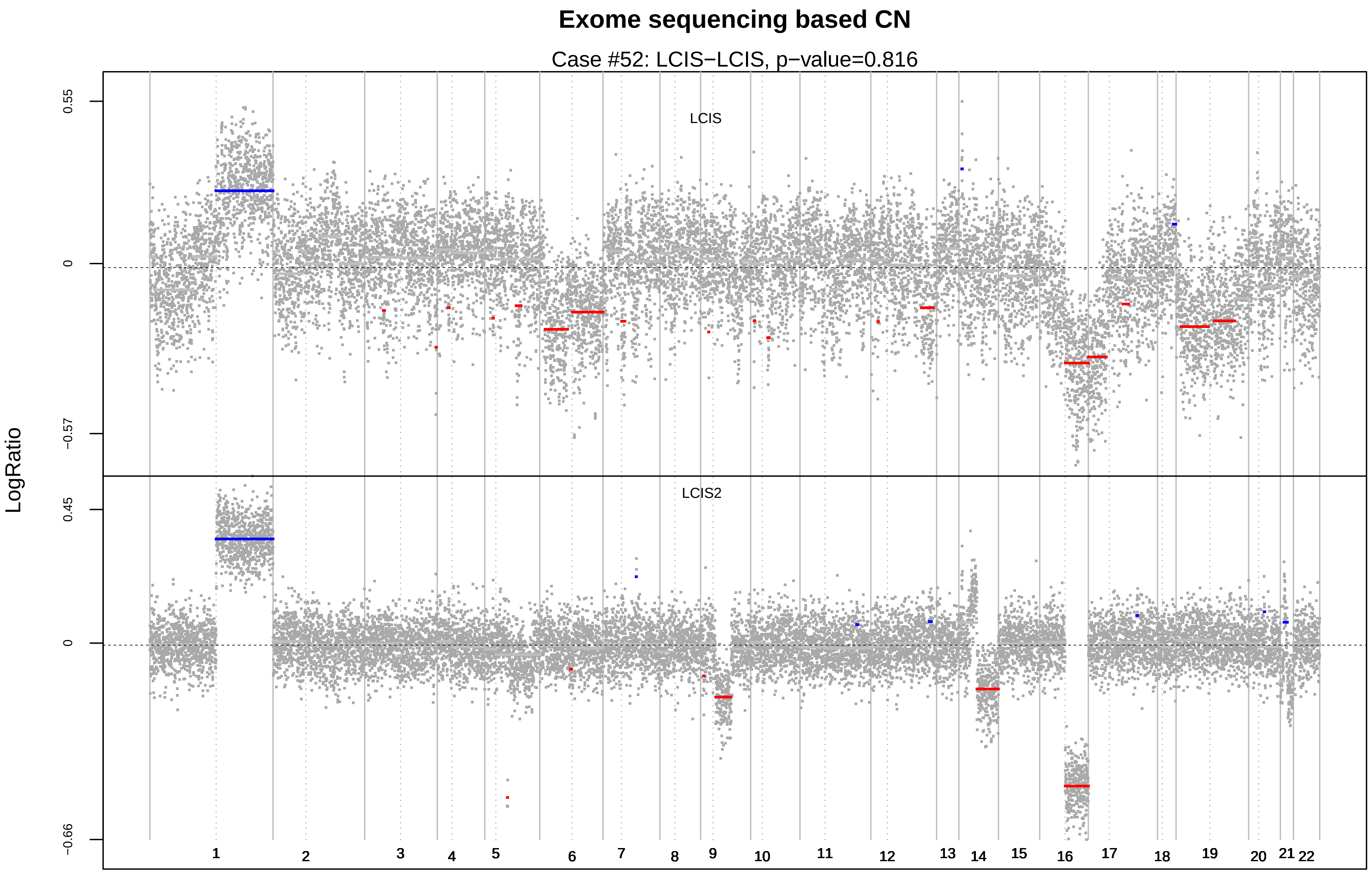

Exome sequencing based CN
Case #52: LCIS−LCIS, p−value=0.816
LogRatio
LCIS
LCIS2
0.55
0
−0.57
0.45
0
−0.66
1 2 3 4 5 6 7 8 9 10 11 12 13 14 15 16 17 18 19 20 21 22

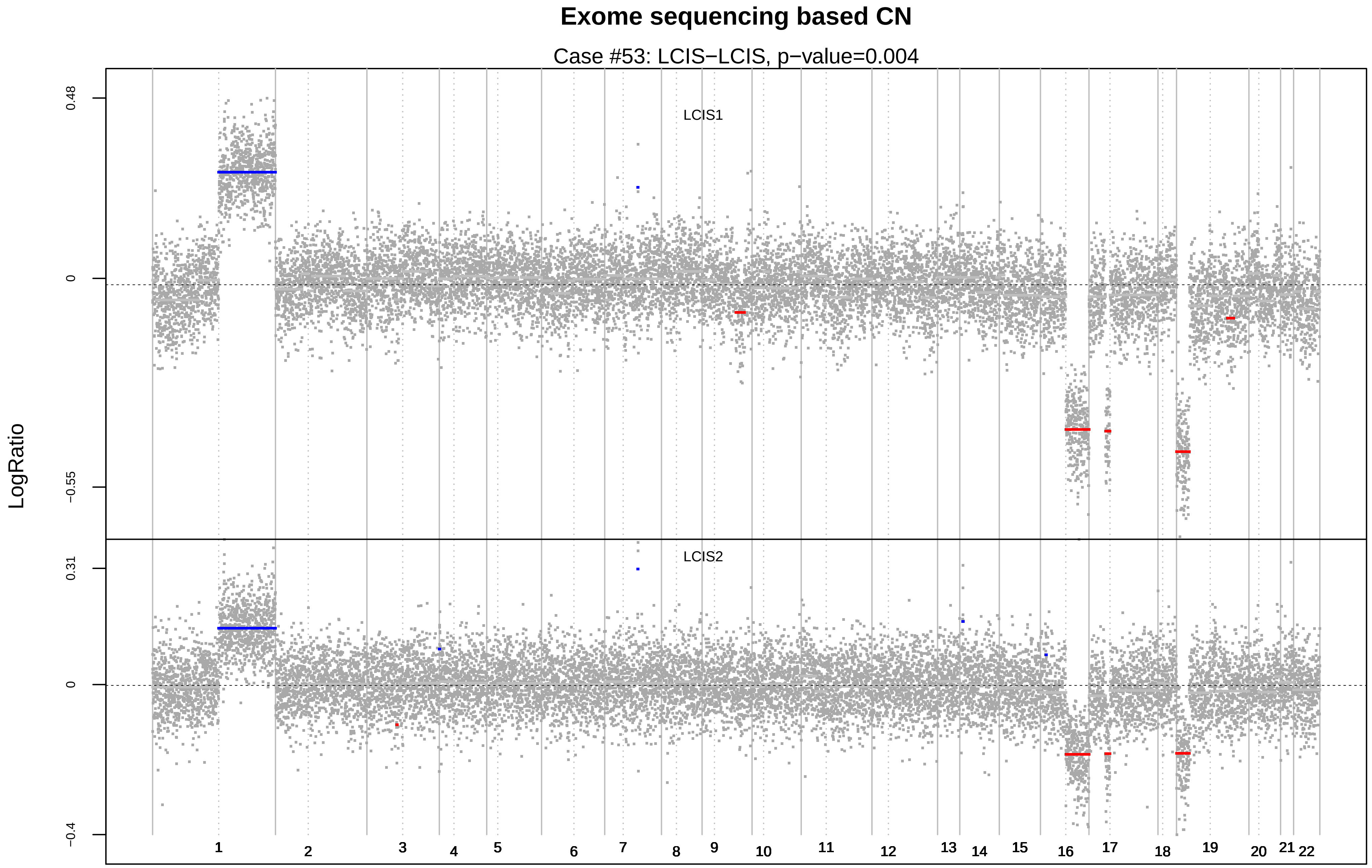

Exome sequencing based CN
Case #53: LCIS−LCIS, p−value=0.004
LogRatio
LCIS1
LCIS2
0.48
0
−0.55
0.31
0
−0.4
1
2
3
4
5
6
7
8
9
10
11
12
13
14
15
16
17
18
19
20
21
22

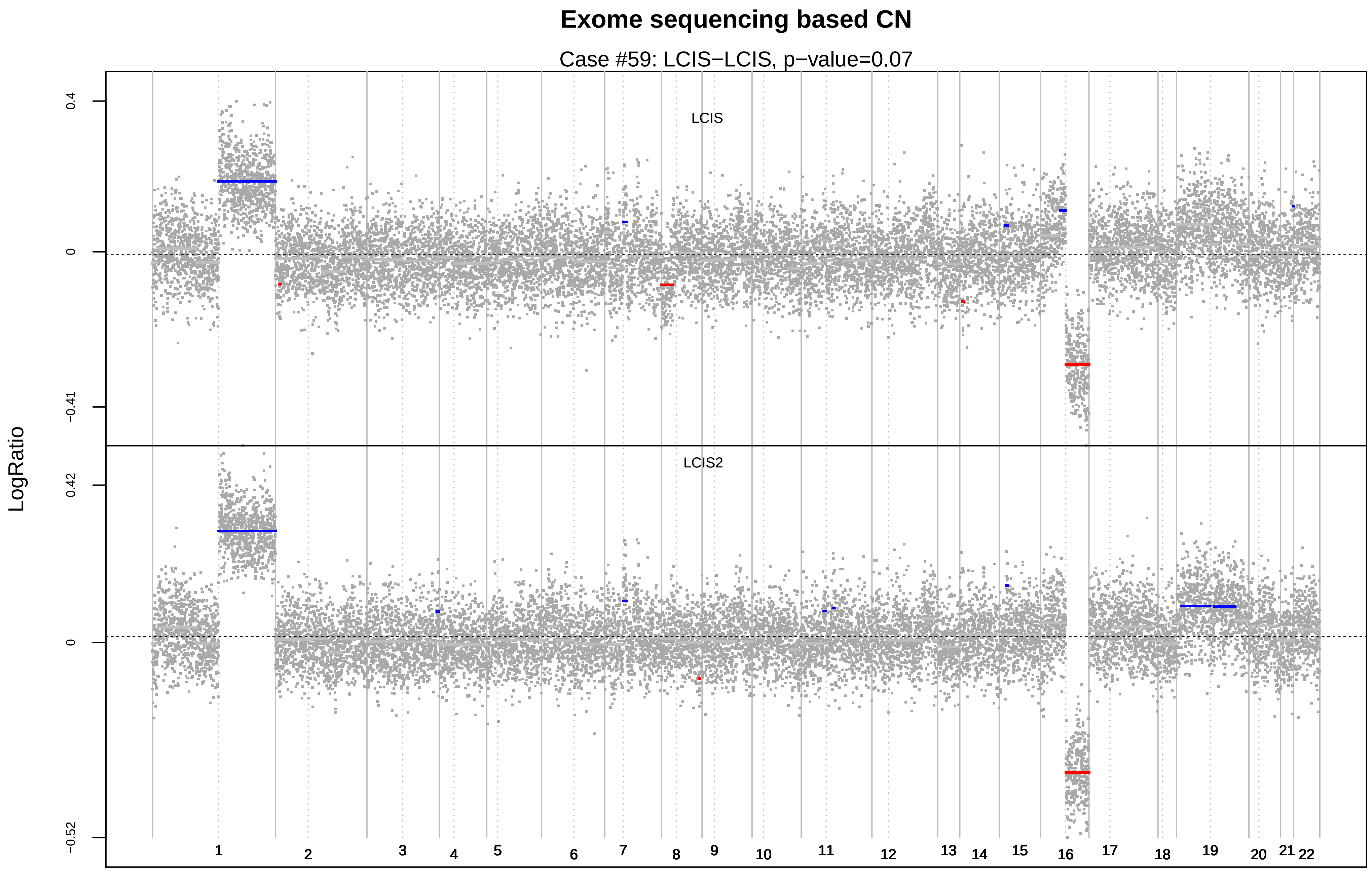

Exome sequencing based CN
Case #59: LCIS−LCIS, p−value=0.07
LogRatio
LCIS
LCIS2
0.4
0
−0.41
0.42
0
−0.52
1
2
3
4
5
6
7
8
9
10
11
12
13
14
15
16
17
18
19
20
21
22

Exome sequencing based CN

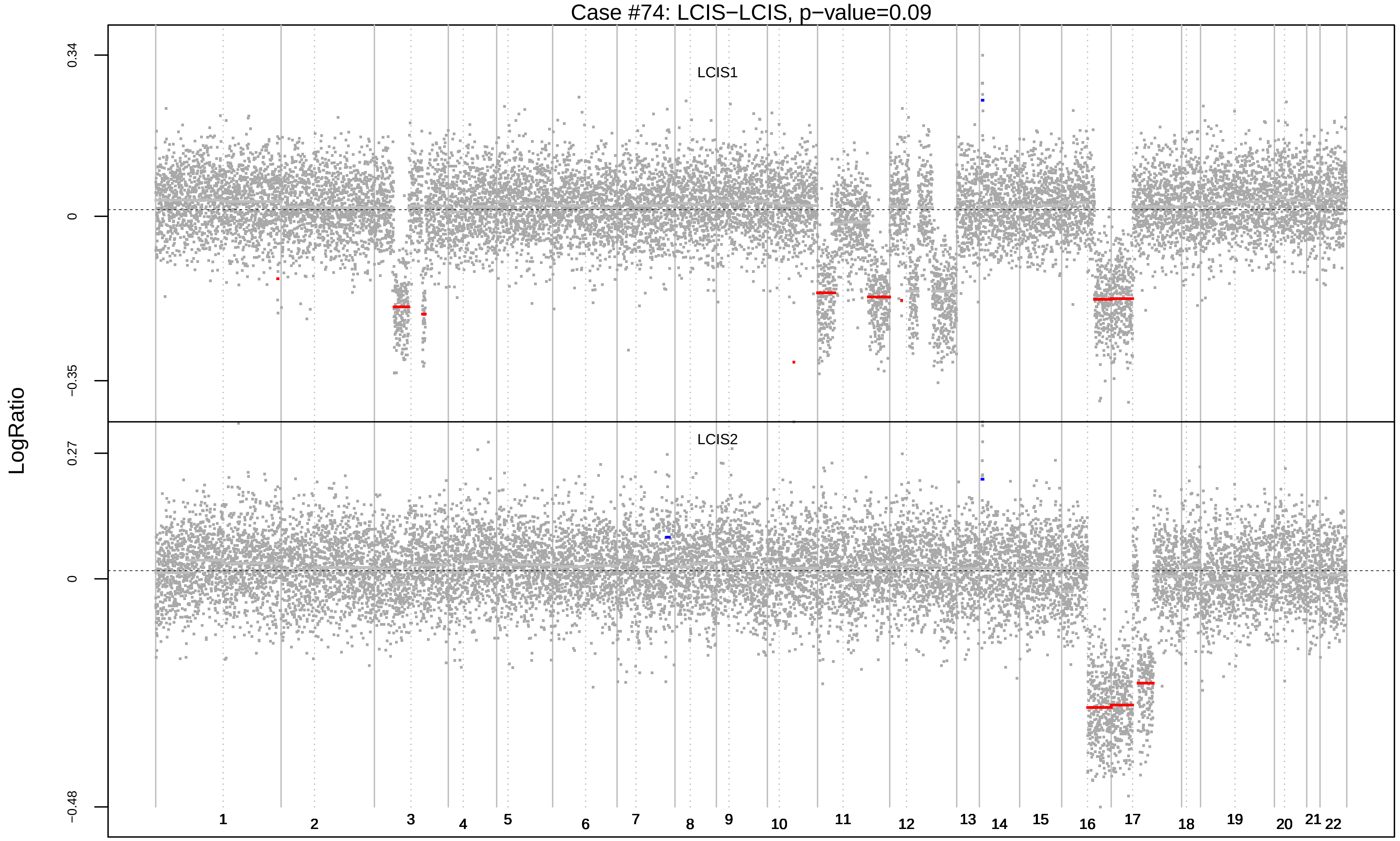

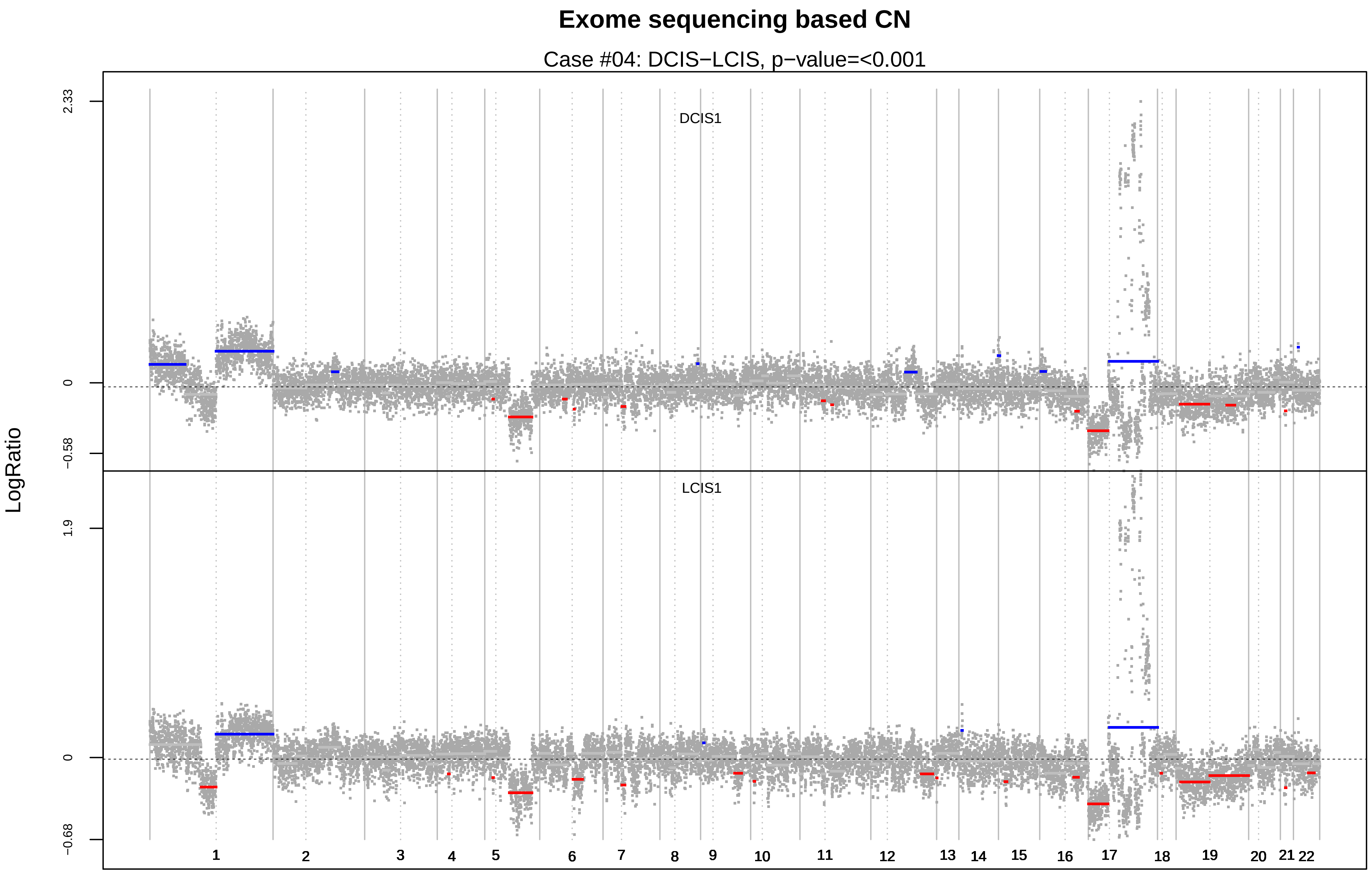
Exome sequencing based CN
Case #04: DCIS−LCIS, p−value=<0.001
DCIS1
LCIS1
LogRatio
2.33
0
−0.58
1.9
0
−0.68
1
2
3
4
5
6
7
8
9
10
11
12
13
14
15
16
17
18
19
20
21
22

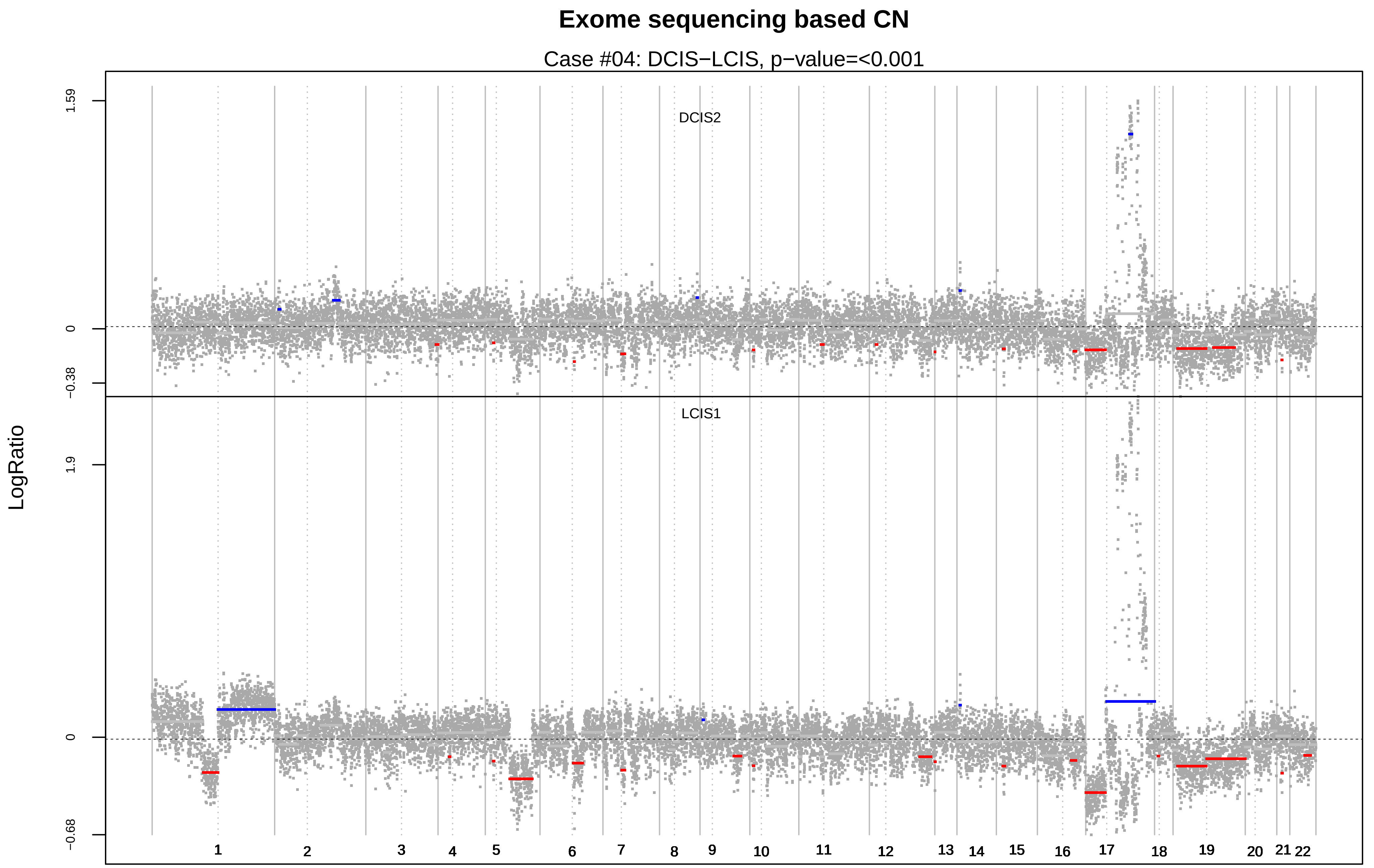
Exome sequencing based CN
Case #04: DCIS−LCIS, p−value=<0.001
DCIS2
LCIS1
LogRatio
1.59
0
−0.38
1.9
0
−0.68
1 2 3 4 5 6 7 8 9 10 11 12 13 14 15 16 17 18 19 20 21 22

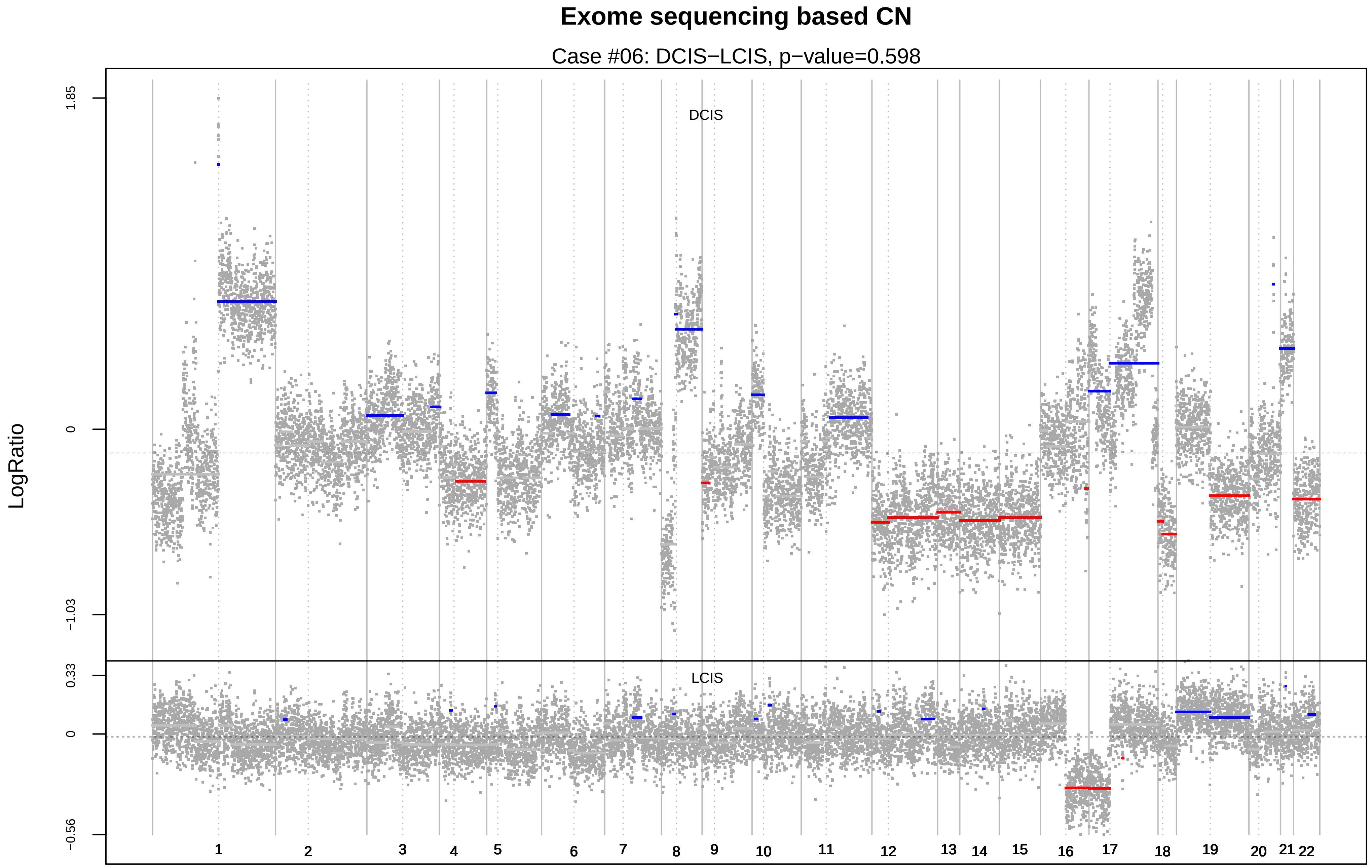

Exome sequencing based CN
Case #06: DCIS−LCIS, p−value=0.598
LogRatio
1.85
0
−1.03
DCIS
0.33
0
−0.56
LCIS
1
2
3
4
5
6
7
8
9
10
11
12
13
14
15
16
17
18
19
20
21
22

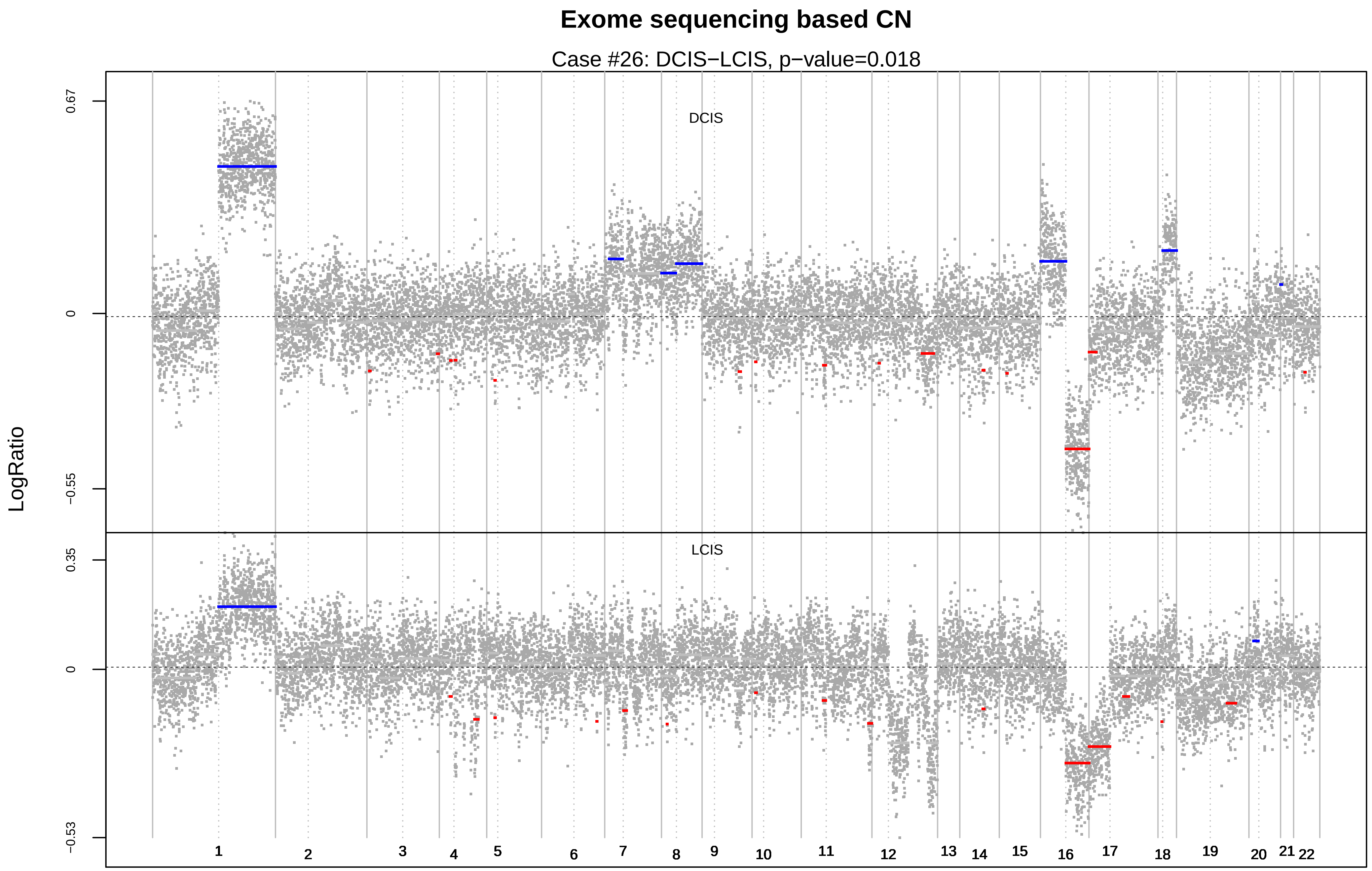
Exome sequencing based CN
Case #26: DCIS−LCIS, p−value=0.018
DCIS
LCIS
LogRatio
0.67
0
−0.55
0.35
0
−0.53
1
2
3
4
5
6
7
8
9
10
11
12
13
14
15
16
17
18
19
20
21
22

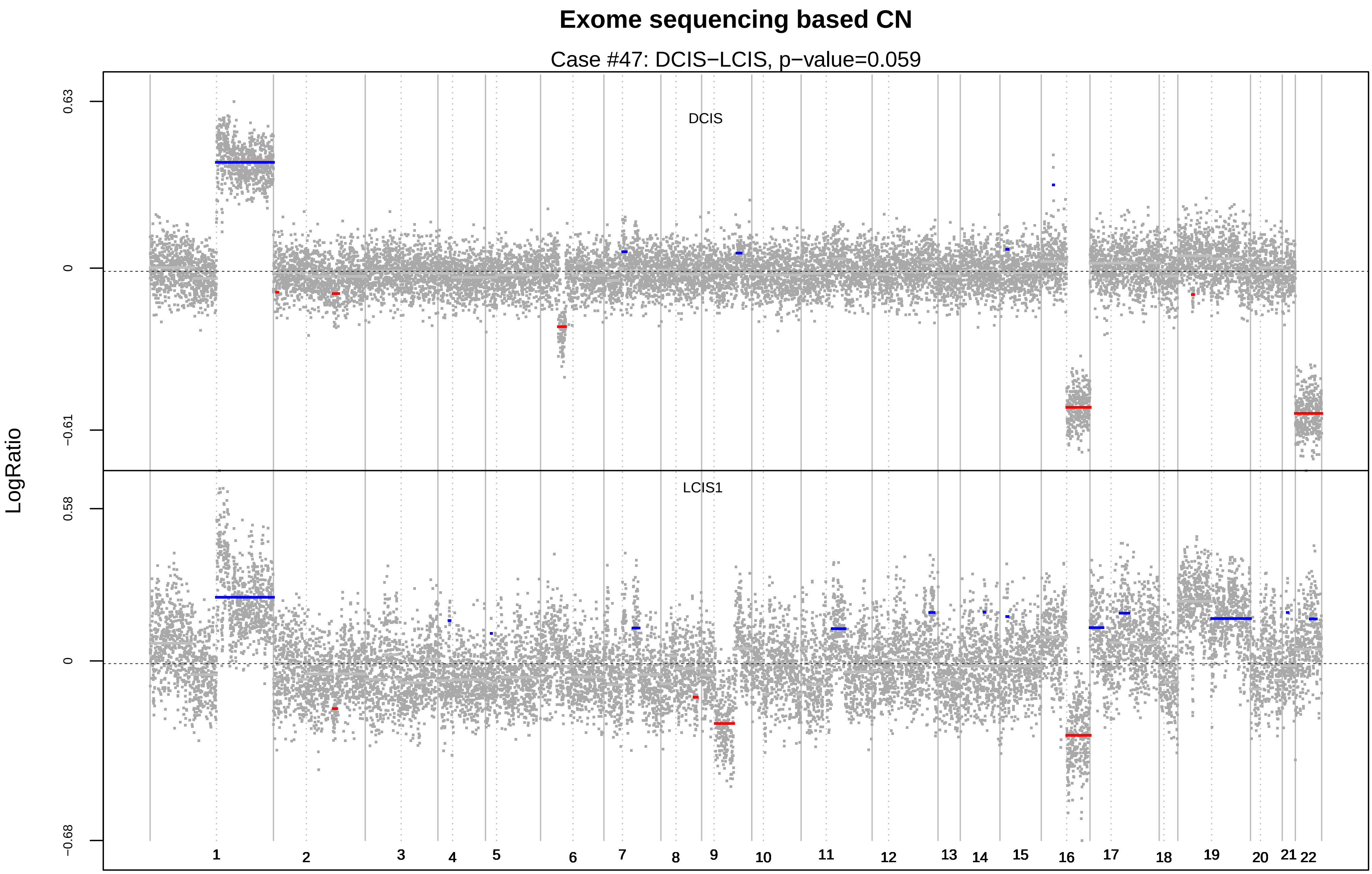
Exome sequencing based CN
Case #47: DCIS−LCIS, p−value=0.059
DCIS
LCIS1
LogRatio
0.63
0
−0.61
0.58
0
−0.68
1
2
3
4
5
6
7
8
9
10
11
12
13
14
15
16
17
18
19
20
21
22

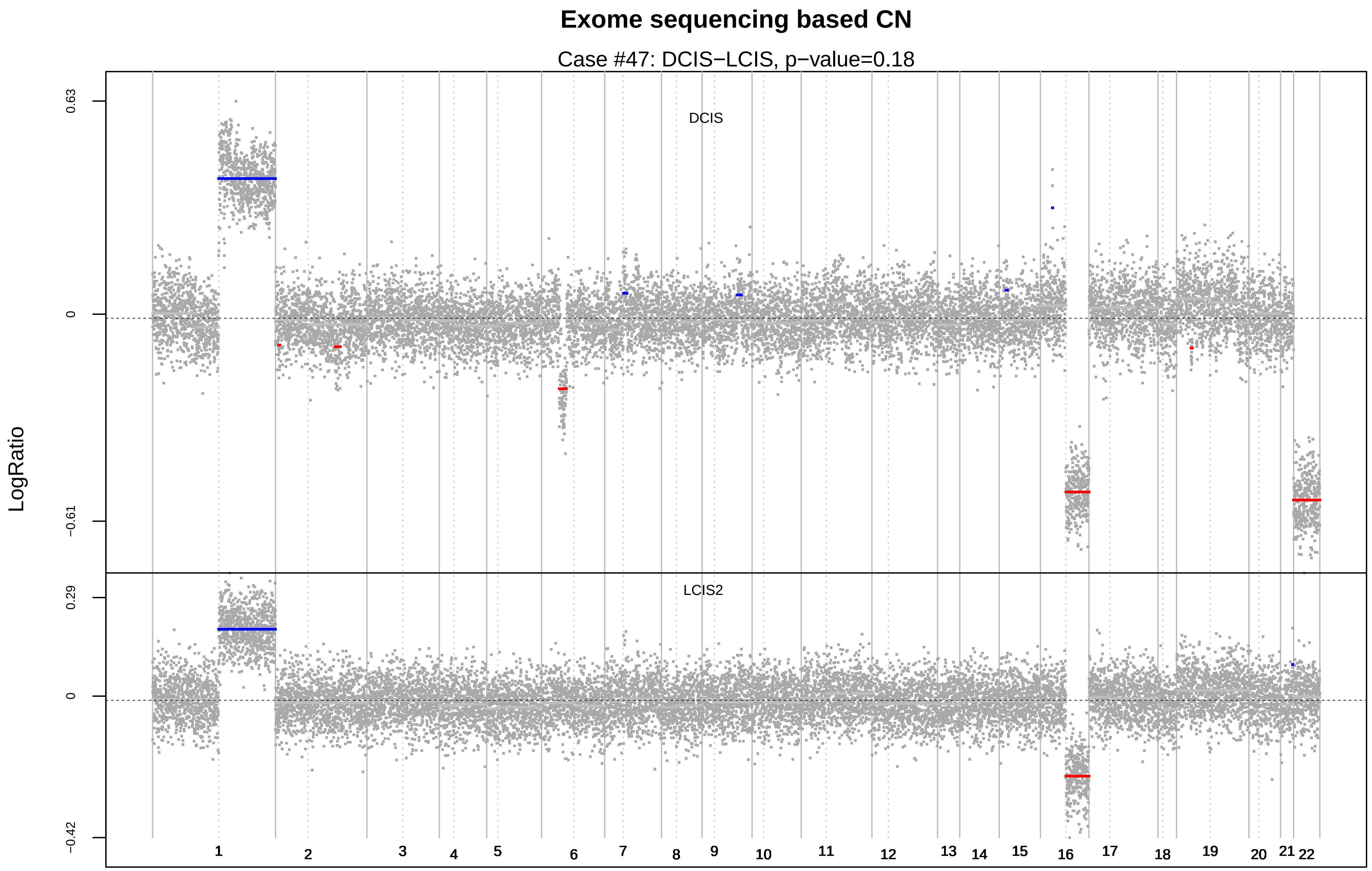

Exome sequencing based CN
Case #47: DCIS−LCIS, p−value=0.18
DCIS
LCIS2
LogRatio
0.63
0
−0.61
0.29
0
−0.42
1
2
3
4
5
6
7
8
9
10
11
12
13
14
15
16
17
18
19
20
21
22

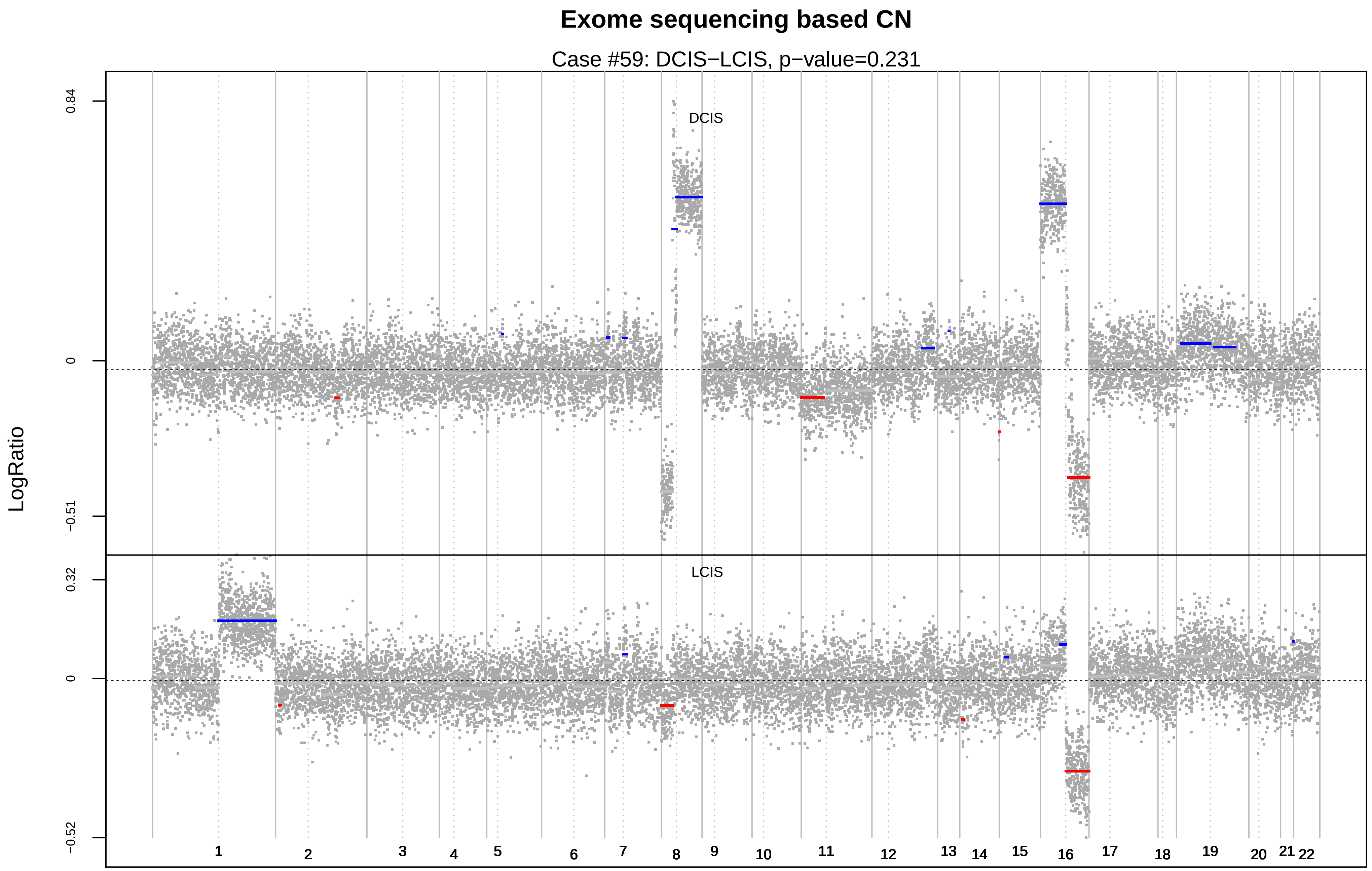

Exome sequencing based CN
Case #59: DCIS−LCIS, p−value=0.231
LogRatio
0.84
0
−0.51
DCIS
0.32
0
−0.52
LCIS
1
2
3
4
5
6
7
8
9
10
11
12
13
14
15
16
17
18
19
20
21
22

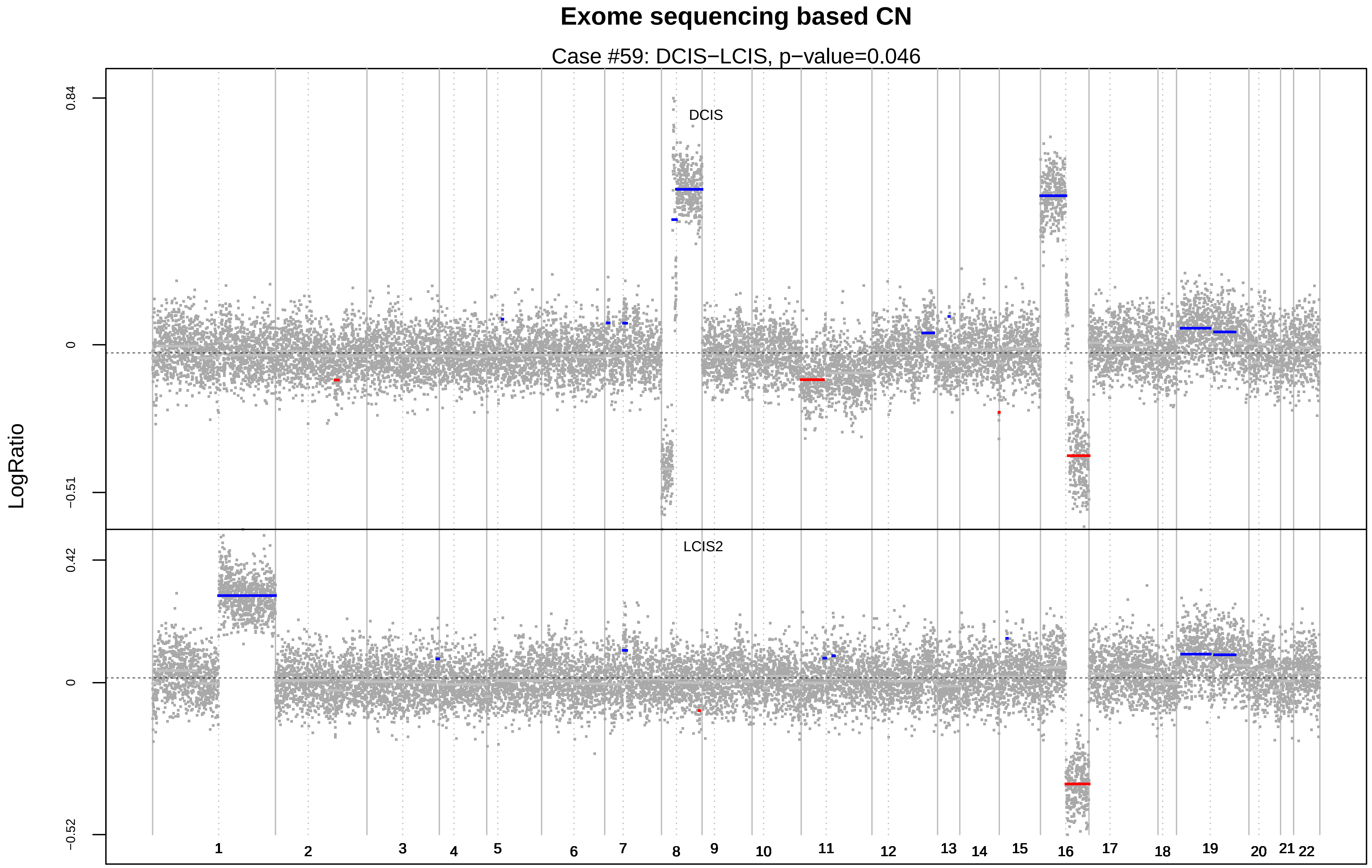

Exome sequencing based CN
Case #59: DCIS−LCIS, p−value=0.046
LogRatio
0.84
0
−0.51
DCIS
0.42
0
−0.52
LCIS2
1
2
3
4
5
6
7
8
9
10
11
12
13
14
15
16
17
18
19
20
21
22

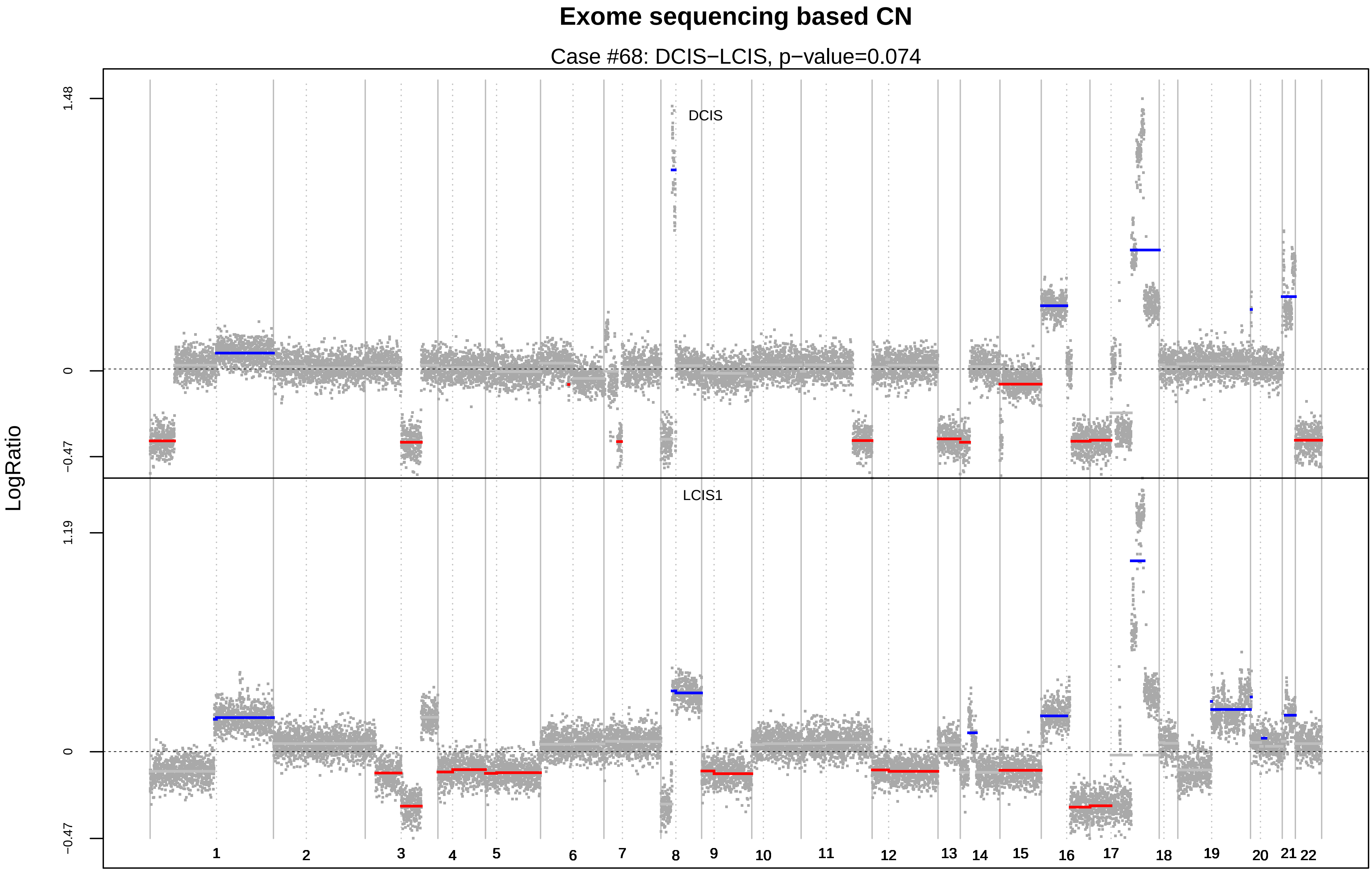

Exome sequencing based CN
Case #68: DCIS−LCIS, p−value=0.074
LogRatio
1.48
0
−0.47
DCIS
1.19
0
−0.47
LCIS1
1
2
3
4
5
6
7
8
9
10
11
12
13
14
15
16
17
18
19
20
21
22
