# Supplementary material for: Clonal relationships between lobular carcinoma in situ and other breast malignancies
Source: Breast Cancer Res. 2016 Jun 23;18:66. doi: 10.1186/s13058-016-0727-z (PMC4918003; doi:10.1186/s13058-016-0727-z)
Supplement: Additional file 4: — This table compares the p values obtained from the clonality tests based on copy number profiling derived using comparative genomic hybridization and the exome arrays. Although there is clearly considerable variation in the actual p values observed, there is consistency in identifying strong clonality signals (p < 0.01), except for case 48 (LCIS2-ILC). (DOCX 28 kb) [file 13058_2016_727_MOESM4_ESM.docx]

**Additional File 4. Comparison for Copy Number Profiling from CGH Arrays Versus Exome Sequencing**

| **Case #^c^** | **Patient Age** | **Lesion Pairs** | **Same Quadrant?** | **Size of Invasive Lesion?** | **Copy Number p-values** | | **Diagnosis^c^** |
| --- | --- | --- | --- | --- | --- | --- | --- |
|  |  |  |  |  | **Exome Arrays^a^** | **CGH Arrays^b^** |  |
| **Panel A: LCIS-ILC** | | | | | | | |
| 13 | 50 | LCIS1-ILC | No | 1.6 | 0.03 | 0.002 | Clonal |
| 24 | 57 | LCIS1-ILC | Yes | 2.1 | <0.001 | <0.001 | Clonal |
| 33 | 65 | LCIS-ILC | Yes | 2.1 | <0.001 | <0.001 | Clonal |
| 38 | 73 | LCIS2-ILC | Yes | Missing | 0.03 | 0.54 | Clonal |
| 47 | 51 | LCIS1-ILC | No | 1.5 | <0.001 | <0.001 | Clonal |
|  |  | LCIS2-ILC | No |  | 0.35 | 0.42 | Independent |
| 48 | 37 | LCIS1-ILC | Yes | 6.0 | 0.06 | <0.001 | Clonal |
|  |  | LCIS2-ILC | No |  | <0.001 | 0.10 | Equivocal |
| 55 | 72 | LCIS-ILC | Yes | 1.3 | 0.02 | 0.002 | Clonal |
| 68 | 48 | LCIS1-ILC | No | 1.4 | 0.29 | 0.58 | Independent |
| 69 | 56 | LCIS-ILC | Yes | 3.0 | 0.004 | 0.001 | Clonal |
| **Panel A: LCIS-IDC** | | | | | | | |
| 47 | 51 | LCIS1-IDC | No | 1.0 | 0.22 | 0.35 | Equivocal |
|  |  | LCIS2-IDC | No |  | 0.23 | 0.48 | Equivocal |
| 53 | 41 | LCIS1-IDC | Yes | 3.7 | 0.28 | 0.40 | Clonal |
|  |  | LCIS2-IDC | Tes |  | 0.10 | 0.62 | Equivocal |
| 74 | 61 | LCIS1-IDC | Yes | 0.75 | 0.16 | 0.50 | Independent |
|  |  | LCIS2-IDC | No |  | 0.25 | 0.73 | Independent |
| **Panel A: LCIS-LCIS** | | | | | | | |
| 47 | 51 | LCIS1-LCIS2 | Yes | N/A | 0.31 | 0.59 | Clonal |
| 48 | 37 | LCIS1-LCIS2 | No | N/A | 0.38 | 0.59 | Independent |
| 52 | 50 | LCIS-LCIS2 | Yes | N/A | 0.82 | 0.02 | Clonal |
| 53 | 41 | LCIS1-LCIS2 | Yes | N/A | 0.004 | 0.005 | Clonal |
| 59 | 47 | LCIS-LCIS2 | No | N/A | 0.07 | 0.10 | Clonal |
| 74 | 61 | LCIS1-LCIS2 | No | N/A | 0.09 | 0.02 | Independent |
| **Panel A: LCIS-DCIS** | | | | | | | |
| 04 | 44 | LCIS1-DCIS1 | Yes | N/A | <0.001 | <0.001 | Clonal |
|  |  | LCIS1-DCIS2 | No | N/A | <0.001 | <0.001 | Clonal |
| 06 | 57 | LCIS-DCIS | Yes | N/A | 0.60 | 0.81 | Clonal |
| 26 | 54 | LCIS-DCIS | No | N/A | 0.02 | 0.14 | Independent |
| 47 | 51 | LCIS1-DCIS | Yes | N/A | 0.06 | 0.71 | Clonal |
|  |  | LCIS2-DCIS | Yes | N/A | 0.18 | 0.03 | Clonal |
| 59 | 47 | LCIS-DCIS | Yes | N/A | 0.23 | 0.74 | Clonal |
|  |  | LCIS2-DCIS | No | N/A | 0.05 | 0.67 | Clonal |
| 68 | 48 | LCIS1-DCIS | Yes | N/A | 0.07 | 0.03 | Clonal |

^a^ Copy number comparisons derived from the exome sequencing data.

^b^ Copy number comparisons derived using Agilent comparative genomic hybridization.

^c^ Cases were included in this table only if good quality data were available from both comparative genomic hybridization and exome sequencing.

^d^ Calls from Table 1, including information on mutation profiling. A lesion pair was designated as clonal if p<0.01 from either the comparison of mutational profiles or the comparison of copy number profiles derived from comparative genomic hybridization. The “equivocal” designation was made if the smaller p-value was in the range 0.01-0.05.
